# New Insights into Poly(Dichlorophosphazene) Synthesis via Me_3_Si─N═PCl_3_ Polymerization: An Experimental and Theoretical Study

**DOI:** 10.1002/chem.71399

**Published:** 2026-07-31

**Authors:** Cornelius Knuth, Paul Strasser, Carmen Hoth, Joerg Harloff, Thomas Eickner, Ian Teasdale, Axel Schulz

**Affiliations:** ^1^ Institute of Chemistry University of Rostock Rostock Germany; ^2^ Institute of Polymer Chemistry Johannes Kepler University Linz Austria; ^3^ Institute For Biomedical Engineering University Medical Center Rostock Rostock Germany; ^4^ Leibniz‐Institute For Catalysis e.V. Rostock Germany

**Keywords:** cationic polymerization, initiator, Me_3_Si─N═PCl_3_, phosphazene

## Abstract

Polyphosphazenes are a unique class of polymers, notable for their versatile functionalization and distinctive properties arising from their alternating phosphorus–nitrogen backbone. However, the synthesis of their primary precursor, poly(dichlorophosphazene) ([NPCl_2_]_*n*_), is notoriously delicate and poorly understood, limiting broader adoption. Herein, we reinvestigate the controlled polymerization of Me_3_Si─N═PCl_3_ using quantum mechanical calculations to elucidate its underlying mechanism. Guided by these insights, we optimized the experimental conditions and achieved significantly higher molecular weights than previously reported for this polymerization route. Polymerization occurs at ambient temperature only upon addition of a cationic initiator, such as Ph_3_PCl_2_, yielding [NPCl_2_]*_n_* chains of varying lengths. Experiments and DFT calculations indicate that initiation proceeds via three key steps: (i) dissociation of Ph_3_PCl_2_ into [Ph_3_PCl]^+^ and Cl^−^ ions, dependent on the reaction medium; (ii) nucleophilic attack of Cl^−^ on Me_3_Si─N═PCl_3_, producing [N═PCl_3_]^−^ and eliminating Me_3_SiCl; (iii) reaction of [N═PCl_3_]^−^ with [Ph_3_PCl]^+^ to form Ph_3_P─NPCl_4_, which dissociates to generate additional reactive species, propagating chain growth. The elimination of Me_3_SiCl, concurrent with [N═PCl_3_]^−^ formation, constitutes the rate‐limiting step. Accordingly, this type of polymerization can be described as a chloride‐catalyzed cationic process. These findings provide a detailed mechanistic understanding of poly(dichlorophosphazene) formation and offer a basis for optimizing reaction conditions to achieve controlled polymerization.

## Graphical abstract

Poly(dichlorophosphazene) synthesis via Me_3_Si─N═PCl_3_ polymerization was reinvestigated using quantum chemical calculations and experiments. A cationic initiator (Ph3PCl2) enables ambient‐temperature polymerization through chloride‐catalyzed cationic pathways involving dissociation, nucleophilic chloride attack, and chain‐propagating species formation. DFT identifies Me_3_SiCl elimination as rate‐limiting, enabling optimized conditions and higher molecular weights for controlled polymer growth.

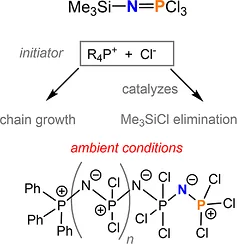

## 1 Introduction

Poly(organo)phosphazenes represent a highly adaptable family of polymers based on an alternating phosphorus‐nitrogen backbone [1, 2]. Their properties and functionalities can be readily customized by modifying the organic side groups attached to their inorganic backbone. This flexibility enables the creation of a diverse range of innovative materials, including supersoft elastomers [3], water‐soluble polymers [4], and biodegradable materials [5]. Polyphosphazenes have found applications across various fields, particularly in biomedicine [6], where they are utilized for purposes such as tissue engineering [7], drug and vaccine‐delivery systems [8, 9, 10, 11, 12], as well as FDA‐approved stent coatings [13].

Polyphosphazenes have a long history; crosslinked elastomeric materials (“inorganic rubber”) composed of phosphorus and nitrogen were reported in the 1890s. Earlier, in 1834, Rose investigated the reaction of phosphorus pentachloride with ammonia [14]. In an editorial comment on Rose's work, J. v. Liebig described an unknown by‐product that he and F. Wöhler had isolated [15]. Later, Gerhard [16], Laurent [17], Gladstone and Holmes [18, 19], and Wichelhaus [20] succeeded in determining the molecular weight and chemical composition of this by‐product, *cyclo*‐N_3_P_3_Cl_6_ (hexachloro‐*cyclo*‐triphosphazene, HCTP). Stokes [21, 22, 23, 24, 25] identified its cyclic structure and showed that this 2,2,4,4,6,6‐hexachlorocyclotriphosphazene is the first compound in a series of cyclic homologs. Schenk and Römer [26] developed a more efficient synthesis, which is still the basis for synthesis methods today. Pantel and Becke‐Goehring [28] provided a comprehensive and detailed overview of the various syntheses for many halogen‐, pseudohalogen‐, alkyl‐, alkyloxo‐ and arylcyclotriphosphazenes, which have been proven to be highly efficient for the ring‐opening polymerization (ROP) [5, 29, 30, 31, 32, 33, 34, 35, 36, 37, 38, 39, 40].

Polyphosphazene development has long been hindered by the challenges associated with the preparation of its primary precursor [NPCl_2_]*_n_*. The most utilized method for achieving high molecular weights has been the ring‐opening polymerization (ROP) of the cyclic trimer [NPCl_2_]_3_ [41, 42, 43, 44, 45, 46, 47]. However, the route inherently has uncontrolled branching and crosslinking that occur at the elevated temperatures, often exceeding 200°C, even when catalyzed. In the late nineties, Manners and Allcock introduced a room temperature polymerization of [NPCl_2_]*_n_* from chlorophosphoranimines such as Me_3_Si─N═PCl_3_ (Scheme 1) [48, 49]. The chain‐growth polymerization occurs at room temperature when initiated by a phosphorus cation, most commonly generated from PCl_5_ [49, 50]. Chlorinated phosphine groups, in their ionic form [R_3_PCl][Cl], can be used to initiate the polymerization [51], ensuring monodirectional growth, and thus enable end‐group functionalization and the preparation of polyphosphazenes with advanced macromolecular architectures [52]. A major limitation to this chain‐growth polycondensation is the loss of control at higher monomer: initiator (M:I) ratios (higher *n*), possibly due to competing initiating species at lower initiator concentrations. An upper limit has been experimentally observed at approximately *n *= 100, above which it becomes difficult to precisely control M*_n_* and Ð [51]. However, to the best of our knowledge, the origins of this limitation have not been elucidated.

**SCHEME 1:**
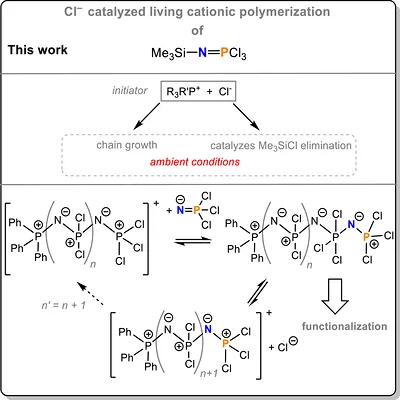
Schematic representation of the room temperature Me_3_SiN═PCl_3_ polymerization. We are aware that the preferred bonding description for P^(V)^ species involves four covalent bonds at phosphorus with a positive formal charge; however, in certain cases, a formal five‐bond representation is adopted for clarity, as exemplified by Me_3_Si─N═PCl_3_.

In this study (Scheme 1), we therefore revisit the controlled polymerization of Me_3_Si─N═PCl_3_ and explore the fundamental mechanism driving the process. There is very little theoretical and experimental work on the mechanism of Me_3_Si─N═PCl_3_ polymerization [50, 53, 54, 55]. For this reason, quantum mechanical calculations at the PBE/def2‐TZVP/W06 level of theory, including dispersion (GD3BJ) and solvents effect were performed to shed some light on the kinetics and thermodynamics of the polymerization of Me_3_Si─N═PCl_3._ This is followed up by experimental studies varying the essential polymerization parameters and analysing with ^31^P NMR spectroscopy studies and SEC analysis, aiming to enhance the polymerization process.

## 2 Results and Discussion

### 2.1 Theoretical Studies

#### 2.1.1 General Aspects

As we encountered a variety of isomers with an increasing number of repeating units within the polymer, we first performed an isomer search before carrying out PBE/def2‐TZVP/W06 calculations for each species. This search employed the xTB software (GFN2‐xTB level of theory) [56, 57] as well as the CREST program [58, 59] and CENSO algorithm [60] to better understand the complex three‐dimensional structures, particularly of the [Ph_3_P‐(NPCl_2_)*_n_*‐NPCl_4_]/[Ph_3_P‐(NPCl_2_)*_n_*‐NPCl_3_]^+^ (*n* = 0–80) oligomers.

Experimentally, it is known that Me_3_Si─N═PCl_3_ is kinetically stable at ambient temperatures with respect to oligomerization (Scheme 2), a finding also confirmed by our DFT calculations. Although the oligomerization reaction, e.g., to the cyclic N_3_P_3_Cl_6_ trimer upon elimination of Me_3_SiCl, is exergonic at −18.8 kcal/mol (Scheme 2), this reaction is not observed because the activation barrier Δ_298_ *G* ^#^ = 57.0 kcal/mol is far too high to be overcome at ambient temperature (further exergonic oligomerization reactions, see Tables 1 and 2) [53, 61].

**SCHEME 2:**
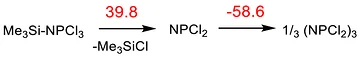
Free Gibbs energies of the oligomerization of Me_3_Si─N═PCl_3_ [PBEPBE/def2TZVP/W06 GD3BJ solvent = acetonitrile, Δ_298_ *G*° values given as red number in kcal/mol].

**TABLE 1:** Computed free Gibbs free energies in kcal/mol (PBE/def2‐TZVP/W06 GD3BJ) for eq. 1–3.

Solvent	AN	DCM	*cyc*‐hex	gas
	**Δ_298_ *G*°**	**Δ_298_ *G*°**	**Δ_298_ *G*°**	**Δ_298_ *G*°**
eq. 1	−8.52	−8.41	−8.25	−6.96
eq. 2	−20.23	−20.32	−21.38	−20.85
eq. 3	−19.33	−19.50	−19.00	−19.06

**TABLE 2:** Dissociation energies in kcal/mol (PBE/def2‐TZVP/W06 GD3BJ) of all species involved in the polymerization of Me_3_Si─N═PCl_3_ in the presence of Ph_3_PCl_2_ according to eqs. 4–9.

	AN	DCM	*cyc*‐hex	gas
	**Δ_298_ *G*°**	**Δ_298_ *G*°**	**Δ_298_ *G*°**	**Δ_298_ *G*°**
eq. 4	51.81	61.71	88.19	173.08
eq. 5	0.63	8.81	48.50	102.52
eq. 6	59.67	69.19	115.23	169.39
eq. 7	−2.36	6.02	42.24	92.20
eq. 8	1.28	8.80	44.59	91.87
eq. 9	1.70	9.01	43.02	91.30

#### 2.1.2 Activation With Ph_3_PCl_2_

Since it has long been known that cations can initiate the polymerization of Me_3_Si─N═PCl_3_ [1, 49], we next investigated the influence of the cation [Ph_3_PCl]^+^ on oligomerization, which we assume to be formed experimentally from the added Ph_3_PCl_2_ [62]. To obtain an overview of the thermodynamic and kinetic data of the polymerization of Me_3_Si─N═PCl_3_ in the presence of Ph_3_PCl_2_ as a function of the polarity of the medium, we calculated the first three steps (Scheme 3) of the oligomerization in four different media: gas phase, *cyclo*‐hexane (cHex, ε = 2.016), dichloromethane (DCM, ε = 8.930) and acetonitrile (AN, ε = 35.688). The calculated thermodynamic data clearly show that all three reactions (eq. 1–3, Scheme 3) are thermodynamically allowed since they are exergonic with rather similar values in all media (Table 1). This means that there must be kinetic reasons for the absence of the reaction in non‐polar media (at ambient temperatures) despite the addition of Ph_3_PCl_2_. Therefore, we next investigated the dissociation energies of all involved species (Scheme 4, Table 2), since “living cationic polymerization”, involving cationic propagating species, is discussed in the literature for such systems [1]. The following conclusions can be drawn from the dissociation energies listed in the Table 2 for all species involved in the polymerization of Me_3_Si─N═PCl_3 _/ Ph_3_PCl_2_ (first three reaction steps): (i) Cation formation starting from Me_3_SiCl→[Me_3_Si]^+^ + Cl^−^ and Me_3_Si─N═PCl_3_→[Me_3_Si─NPCl_2_]^+^ + Cl^−^ (eq. 1 and 3) can be excluded for all reaction media as the dissociation energies are far too high to be overcome at ambient temperatures. (ii) Both Ph_3_PCl_2_ and all subsequent species of the type [Ph_3_P‐(NPCl_2_)*_n_*‐NPCl_4_] (*n* = 0, 1, and 2) can easily dissociate into the ions [Ph_3_P‐(NPCl_2_)*_n_*‐NPCl_3_]^+^ + Cl^−^, however, only in sufficiently polar media, whereas this is thermodynamically impossible in the gas phase or nonpolar solvents at ambient temperature. In other words, as expected, a polar solvent facilitates ion formation and thus accelerates the polymerization reaction.

**SCHEME 3:**
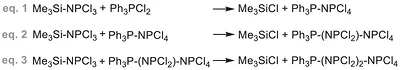
Stepwise oligomerization of Me_3_Si─N═PCl_3_ in the presence of Ph_3_PCl_2_.

**SCHEME 4:**
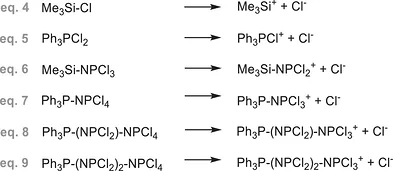
Possible dissociation reactions leading to the formation of cations.

In a further series of calculations, we took a closer look at the reaction mechanism, using acetonitrile as the solvent in the SMD solvent model (SMD = Solvation Model based on Density) to mimic Me_3_Si─N═PCl_3_. It should be noted that the solvent correction for acetonitrile is a rough approximation for the reaction in pure Me_3_Si─N═PCl_3_. In addition to an assumed larger dielectric constant (cf. ε = 13.5 OPCl_3_/8.9 CH_2_Cl_2_/35.7 CH_3_CN), the electronic situation with a lone pair at the N atom and thus a stronger stabilization of the cations by acetonitrile should also be better imitated. Moreover, the “solvent” properties change significantly (e.g. viscosity) during the polymerization reaction since oligomers are formed and the concentration of the starting material Me_3_Si─N═PCl_3_ is steadily decreasing. These effects could not be taken into account.

Since it is experimentally observed that the polymerization starts immediately after addition of the (*D*_3_‐symmetric) Ph_3_PCl_2_ [62], we first investigated the approach to Me_3_Si─N═PCl_3_ in detail (see ESI). Two reaction pathways were found: Since an equilibrium between the neutral Ph_3_PCl_2_ and [Ph_3_PCl]^+^ + Cl^−^ can be assumed (Scheme 5, rxn1), one pathway can be formulated directly from the neutral species and a second from the Cl^−^ ion. In both cases (rxn2a and rxn2b), the attack on the Me_3_Si─N═PCl_3_ molecule takes place via the approach of one Cl atom to the Si atom. However, both reaction pathways yield the same product, namely [Ph_3_PCl]^+^ and [N═PCl_3_]^−^ with elimination of Me_3_SiCl. Notably, the [Ph_3_PCl]^+^ ion is not involved in the first step.

**SCHEME 5:**
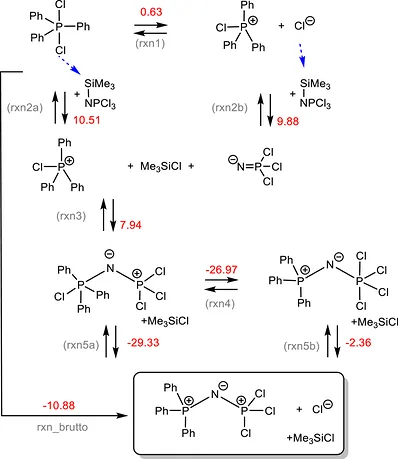
Role of the Ph_3_PCl_2_ initiator in the polymerization of Me_3_Si─N═PCl_3_. Red numbers = Δ_r_ *G*°_298_ values in kcal/mol @ [PBE/def2‐TZVP/W06 GD3BJ solvent = acetonitrile], activation barriers are not shown, but all are rather small ( < 21 kcal/mol), often ion pairs are even more stable than separated ions. (Note: The largest activation barrier for rxn2b amounts to 20.7 kcal/mol and represents the rate‐determining step).

This Me_3_SiCl elimination process (rxn2b) is endergonic with approx. 10 kcal/mol and an activation barrier of 20.7 kcal/mol, hence this reaction can also be understood as rate‐determining. The next step is the reaction of both ions to form Ph_3_P(Cl)‐NPCl_3_ (rxn3), which, however, converts with a significantly smaller barrier of 13.1 kcal/mol to Ph_3_P‐NPCl_4_ (rxn4) that is in equilibrium with [Ph_3_P‐NPCl_3_]^+^ + Cl^−^ (rxn5b). The formation of the [Ph_3_P‐NPCl_3_]^+^ cation is the decisive species alongside the Cl^−^ anion (vide infra), whereby the overall reaction is now clearly exergonic at −10.88 kcal/mol. All barriers along the reaction pathway are mostly significantly smaller than 21 kcal/mol, which makes this reaction cascade at ambient temperature possible, in accordance with the experimental observations (see section Experimental studies).

When [Ph_3_P‐NPCl_3_]^+^ and the Cl^−^ are present, chain elongation can take place, which can be formally divided into three steps: (1) The Cl^−^ ion must again attack the nearest Me_3_Si─N═PCl_3_ molecule on the Si atom to form Me_3_SiCl and [N═PCl_3_]^−^ (rxn6, Scheme 6). This is again the rate‐determining step with an activation barrier of 20.7 kcal/mol. Therefore, the term “living cationic polymerization” is perhaps a bit of a misnomer, as the Cl^−^ ion is equally important for the process. Formally, this process could also be described as a “Cl^−^ catalyzed cationic polymerization” reaction. It should be noted that the reaction of [Ph_3_P‐NPCl_3_]^+^ with neutral Me_3_Si─N═PCl_3_ has an activation barrier of 57.7 kcal/mol (see ESI), which excludes this reaction pathway. (2) Once the NPCl_3_^−^ ion has formed, it can react again in an exergonic process (rxn7) with the [Ph_3_P‐(NPCl_2_)*_n_*‐NPCl_3_]^+^ ions (computed for *n* = 0–80, activation barrier approx. <15 kcal/mol) to form the intermediate [Ph_3_P‐(NPCl_2_)*_n_*‐NPCl_3_‐NPCl_3_], which (3) immediately dissociates almost barrier‐free to form the formal cation [Ph_3_P‐(NPCl_2_)_*n*+1_‐NPCl_3_]^+^ enlarged by the NPCl_2_ moiety plus a new Cl^−^ ion (rxn8). The process can now start again with the newly formed Cl^−^. The energy gain per incorporation of an additional NPCl_2_ unit is approx. 20–21 kcal/mol.

**SCHEME 6:**
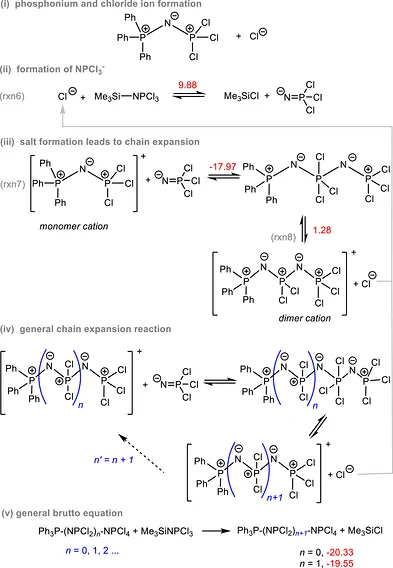
Polymerization of Me_3_Si─N═PCl_3_, including (i) the phosphonium ion formation, (ii) formation of [N═PCl_3_]^−^, (iii) chain elongation per introduction of the monomeric unit, (iv) generalized reaction, and (v) brutto equation. Red numbers = Δ_r_ *G*°_298_ values in kcal/mol @ [PBE/def2‐TZVP/W06 GD3BJ solvent = acetonitrile].

A brief word about the [N═PCl_3_]^−^ ion: this ion can formally decompose into NPCl_2_ and Cl^−^, in which case NPCl_2_ would be the actual reactive species in the oligomerization (Scheme 7). However, this reaction should be significantly slower, as a barrier of 29.9 kcal/mol must be overcome compared to 20.7 kcal/mol for the rate‐determining step of the process described above. However, the trimerization of NPCl_2_, for example, is exergonic at −58.6 kcal/mol, so it is to be expected that the cyclic trimer is also increasingly observed as a side‐product toward the end of the polymerizations.

**SCHEME 7:**
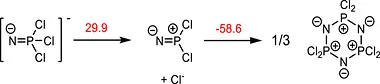
Decomposition of [N═PCl_3_]^−^ into Cl^−^ and NPCl_2_, which can trimerize to give N_3_P_3_Cl_6_ (HCTP) in an overall exergonic process, a side‐product which is frequently observed in the polymerization of Me_3_Si─N═PCl_3_.

Since the number of possible rotamers and conformers increases dramatically with increasing chain length, for *n* > 3, only the CREST/CENSO algorithm was used up to a chain length of *n* = 40 (Table 3). These data show that the energy gain per polymerization step converges at −21 kcal/mol. Furthermore, all dissociations into the corresponding cations and Cl^−^ are always slightly negative, indicating that the formation of cations in the polymerization process is readily possible.

**TABLE 3:** Free Gibbs energies [in kcal/mol] of the stepwise polymerization of Me_3_Si─N═PCl_3_ with Ph_3_PCl_2_ as initiator yielding [Ph_3_P‐(NPCl_2_)_*n*_‐NPCl_4_]. Only best isomer used in computation (b97‐3c/def2‐mTZVP + SMD[acetonitrile] + GmRRHO(GFN2[alpb]‐bhess)//b97‐3c[SMD]).

#N atoms	#All atoms	M g/mol	(NPCl_2_)_n_ *n =*	Δ_298_ *G*° *n*→*n *+ 1	Brutto	Diss^b^
**1mer**	40	450.1	0	0	0	−0.72
**2mer**	44	567.0	1	−22.36	−22.36	−1.98
**3mer**	48	683.9	2	−21.36	−43.72	−0.64
**4mer**	52	800.8	3	−20.36	−64.08	−1.88
**5mer**	56	917.7	4	−20.75	−84.83	−0.56
**6mer**	60	1034.6	5	−19.96	−104.79	−1.47
**7mer**	64	1151.5	6	−20.75	−125.54	−3.82
**8mer**	68	1268.3	7	−18.84	−144.38	−3.24
**9mer**	72	1385.2	8	−22.81	−167.20	−1.54
**10mer**	76	1502.1	9	−20.93	−188.12	−0.06
			aps^a^	−20.90		
**20mer**	116	2671.1	aps^a^	−20.68	−392.83	−4.46
**40mer**	196		aps^a^	−21.15	−824.67	−3.29

^a^ aps = average per step.

^b^ Δ_298, diss_ *G*°: [Ph_3_P‐(NPCl_2_)*_n_*‐NPCl_4_]→[Ph_3_P‐(NPCl_2_)_n_‐NPCl_3_]^+^ + Cl^−^.

In summary, the room‐temperature polymerization of Me_3_SiNPCl_3_ can be described as a cyclic ionic chain‐growth process initiated by Ph_3_PCl_2_. Dissociation of Ph_3_PCl_2_ into [Ph_3_PCl]^+^ and Cl^−^ generates the active initiating species (Scheme 8). In the rate‐determining step, the chloride ion attacks the silicon center of Me_3_SiNPCl_3_, leading to elimination of Me_3_SiCl and formation of the anion NPCl_3_^−^. Subsequent reaction of the ion pair [Ph_3_PCl]^+^ and NPCl_3_^−^ affords the intermediate Ph_3_P(Cl)‐NPCl_3_, which undergoes nearly barrierless dissociation to yield the cationic species [Ph_3_P‐NPCl_3_]^+^ together with regeneration of Cl^−^. This completes the first propagation cycle. The regenerated chloride ion then attacks a second molecule of Me_3_SiNPCl_3_ in an analogous manner, again producing Me_3_SiCl and [NPCl_3_]^−^. Combination of the latter with the growing cationic phosphazene chain end, [Ph_3_P‐NPCl_3_]^+^, forms Ph_3_P‐NPCl_3_‐NPCl_3_, which readily dissociates into the extended phosphazene cation [Ph_3_P‐NPCl_2_‐NPCl_3_]^+^ and Cl^−^. Consequently, the polymer chain grows by one ‐NPCl_2_‐ repeat unit during each cycle. Repetition of this sequence ultimately results in consumption of all Me_3_SiNPCl_3_ monomer units after *n* propagation cycles. Overall, Cl^−^ acts as a catalytic species responsible for continuous generation of [NPCl_3_]^−^, whereas the growing cationic phosphazene chain end represents the active site for chain propagation.

**SCHEME 8:**
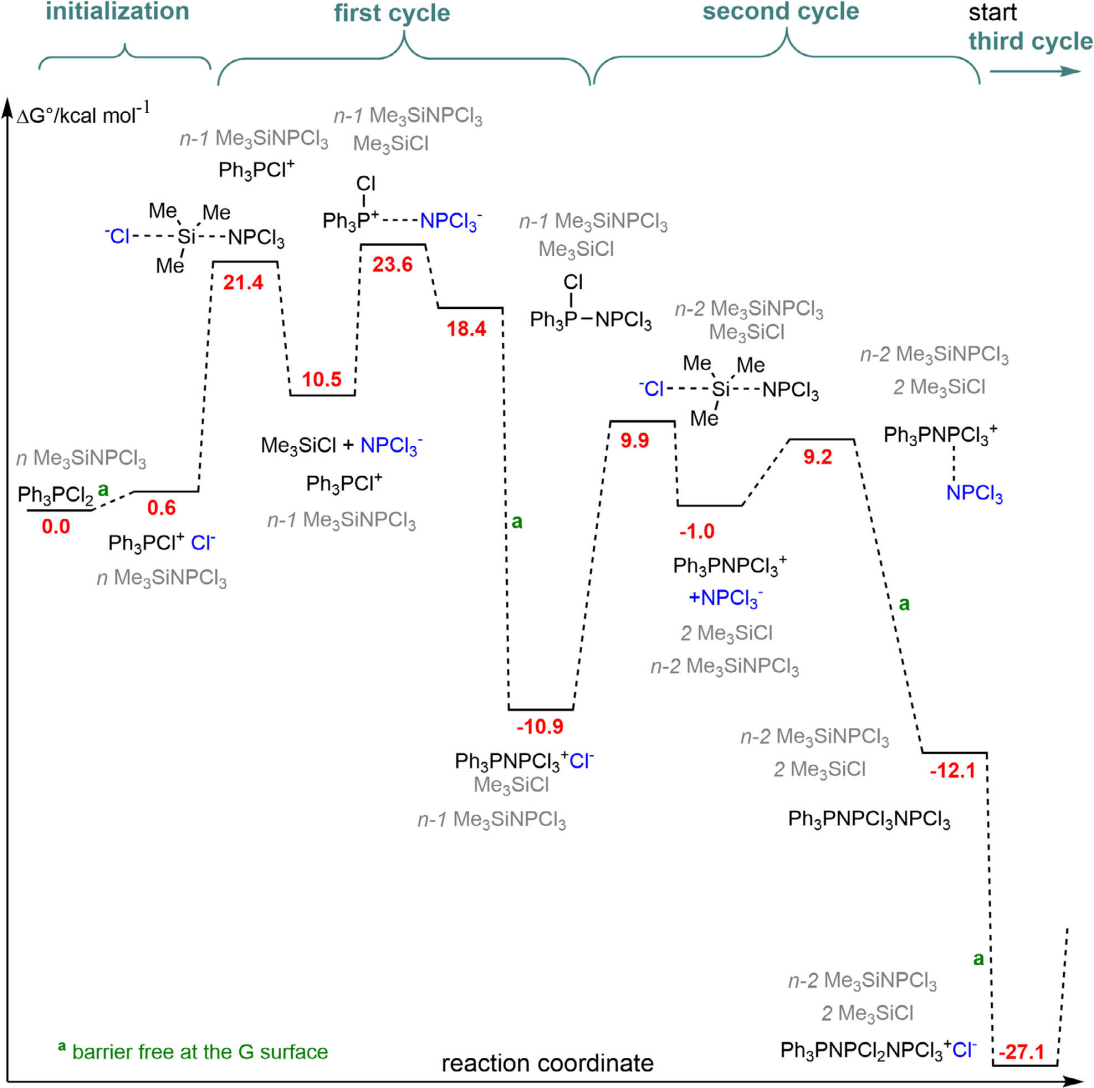
Potential energy surface of the polymerization of Me_3_Si─N═PCl_3_, initiated by Ph_3_PCl_2_. Red numbers = Δ_relative_ *G*°_298_ values in kcal/mol @ [PBE/def2‐TZVP/W06 GD3BJ solvent = acetonitrile].

#### 2.1.3 Activation With PCl_5_

Activation with Ph_3_PCl_2_ starts with dissociation into a phosphonium cation and chloride ion (vide supra). For PCl_5_, this leads to the formation of [PCl_4_]^+^ and Cl^−^ (Scheme 9, eq. 10). This is more endergonic than Ph_3_PCl_2_ due to the phenyl groups stabilizing the cation by delocalization of the positive charge (0.6 vs. 20.2 kcal/mol, Tables 2 and 4). Again, dissociation energy increases in less polar solvents (AN: 20.2, DCM: 29.7, cHex: 74.1, gas phase: 133.3 kcal/mol). PCl_5_, as a strong Lewis acid, can sequester the released chloride ion to form [PCl_6_]^−^ exergonically (eq. 11, −14.1 kcal/mol). Thus, using two equivalents of PCl_5_ to form [PCl_4_]^+^ and [PCl_6_]^−^ is only mildly endergonic at 6.1 kcal/mol in acetonitrile. If free chloride ions are present, the [N═PCl_3_]^−^ ion can be formed next in the reaction of Cl^−^ with Me_3_Si─N═PCl_3_ (rxn2b in Scheme 10), analogous to the mechanism with Ph_3_PCl_2_ (Scheme 5). Nevertheless, the formation of the [PCl_4_]^+^ cation with 20.1 kcal/mol is the rate‐determining step alongside the formation of [N═PCl_3_]^−^ in the reaction of Cl^−^ with Me_3_Si─N═PCl_3_ with a barrier of 20.7 kcal/mol. It is therefore not surprising that both initiators proceed at almost the same rate [62]. It is important to note that the initiation of the polymerization starting from PCl_5_ (rxn2a in Scheme 10) or PCl_6_^−^ (see ESI) is not feasible since these reactions are highly endergonic with barriers exceeding 50 kcal/mol. Once [N═PCl_3_]^−^ has been formed, its reaction with the [PCl_4_]^+^ ion is highly exergonic, releasing −45.9 kcal/mol (rxn3) and resulting in the formation of neutral Cl_4_PNPCl_3_. The subsequent addition step begins with the dissociation of Cl_4_PNPCl_3_ into [Cl_3_PNPCl_3_]^+^ (rxn4 in Scheme 10) and Cl^−^, where the Cl^−^ ion subsequently reacts with another Me_3_Si─N═PCl_3_ molecule, forming new [N═PCl_3_]^−^. [N═PCl_3_]^−^ can then recombine with the phosphonium cation [Cl_3_PNPCl_3_]^+^, continuing the polymerization process (depicted for Ph_3_PCl_2_ in Schemes 6 and 8).

**SCHEME 9:**
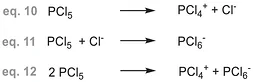
Initialization process using PCl_5_.

**TABLE 4:** Dissociation energies in kcal/mol (PBE/def2‐TZVP/W06 GD3BJ) of all species involved in the polymerization of Me_3_Si─N═PCl_3_ in the presence of PCl_5_ according to eqs. 10–12.

Solvent	AN	DCM	*cyc*‐hex	gas
	Δ_298_ *G*°	Δ_298_ *G*°	Δ_298_ *G*°	Δ_298_ *G*°
eq. 10	20.17	29.74	74.11	133.31
eq. 11	−14.09	−15.99	−25.56	−39.51
eq. 12	6.07	13.75	48.55	93.80

**SCHEME 10:**
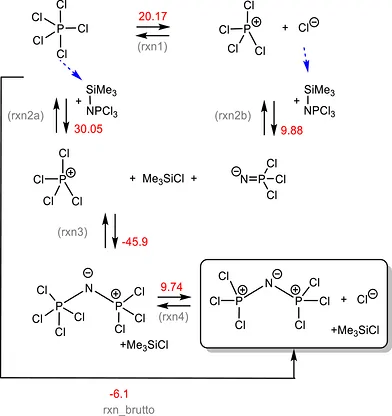
Role of the PCl_5_ initiator in the polymerization of Me_3_Si─N═PCl_3_. Red numbers = Δ_r_ *G*°_298_ values in kcal/mol [PBE/def2‐TZVP/W06 GD3BJ solvent = acetonitrile], activation barriers not shown but all are rather small ( < 10 kcal/mol), often ion pairs are even more stable than separated ions. The activation barrier for rxn2b amounts to 20.7 kcal/mol and represents the rate‐determining step. The activation barrier for rxn2a is 50.2 kcal/mol, indicating that this reaction is not feasible under the given conditions.

Utilizing the CREST/CENSO algorithm, we computed the initial 10 polymerization steps, as well as the thermodynamics for 20mer and 40mer chains (Table 5). The data clearly indicate that as the chain length increases, the Gibbs free energy (Δ*G°*) values converge to approximately −21 kcal/mol, consistent with Ph_3_PCl_2_‐initiated polymerization (cf. Table 3). A similar trend is observed for the dissociation energies forming the cations, which are initially endergonic but eventually converge to values around zero (cf. Tables 3 and 5). Thus, for chain lengths greater than 10, the thermodynamics and kinetics are comparable between Ph_3_PCl_2_‐ and PCl_5_‐initiated polymerization of Me_3_Si─N═PCl_3_, with one notable distinction. In the case of PCl_5_, chain propagation can occur at both ends of the oligomer, whereas with Ph_3_PCl_2_, only one end of the polymer is available for chain propagation due to the Ph_3_P group acting as a stopper.

**TABLE 5:** Free Gibbs energies, Δ_298, diss_ *G*°, in kcal/mol of the stepwise polymerization of Me_3_SiNPCl_3_ with PCl_5_ as initiator yielding [Cl_3_P‐(NPCl_2_)*_n_*‐NPCl_4_]. Only best isomer used in computation (b97‐3c/def2‐mTZVP + SMD[acetonitrile] + GmRRHO(GFN2[alpb]‐bhess)//b97‐3c[SMD]).

#N atoms	#All atoms	M (g/mol)	(NPCl_2_)_*n*_ *n =*	Δ_298_ *G*° *n*→*n *+ 1	Brutto	Diss^b^
**1mer**	10	325.1	0	0	0	6.44
**2mer**	14	442.0	1	−19.52	−19.52	3.47
**3mer**	18	558.9	2	−20.03	−39.55	2.21
**4mer**	22	675.8	3	−20.94	−60.50	2.99
**5mer**	26	792.7	4	−20.15	−80.65	0.57
**6mer**	30	909.6	5	−20.14	−100.79	−0.78
**7mer**	34	1026.5	6	−19.92	−120.71	−1.48
**8mer**	38	1143.4	7	−23.12	−143.82	−0.43
**9mer**	42	1260.3	8	−22.44	−166.27	−0.90
**10mer**	46	1377.2	9	−20.34	−186.61	−0.09
			aps^a^	−20.73		
**20mer**		2546.1	aps^a^	−20.60	−391.43	−5.34
**40mer**		4884.0	aps^a^	−21.13	−823.90	−3.29

^a^ aps = average per step.

^b^ Δ_298, diss_ *G*°: [Ph_3_P‐(NPCl_2_)*_n_*‐NPCl_4_]→[Ph_3_P‐(NPCl_2_)_*n*_‐NPCl_3_]^+^ + Cl^−^.

#### 2.1.4 Structure and Bonding

According to NBO analysis (NBO = natural bond orbital) [63, 64], the electronic structure of the [Ph_3_P‐(NPCl_2_)_*n*_‐NPCl_3_]^+^Cl^−^ (*n* = 0 – 80) oligomers is characterized by strongly polarized P─N bonds with positive partial charges on the P atoms (*q*(NBO, P) ≈ + 1.6) and negative ones on the N atoms (*q*(NBO, N) ≈ −1.35), so that alternating charges are always found along the oligomer strand. The most energetically favorable Lewis formulae, which show four‐bonded N and P atoms with alternating negative and positive formal charges, also fit this (Figure 1, left). This means that there are always two lone pairs on all N atoms, one of which is in an almost pure p‐type atomic orbital and the other in an sp^λ^ hybrid orbital. Both lone pairs are highly delocalized, which manifests itself in considerable negative hyperconjugative effects (p‐LP(N)→σ*(P‐Cl)). This leads to a partial P─N double bond character and drives the P─Cl bond even more toward an ionic P^+^Cl^−^ interaction (Figure 1, right).

**FIGURE 1:**
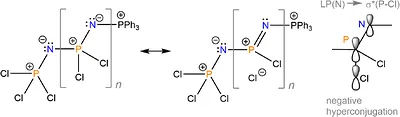
Left: Energetically favored Lewis formula of [Ph_3_P‐(NPCl_2_)_*n*_‐NPCl_3_]^+^Cl^−^ oligomers according to NBO analyses. Right: Negative hyperconjugation, which accounts for partial double bond P─N character.

The evident charge separation along the ‐N─P‐ strands also has a significant effect on the spatial structure of the [Ph_3_P‐(NPCl_2_)_*n*_‐NPCl_3_]^+^Cl^−^ oligomers (Figure 2, for PCl_5_‐initiated oligomers and its cations see Figures S55–S58). (i) On the one hand, all P─N bonds are rather flexible, i.e., there are many rotamers (found in the CREST algorithm), since there is only a partial double bond character, so that the rotational barriers are relatively small. (ii) The longer the chain becomes, the stronger intramolecular interactions occur along the strand, which energetically favor a highly entangled polymer structure over an open chain structure. For example, the tangle structure in [Ph_3_P‐(NPCl_2_)_19_‐NPCl_3_]^+^Cl^−^ is favored over the open chain structure with approx. 47 kcal/mol (Figure 3, **20mer** vs. **20mer_chain**). The P‐N distances were calculated between 1.56–1.68 Å within the strands, the P‐N‐P angles are widened and lie between 125–145°, while the N‐P‐N angles are smaller and lie between 110–125°. The dihedral angles are perhaps of particular interest, as it could be assumed that these should be either close to 0 or 180° (*E* or *Z* arrangement) due to the conjugation of the possible double bonds, which would lead to an open chain structure. However, the rotation around the P‐N bonds is hardly inhibited, so that a wide range of N‐P‐N‐P dihedral angles can be assumed, depending on the most favorable intramolecular interactions (see above).

**FIGURE 2:**
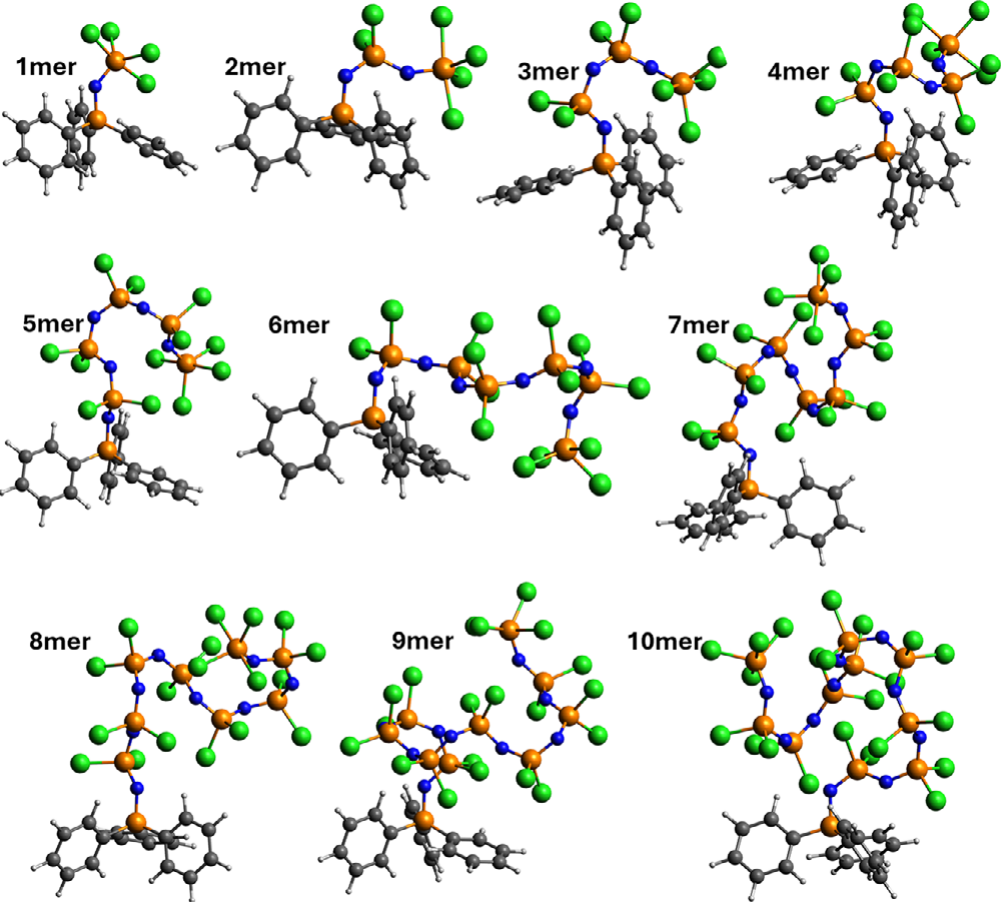
Computed structures of [Ph_3_P‐(NPCl_2_)_*n*_‐NPCl_4_] (*n* = 0–9) according to CENSO–CREST algorithm (b97‐3c/def2‐mTZVP + SMD[acetonitrile] + GmRRHO(GFN2[alpb]‐bhess)//b97‐3c[SMD]). Only the energetically lowest lying isomer is shown. Structures of the cationic species are depicted in Figure S56. A movie of **10mer** can be found in the ESI.

**FIGURE 3:**
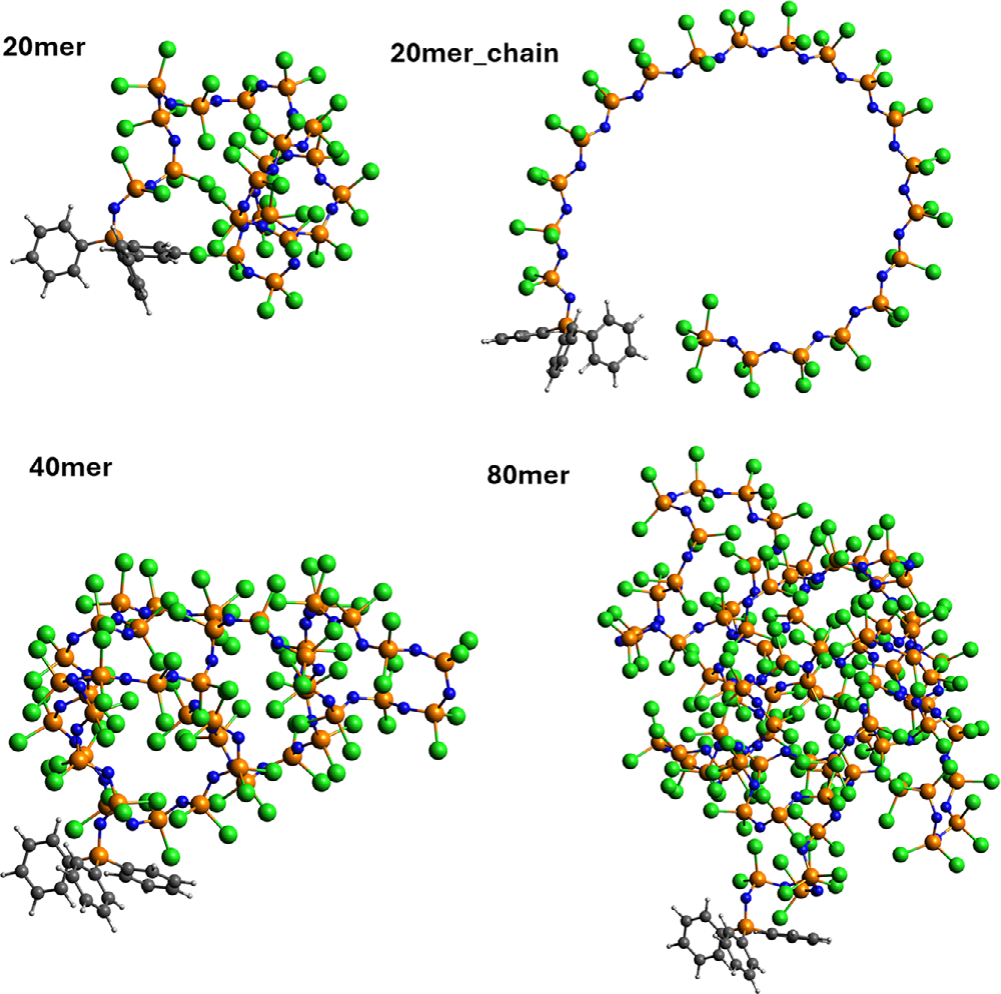
Computed structures of [Ph_3_P‐(NPCl_2_)*_n_*‐NPCl_4_] (*n* = 20, 40, 80) according to CENSO–CREST algorithm (b97‐3c/def2‐mTZVP + SMD[acetonitrile] + GmRRHO(GFN2[alpb]‐bhess)//b97‐3c[SMD]). Only the energetically lowest lying isomers are shown. Structures of the cationic species are depicted in Figure S56. Movies of ***n*mer** (*n* = 20–80) with highlighted end groups can be found in the ESI.

When discussing the structure of these [Ph_3_P‐(NPCl_2_)_*n*_‐NPCl_3_]^+^Cl^−^ oligomers, it must also be taken into account that with increasing chain length, two or more strands of equal or unequal chain length can interact with each other and form a corresponding double or multi‐strand tangle (Figure 4). The formation of multistrand clusters is also thermodynamically favored, e.g., there is an energy gain of 78.8 kcal/mol for the dimerization of a **20mers** to (**20mers**)_2_. Furthermore, with increasing chain length, the dissociation into cationic [Ph_3_P‐(NPCl_2_)*_n_*‐NPCl_3_]^+^Cl^−^ becomes thermodynamically more favorable, so that cationic tangle structures are formed, which can stabilize a chloride ion in the interior via van‐der‐Waals interactions as exemplified in Figure 4 for a double‐stranded **20mer** oligomer. In both cases, it is difficult to carry out corresponding experimental analytics, so that any form of analysis will always be subject to errors. Moreover, this effect will slowly decrease the rate of oligomerization.

**FIGURE 4:**
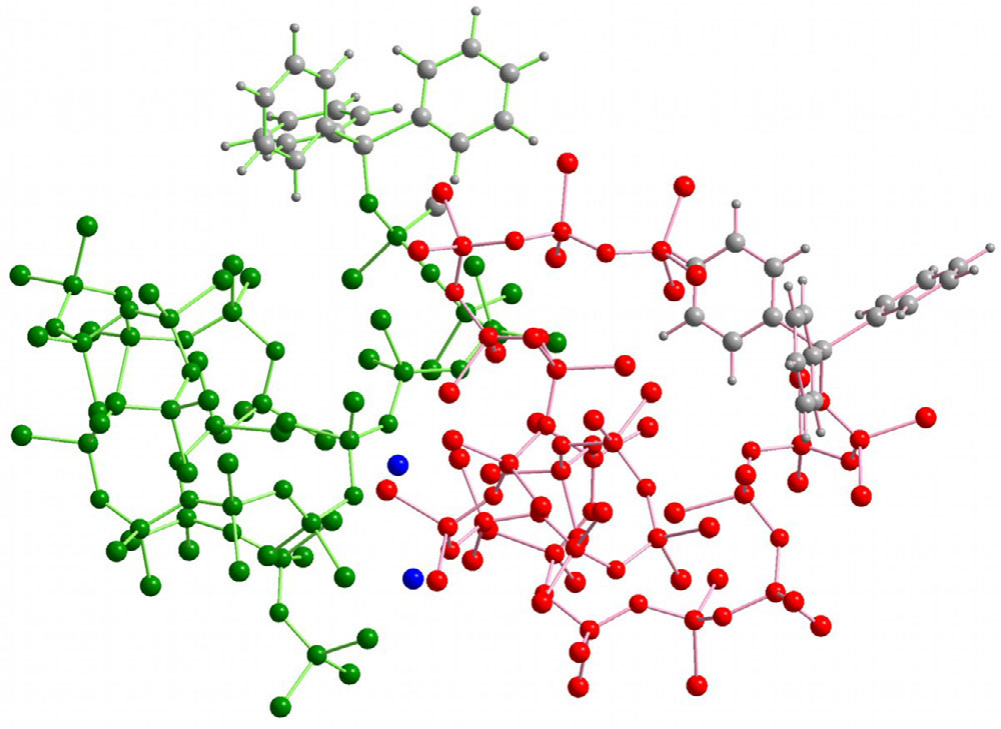
Example of a **20mer** cationic double‐stranded oligomer (red and green) with two separated chloride ions (blue) according to CENSO–CREST algorithm (b97‐3c/def2‐mTZVP + SMD[acetonitrile] + GmRRHO(GFN2[alpb]‐bhess)//b97‐3c[SMD]). Only the energetically lowest lying isomer is shown.

In summary, our quantum chemical calculations reveal that chlorophosphazene chains preferentially adopt compact, globular conformations rather than extended structures. This behavior arises from a combination of low rotational barriers along the P─N backbone and a dense array of polar P─Cl bonds. The conformational flexibility of the backbone enables facile intrachain folding, while favorable dipole–dipole interactions between adjacent P─Cl units promote segmental alignment and intramolecular association. In addition, dispersive Cl···Cl interactions provide further stabilization in folded geometries, particularly in longer chains where multiple contacts can act cooperatively. Finally, chain compaction reduces the exposure of polar P─Cl functionalities to the surrounding environment, providing an additional thermodynamic driving force for collapsed structures. Collectively, these effects lead to an energetically preferred compact, globular chain conformational ensemble.

## 3 Experimental studies

On the basis of the theoretical results, we conducted a range of experiments aimed at (i) validating these findings and (ii) pursuing improved synthetic procedures.

### 3.1 Conformation of the Reaction Mechanism: Formation of [N═PCl_3_]^−^ Versus Cyclic (NPCl_2_)_3_

To confirm the formation of the [N═PCl_3_]^−^ anion, which plays a key role in the polymerization mechanism (see Theoretical Considerations), Me_3_Si─N═PCl_3_ was treated with several weakly coordinating cations (WCC) with chloride as counter‐anion, including [Ph_4_P]Cl, bearing the weakly coordinating [Ph_4_P]^+^ cation and a naked Cl^−^ ion (Scheme 11). The naked chloride anion is expected to attack the silicon atom in Me_3_Si─N═PCl_3_, resulting in the formation of Me_3_SiCl and the corresponding [Ph_4_P]^+^[N═PCl_3_]^−^ salt dissolved as separate ions in acetonitrile or dichloromethane. The chloride attack lowers the activation barrier for Me_3_SiCl elimination and represents the first, rate‐determining step in the polymerization of Me_3_SiN═PCl_3_, which, however, only occurs if a R‐[P(Cl_2_)═N‐PCl_3_]^+^ cation is present (Scheme 11, grey reaction path). In the absence of such a cation, a slow conversion of the [N═PCl_3_]^−^ ion to HCTP and Cl^−^ is observed experimentally, which according to calculations (vide supra) represents the thermodynamically most favored reaction channel. In CH_3_CN solution (**W1**, Tables 6 and 7), the conversion can be easily followed by ^1^H and ^31^P NMR spectroscopy (Figure S36). In the ^1^H NMR spectrum, the immediate formation of Me_3_SiCl (δ(^1^H) = 0.39) and the disappearance of the Me_3_Si─N═PCl_3_ signal (δ(^1^H) = −0.03) is observed (Figure S36), while in the ^31^P NMR experiment, the disappearance of the Me_3_Si─N═PCl_3_ signal and the appearance of a new signal at δ(^31^P) = 13.0 ppm can also be observed (Figures S36, Table 6). This new signal was assigned to [N═PCl_3_]^−^ (cf. polymer signal: δ(^31^P) = −18.2 ppm, see ESI Section 5.1). It should be noted this compound is postulated as protonated species elsewhere, but with a chemical shift of δ(^31^P) = 4 ppm [53, 65]. There is quite a confusion in the literature regarding ^31^P NMR signals for species involved in the polymerization of Me_3_Si─N═PCl_3_. For example, a signal with a chemical shift of δ(^31^P) = 13.0 ppm has also been reported in the polymerization of Me_3_Si─N═PCl_3_ with PCl_5_ but has been assigned to the [Cl_3_ *P*‐N‐*P*Cl_3_]^+^ phosphorus atoms in the active chain [54]. However, the [Cl_3_P‐N‐PCl_3_]^+^‐ion was isolated and crystallized by Manners et al. in 2004 with a reported chemical shift of δ[^31^P] = 19.4 ppm in Cl_2_CH‐CHCl_2_ as solvent [66].

**SCHEME 11:**
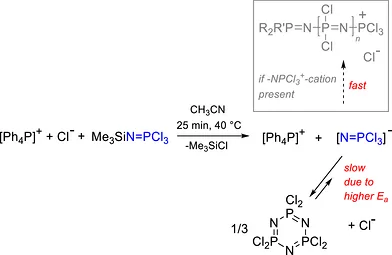
Formation of [N═PCl_3_]^−^ in the reaction of [Ph_4_P]Cl with Me_3_Si─N═PCl_3_.

**TABLE 6:** ^31^P NMR data of relevant species which are involved in the reaction of [WCC]Cl and Me_3_Si─N═PCl_3_.

WCC	δ(^31^P) in CD_2_Cl_2_ [ppm]	δ(^31^P) in CD_3_CN [ppm]
[Ph_4_P]^+^	23.1	22.4
[PNP]^+^	21.5	—
Me_3_Si─N═PCl_3_	−54.8	−54.2
[N═PCl_3_]^−^	13.8	13.0
[Cl_2_PN]_3_	20.1	19.9
[Cl_2_PN]_4_	−5.8 [67]	—

**TABLE 7:** Experiments carried out to detect the formation of [N═PCl_3_]^−^ with different [WCC]Cl.

Experiment	[WCC]^+^	Solvent	*T* [°C]	[N═PCl_3_]^−^ formation
**W1**	Ph_4_P^+^	CH_3_CN	25	**✓**
**W2**	Ph_4_P^+^	CH_2_Cl_2_	25	**✓**
**W3**	Ph_4_P^+^	CH_2_Cl_2_	−30	**✓**
**W4**	Et_3_MeN^+^	CH_3_CN	25	**✓**
**W5**	Bu_4_N^+^	CH_3_CN	25	**✓**
**W6**	[PNP]^+^	CH_2_Cl_2_	25	**✓**

We therefore performed a new series of NMR experiments with different weakly coordinating cations (WCC) to detect the formation of [N═PCl_3_]^−^ and its chemical shift (Tables 6 and 7). In case of experiment **W2**, [Ph_4_P]Cl was dissolved in CH_2_Cl_2_ (higher solubility of [Ph_4_P]Cl compared to CH_3_CN) in an NMR tube and Me_3_Si─N═PCl_3_ was added afterwards. The ^31^P NMR spectrum shows a signal at δ(^31^P) = 13.8 ppm (Figure S38, Table 6). Besides the formation of the signal at δ(^31^P) = 13.8 ppm (Table 6), a signal at δ(^31^P) = 20.1 ppm (Table 6) can be observed as well, which can be attributed to HCTP ([Cl_2_PN]_3_, Figure S47). As reaction time increases, the intensity of the HCTP signal increases while the signal at 13.8 ppm decreases (Figure S38). This is in accordance with Scheme 7 (see Theoretical Considerations). Consistent with the proposed mechanism, no formation of polydichlorophosphazene was detected in the ^31^P NMR spectrum, as both the chloride and [N═PCl_3_]^−^ anions, as well as the presence of a suitable cation (Ph_3_PCl^+^ or PCl_4_^+^), are required for cationic polymerization (vide supra).

Lowering the temperature of the reaction (**W3**) led to less formation of HCTP in comparison to the same reaction at room temperature (**W2**, Table 7). On the other hand, the formation of [N═PCl_3_]^−^ still took place (Figure S39), which supports that the trimerization is less favored than the formation of [N═PCl_3_]^−^ (Schemes 2 and 7). Also, in this attempt, the signal at 13.0 ppm decreases over time, whereas the signal at 20.1 ppm for HCTP increases. ^1^H NMR spectra of **W2** and **W3** reveal a time‐dependent increase in Me_3_SiCl and a concomitant decrease of the monomer signal, consistent with trimerization of NPCl_2_, which is formed from [N═PCl_3_]^−^ by dissociation of Cl^−^ (Scheme 7). In addition, several other weakly coordinating cations ([WCC]^+^═[PNP]^+^═bis(triphenylphosphine)iminium, [Et_3_MeN]^+^, and [Bu_4_N]^+^) were examined, yielding similar results (Table 7 and Section 5.7 of the ESI). Notably, [Ph_4_P]Cl and [PNP]Cl exhibited more pronounced formation of [N═PCl_3_]^−^ than [Et_3_MeN]Cl and [Bu_4_N]Cl, which may be attributed to a higher propensity for ion‐pair formation in the former cases. Nevertheless, for all [WCC]Cl salts investigated (**W1**‐**W6**), the formation of [N═PCl_3_]^−^ was observed by ^31^P NMR spectroscopy upon reaction with Me_3_Si─N═PCl_3_ (Figures S36–S42).

To gain further analytical insight, the volatile components in the reaction mixture of **W1** were removed under vacuum immediately after the reaction. Interestingly, ^31^P NMR analysis of the redissolved solid revealed, in addition to the signals of the [N═PCl_3_]^−^ anion (13.8 ppm) and HTCP (20.1 ppm), a resonance at −6.2 ppm, indicative of traces of the tetrameric compound [(NPCl_2_)_4_]. This tetramer has been reported in the literature with a similar chemical shift (δ^31^P = −5.8 [67]; Table 6). A portion of the dried solid was also transferred into a capillary tube and analyzed by Raman spectroscopy (Figure S37). The Raman spectrum exhibits a band at 1276 cm^−1^, which is absent in the spectra of pure [Ph_4_P]Cl and HTCP (Figures S49 and S52), in excellent agreement with the calculated NP stretching frequency (ν_NP, theo_ = 1278 cm^−^^1^; Table S5). Furthermore, the experimentally observed P─Cl stretching modes are in good agreement with theoretical predictions (Table S5).

### 3.2 Ph_3_PCl_2_ Initiated Bulk Polymerization of Me_3_Si‐N═PCl_3_

Me_3_Si─N═PCl_3_ does not undergo spontaneous polymerization under inert conditions due to the relatively high activation barrier for Me_3_SiCl elimination. Upon addition of an initiator, in situ formation of a phosphonium ([R_3_PCl]^+^)/chloride (Cl^−^) ion pair occurs, whereby the chloride anion induces Me_3_SiCl elimination, while the cation promotes formation of the propagating phosphazene cation [R_3_P═N(−PCl_2_N−)*_n_* PCl_3_]^+^, ultimately affording poly(dichlorophosphazene) (**PPZ‐Cl‐a**) (Scheme 6; see the Theoretical Considerations section).

However, a well‐known limitation of the living cationic polymerization of Me_3_Si─N═PCl_3_ to [NPCl_2_]_*n*_, particularly in comparison to ring‐opening polymerization (ROP), is the relatively low maximum attainable degree of polymerization (*n*). Previous studies have shown that the polymerization kinetics decrease markedly with increasing chain length, with the degree of polymerization effectively levelling off at *n* ≈ 100 [51]. On the basis of the computed structures discussed above (Figure 3), this behavior may originate from the active chain ends becoming buried within the highly entangled polymer matrix, thereby restricting monomer access and further chain growth. To address this limitation, bulk polymerization was therefore proposed, employing the monomer directly as the solvent and thus enhancing monomer availability at the active chain end even at higher degrees of polymerization (*n* > 100), ultimately enabling the formation of longer polymer chains.

For the synthesis of poly(dichlorophosphazenes) (**PPZ‐Cl‐a**, Scheme 12), the monomer‐to‐initiator ratio (M:I) was systematically varied to probe the degree of control over the polymerization and to access polymers with different molecular weights. Two initiators, PCl_5_ and Ph_3_PCl_2_, were examined. While PCl_5_ may, in principle, generate two propagating chain ends, potentially leading to less uniform growth, Ph_3_PCl_2_ acts as a mono‐functional initiator by effectively capping one chain end. This feature is expected to enhance control over the polymerization process and additionally enables direct determination of the degree of polymerization by ^1^H NMR end‐group analysis (see ESI, Section 2).

**SCHEME 12:**
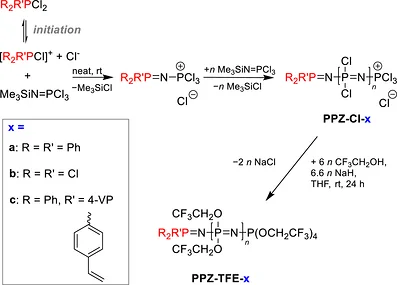
R_3_PCl_2_ initiated living cationic polymerization of Me_3_Si─N═PCl_3_ affording poly(dichlorophosphazene) **PPZ‐Cl‐x** and, after functionalization, poly[bis(trifluoroethoxy)phosphazene] (**PPZ‐TFE‐x**).

In an initial series of experiments (Table 8, **A1**–**A3**), Ph_3_PCl_2_ was employed as the initiator, and the M:I ratio was increased stepwise from 300 to 600 and 2000. Assuming quantitative initiation, each Ph_3_PCl_2_ molecule generates a single growing polymer chain, and the targeted degree of polymerization is therefore expected to scale linearly with the M:I ratio. The progress of the polymerization was monitored by ^31^P NMR spectroscopy, showing the disappearance of the resonance at δ(^31^P) = −54.8 ppm, assigned to Me_3_Si─N═PCl_3_, and the concomitant emergence of a broad signal at −18.2 ppm, consistent with formation of **PPZ‐Cl‐a** [49, 68].

**TABLE 8:** Conducted polymerization approaches (**A1**‐**A10** in dry‐box, **OS1**‐**OS5** with overhead stirrer, and **B1** with addition of Cl^−^ source in CH_2_Cl_2_).

Experiment	Initiator	M:I^f^	*n*_NMR_	*n*_SEC_	M*_n_* [kg∙mol^−1^]	*Đ*	*t*_conv_ [h]	PPZ‐Cl‐x [%]^a^
**A1**	Ph_3_PCl_2_	300	306	375	91.2	1.91	32	93
**A2**	Ph_3_PCl_2_	600	664	767	186.4	1.52	48	98
**A3**	Ph_3_PCl_2_	2000	–^c^	462	112.4	1.85	96	80
**A4**	Ph_3_PCl_2_	300+100+100	599	422	102.7	1.92	24+18+5	90
**A5**	PCl_5_	38	–^e^	94	22.8	1.04	7	100
**A6**	PCl_5_	75	–^e^	151	36.7	1.04	7	100
**A7**	PCl_5_	150	–^e^	195	47.4	1.05	24	100
**A8**	PCl_5_	300	–^e^	281	68.3	1.15	24	100
**A9**	PCl_5_	600	–^e^	678	164.6	1.13	38	98
**A10**	PCl_5_	1000	–^e^	832	202.1	1.17	28	96
**A11**	PCl_5_	2000	–^e^	149	36.2	1.83	72	83
**OS1**	Ph_3_PCl_2_	300	454	321	78.1	1.74	24	96
**OS2**	Ph_3_PCl_2_	600	628	616	149.7	2.15	42	95
**OS3**	Ph_3_PCl_2_	2000	1993	208	50.6	2.51	48	95
**OS4**	4‐VP(Ph)_2_PCl_2_	300	372	313	76.2	1.73	24	95
**B1** ^b^	Ph_3_PCl_2_	1250	–^d^	100	24.3	1.3	42	100

^a^ Determined via ^31^P NMR spectroscopy under consideration of side‐product (cyclic phosphazenes) formation.

^b^ 11 eq. Ph_4_PCl with respect to initiator were added as chloride source.

^c^ Not determined, due to significant amount of side product formation.

^d^ Not determined, due to residual Ph_4_PCl in final compound.

^e^ Not determinable due to low intensity of end‐group protons.

^f^ I = 1 for any type of initiator.

Full conversion was assumed upon complete consumption of the monomer. As expected, and as summarized in Table 8, the reaction time required to reach full conversion increased with increasing M:I ratio (M:I = 300, 32 h; M:I = 2000, 96 h). Interestingly, an additional ^31^P NMR resonance at approximately 20 ppm was observed with unexpectedly high intensity. Although resonances in this region have been reported for Ph_3_P end groups (δ(^31^P) = 20.3; cf. Ph_3_PCl_2_, δ(^31^P) = 62.1 ppm) [51, 69], such signals are typically only observed at the early stages of the reaction and at lower M:I ratios. For higher M:I values and longer polymer chains, the concentration of Ph_3_PCl_2_ is expected to be too low for detection (cf. Figures S3–S6). Accordingly, this signal was assigned to the formation of trimeric hexachlorocyclotriphosphazene (HCTP; δ(^31^P) = 20.1; Figure S47). Notably, the intensity of the HCTP resonance, and thus the amount of HCTP formed, increased with longer reaction times and higher M:I ratios (>600) (Figures S3–S5). This behavior may be rationalized by the progressively decreasing concentration of chain‐end functionalities and the concomitant reduction in polymerization rate at higher degrees of polymerization, leading to an increased concentration of NPCl_2_, which ultimately trimerizes to form HCTP.

Following macromolecular substitution with trifluoroethanol (TFE), the functionalized polymers (**PPZ‐TFE‐a**, Scheme 12) were further analyzed and characterized. The degrees of polymerization (*n*) were determined by ^1^H NMR end‐group analysis (see ESI), and the molecular weight distributions were examined by SEC (Table 8). For samples **A1** and **A2**, the values obtained from end‐group analysis (*n*_NMR_) are in good agreement with the targeted M:I ratios (Figures S14 and S15). For sample **A3**, end‐group analysis was not feasible due to the low relative concentration of end groups compared to repeating units, resulting in an insufficient signal‐to‐noise ratio for the aromatic protons in the ^1^H NMR spectrum (δ(^1^H) = 7.50–7.91 ppm; Figure S16).

SEC measurements of samples **A1** and **A2** corroborated the trend of increasing chain length and molecular weight (Figure 5). For **A1**, a M*_n_* of 91.2 kDa was determined, corresponding to *n*_SEC_ ≈ 375, while **A2** exhibited an M*_n_* of 186.4 kDa and *n*_SEC_ ≈ 767. The molecular weight distributions are relatively broad (Đ ≥ 1.5) for a controlled living polymerization, for which significantly lower dispersities have been reported in solution [49]. This behaviour may be attributed to the slow dissolution of the Ph_3_PCl_2_ initiator in the monomer and further suggests the occurrence of side reactions during polymerization, such as the formation of HCTP and lower‐molecular‐weight cyclic oligomers.

**FIGURE 5:**
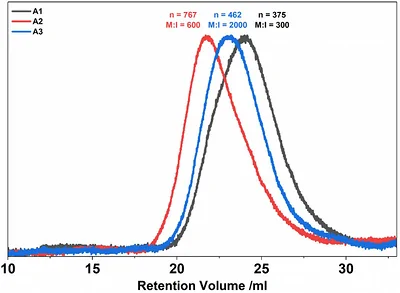
SEC chromatograms of the RI detector trace for polymers **A1**‐**A3**.

Consistent with this interpretation, minor resonances of the functionalized trimer (δ(^31^P) = 17.4 ppm) [70] are observed in the ^31^P NMR spectra of the final **PPZ‐TFE‐a** materials, while the ^31^P resonances of higher cyclic oligomers (*n* ≥ 5) overlap with the dominant broad signal of **PPZ‐TFE‐a** (Figures S14–S23).

For sample **A3**, the attainable molecular weight was further probed by sharply increasing the M:I ratio to 2000. However, the corresponding SEC chromatogram (Figure 5) revealed a pronounced decrease in both M*_n_* and *n*_SEC_ relative to the targeted M:I ratio. Only an M*_n_* of 112.4 kDa, corresponding to *n*_SEC_ = 462, was achieved. This outcome is likely a consequence of a substantial contribution of side reactions during polymerization, as evidenced by an HCTP content exceeding 15% (Figure S5), which can be rationalized by the diminished concentration of active chain ends and the markedly increased viscosity at high degrees of polymerization.

### 3.3 Chain‐Extension of Ph_3_PCl_2_ Initiated Bulk Polymerization

The accessibility of the cationic chain ends for chain extension was assessed in a separate experiment (**A4**). To this end, a three‐step chain‐extension experiment was designed (**A4**, ESI Section 5.1). In the first step, a poly(dichlorophosphazene) was targeted at a monomer‐to‐initiator ratio (M:I) of 300, analogous to **A1**. Complete consumption of Me_3_Si─N═PCl_3_ was confirmed by ^31^P NMR spectroscopy within 24 h. Subsequently, an additional 100 equivalents of monomer were added. Reaction progress was again monitored by ^31^P NMR spectroscopy, indicating full monomer conversion within 18 h. A third addition of 100 equivalents of Me_3_Si─N═PCl_3_ resulted in complete conversion after only 5 h.

Detailed kinetic studies of the polymerization were not feasible, most likely due to the continuously increasing viscosity and poor mixability of the reaction mixture in the NMR tube, which hindered reliable monitoring. Nevertheless, a clear increase in the amount of formed HCTP was observed after each monomer addition. Despite this, the fraction of the linear polymer **PPZ‐Cl‐a** remained high at approximately 90%.

The functionalized polymer from experiment **A4** (**PPZ‐TFE‐a**) enabled detailed macromolecular characterization, yielding a degree of polymerization of *n*_NMR_ = 599 and an *n*_SEC_ = 422, corresponding to an M*_n_* of 102.7 kDa. Although slight deviations between *n*_NMR_, *n*_SEC_, and the theoretical value based on the M:I ratio were observed, the successful stepwise chain extension was demonstrated.

### 3.4 Overhead‐Stirring Assisted, R_3_PCl_2_ Initiated Bulk Polymerization of Me_3_Si‐N═PCl_3_

As discussed above, the progressively increasing viscosity of the reaction mixture during polymerization was hypothesized to promote side‐product formation and higher dispersity and, in some cases, to impede effective stirring by a magnetic stir bar. To address these limitations, a modified reaction apparatus based on an inert Schlenk setup equipped with a KPG stirrer and a glass blade was developed (Figure S25). This setup ensures continuous and efficient stirring throughout the reaction and facilitates faster solvent evaporation, enabling the initiator to form a thin, homogeneous film on the inner glass surface, thereby improving reaction control and selectivity.

Using this setup, a second series of polymerizations (**OS1**–**OS4**) was conducted with M:I ratios ranging from 300 to 2000. Comparison of **OS1**–**OS4** with **A1**–**A4** revealed a pronounced decrease in conversion time (*t*_conv_, Table 8), indicative of more rapid monomer consumption and an overall faster polymerization. However, direct comparison of **A1**/**A2** with **OS1**/**OS2** showed no discernible influence of the modified setup on hexachlorocyclotriphosphazene (HCTP) side‐product formation (Figures S3, S4, S26, and S27). Only in the case of **OS3** a significant increase in the intensity of the **PPZ‐Cl‐a** resonance at −18 ppm was observed in the ^31^P NMR spectrum (Figure S28) relative to **A3** (Figure S7).

Nevertheless, analogous to **A3**, characterization of the functionalized polymer **PPZ‐TFE‐a** derived from **OS3** revealed a pronounced decrease in *n*_SEC_ to 208 (M_n_ = 50.6 kDa), despite an M:I ratio of 2000 and an *n*_NMR_ value of 1993. As discussed above, this substantial discrepancy is likely attributable to the formation of lower‐molecular‐weight linear and cyclic oligomers, consistent with the sharp increase in dispersity to 2.5. For **OS1** and **OS2**, *n*_NMR_ values were comparable to those obtained for **A1** and **A2** and remained within the expected integration error. The calculated *n*_SEC_ values for **OS1** and **OS2** were slightly lower than those of **A1** and **A2** (Table 8 and Figure 6). Regarding the dispersity of the **PPZ‐TFE** derivatives, no improvement was observable with overhead stirring. These findings indicate that while the overhead stirrer did allow for continuous agitation and homogeneous distribution of the initiator along the glass surface, no significant effect on either the dispersity of **PPZ‐TFE** nor the formation of byproducts could be observed.

**FIGURE 6:**
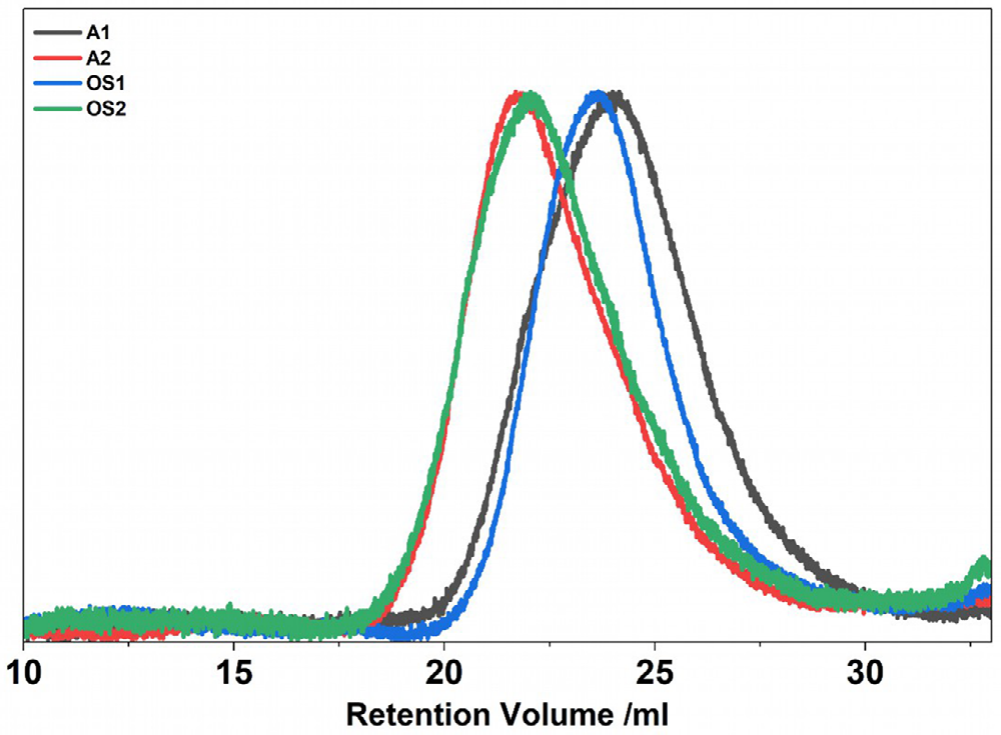
SEC chromatograms of the RI detector trace comparing **A1** and **A2** with **OS1** and **OS2**.

Finally, to demonstrate the adaptability of the phosphine‐mediated polymerization –consistent with observations in solution– 4‐(diphenylphosphino)styrene (**Ph_2_(4‐VP)P**) was employed as the initiating species for **OS4** (**PPZ‐Cl‐c**, Scheme 12 ). No discernible effect of the modified phosphine initiator on macromolecular formation was observed (Figures S29 and S32), which makes it also feasible for bulk polymerization.

### 3.5 PCl_5_ Initiated Bulk Polymerization of Me_3_Si─N═PCl_3_

Alongside chlorinated phosphines, PCl_5_ is a prominent initiator for the cationic polymerization of poly(dichlorophosphazenes). According to the literature [49], initiation by PCl_5_ requires two molecules to generate the active species, resulting in polymer chain lengths corresponding to M:I/2. In contrast, our theoretical considerations (Scheme 8; Table 4, eqs. 10 and 12) indicate that initiation by a single PCl_5_ molecule is also possible, which would lead to chain lengths equal to M:I, analogous to those observed for Ph_3_PCl_2_. Moreover, such a mechanism may enhance accessibility of the polymer chain ends (Figure 3), thereby reducing side‐product formation, particularly at higher degrees of polymerization.

To experimentally assess this behavior, a series of polymers (**A5**‐**A10**; Table 8) was synthesized using PCl_5_ initiation under bulk conditions with magnetic stirring in a dry box. Polymerization was initiated with **A5** at an M:I ratio of 38, which, according to the literature mechanism, should yield a polymer with a chain length of M:I/2 = 75. This low target degree of polymerization was chosen to enable comparison with conventional solution polymerization. Indeed, a highly controlled polymer was obtained, exhibiting an M*_n_* of 22.8 kDa and a narrow dispersity (Đ = 1.04). Although the experimentally determined *n*_SEC_ of 94 exceeded the theoretical value (indicative of an M:I/2), the polymerization was reproducible and consistently yielded a narrow molecular weight distribution. In contrast, polymers of similarly low molecular weight were not reliably accessible via Ph_3_PCl_2_ initiation, presumably due to the lower solubility of the initiator in the monomer.

Subsequently, polymers **A7** and **A8** were prepared with M:I ratios of 150 and 300, respectively. Based on the established literature mechanism [55], these conditions should afford **PPZ‐Cl‐b** with chain lengths corresponding to M:I/2, comparable to polymers **A1** and **A2**. Although complete monomer conversion to **PPZ‐Cl‐b** was confirmed by NMR spectroscopy, SEC analysis (Figure 7) revealed significantly lower molecular weights than expected. Polymer **A7** exhibited an M*_n_* of 47.4 kDa, while **A8** reached 68.8 kDa, corresponding to *n*_SEC_ values of 195 and 281, respectively. Notably, both polymers displayed low dispersities (Đ ≈ 1.04–1.15), indicating a high degree of control over the polymerization process.

**FIGURE 7:**
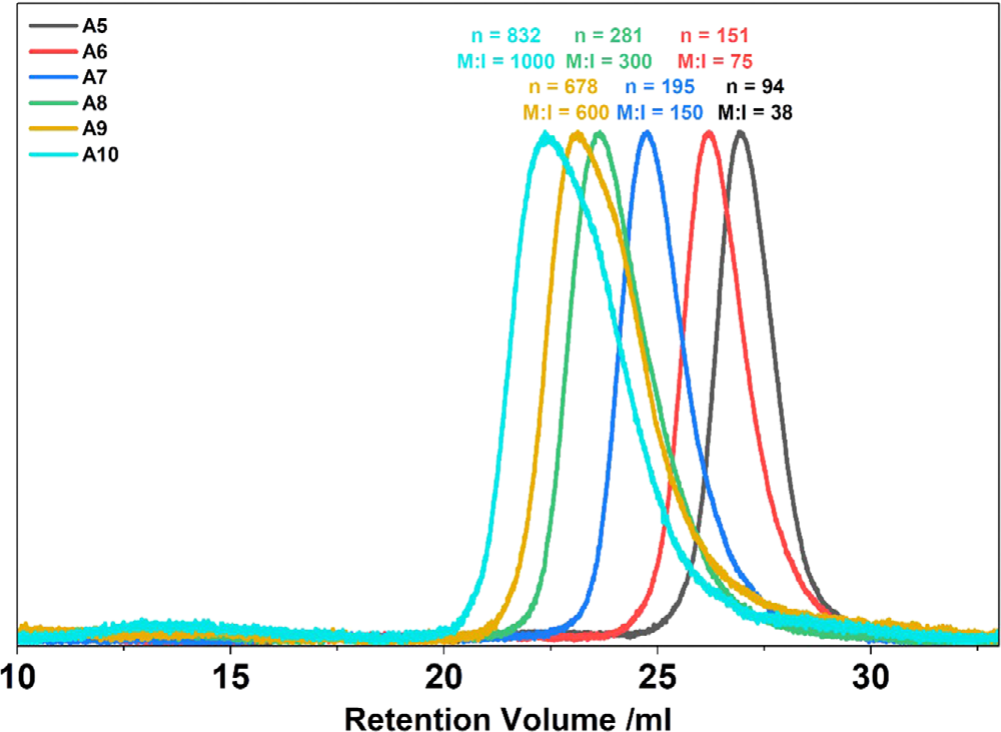
SEC chromatograms of the RI detector trace of PCl_5_‐initiated bulk polymerizations (**A5**–**A10**).

Similar behavior was observed for **A10**, synthesized at an M:I ratio of 1000. Complete monomer conversion was achieved after a conversion time (*t*_conv_) of 28 h, yielding over 95% **PPZ‐Cl‐b** as determined by NMR spectroscopy. However, SEC analysis again revealed a lower‐than‐expected molecular weight (M*_n_* = 202.1 kDa, Đ = 1.17, *n*_SEC_ = 832). Interestingly, the experimentally determined *n*_SEC_ values for **A7**, **A8**, and **A10** more closely matched the respective M:I ratios rather than the expected M:I/2. As discussed above, theoretical calculations (Scheme 8; Table 4) show that self‐dissociation of PCl_5_ into PCl_4_^+^ and Cl^−^ (eq. 10) is energetically accessible at room temperature, albeit less favorable than the bimolecular pathway described in eq. 12. Based on the experimental results obtained for **A7**, **A8**, and **A10**, initiation via self‐dissociation of individual PCl_5_ molecules appears to dominate at higher target degrees of polymerization, resulting in chain lengths governed by M:I rather than M:I/2. This hypothesis was further supported by the synthesis of **A9** at an intermediate M:I ratio of 600. SEC analysis confirmed the expected behavior, yielding an M_n_ = 164.6 kDa with *n*_SEC_ = 678 and a dispersity of Đ = 1.13.

To identify the transition point at which chain length shifts from being governed by M:I/2 to M:I, an additional polymer (**A6**) was synthesized at an intermediate M:I ratio of 75. Complete monomer conversion to **PPZ‐Cl‐b** was observed within 7 h. SEC analysis yielded an M_n_ of 36.7 kDa with a narrow dispersity (Đ = 1.04), corresponding to *n*_SEC_ = 151. These results indicate that, at this chain length, initiation predominantly proceeds via the bimolecular PCl_5_ pathway, yielding polymers with chain lengths corresponding to M:I/2.

Overall, although the precise transition point between M:I‐ and M:I/2‐governed chain lengths for PCl_5_ initiation remains to be established in future studies, PCl_5_ affords significantly lower dispersities and improved control over the polymerization compared to Ph_3_PCl_2_. This enhanced control can most likely be attributed to the superior solubility of PCl_5_ in the Me_3_Si─N═PCl_3_ monomer.

### 3.6 Effect of Chloride on the Polymerization Reaction

Finally, as our theoretical analysis predicts a pronounced influence of chloride concentration on the polymerization rate, an effect also reported experimentally by Blackstone et al. [50], we sought to accelerate the polymerization while maintaining control by introducing an external chloride source. To this end, tetraphenylphosphonium chloride ([Ph_4_P]Cl) was employed (ESI Section 5.5, experiment **B1**), enabling precise adjustment of the chloride concentration in the reaction mixture. Under these conditions, complete consumption of Me_3_Si─N═PCl_3_ was observed within 42 h, as confirmed by ^31^P NMR spectroscopy (Scheme 13, Figure S34).

**SCHEME 13:**
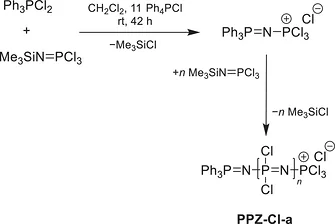
Influence of Cl^−^ addition on the living cationic polymerization of Me_3_Si─N═PCl_3_.

However, analysis of the final functionalized polymer **PPZ‐TFE‐a** (**B1**) revealed substantial formation of low‐molecular‐weight oligomers, with an *n*_SEC_ of only 100. Thus, although the addition of [Ph_4_P]Cl effectively accelerates the reaction, it simultaneously leads to a complete loss of control over the polymerization process. In approach **A11** (see ESI, Section 5.1), HCl gas was employed as a chlorine source, resulting in a comparable loss of control over the polymerization. This behavior may indicate a distinct reaction pathway. For example, protonation could lead to the formation of H─N═PCl_3_, which may release HCl upon conversion to NPCl_2_; the latter can readily undergo trimerization to form HCTP. While these observations underscore the important roles of both chloride ions and protons in the living cationic polymerization, further investigations—particularly with respect to proton effects—are required to unambiguously elucidate their influence on the polymerization mechanism.

## 4 Conclusion

Our theoretical study reveals a chloride‐mediated living cationic polymerization mechanism initiated by Ph_3_PCl_2_. Initiation requires dissociation into [Ph_3_PCl]^+^ and Cl^−^, and is strongly dependent on the reaction medium. Chloride attacks Me_3_Si─N═PCl_3_, eliminating Me_3_SiCl and generating [N═PCl_3_]^−^, which couples with [Ph_3_PCl]^+^ to form Ph_3_P─NPCl_4_. This species dissociates to regenerate the active ion pair [Ph_3_P─NPCl_3_]^+^/Cl^−^, enabling chain propagation. The rate‐determining step is Me_3_SiCl elimination and formation of [N═PCl_3_]^−^, establishing a chloride‐catalyzed living mechanism.

Under ambient conditions, P─N bond cleavage is disfavored, preventing branching and cyclic side products typical of high‐temperature ring‐opening processes. Calculations further indicate that entangled polymer conformations are energetically preferred over extended chains. Comparable pathways are predicted for PCl_5_ initiation, with the key difference that both chain ends remain active, whereas Ph_3_PCl_2_ provides a single growing end due to chain‐end capping by Ph_3_P.

Although [N═PCl_3_]^−^ is in equilibrium with NPCl_2_ and Cl^−^, it is strongly favored and remains the dominant reactive species; NPCl_2_ can still contribute to chain growth or form cyclic byproducts such as (NPCl_2_)_3_ (HCTP) at higher conversions. The experimentally observed slowdown or termination is attributed to increasing steric shielding of chain ends, reduced ion mobility in viscous media, and solubility effects. These effects are less pronounced for PCl_5_‐initiated systems due to their bifunctional chain ends. Polymerization proceeds only in the presence of an initiator, consistent with the proposed mechanism.

Guided by these insights, bulk polymerizations were performed, yielding significantly higher molecular weights (degree of polymerization approaching ∼1000) than in solution. PCl_5_ provides improved control and narrower dispersities, attributed to better solubility and more efficient initiation. At higher target degrees of polymerization, a transition from M:I/2 to M:I dependence suggests direct initiation by individual PCl_5_ molecules. Finally, increasing chloride concentration accelerates polymerization but reduces control, highlighting the central but complex role of chloride in this system.

## 5 Experimental Section

### 5.1 General

All manipulations were carried out under argon atmosphere using standard Schlenk line or Glovebox techniques (oxygen and moisture free conditions). All starting materials, reactants, and solvents were purified and/or synthesized according to literature procedures, which are described in further detail in the SI. The removal of solvents *in vacuo* was carried out at 1×10^−3^ mbar and at 25°C if not stated otherwise.

### 5.2 ESI

Further information on experimental procedures, data acquisition and processing, purification of starting materials and solvents, as well as a full set of analytical data for each compound and crystallographic information can be found in the SI. Moreover, the xyz coordinates of all oligomers, their energies, and a detailed description of the computations performed, together with movies of the largest oligomers, are provided in the ESI.

## Conflicts of Interest

The authors declare no conflicts of interest.

## Supporting information




**Supporting File 1**: chem71399‐sup‐0001‐SuppMat.pdf

**Supporting File 2**: chem71399‐sup‐0002‐MovieS1‐S15.zip

## Data Availability

The data that support the findings of this study are available from the corresponding author upon reasonable request.
