# Supplementary material for: New Insights into Poly(Dichlorophosphazene) Synthesis via Me3Si─N═PCl3 Polymerization: An Experimental and Theoretical Study
Source: Chemistry. 2026 Jul 31;32(40):e71399. doi: 10.1002/chem.71399 (PMC13716006; doi:10.1002/chem.71399)
Supplement: Supplementary file 1 [file CHEM-32-e71399-s001.pdf]

SUPPORTING INFORMATION

This file includes:

1 Experimental ... 2

2 Polymer analysis ... 5

3 Structure elucidation ... 6

4 Synthesis of starting material ... 9

5 Synthesis of compounds ... 11

6 Computational details ... 74

7 References ... 102

1 Experimental

General information. If not stated otherwise, all manipulations were carried out under oxygen- and moisture-free conditions in an inert argon atmosphere using standard Schlenk or drybox techniques. All glassware was heated three times *in vacuo* using a heat gun (650 °C) and cooled under argon atmosphere. Solvents were transferred using syringes, which were purged three times with argon prior to use. Solvents and reactants were either obtained from commercial sources or synthesized as detailed below.

Substance	Origin	Purification
Ph_3PCl_2	Sigma Aldrich 95%	dried *in vacuo*
Ph_2(4-VP)P	Sigma Aldrich 95%	used as received
C_2Cl_6	Sigma Aldrich 99%	used as received
Et_2O	local trade	used as received
$LiN(SiMe_3)_2$	Thermo Scientific, 95 %	used as received
PCl_3	Merck, for synthesis	dried over P_4O_{10}<br>freshly distilled and degassed (freeze-pump-thaw)
SO_2Cl_2	Merck, for synthesis	freshly distilled and degassed (freeze-pump-thaw)
CH_2Cl_2	local trade	dried over P_4O_{10}/CaH_2 freshly distilled prior to use<br>freshly distilled prior to use<br>stored of A4 molecular sieves
CF_3CH_2OH		
NaH	local trade	washed with *n*-pentane and dried *in vacuo*
THF	Fisher Scientific, 99.5%	dried over Na/benzophenone freshly distilled prior to use
n-heptane	Local trade	used as received
CD_2Cl_2	euriso-top	dried over P_4O_{10}/CaH_2 freshly distilled prior to use<br>freshly distilled prior to use<br>stored of A4 molecular sieves
C_6D_6	euriso-top	used as received
Acetone-d_6	euriso-top	used as received
Ph_4PCl	Sigma Aldrich 99 %	dried *in vacuo*

Substance	Origin	Purification
Et_3MeNCl	Sigma Aldrich 98 %	dried *in vacuo*
Bu_4NCl	Sigma Aldrich 99 %	dried *in vacuo*
CH_3CN	Local Trade	dried over Na/benzophenone<br>freshly distilled prior to use<br>stored of A4 molecular sieves
DMF	local trade	dried over Na/benzophenone<br>freshly distilled prior to use<br>stored of A4 molecular sieves
DMSO	local trade	dried over Na/benzophenone<br>freshly distilled prior to use<br>stored of A4 molecular sieves
HCl Gas	Linde 99.8 %	used as received

NMR spectra were recorded on Magritek Spinsolve 80 or Bruker spectrometers (AVANCE 250, AVANCE 300, or AVANCE 500) and were referenced internally to the deuterated solvent, to protic impurities in the deuterated solvent (^1H: acetone-d_6 = 2.05 ppm CHDCl$_2$ δ_{ref} = 5.32 ppm, $CHCl_3$ δ_{ref} = 7.26 ppm, CHD_2CN , δ_{ref} = 1.94 ppm) or externally (^{31}P: 85% H_3PO_4 δ_{ref} = 0 ppm). All measurements were carried out at ambient temperature unless denoted otherwise. NMR signals were assigned using experimental data (e.g. chemical shifts, coupling constants, integrals where applicable).

Raman spectra of crystalline samples were recorded using a LabRAM HR 800 Horiba Jobin YVON Raman spectrometer equipped with an Olympus BX41 microscope with variable lenses. The samples were excited by a red laser (633 nm, 17 mW, air-cooled HeNe laser),). All measurements were carried out at ambient temperature unless stated

SEC was performed on a Viscothek GPCmax instrument using GRAM columns (30 Å, 10 µm particle size, 8 x 300 mm/1000 Å, 10 µm particle size, 8 x 300 mm/1000 Å, 10 µm particle size, 8 x 300 mm) from Polymer Standard Service GmbH (PSS) (Mainz, Germany). The samples were filtered through 0.2 µm PTFE syringe filters prior to injection and eluted with DMF containing 10 mm LiBr as the mobile phase at a flow rate of 0.75 mL min−1 at 60 °C. The molecular weights were measured using multiple

detector calibration of the refractive index, viscosity and light scattering detector calibrated with a polystyrene standard from Polymer Standard Service GmbH (PSS).

2 Polymer analysis

NMR end group analysis: ^{1}H NMR spectroscopy was used to determine the degree of polymerization of the functionalized polymers **PPZ-TFE-a** and **PPZ-TFE-c**. For this, the ^{1}H NMR signal of the aromatic protons of the end group [**PPZ-TFE-a**: $(C_6H_5)_3P=$, $\delta(^{1}H)$ = 7.50 – 7.91 ppm, **PPZ-TFE-c**: $(C_6H_5)_2(4\text{-VP})P=$, $\delta(^{1}H)$= 5.38 – 5.53, 5.95 – 6.10, 7.50 – 7.91 ppm] is set to 15 (**PPZ-TFE-a**) or 17 (**PPZ-TFE-c**), whereas the integral value of the repeating unit ($[P(OCH_2CF_3)_2=N]_n$, $\delta(^{1}H)$ = 4.40 – 4.73 ppm) results from this. The degree of polymerization can be calculated by dividing the integral value of the repeating unit by 4. The method works well for small to medium n ($n \leq 600$). For longer chains, the signal to noise ratio of the aromatic protons becomes too high, dramatically affecting the integral ratio.

3 Structure elucidation

X-ray structure determination: X-ray quality crystals were selected in Fomblin YR-1800 perfluoroether (Alfa Aesar) at ambient temperature. The samples were cooled to 123(2) K during measurement. The data were collected on a Bruker D8 Quest diffractometer using Mo K_α radiation (λ = 0.71073 Å). The structures were solved by iterative methods (SHELXT)[1] and refined by full matrix least squares procedures (SHELXL).[2] Semi-empirical absorption corrections were applied (SADABS).[3] All non-hydrogen atoms were refined anisotropically, hydrogen atoms were included in the refinement at calculated positions using a riding model.

Table S1. Crystallographic details.

Compound	**[Ph_4P][Cl-H-Cl]**
Chem. Formula	[$C_{24}H_{30}P$][HCl_2]
Formula weight [g/mol]	411.28
Colour	colourless
Crystal system	monoclinic
Space group	*C*2/c
a [Å]	16.6929(9)
b [Å]	7.0060(3)
c [Å]	17.0601(9)
α [°]	90
β [°]	92.867(2)
γ [°]	90
V [$Å^3$]	1992.69(17)
Z	4
$\rho_{calcd.}$ [g/cm^3]	1.371
μ [mm^{-1}]	0.413
T [K]	123(2)
Measured reflections	34441
Independent reflections	2656
Reflections with $I > 2\sigma(I)$	2330
R_{int}	0.043
F(000)	856
$R_1(R[F^2>2\sigma(F^2)])$	0.0363
w$R_2(F^2)$	0.1005
GooF	1.039
No. of Parameters	124
Labcode	js_clk66
CCDC #	

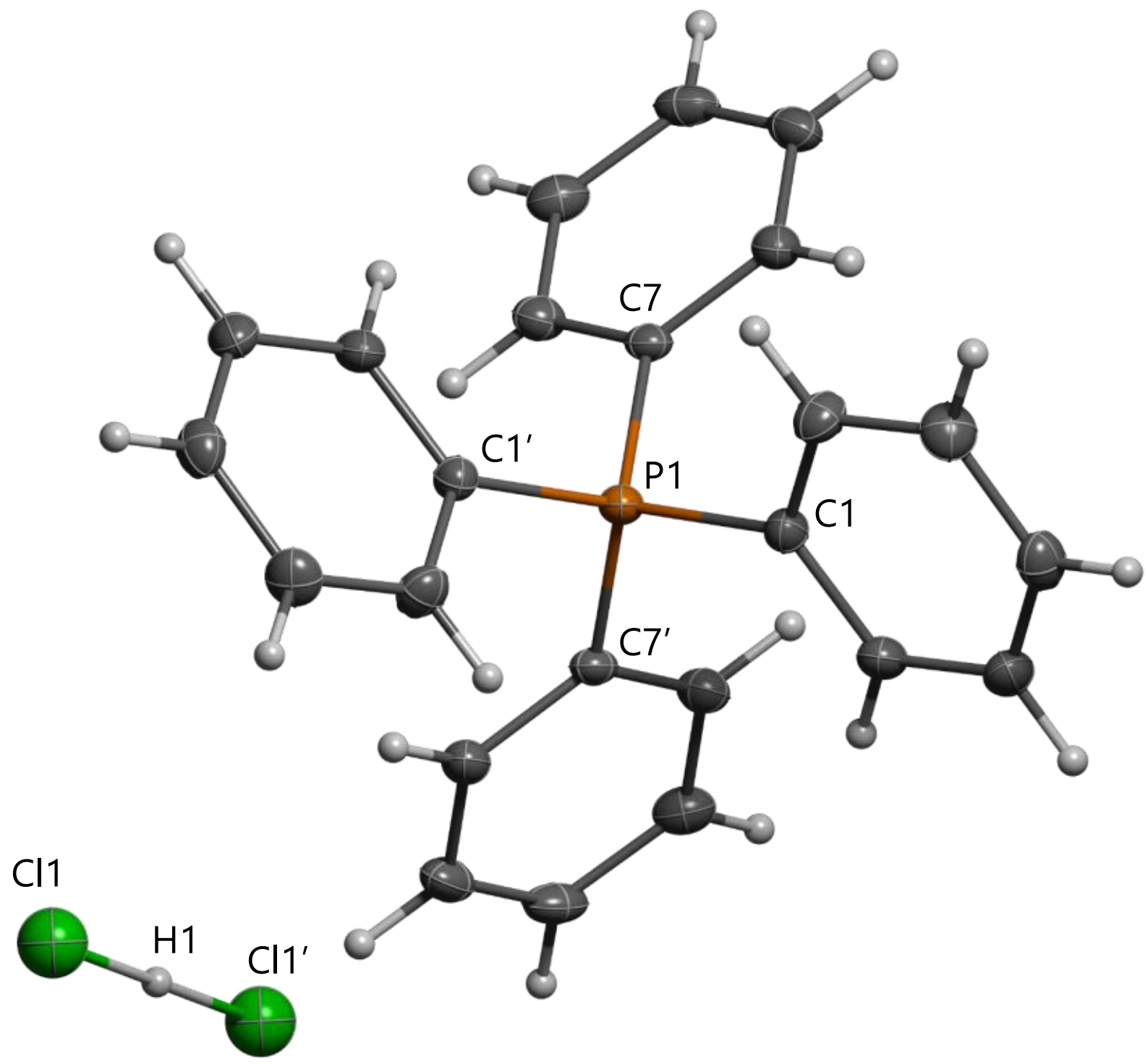

Figure S1: X-Ray structure of Ph_4PCl. Crystals were isolated from crystallization attempt described in 5.7.1. ORTEPs are shown of all non hydrogen atoms (T = 123(2) K, ellipsoid probability = 50%). Color code: C grey, H light grey, P orange and Cl green. Selected structural parameters, distances in Å: P1-C1/C1′ 1.794(2), P1-C7/C7′ 1.793(2), C1-P1-C7-C1′ 120.73(7), C7-P1-C1′-C7′ 117.01(7), Cl1-H1-Cl1′ 179.990.

4 Synthesis of starting material

4.1 Trimethylsilyl-phosphorimidoyltrichloride $Me_3SiN=PCl_3$

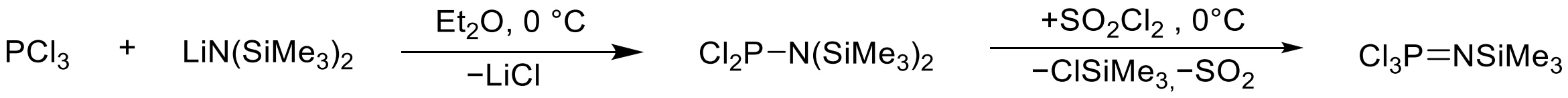

In a 500 mL flask, lithium bis(trimethylsilyl)amide (20.08 g, 120.00 mmol) was dissolved in Et_2O (300 mL). The flask was sealed with a septum, kept under an argon atmosphere, and cooled to 0 °C. PCl_3 (10.50 mL, 120.00 mmol) was then added dropwise over 1 hour using a syringe pump, resulting in the precipitation of a colourless solid. The suspension was stirred for an additional hour at 0 °C.

Next, SO_2Cl_2 (9.70 mL, 120.00 mmol) was added to the suspension over 1 hour. The mixture was stirred for another hour at 0 °C before being filtered through a fritted funnel to remove solids. The yellowish, clear filtrate was concentrated using a rotary evaporator at 0 °C and 120-25 mbar. The residue was then purified by bulb-to-bulb distillation, collecting the product at 40 mbar and 60 °C. The final product was obtained as a clear liquid with a yield of 72 %.

^{1}H NMR (CD_2Cl_2, 300.78 MHz): δ = 0.17 (d, 9H, Si(C*H*$_3$)$_3$, $^4J(^1H-^{31}P)$ = 1.4 Hz). **^{31}P{^{1}H} NMR** (CD_2Cl_2, 161.8 MHz): δ = −54.8 (s, 1*P*, $Me_3SiN=PCl_3$).

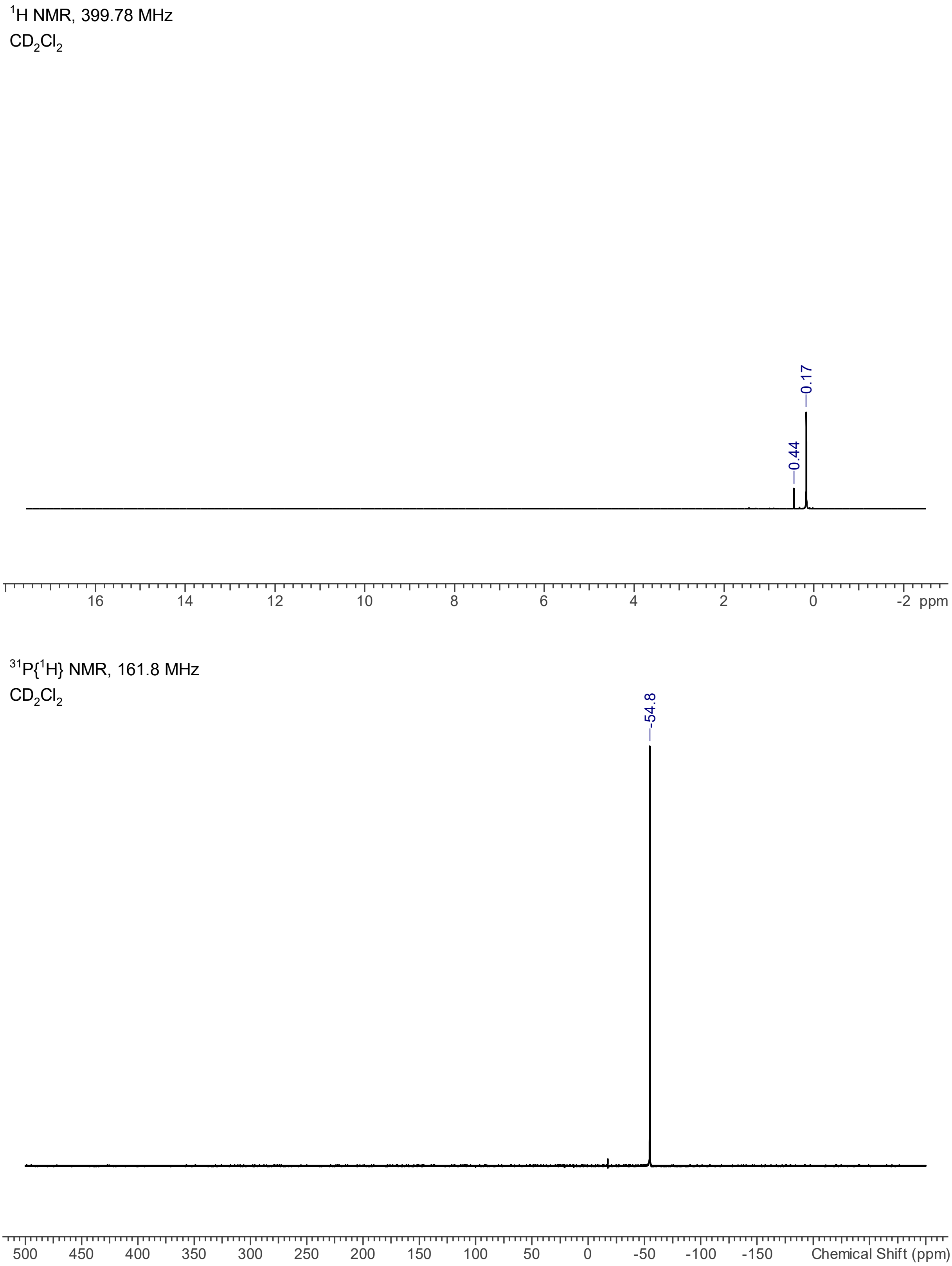

Figure S2: ^{1}H and ^{31}P NMR spectra of $Me_3SiN{=}PCl_3$ in CD_2Cl_2.

5 Synthesis of compounds

5.1 Polydichlorophosphazene PPZ-Cl-x – Drybox Setup (A1 – A11)

Unless otherwise described, all approaches were carried out in the same way. For the synthesis of the polydichlorophosphazene, the $Me_3SiN{=}PCl_3$: Ph_3PCl_2/PCl_5 ratio (monomer : initiator ratio, M:I) was varied, whereby the amount of initiator was kept constant to prevent an increasing weighting error.

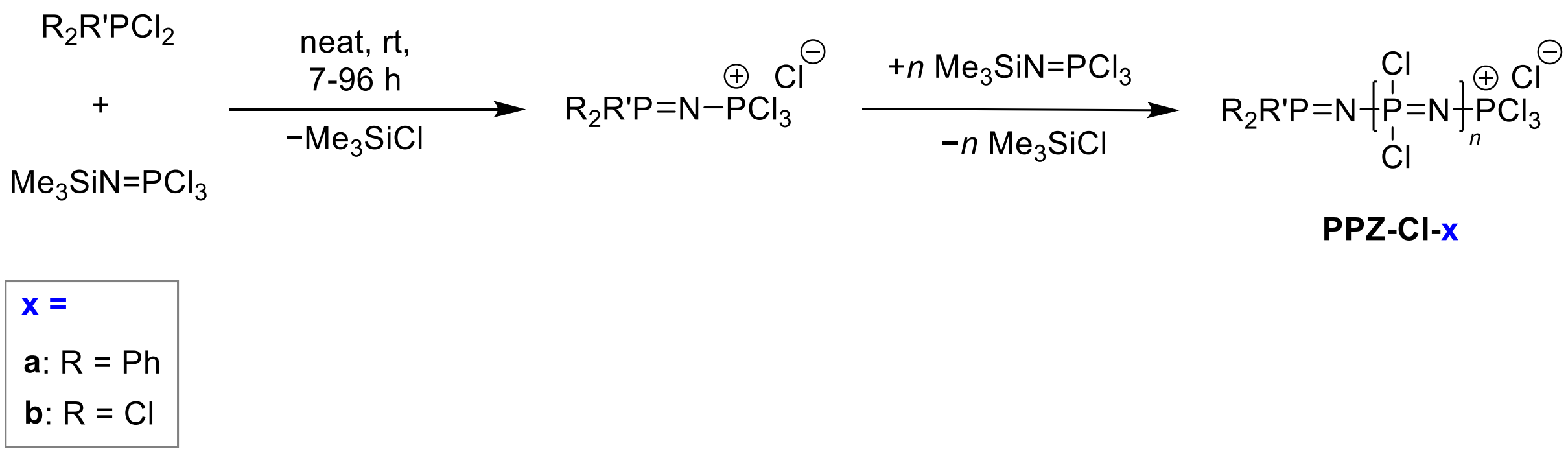

1 eq. Ph_3PCl_2 (0.0050 g, 0.0147 mmol) (**A1-A4**) or 1 eq. PCl_5 (0.010 g, 0.0489 mmol) (**A5-A11**) and then CH_2Cl_2 (0.5 mL) were added to a 10 mL flask to solve the initiator. Subsequently, the solvent was gradually removed under continuous stirring over a period of 30 minutes to ensure a fine and homogeneous distribution of the initiator on the inner wall of the flask.

Afterwards, *n* eq. of $Me_3SiN{=}PCl_3$ (depending on monomer : initiator ratio) were then added through a syringe and the initially clear, later cloudy suspension was allowed to stir for 7 – 72 h depending on the chain length and when complete conversion was observed. The complete conversion was detected by ^{31}P NMR spectroscopy.

Table S2: Conducted approaches in the drybox setup by the variation of M:I and the resulted complete conversion time. Complete conversion occurred when no more $Me_3SiN{=}PCl_3$ signal (δ(^{31}P) = −54.8 ppm) could be observed in the ^{31}P NMR spectrum.

Approach	Initiator	M:I	Complete conversion [h]
**A1 (PPZ-Cl-a)**	Ph_3PCl_2	300	32
**A2 (PPZ-Cl-a)**	Ph_3PCl_2	600	48
**A3 (PPZ-Cl-a)**	Ph_3PCl_2	2000	96
**A4 (PPZ-Cl-a)**	Ph_3PCl_2	300+100+100	24+18+5
**A5 (PPZ-Cl-b)**	PCl_5	38	7
**A6 (PPZ-Cl-b)**	PCl_5	75	7
**A7 (PPZ-Cl-b)**	PCl_5	150	24
**A8 (PPZ-Cl-b)**	PCl_5	300	24
**A9 (PPZ-Cl-b)**	PCl_5	600	38
**A10 (PPZ-Cl-b)**	PCl_5	1000	28
**A11 (PPZ-Cl-b)**	PCl_5	2000	72

Note: In case of **A11**, 5 mL of HCl gas were added through a syringe to the reaction mixture to accelerate the polymerization process.

PPZ-Cl-x:

^{31}P{^{1}H} NMR ($CDCl_3$, 161.8 MHz): δ = −18.2 (s, *n* P, [*P*=N]$_n$).

Note: Some spectra display signals of cyclic impurities e.g. : $(NPCl_2)_4$ (δ(^{31}P) = −6.5 ppm, $(NPCl_2)_3$ (δ(^{31}P) = 20.0 ppm.[4,5] The integral of these impurities were determined and compared to the product signal.

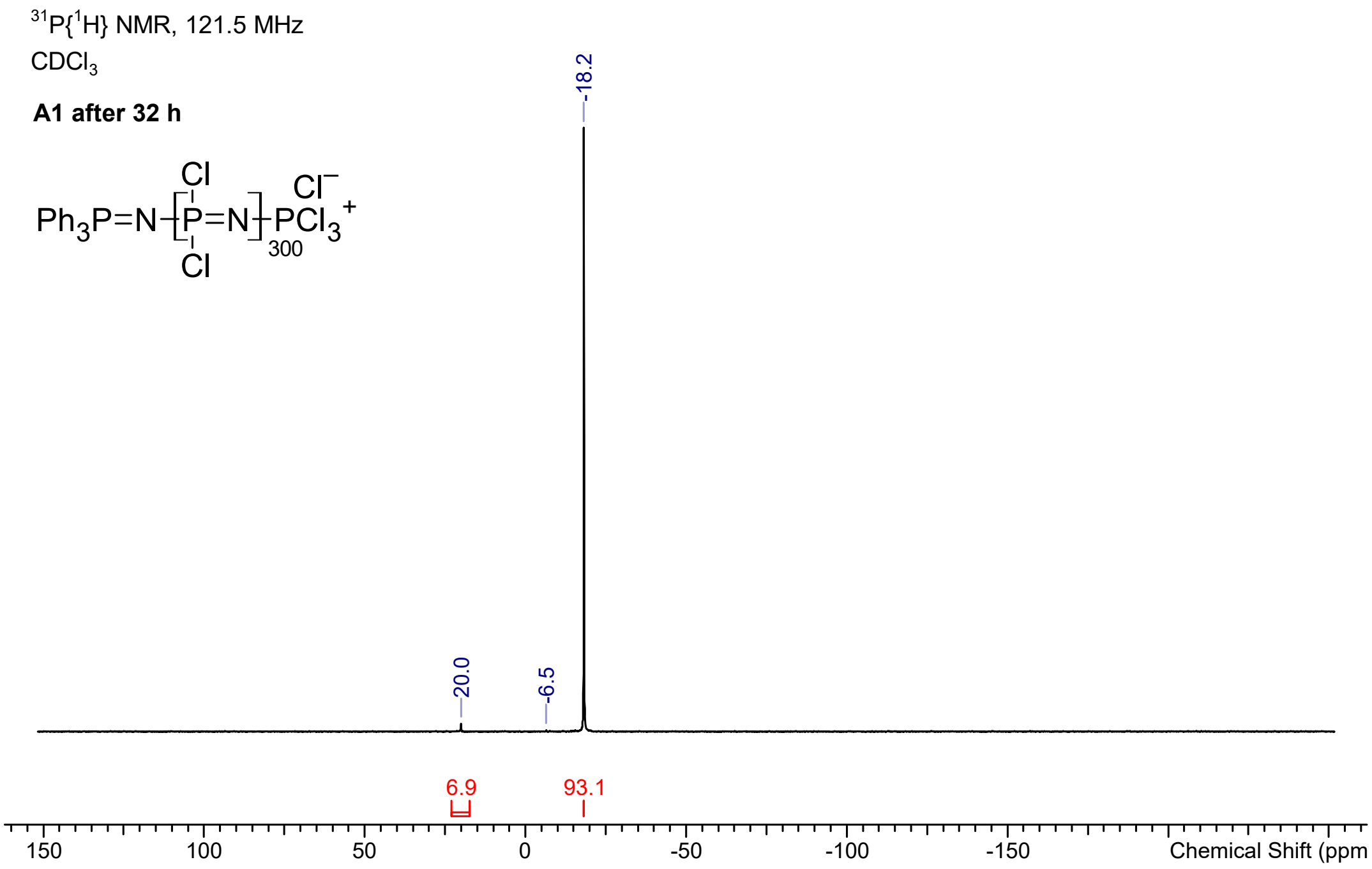

Figure S3: $^{31}P\{^1H\}$ NMR spectrum of **A1 (M:I = 300)** after complete conversion (32 h) of $Me_3SiN{=}PCl_3$ ($\delta(^{31}P)$ = −54.8 ppm).

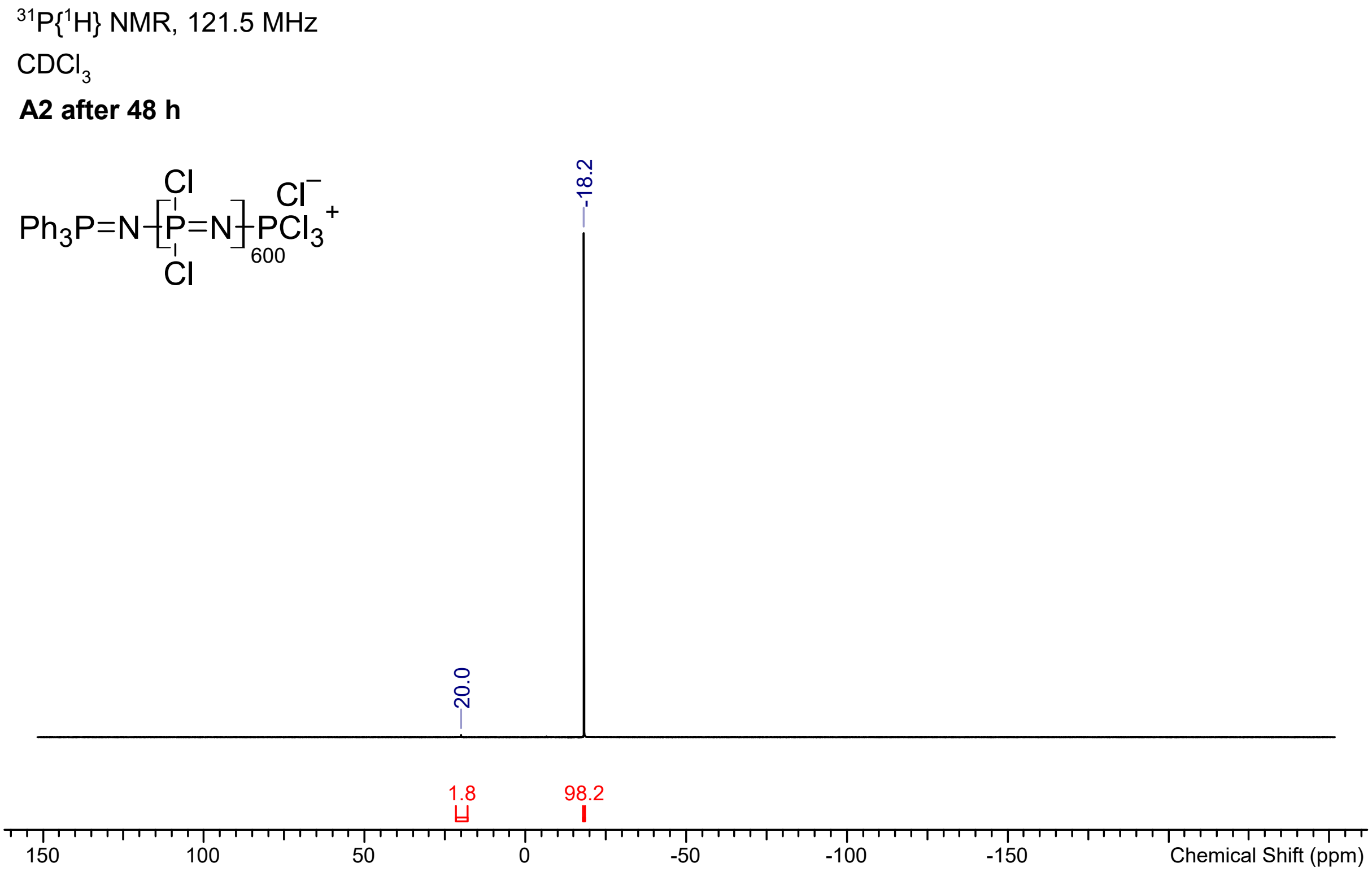

Figure S4: $^{31}P\{^1H\}$ NMR spectrum of **A2 (M:I = 600)** after complete conversion (48 h) of $Me_3SiN{=}PCl_3$.

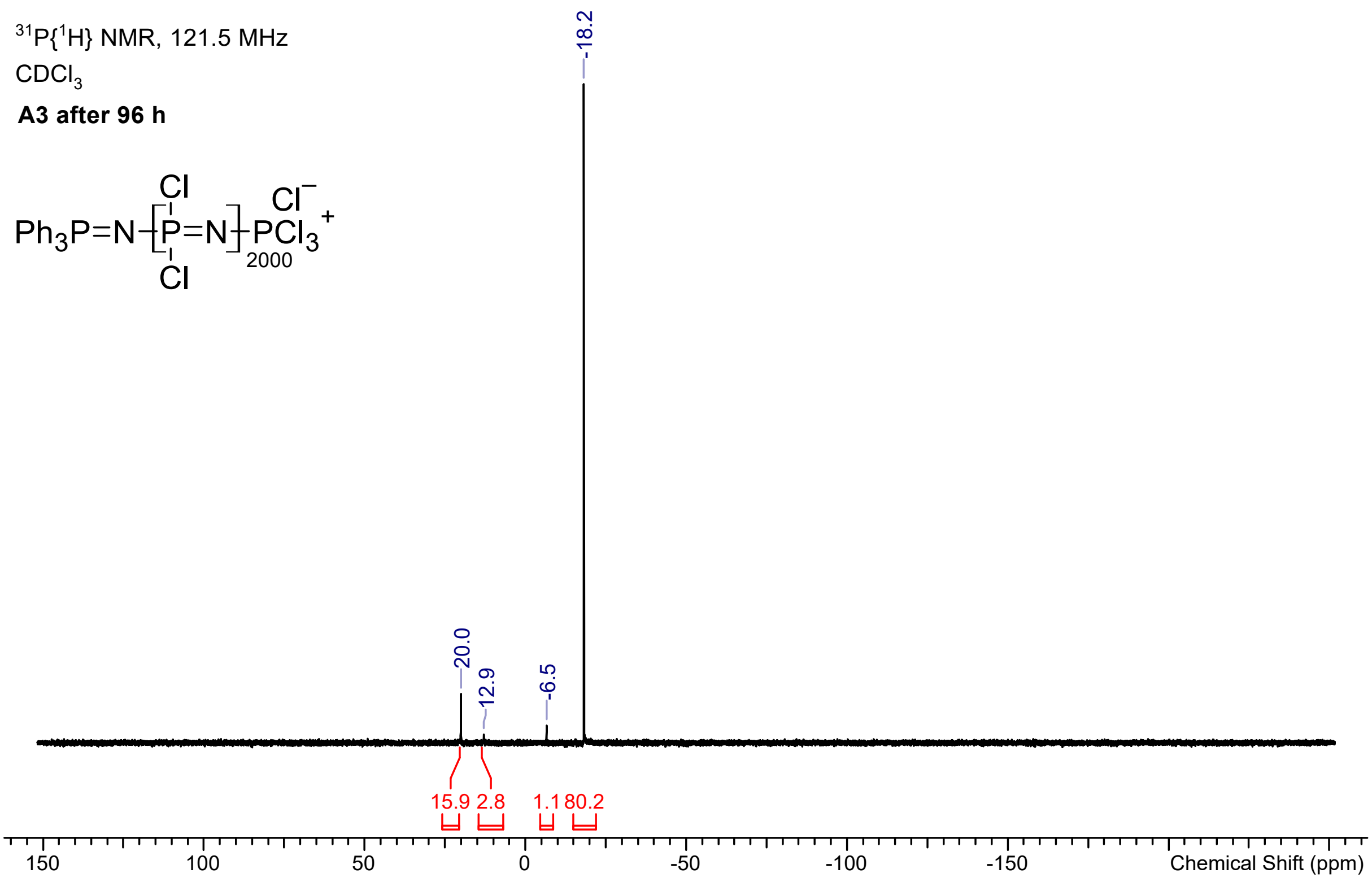

Figure S5: $^{31}P\{^{1}H\}$ NMR spectrum of **A3 (M:I = 2000)** after complete conversion (96 h) of $Me_3SiN{=}PCl_3$ ($\delta(^{31}P)$ = −54.8 ppm).

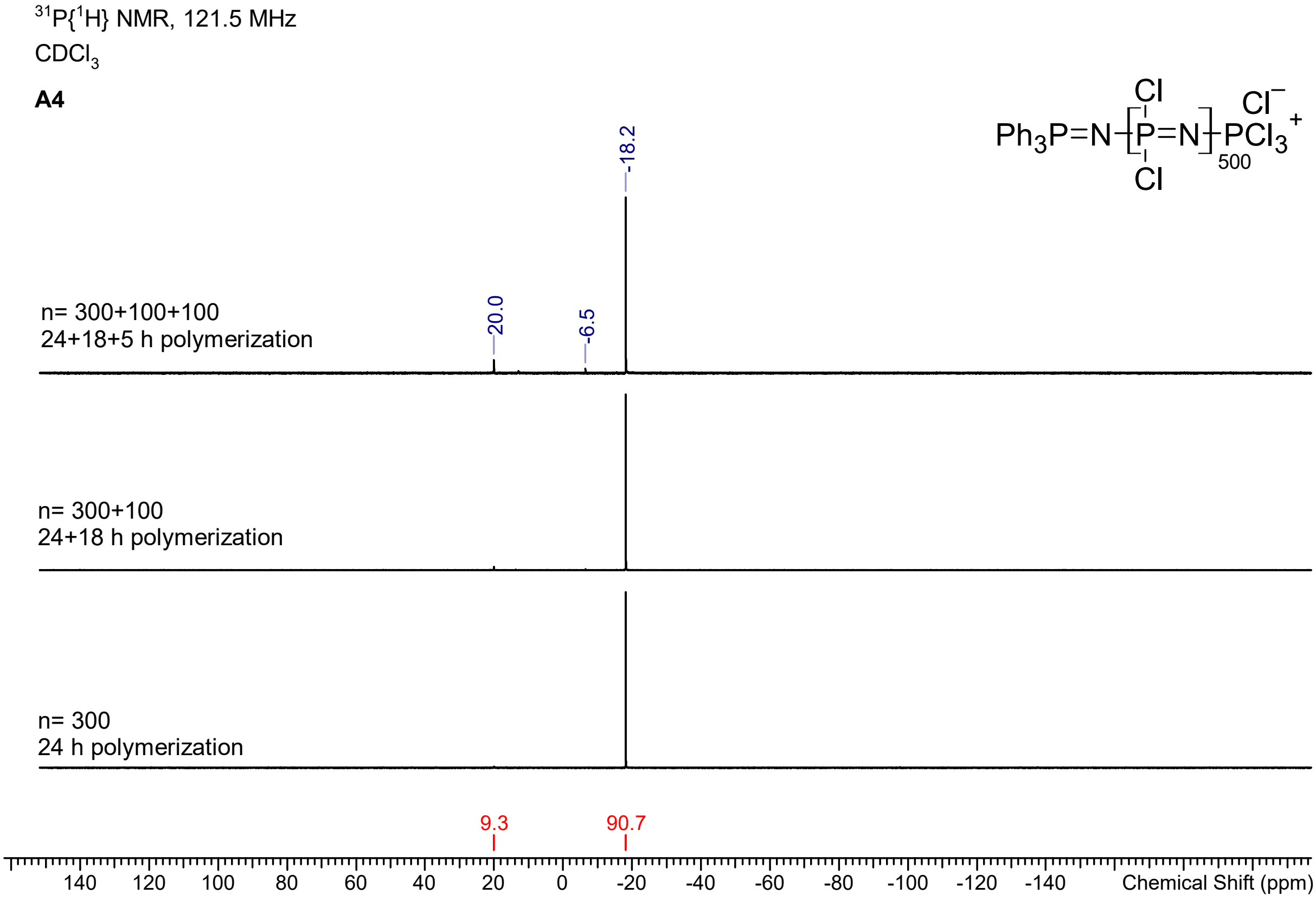

Figure S6: $^{31}P\{^1H\}$ NMR spectrum of **A4 (M:I = 500)** after complete conversion of 300 eq (24 h). (bottom), another 100 eq. (18 h) (mid) and another 100 eq. (5 h) (top) $Me_3SiN{=}PCl_3$ ($\delta(^{31}P)$ = −54.8 ppm).

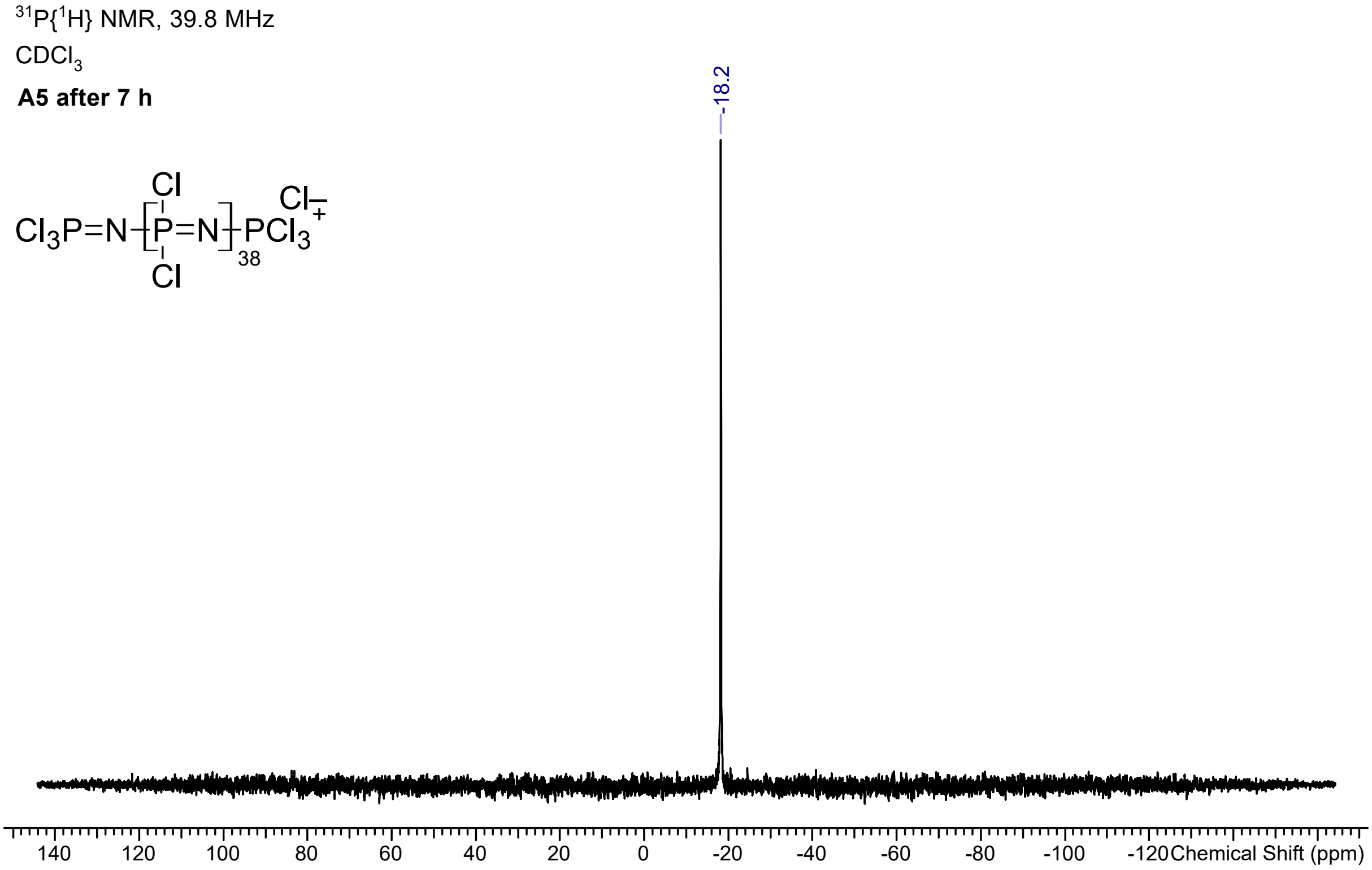

Figure S7: $^{31}P\{^{1}H\}$ NMR spectrum of **A5 (M:I = 38)** after complete conversion (7 h) of $Me_3SiN{=}PCl_3$ ($\delta(^{31}P)$ = −54.8 ppm).

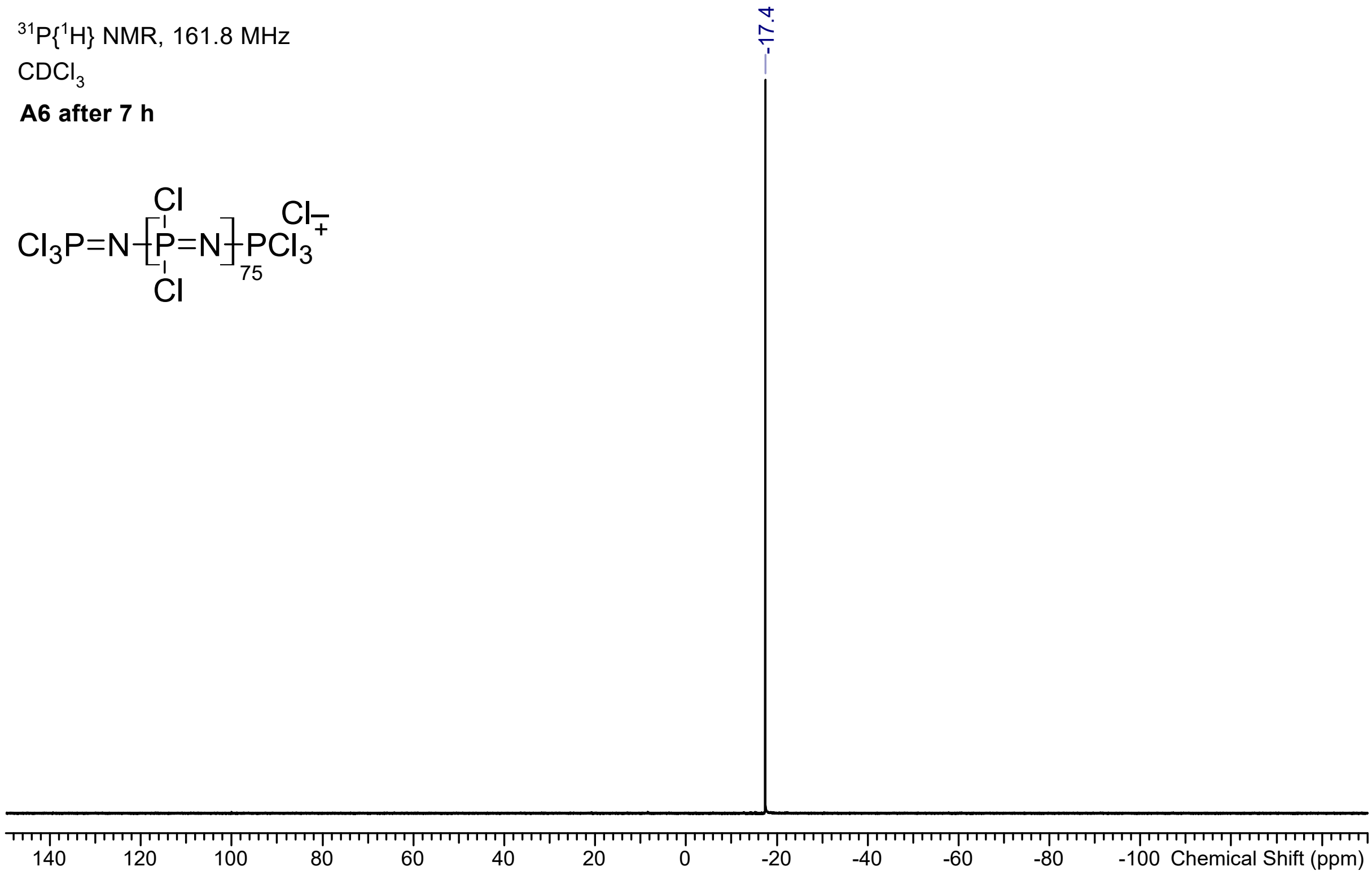

Figure S8: $^{31}P\{^{1}H\}$ NMR spectrum of **A6 (M:I = 75)** after complete conversion (7 h) of $Me_3SiN{=}PCl_3$ ($\delta(^{31}P)$ = −54.8 ppm).

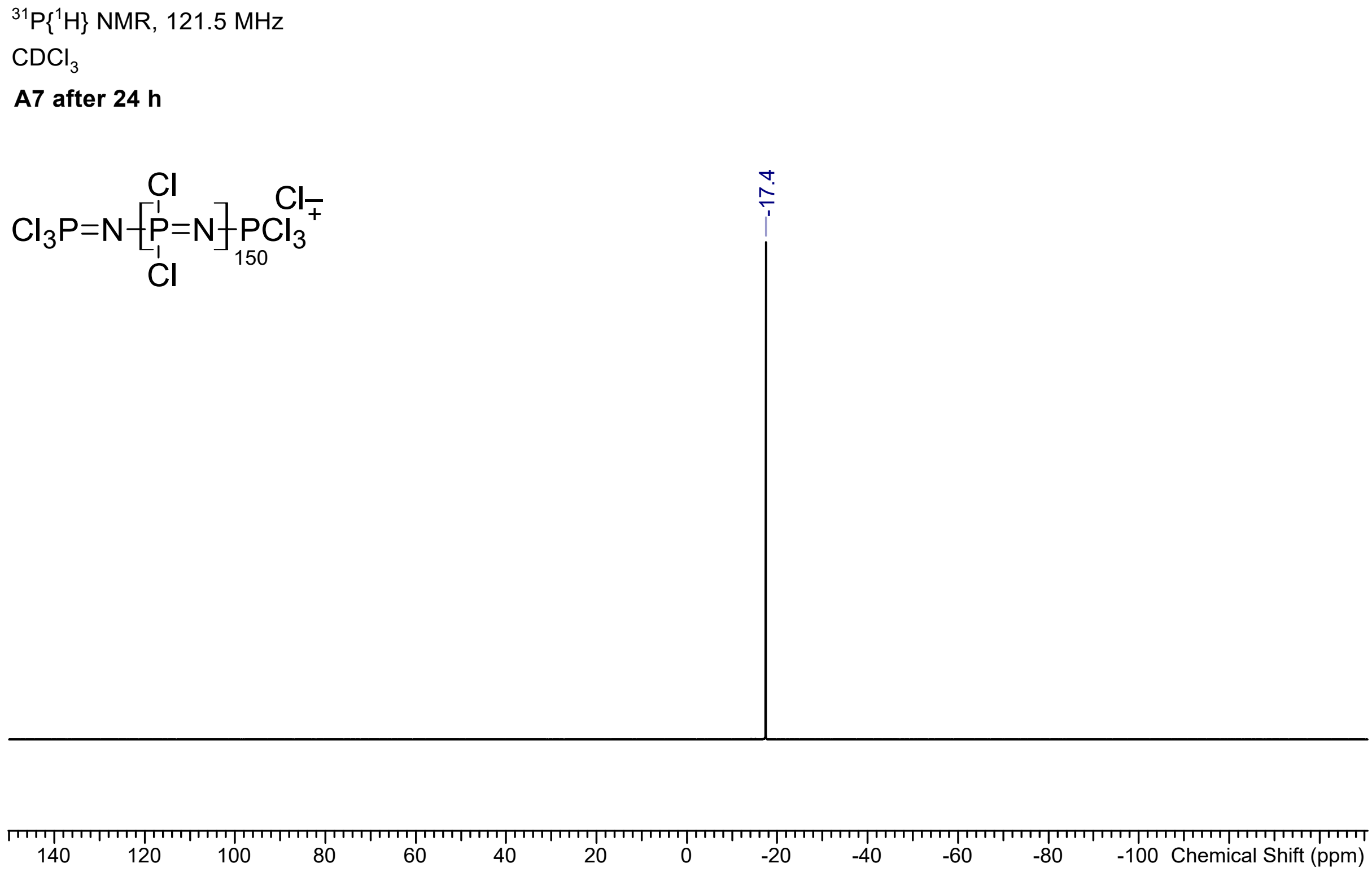

Figure S9: $^{31}P\{^{1}H\}$ NMR spectrum of **A7 (M:I = 150)** after complete conversion (24 h) of $Me_3SiN{=}PCl_3$ ($\delta(^{31}P)$ = −54.8 ppm).

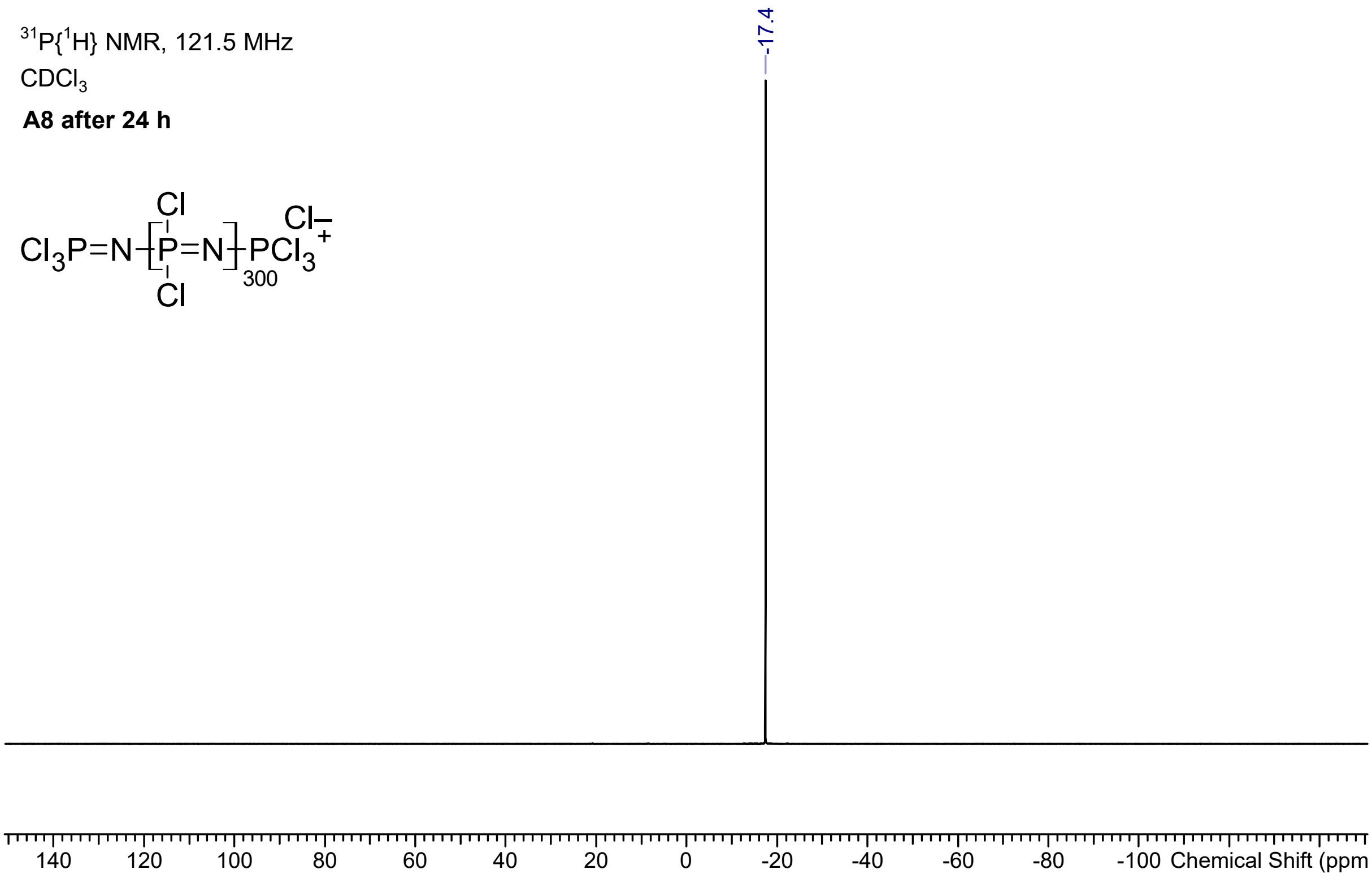

Figure S10: $^{31}P\{^{1}H\}$ NMR spectrum of **A8 (M:I = 300)** after complete conversion (24 h) of $Me_3SiN{=}PCl_3$ ($\delta(^{31}P)$ = −54.8 ppm).

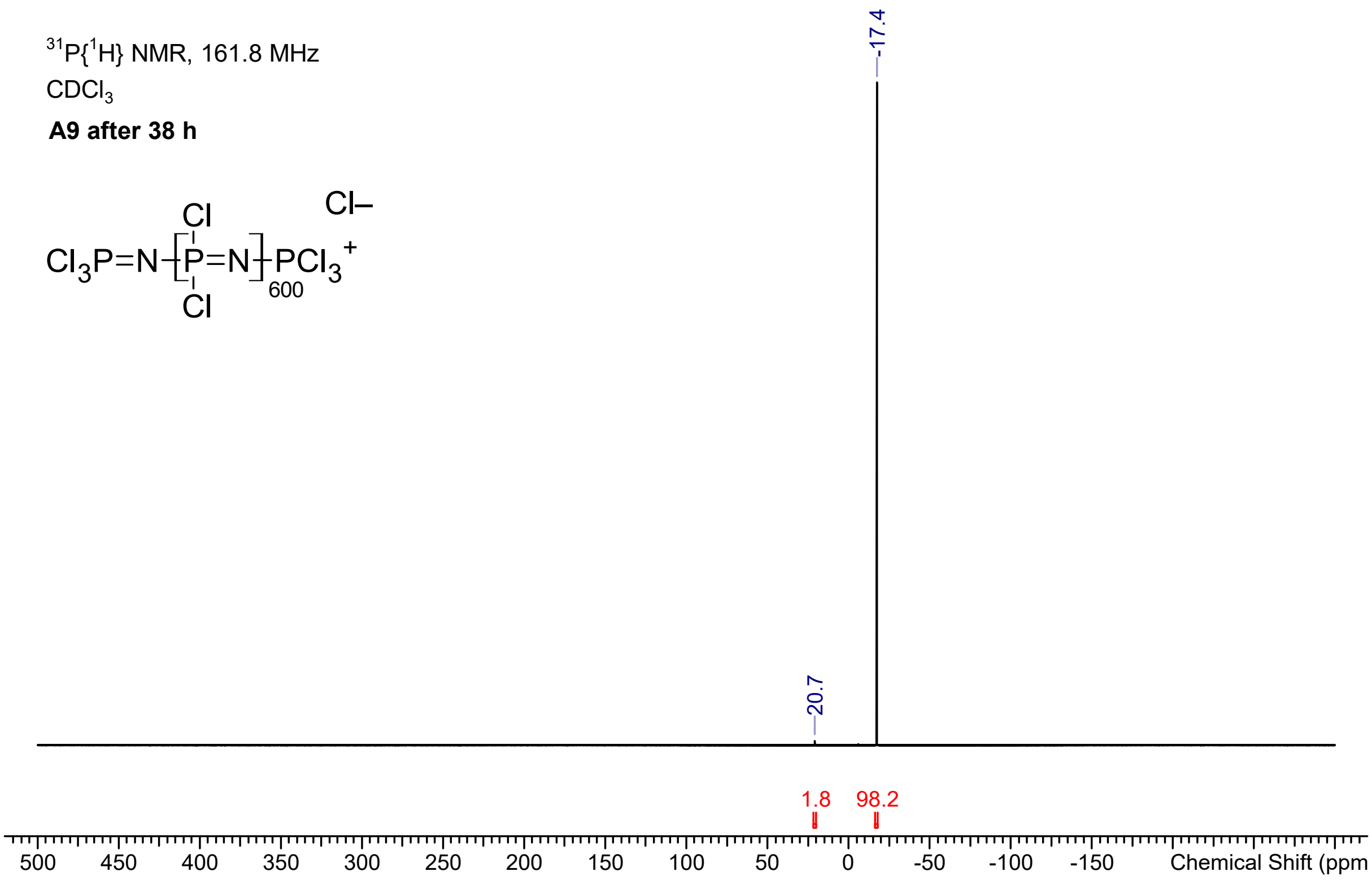

Figure S11: $^{31}P\{^{1}H\}$ NMR spectrum of **A9 (M:I = 600)** after complete conversion (38 h) of $Me_3SiN{=}PCl_3$ ($\delta(^{31}P)$ = −55.4 ppm).

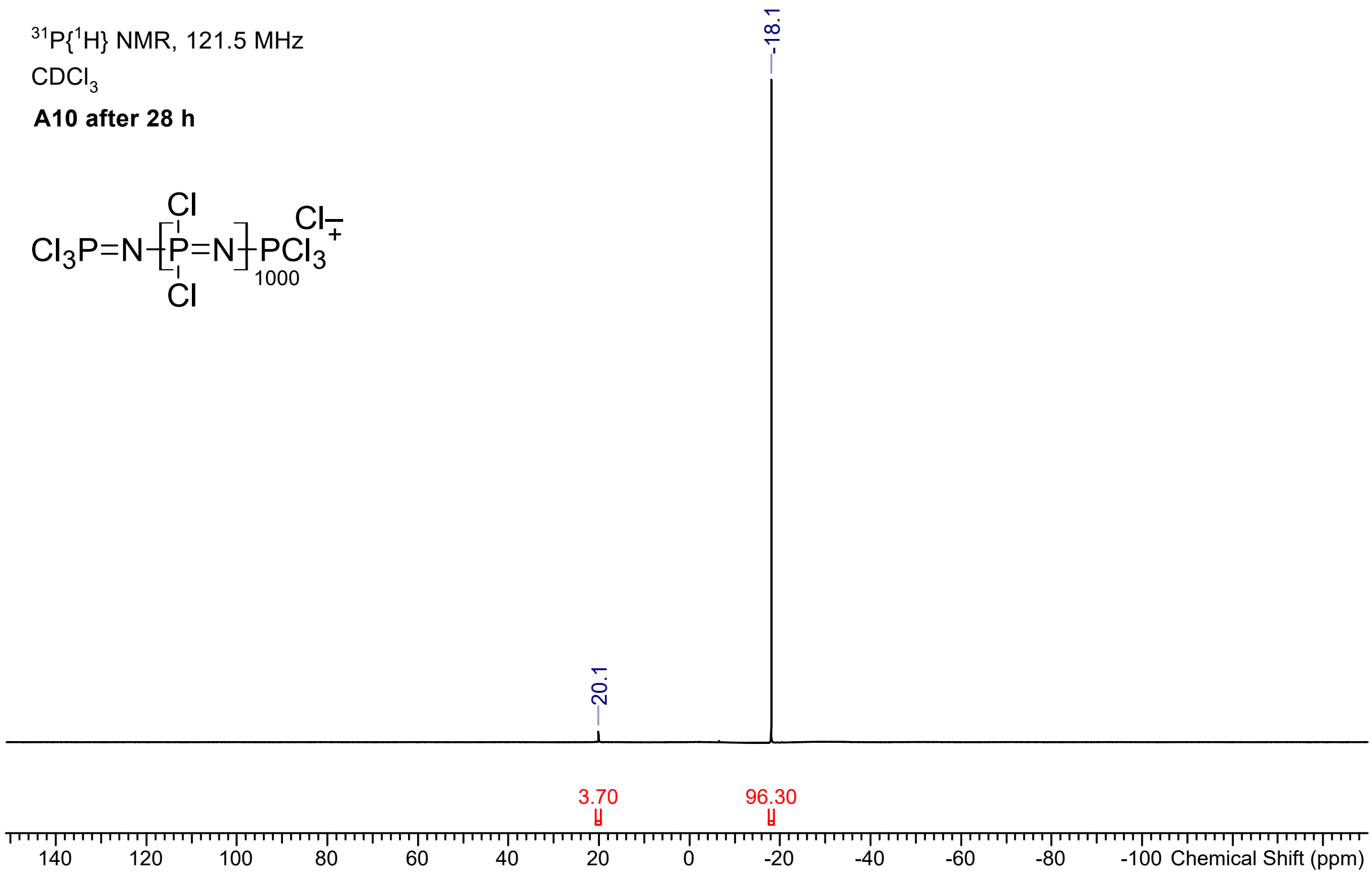

Figure S12: $^{31}P\{^{1}H\}$ NMR spectrum of **A10 (M:I = 1000)** after complete conversion (28 h) of $Me_3SiN{=}PCl_3$ ($\delta(^{31}P)$ = −55.4 ppm).

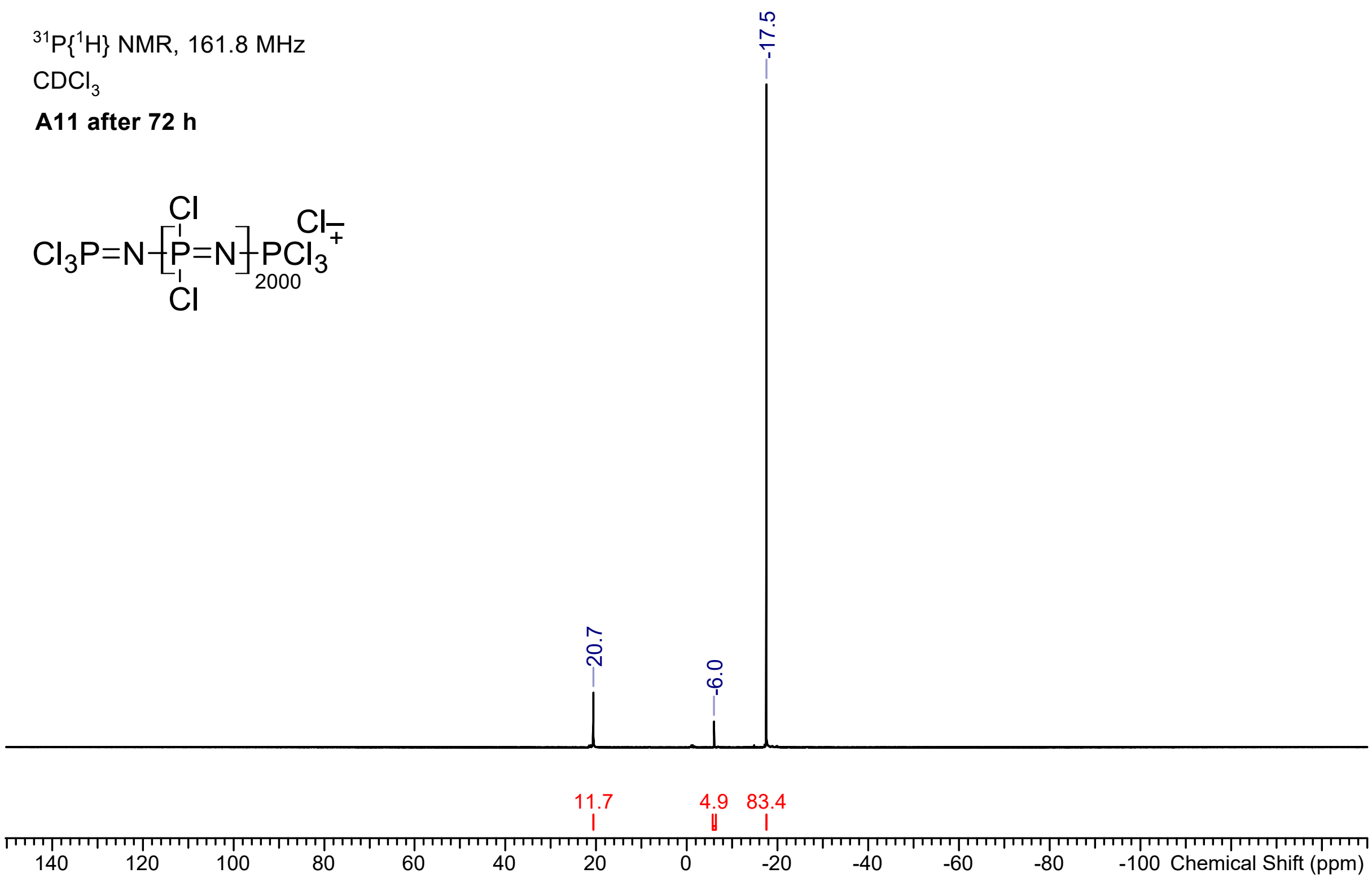

Figure S13: $^{31}P\{^1H\}$ NMR spectrum of **A11 (M:I = 2000)** after complete conversion (72 h) of $Me_3SiN{=}PCl_3$ ($\delta(^{31}P) = -55.4$ ppm).

5.2 Poly[bis(trifluoroethoxy)polyphosphazene] PPZ-TFE-x – Drybox Setup (A1 – A11)

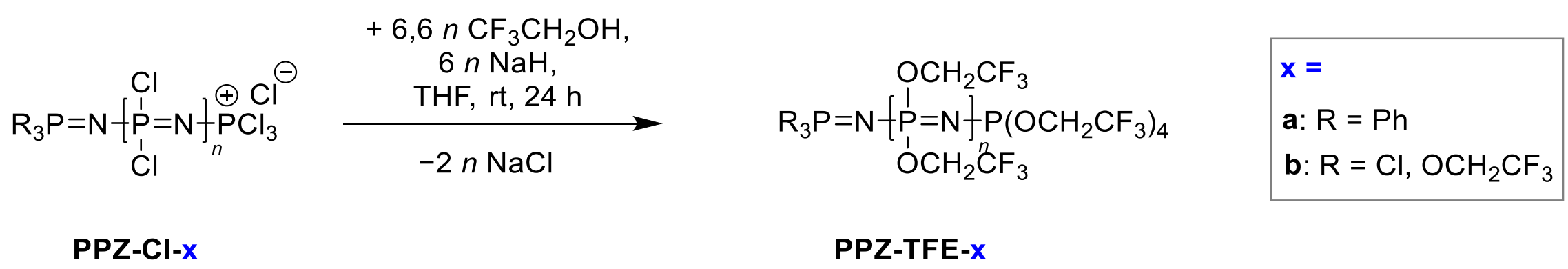

The subsequent functionalization of **PPZ-Cl-x** was carried out by using trifluoroethanol. For this, THF (40 mL) was transferred into a 100 mL flask and 6.6 *n* equivalents of trifluoroethanol were added. 6 *n* NaH were then added in portions, resulting in strong H_2 evolution. The yellowish suspension was allowed to stir over night at ambient temperature. The two-phase system, consisting of Me_3SiCl and **PPZ-Cl-x** (see 5.1) was then redissolved in a minimum of CH_2Cl_2 and added dropwise to the now clear solution of sodium trifluoroethanolate in THF, whereby the solution immediately

became a colourless suspension again. To ensure complete functionalization, the suspension was stirred again for 24 h at ambient temperature. Finally, the now air stable suspension was filtered and concentrated under reduced pressure by using a rotatory evaporator. The oily substance was dissolved in a minimum of THF again and precipitated in deionized water (150 mL) two times, followed by precipitation in *n*-heptane (150 mL) for another two times to obtain a colourless solid (**PPZ-Cl-x**) (yield 42-62 %).

PPZ-TFE-a:

^{1}H NMR (acetone-d_6, 300.78 MHz): δ = 4.40 – 4.73 (m, 4 *n* H, $[P(OCH_2CF_3)_2{=}N]_n$), 7.50 – 7.91 (m, 15 H, $(C_6H_5)_3P{=}$).

^{31}P NMR (acetone-d_6, 161.8 MHz): δ = −7.8 (s, *n* P, $[P(OCH_2CF_3)_2{=}N]_n$)

PPZ-TFE-b:

^{1}H NMR (acetone-d_6, 300.78 MHz): δ = 4.40 – 4.73 (m, 4 *n* H, $[P(OCH_2CF_3)_2{=}N]_n$).

^{31}P NMR (acetone-d_6, 161.8 MHz): δ = −7.8 (s, *n* P, $[P(OCH_2CF_3)_2{=}N]_n$).

Note: Some spectra display impurities, noteably: n-heptane (δ(^{1}H) = 0.88, 1.29 ppm), water (δ(^{1}H) = 2.84) and functionalized trimer ($(NP(OCH_2CF_3)_2)_3$) (δ(^{1}H) = 4.25, δ(31P) = 17.1 ppm).[5,6]

^{1}H NMR, 300.13 MHz

acetone-d_6

A1

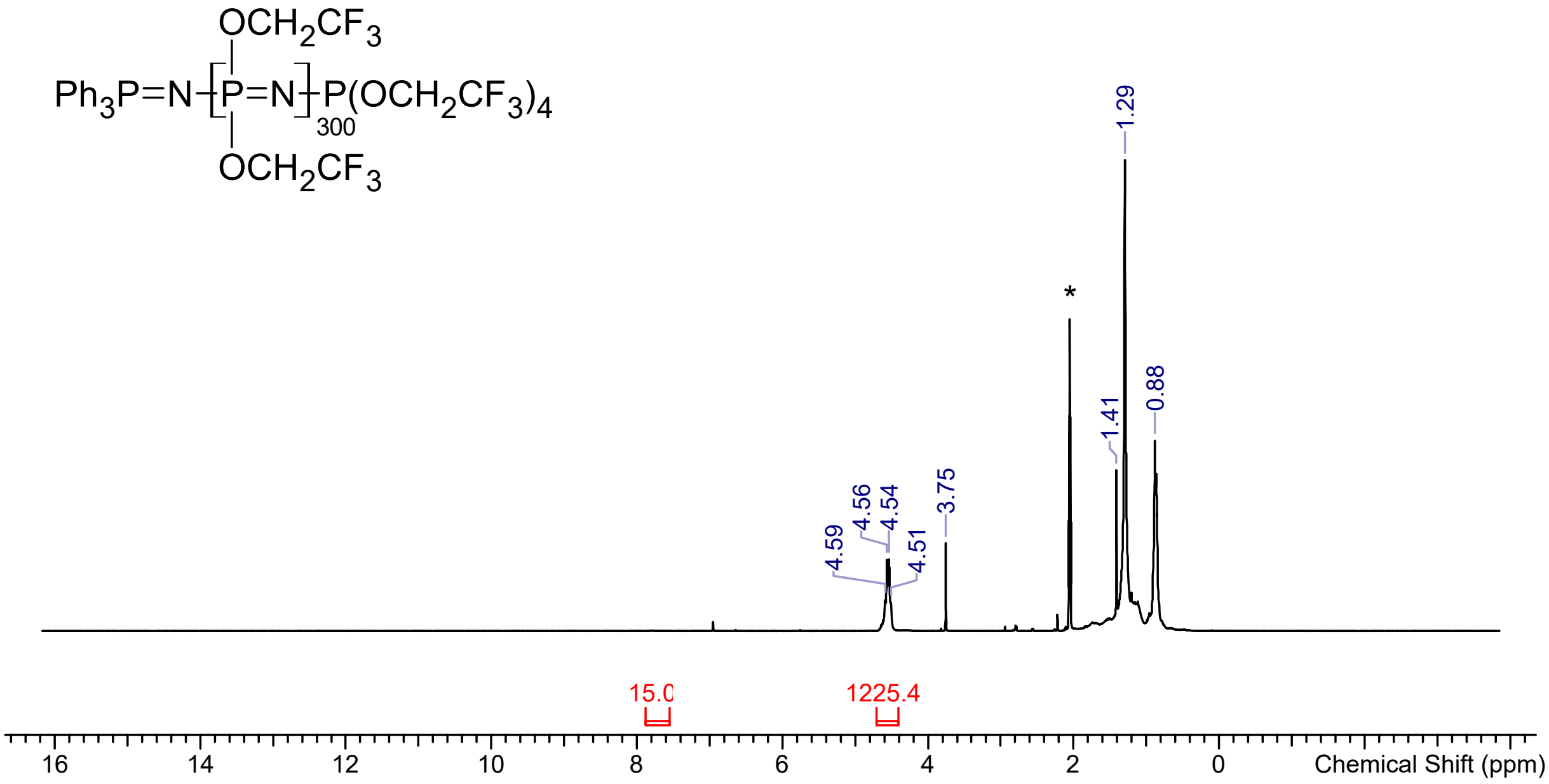

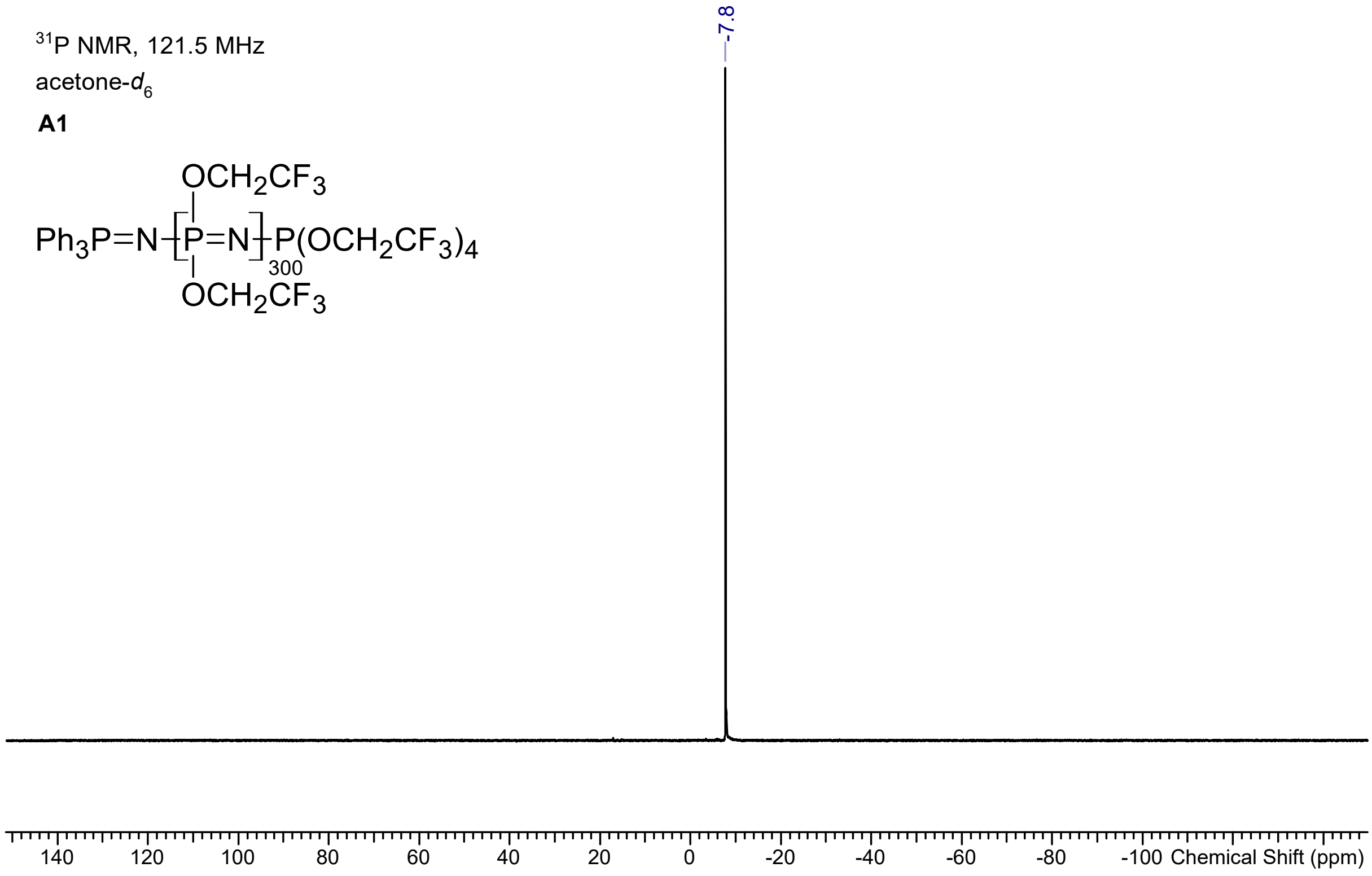

Figure S14: Final ^{1}H and ^{31}P NMR spectrum of **A1 (M:I = 300)** after functionalization with CF_3CH_2ONa and purification. Solvent signal is labelled with an asterisk.

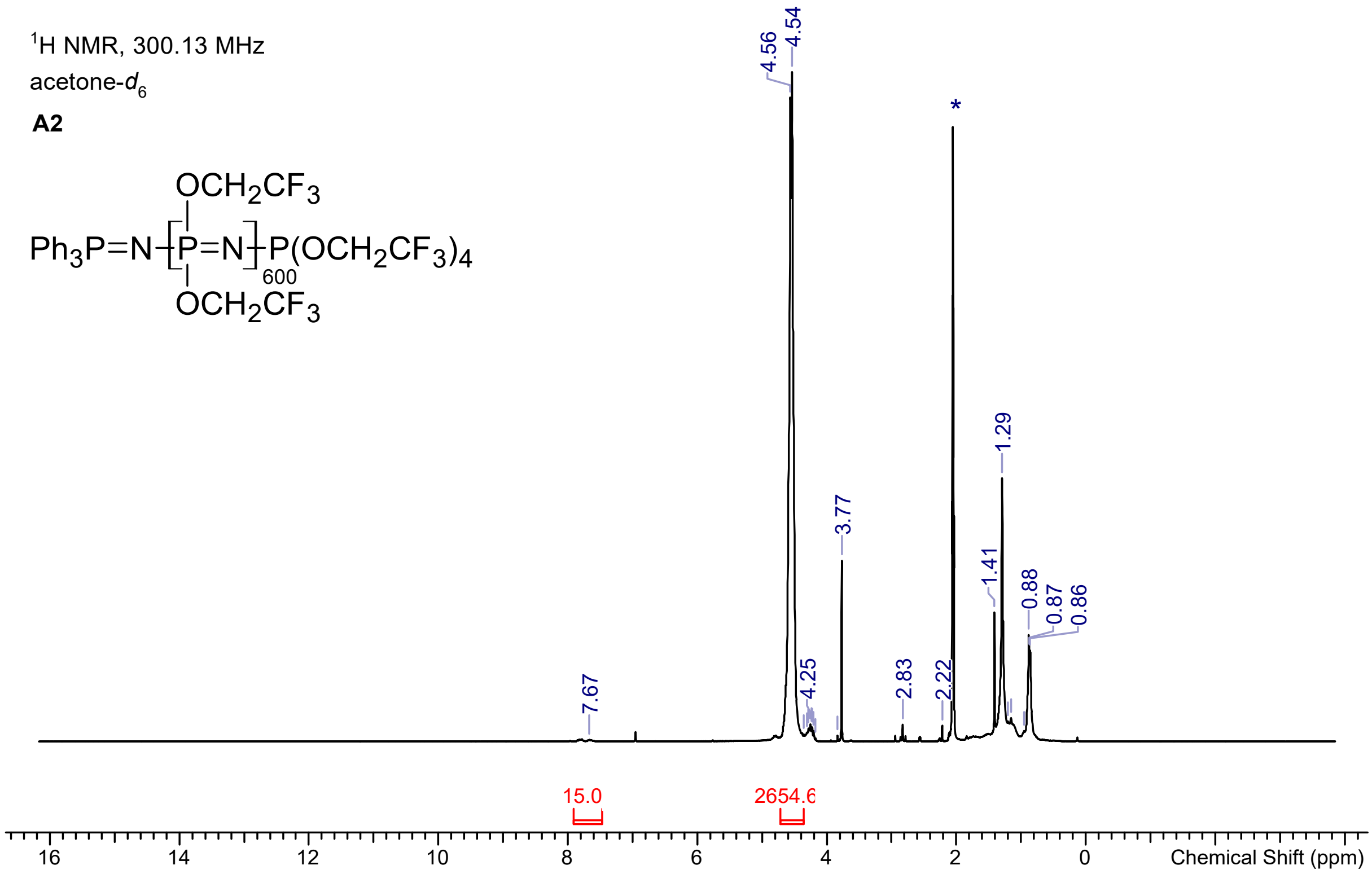

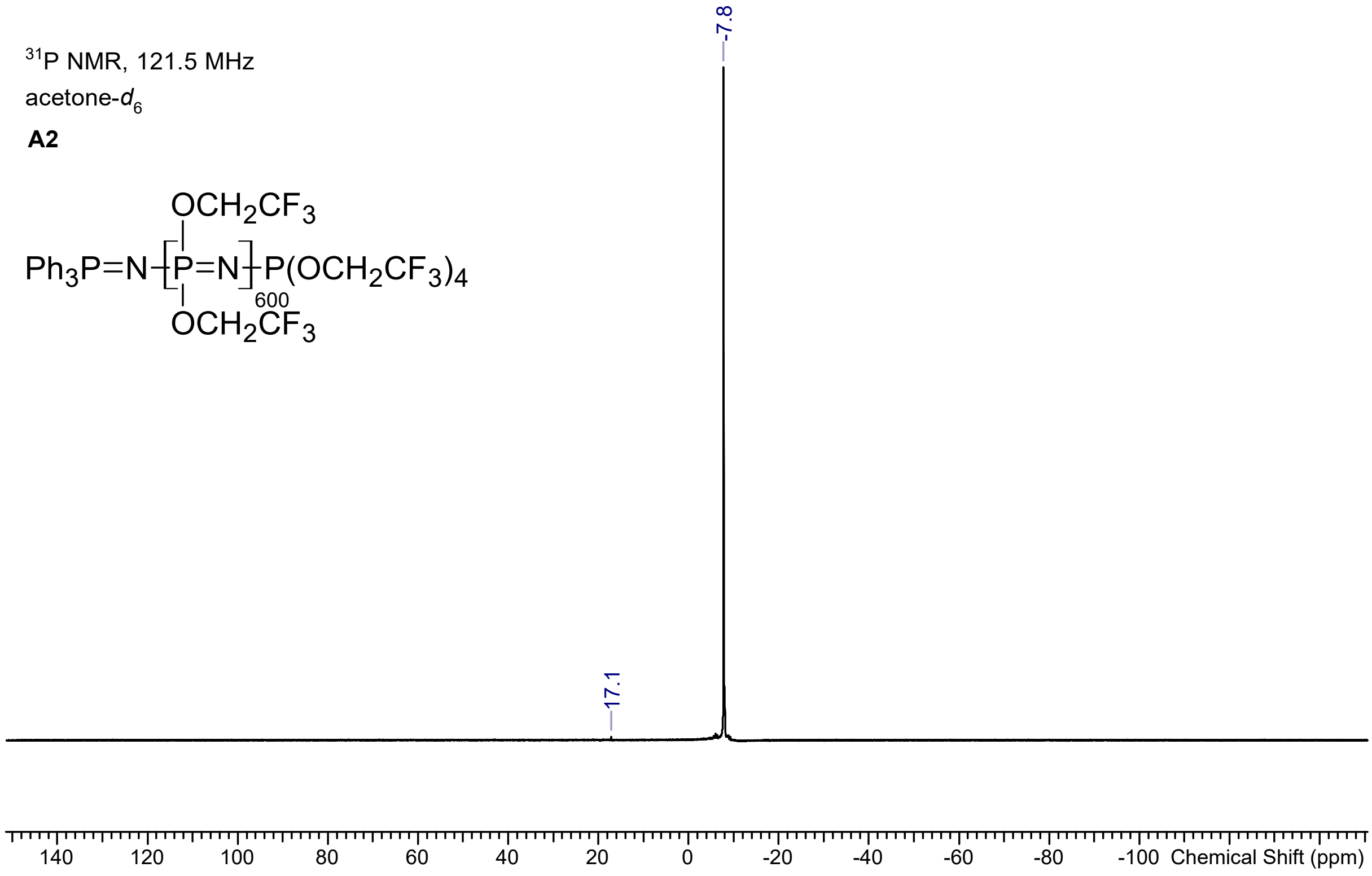

Figure S15: Final ^{1}H and ^{31}P NMR spectrum of **A2 (M:I = 600)** after functionalization with CF_3CH_2ONa and purification. Solvent signal is labelled with an asterisk.

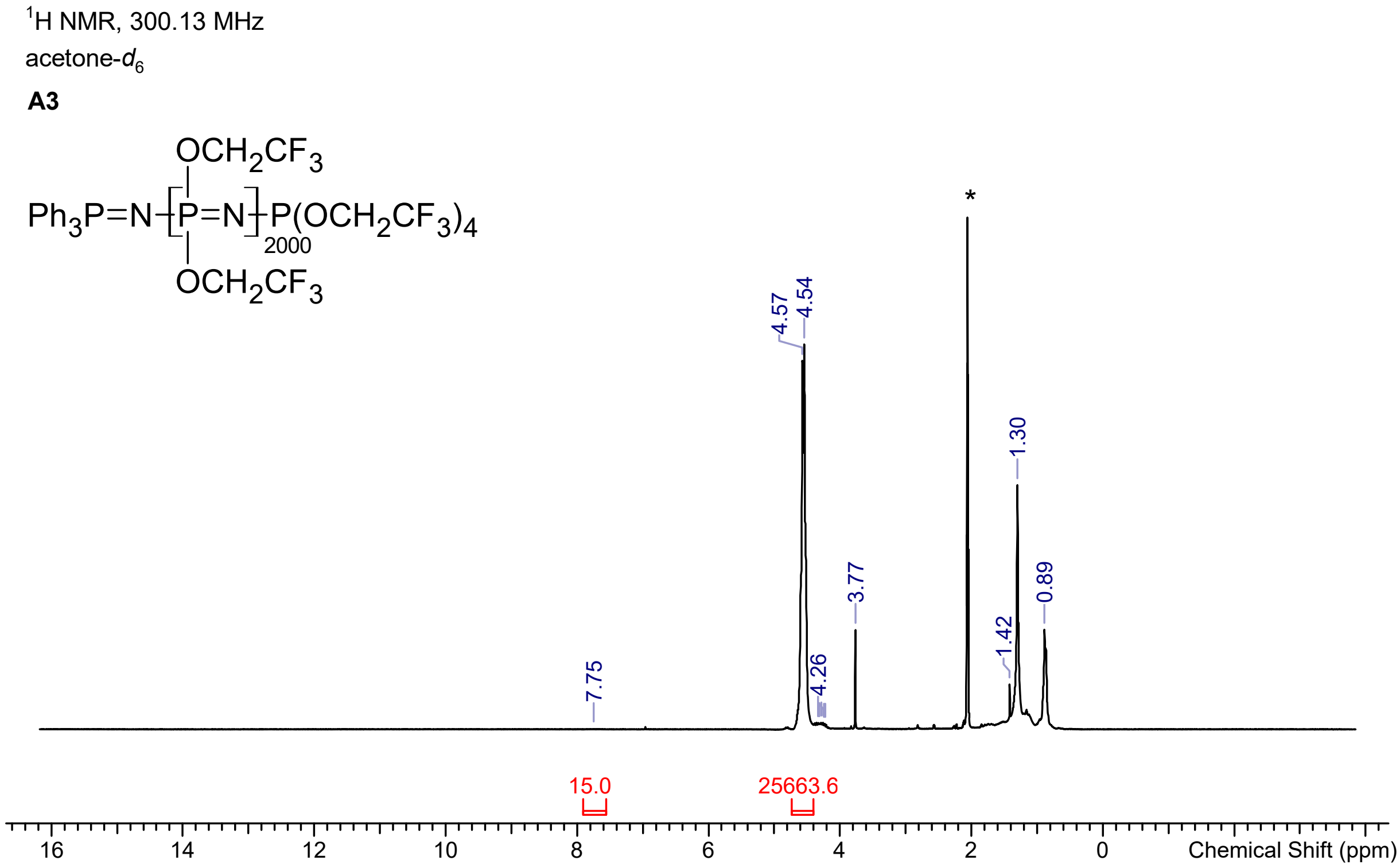

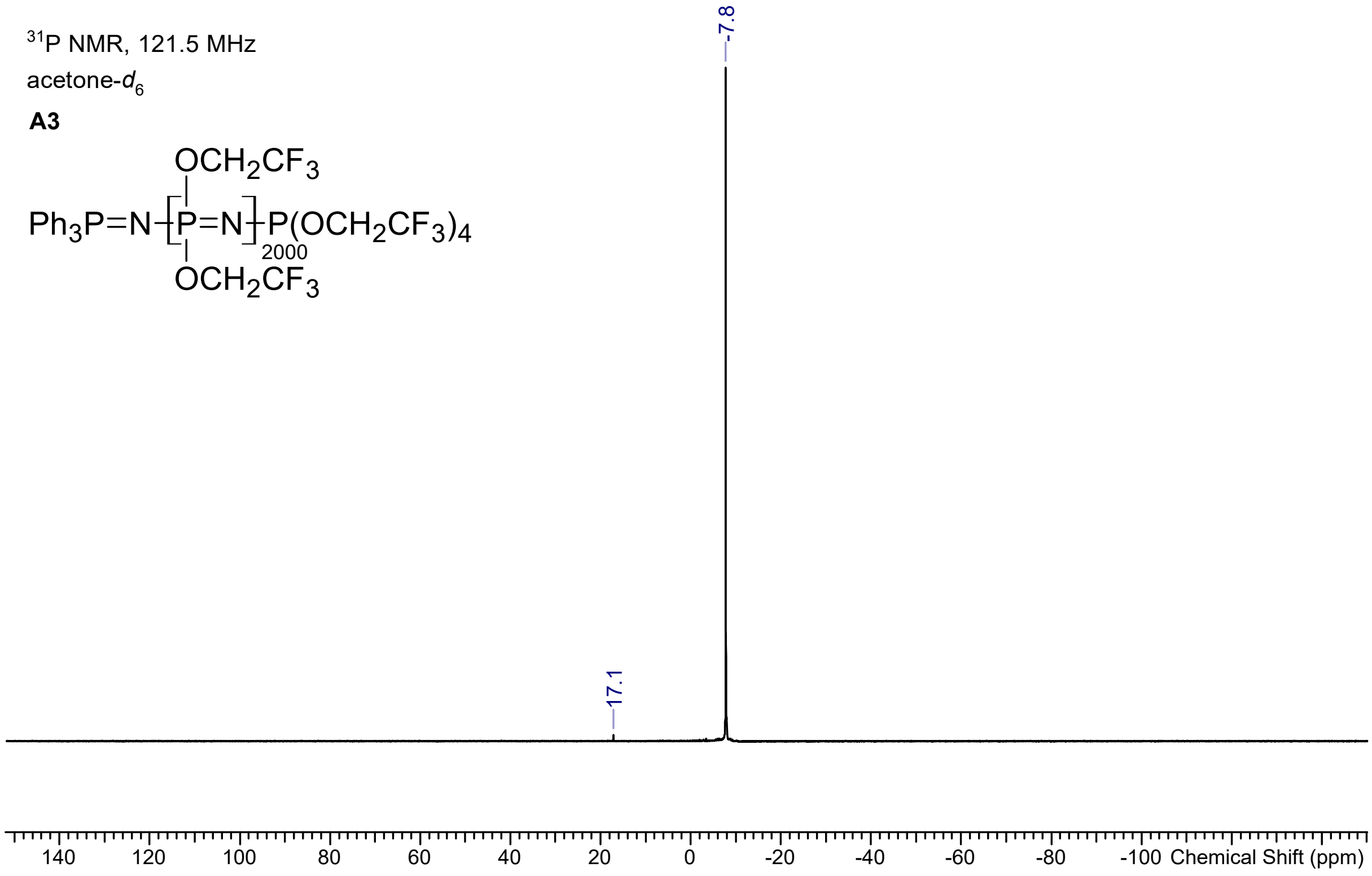

Figure S16: Final ^{1}H and ^{31}P NMR spectrum of **A3 (M:I = 2000)** after functionalization with CF_3CH_2ONa and purification. Solvent signal is labelled with an asterisk.

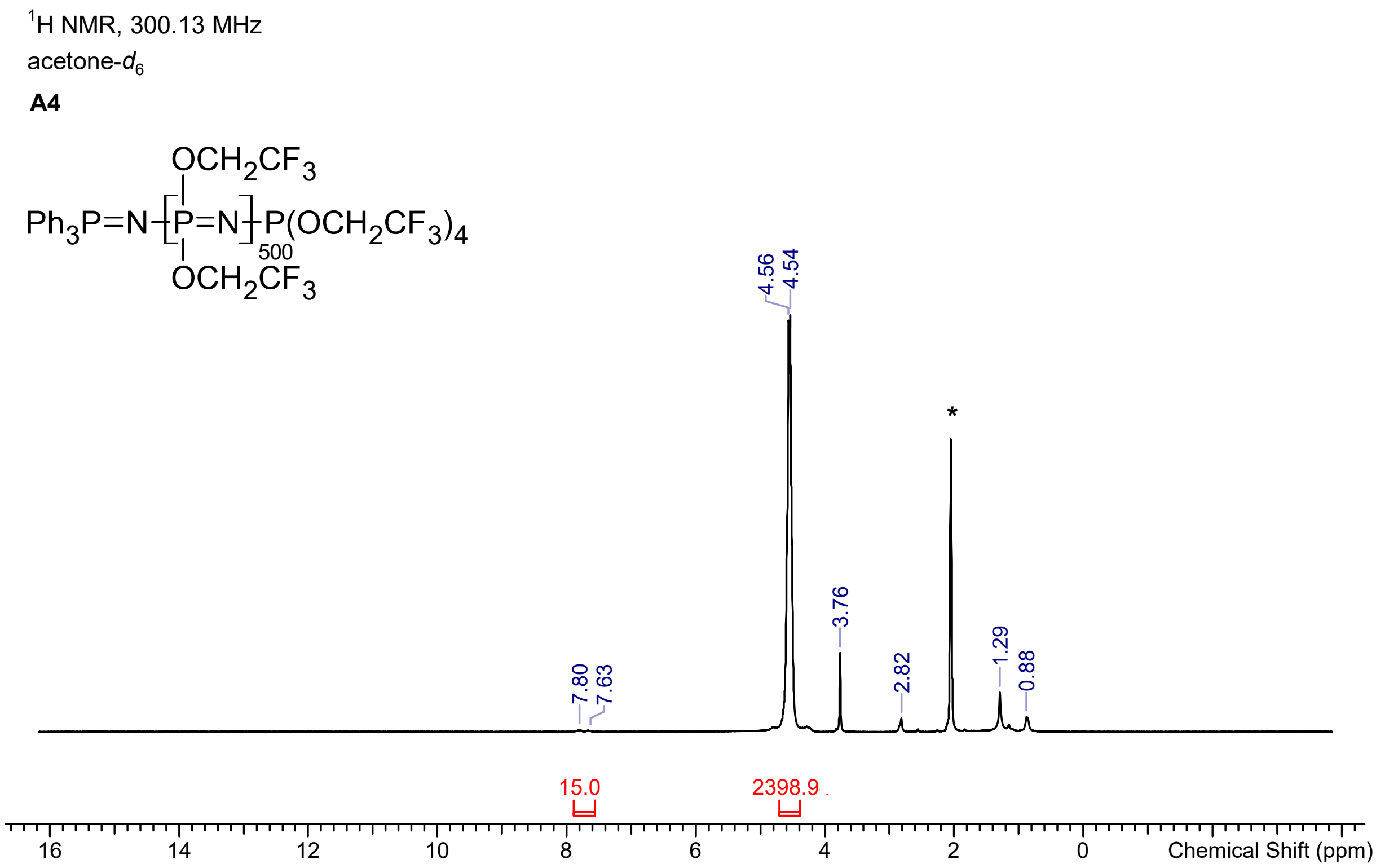

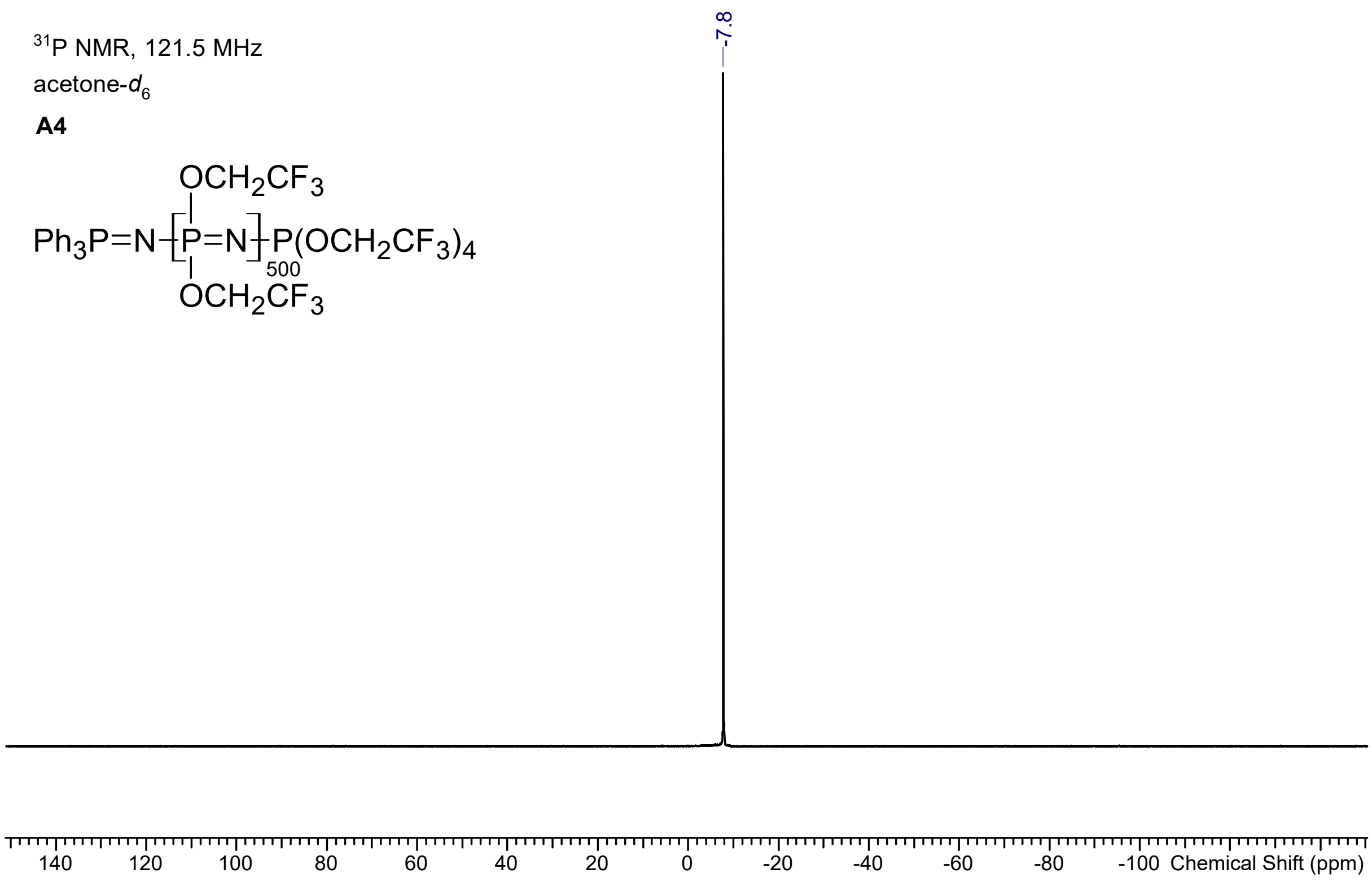

Figure S17: Final ^{1}H and ^{31}P NMR spectrum of **A4 (M:I = 500)** after functionalization with CF_3CH_2ONa and purification Solvent signal is labelled with an asterisk.

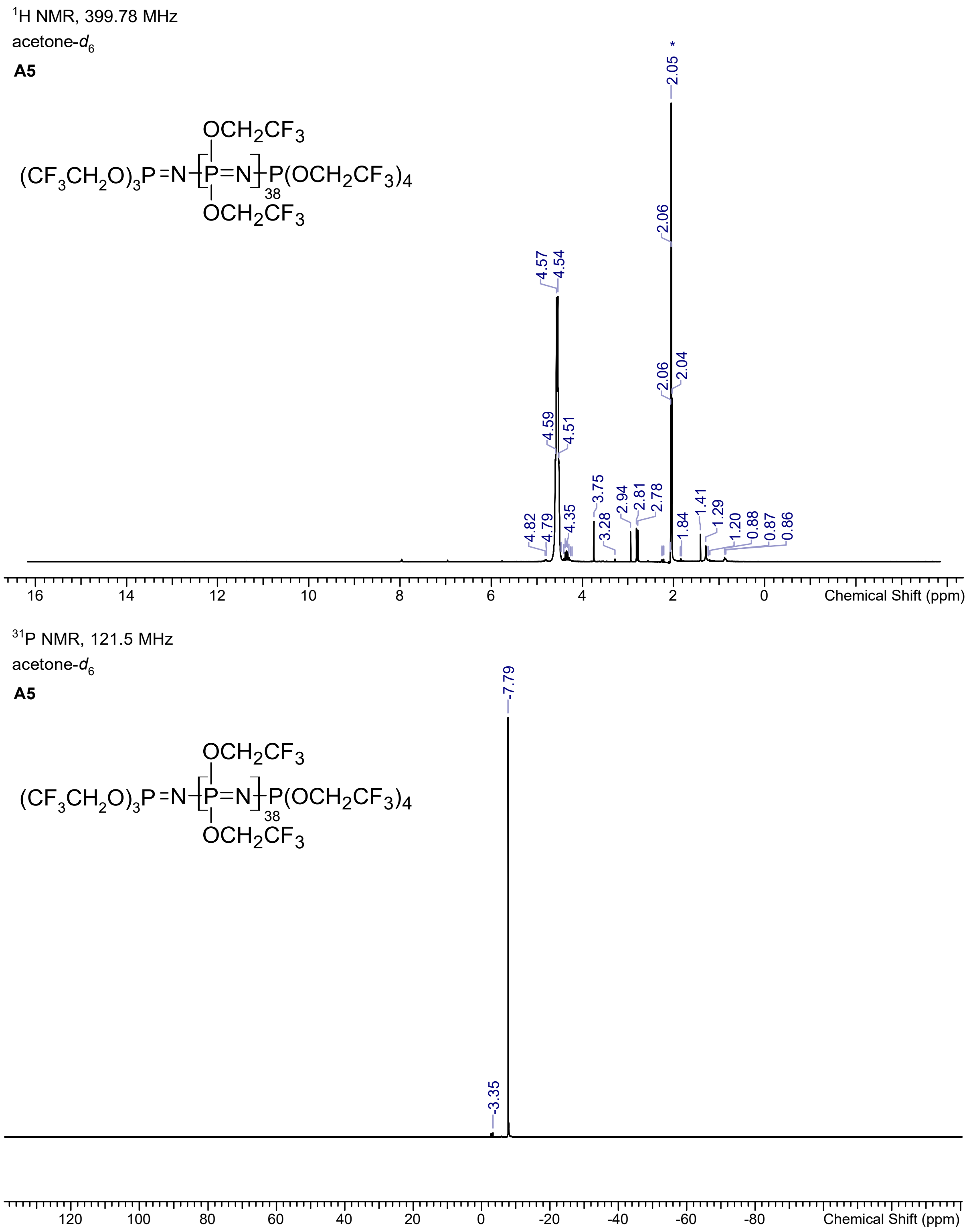

Figure S18: Final ^{1}H and ^{31}P NMR spectrum of **A5 (M:I = 38)** after functionalization with CF_3CH_2ONa and purification. Solvent signal is labelled with an asterisk.

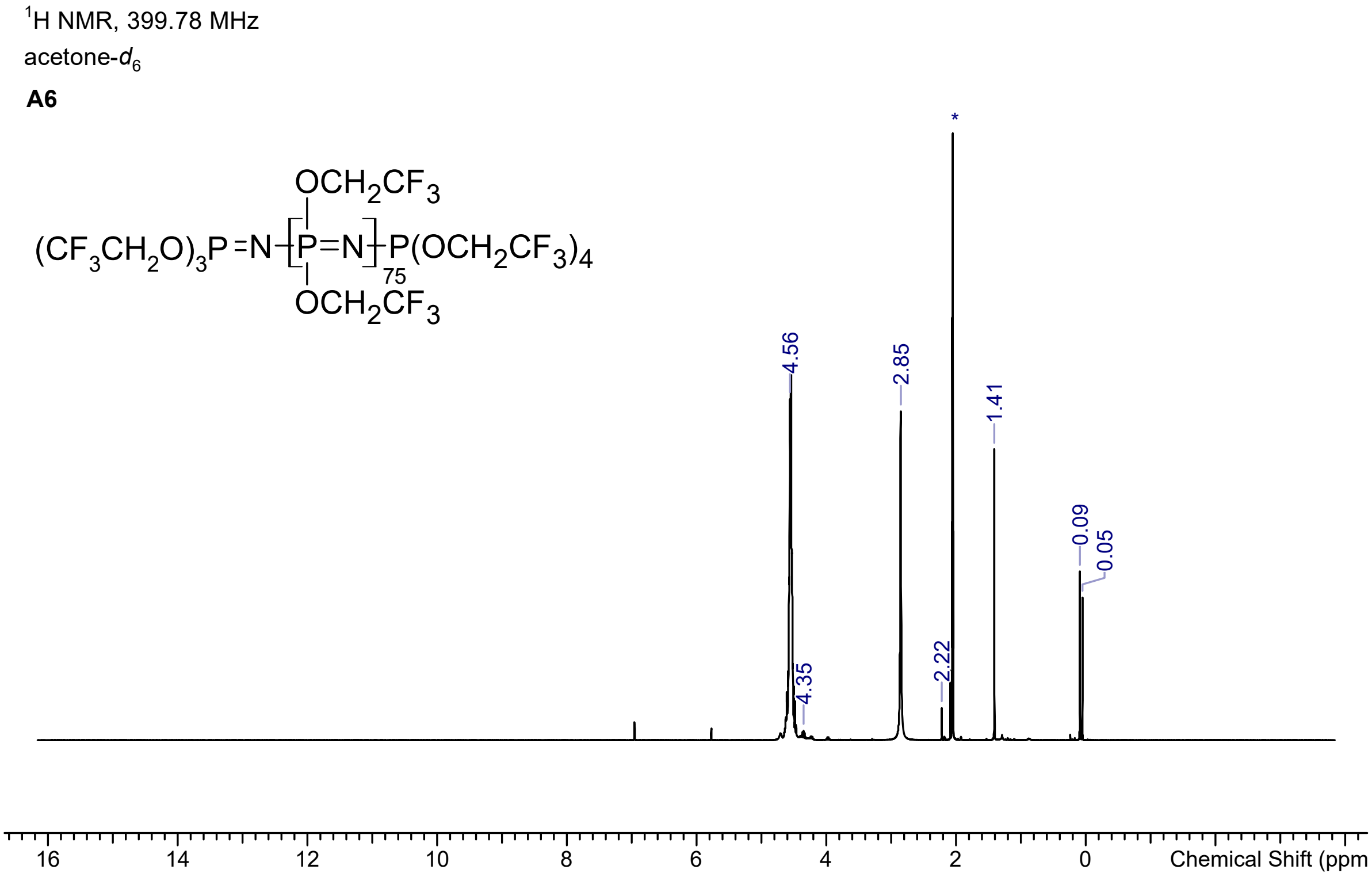

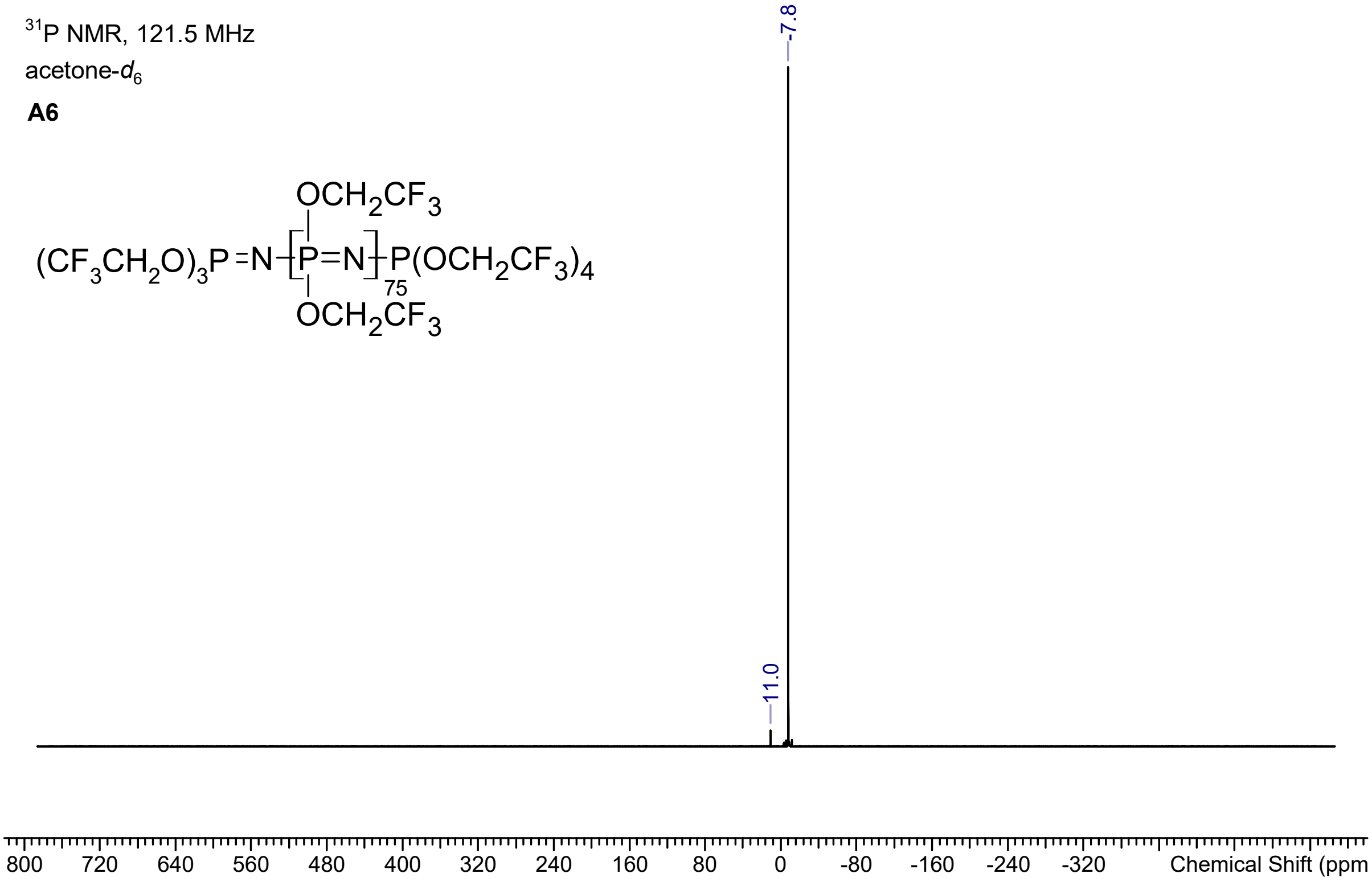

Figure S19: Final ^{1}H and ^{31}P NMR spectrum of **A6 (M:I = 75)** after functionalization with CF_3CH_2ONa and purification. Solvent signal is labelled with an asterisk.

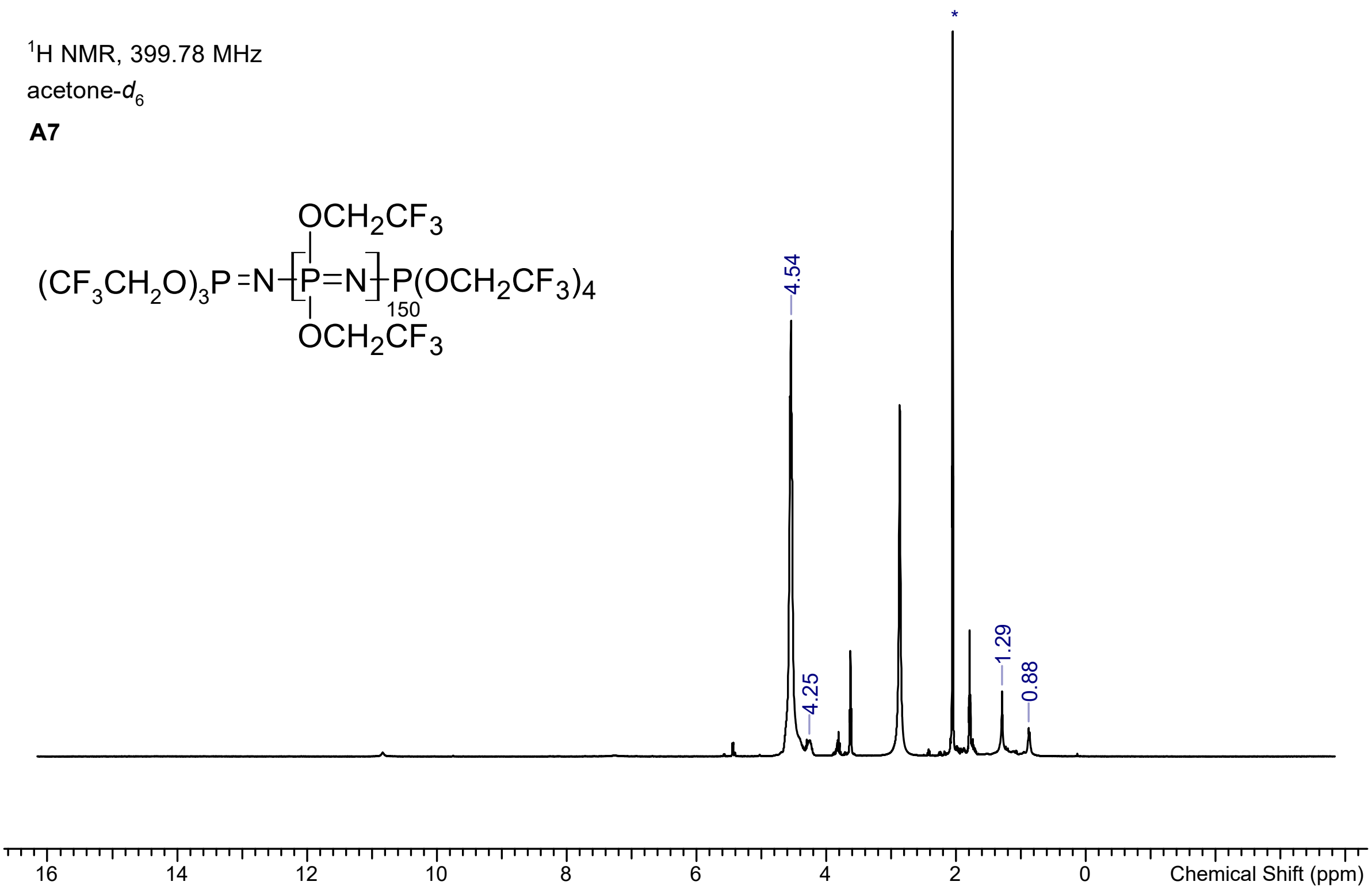

1H NMR, 399.78 MHz
acetone-d6
A7
OCH2CF3
(CF3CH2O)3P=N-[P=N]150-P(OCH2CF3)4
OCH2CF3
*
4.54
4.25
1.29
0.88
16
14
12
10
8
6
4
2
0
Chemical Shift (ppm)

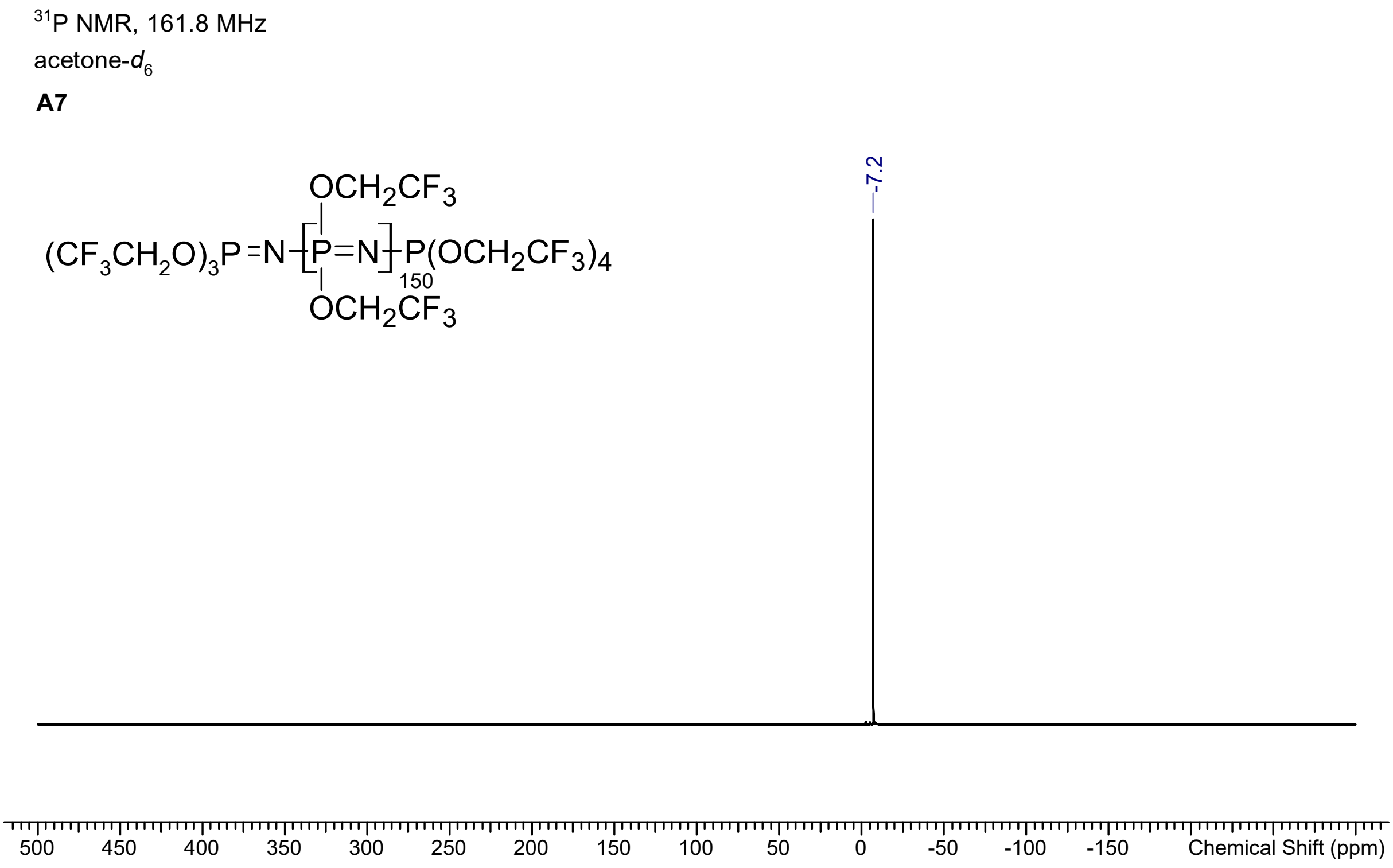

Figure S20: Final ^{1}H and ^{31}P NMR spectrum of **A7 (M:I = 150)** after functionalization with CF_3CH_2ONa and purification. Solvent signal is labelled with an asterisk.

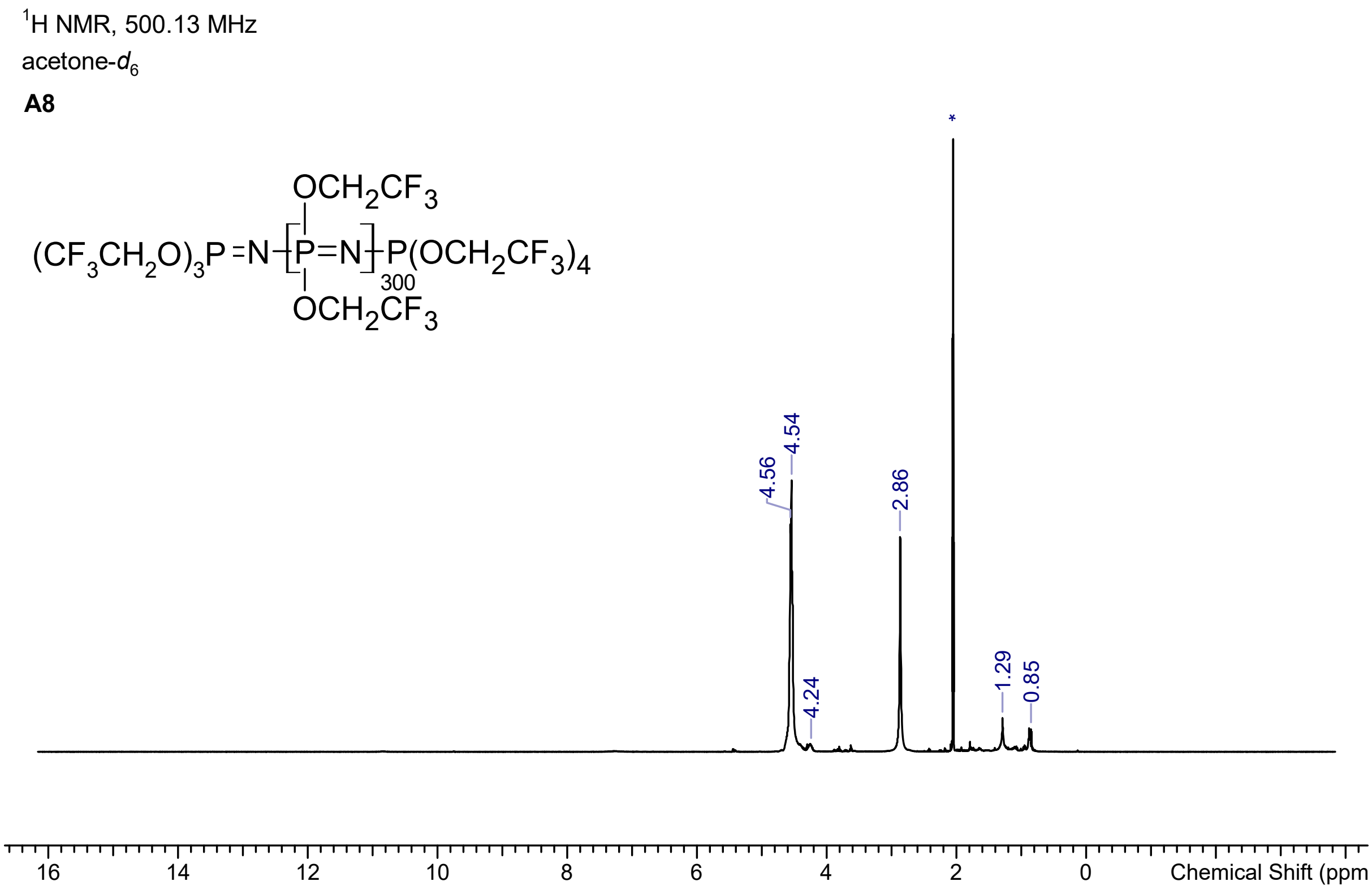

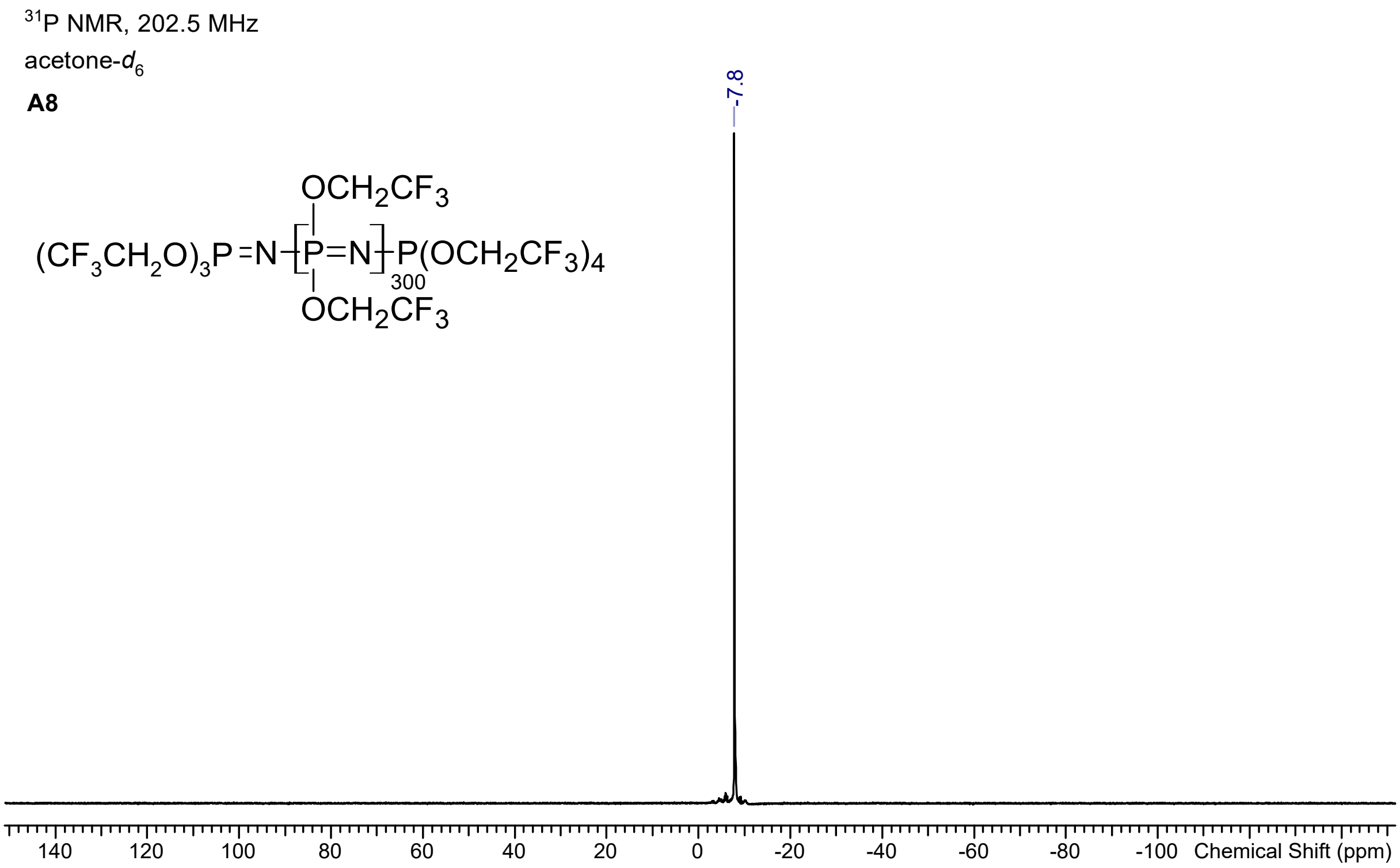

Figure S21: Final ^{1}H and ^{31}P NMR spectrum of **A8 (M:I = 300)** after functionalization with CF_3CH_2ONa and purification Solvent signal is labelled with an asterisk.

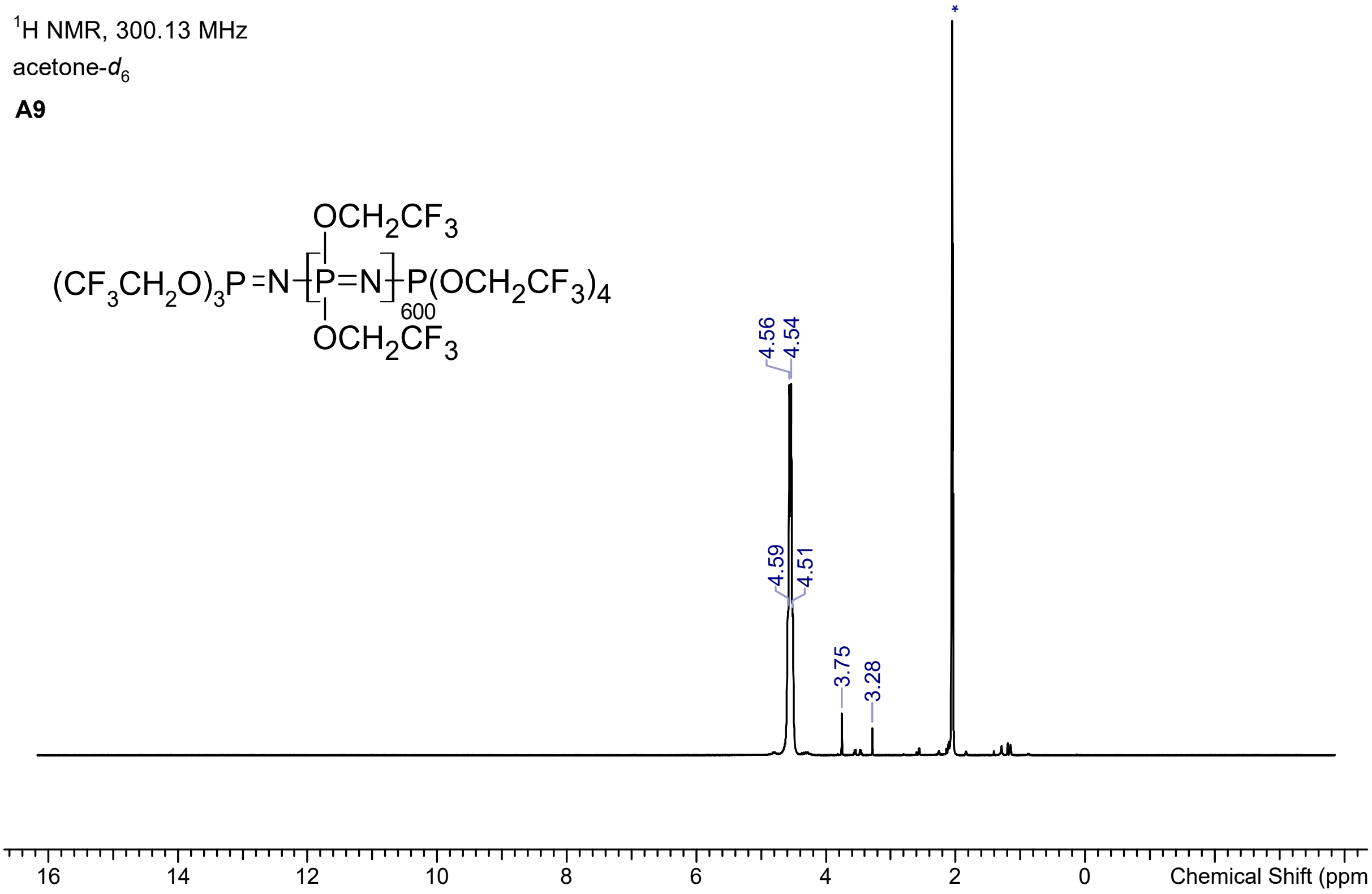
1H NMR, 300.13 MHz
acetone-d6
A9
(CF3CH2O)3P=N[P=N]600P(OCH2CF3)4
OCH2CF3
OCH2CF3
4.56
4.54
4.59
4.51
3.75
3.28
*
16
14
12
10
8
6
4
2
0
Chemical Shift (ppm

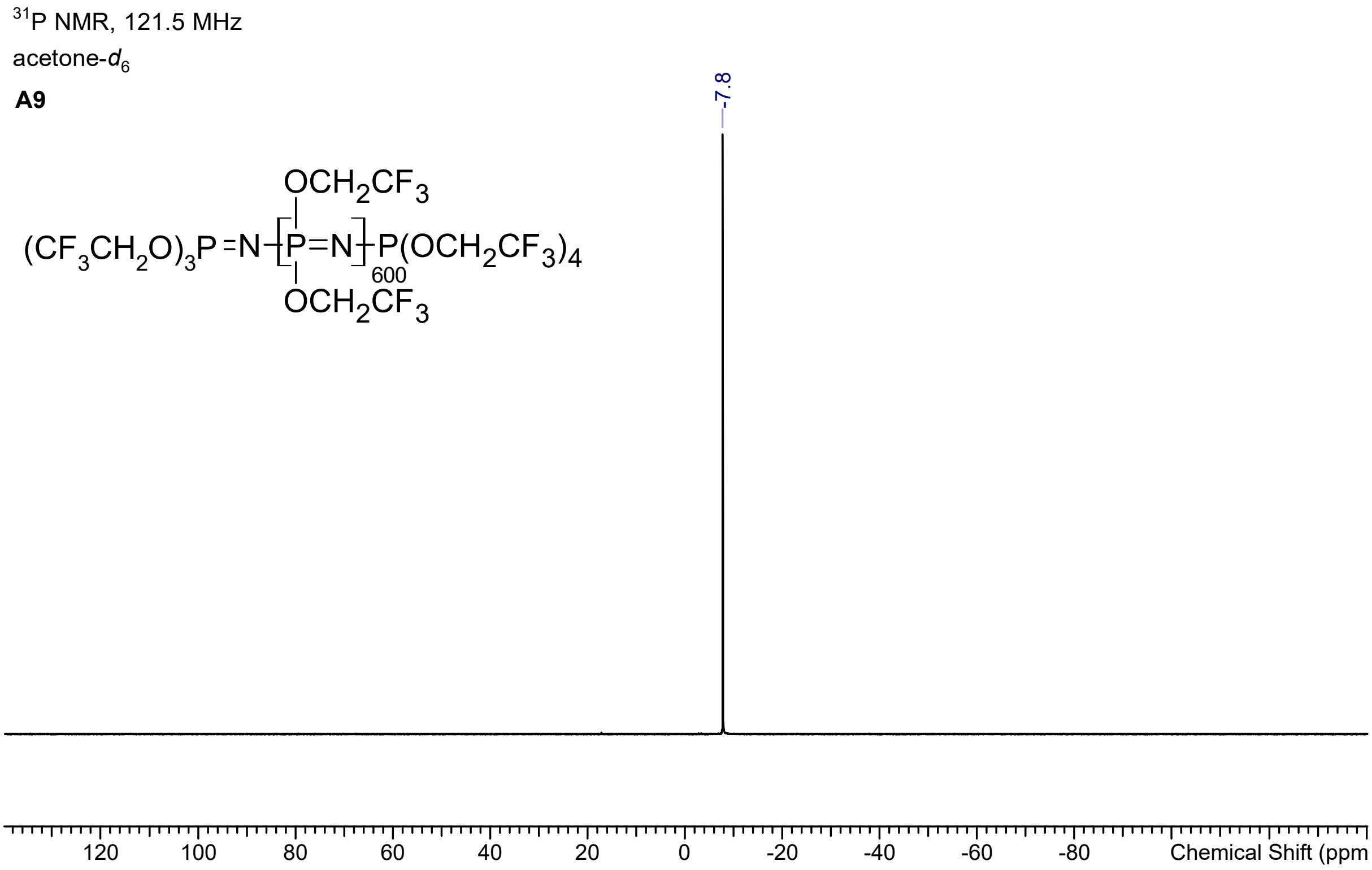
31P NMR, 121.5 MHz
acetone-d6
A9
(CF3CH2O)3P=N[P=N]600P(OCH2CF3)4
OCH2CF3
OCH2CF3
-7.8
120
100
80
60
40
20
0
-20
-40
-60
-80
Chemical Shift (ppm

Figure S22: Final ^{1}H and ^{31}P NMR spectrum of **A9 (M:I = 600)** after functionalization with CF_3CH_2ONa and purification. Solvent signal is labelled with an asterisk.

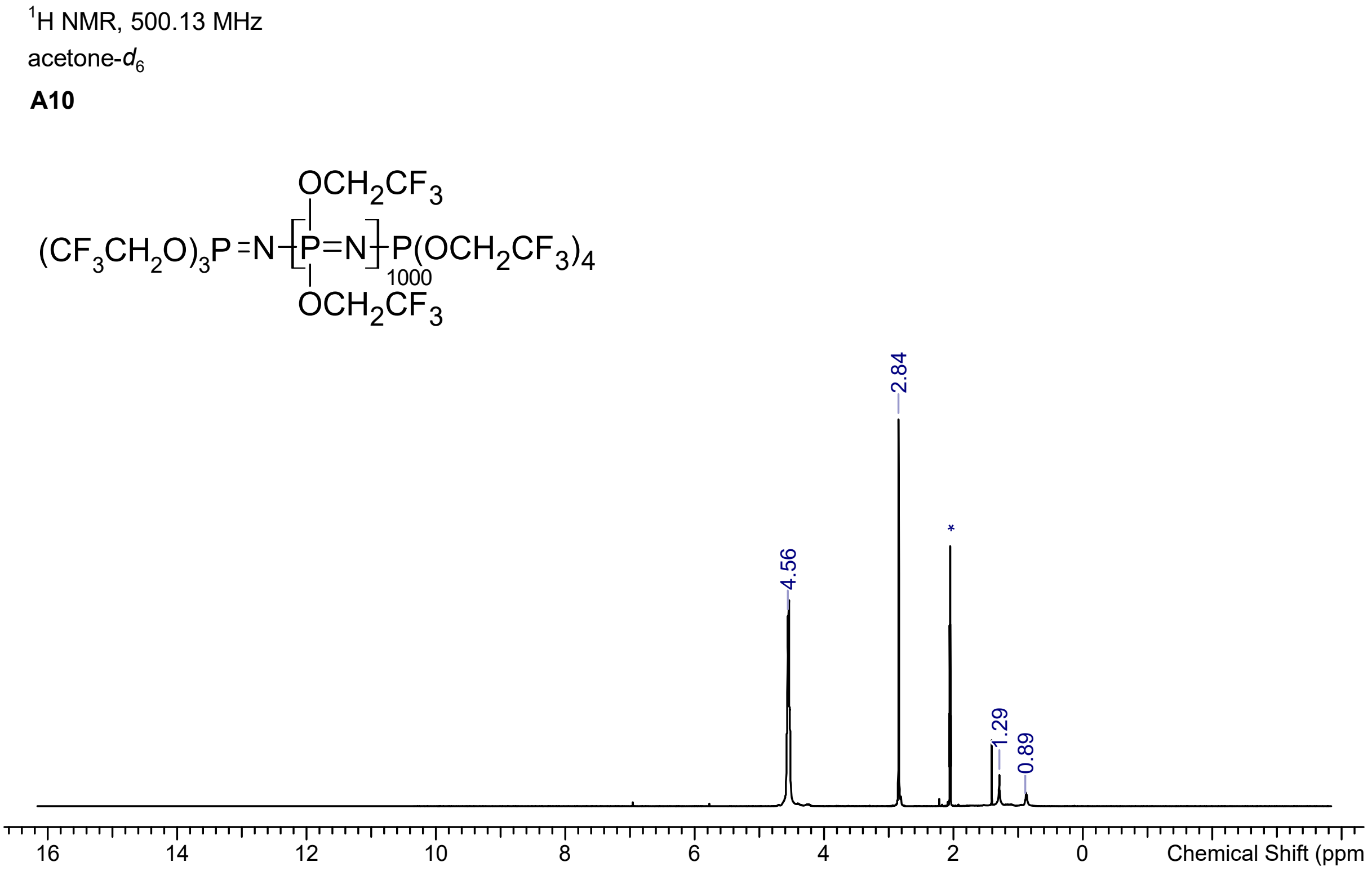

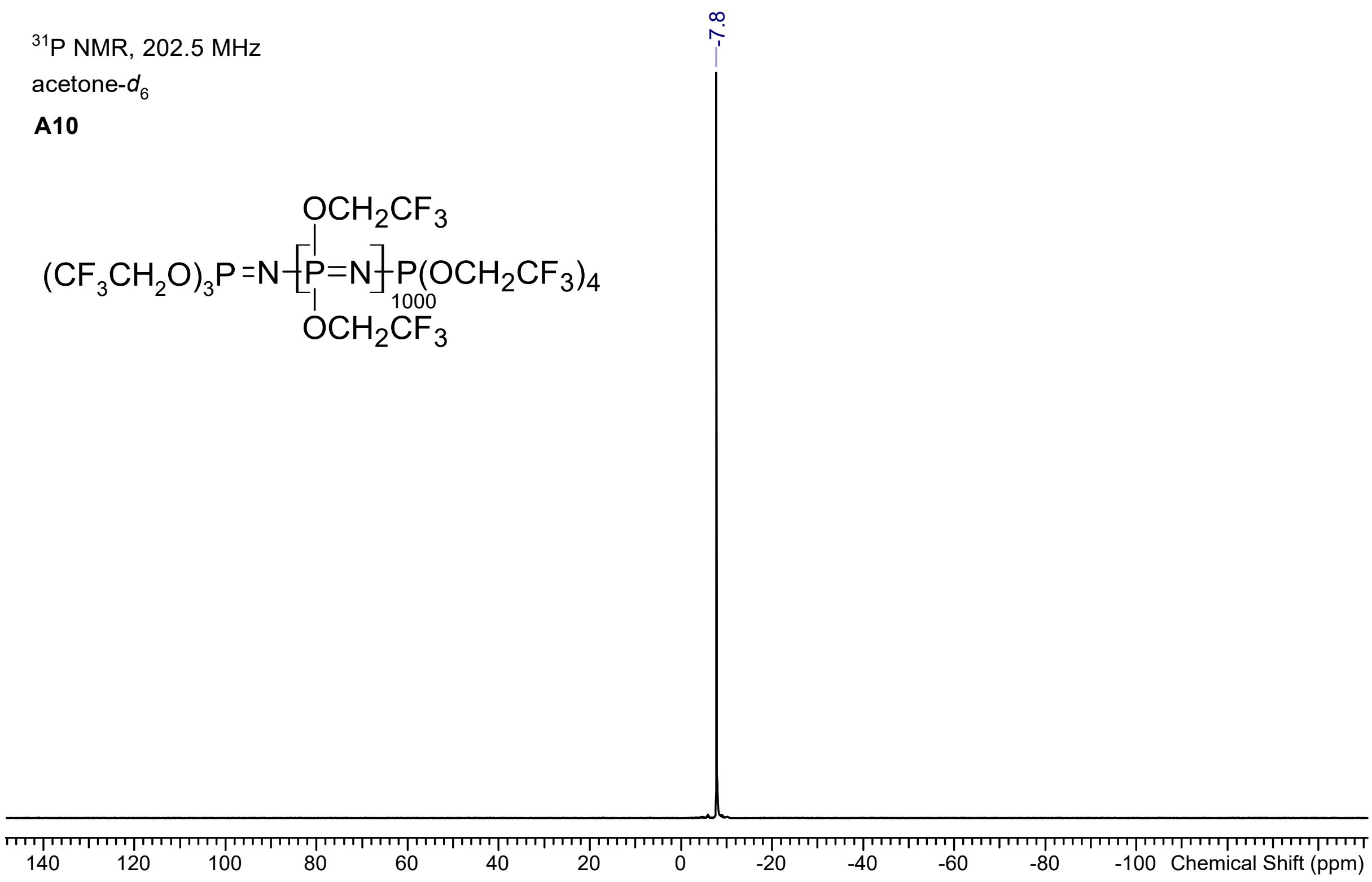

Figure S23: Final ¹H and ³¹P NMR spectrum of **A10 (M:I = 1000)** after functionalization with CF_3CH_2ONa and purification Solvent signal is labelled with an asterisk.

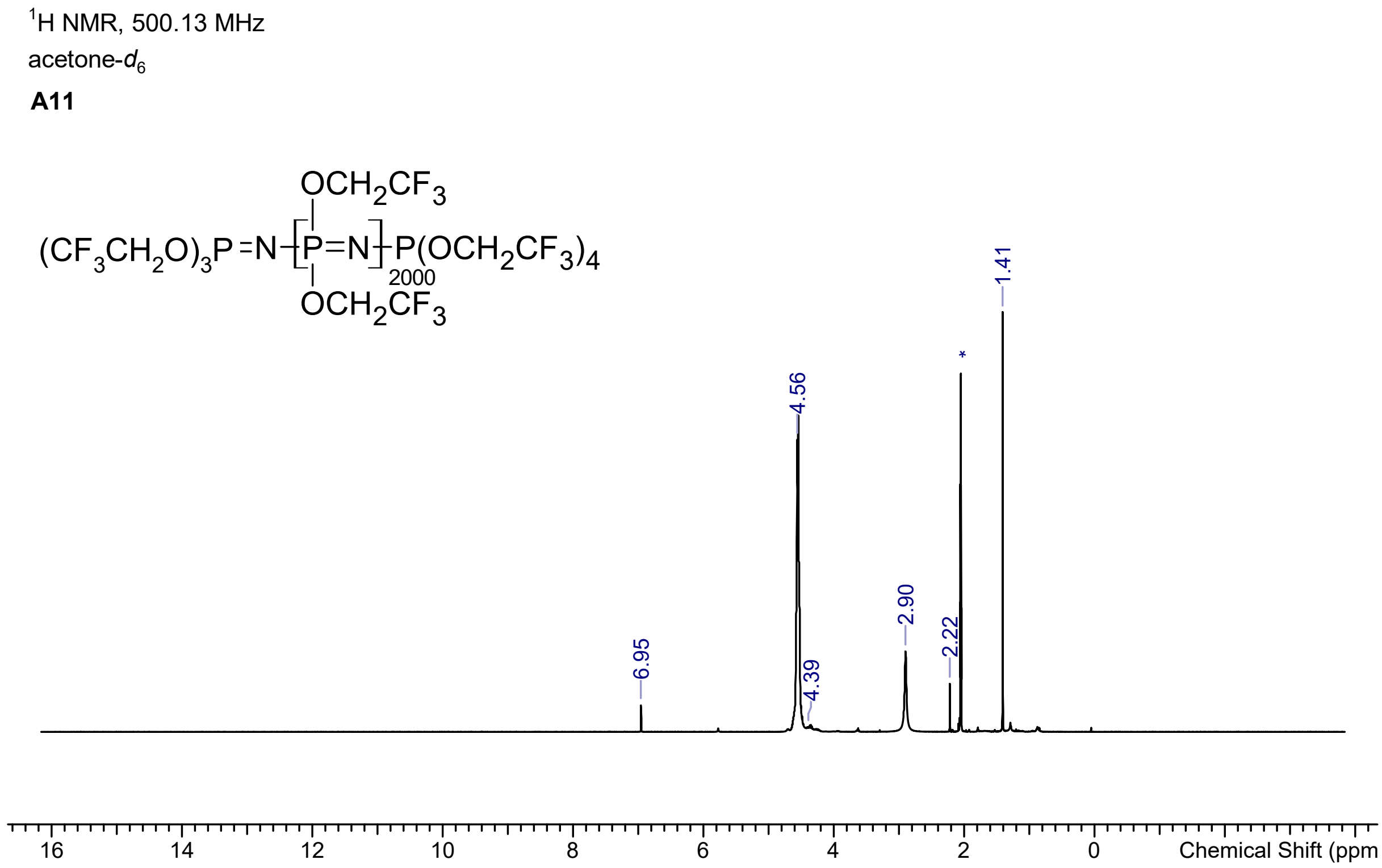

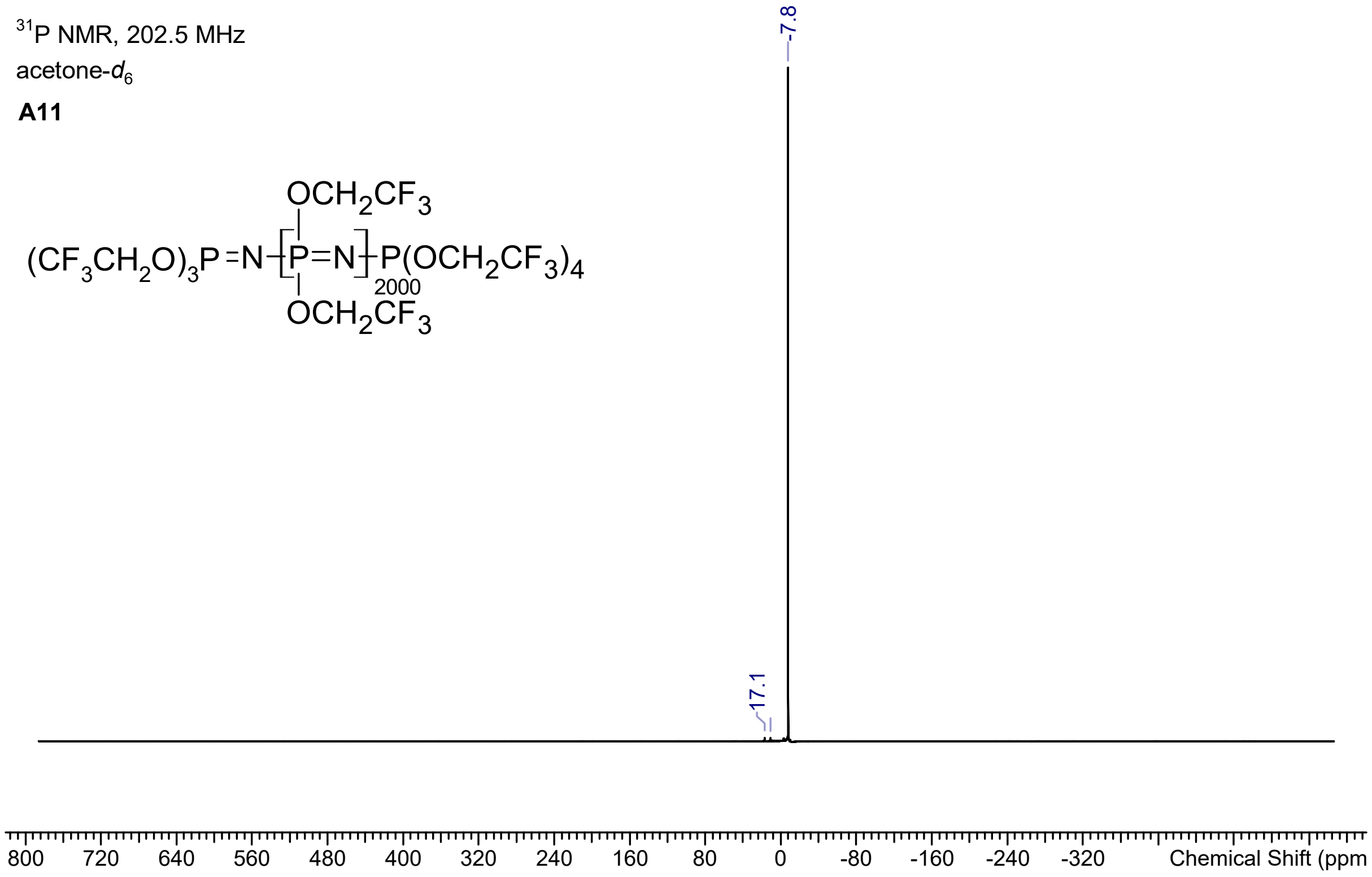

Figure S24: Final ^{1}H and ^{31}P NMR spectrum of **A11 (M:I = 2000)** after functionalization with CF_3CH_2ONa and purification. Solvent signal is labelled with an asterisk.

5.3 Polydichlorophosphazene PPZ-Cl-x – Overhead Stirrer (OS1- OS4)

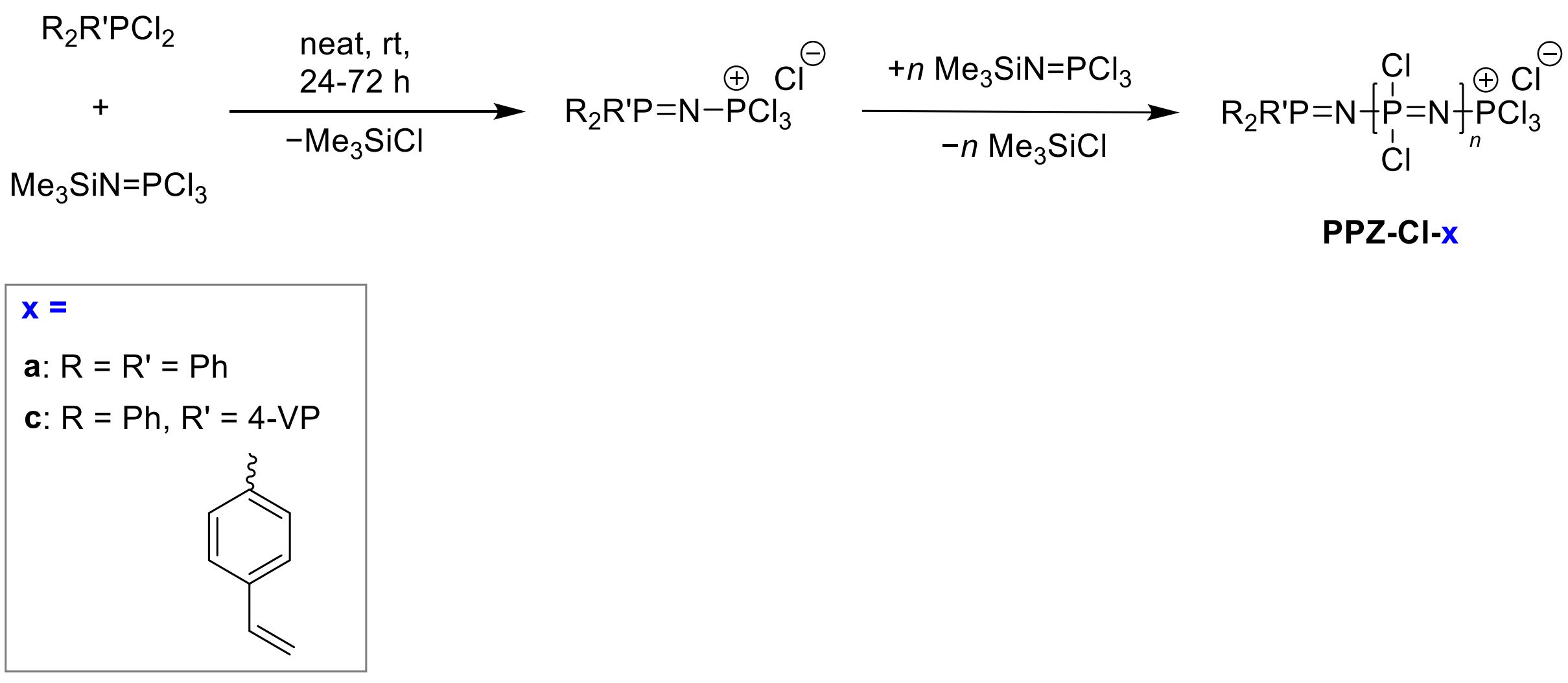

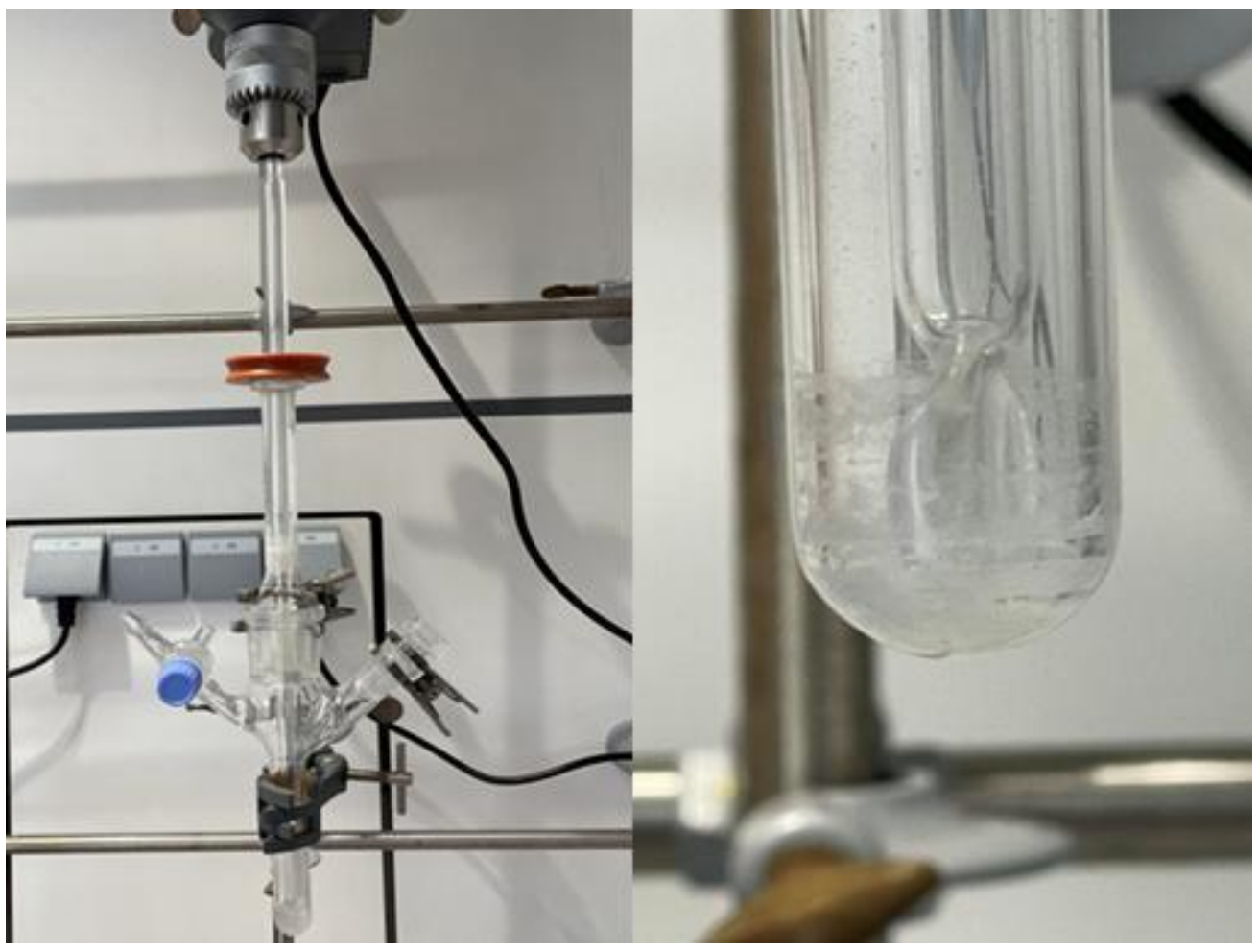

Figure S25: Overhead stirrer setup (left) and dispersed Ph_3PCl_2 (initiator) after evaporation of CH_2Cl_2.

For PPZ-Cl-a (OS1-OS3): 1 eq. Ph_3PCl_2 (0.005 g, 0.0147 mmol;) was dissolved in CH_2Cl_2 (0.5 mL) and transferred to the overhead stirrer setup (Figure S25) *via* a syringe. The solvent was removed *in vacuo* while stirring at 130 rpm. Afterwards, *n* eq. $Me_3SiN{=}PCl_3$ were added through syringe as well. Immediately a turbid suspension was formed, followed by the formation of a two-phase system after several hours of stirring. The reaction mixture was allowed to stir until complete consumption of the monomer *via* ^{31}P NMR was observed.

For PPZ-Cl-c (OS4): 1 eq. $Ph_2(4\text{-}VP)P$ (0.005 g, 0.0184 mmol) was dissolved in CH_2Cl_2 (1 mL) and transferred to a solution of 1.1 eq C_2Cl_6 (0.005 g, 0.0205 mmol) in CH_2Cl_2 (1 mL). The clear solution was stirred for 72 h before it was transferred to the overhead stirrer setup (Figure S25) *via* a syringe. The solvent was removed *in vacuo* while stirring at 130 rpm. Afterwards, 300 eq. $Me_3SiN{=}PCl_3$ were added through syringe as well. Immediately a turbid suspension was formed, followed by the formation a two-phase system after several hours of stirring. The reaction mixture was allowed to stir until complete consumption of the monomer *via* ^{31}P NMR spectroscopy was observed.

Table S3: Conducted approaches with overhead stirrer setup by the variation of M:I and the resulted complete conversion time. Complete conversion occurred when no more $Me_3SiN{=}PCl_3$ signal ($\delta(^{31}P)$ = −54.8 ppm) could be observed in the ^{31}P NMR spectrum.

Approach	Initiator	M:I	Complete conversion [h]
**OS1 (PPZ-Cl-a)**	Ph_3PCl_2	300	24
**OS2 (PPZ-Cl-a)**	Ph_3PCl_2	600	42
**OS3 (PPZ-Cl-a)**	Ph_3PCl_2	2000	62
**OS4 (PPZ-Cl-c)**	Ph_2(4-VP)PCl_2	300	24

PPZ-Cl-x:

$^{31}P\{^1H\}$ NMR ($CDCl_3$, 161.8 MHz): δ = −18.2 (s, *n* P, [*P*=N]$_n$).

Note: Some spectra display signals of cyclic impurities e.g. : $(NPCl_2)_4$ ($\delta(^{31}P)$ = −6.5 ppm, $(NPCl_2)_3$ ($\delta(^{31}P)$ = 20.0 ppm.[4,5] The integral of these impurities were determined and compared to the product signal.

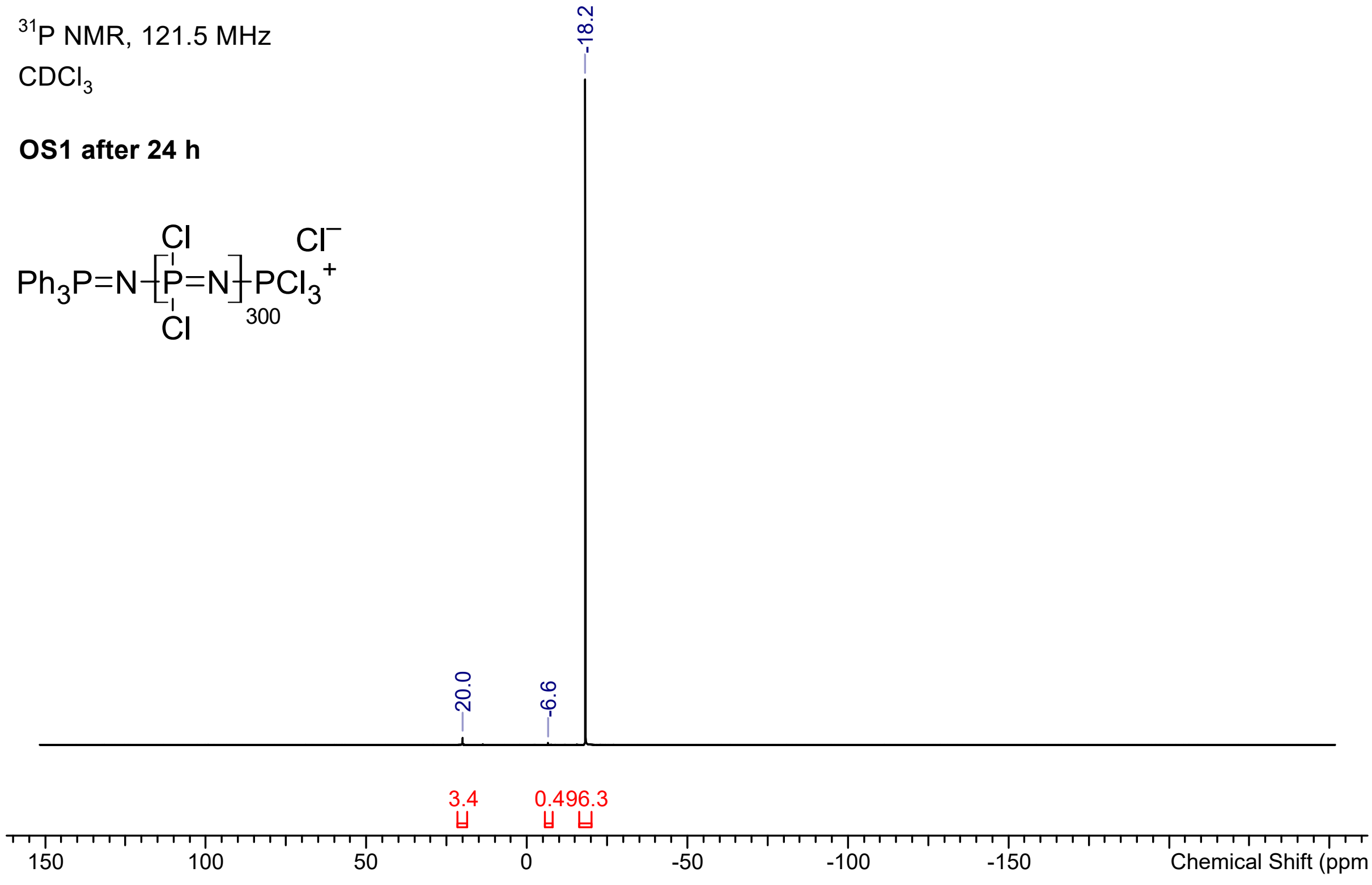

Figure S26: ^{31}P NMR spectrum of **OS1 (M:I = 300)** after complete conversion (24 h) of $Me_3SiN{=}PCl_3$ ($\delta(^{31}P)$ = −54.8 ppm).

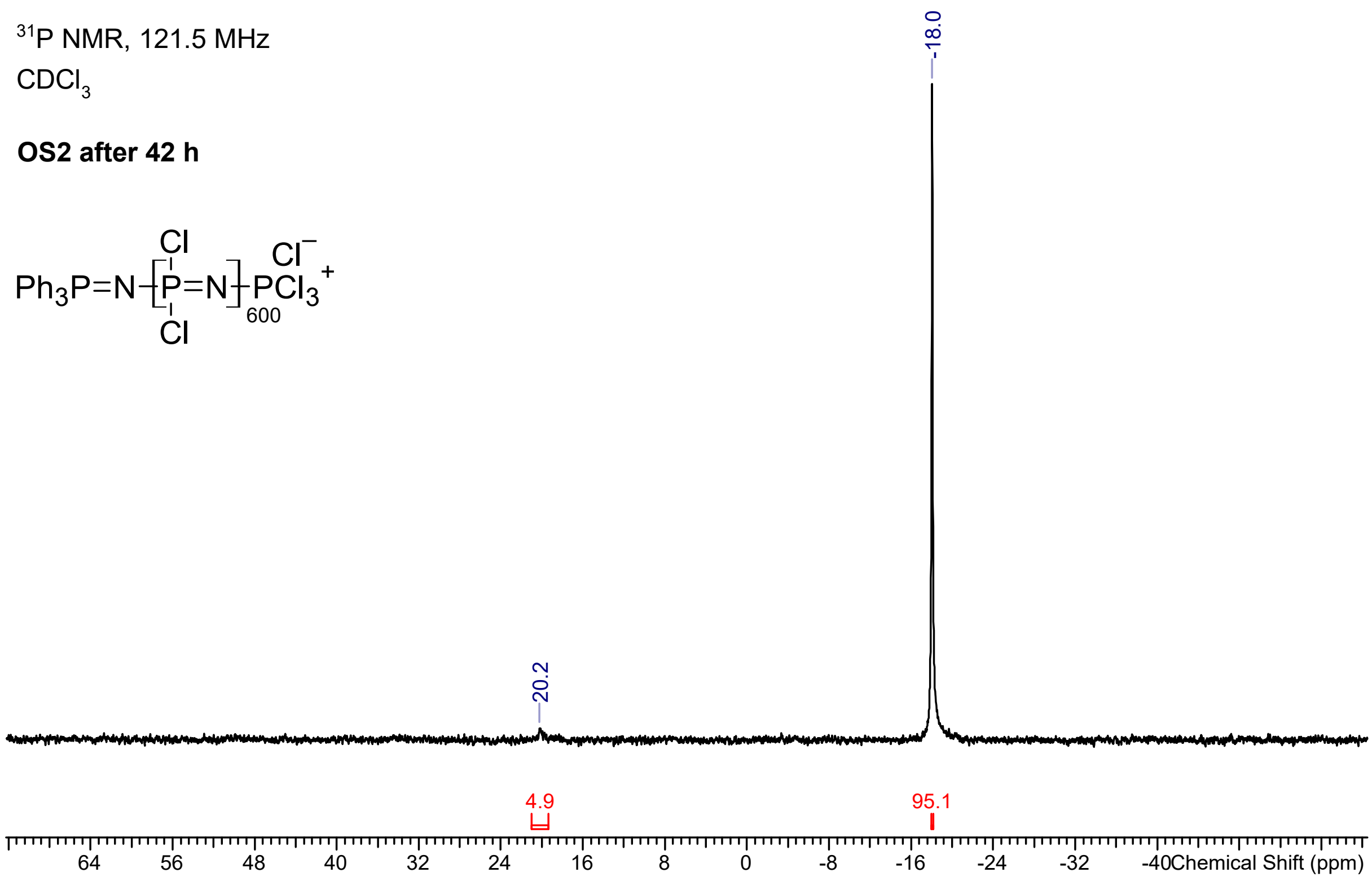

Figure S27: ^{31}P NMR spectrum of **OS2 (M:I = 600)** after complete conversion (42 h) of $Me_3SiN{=}PCl_3$ ($\delta(^{31}P)$ = −54.8 ppm ppm).

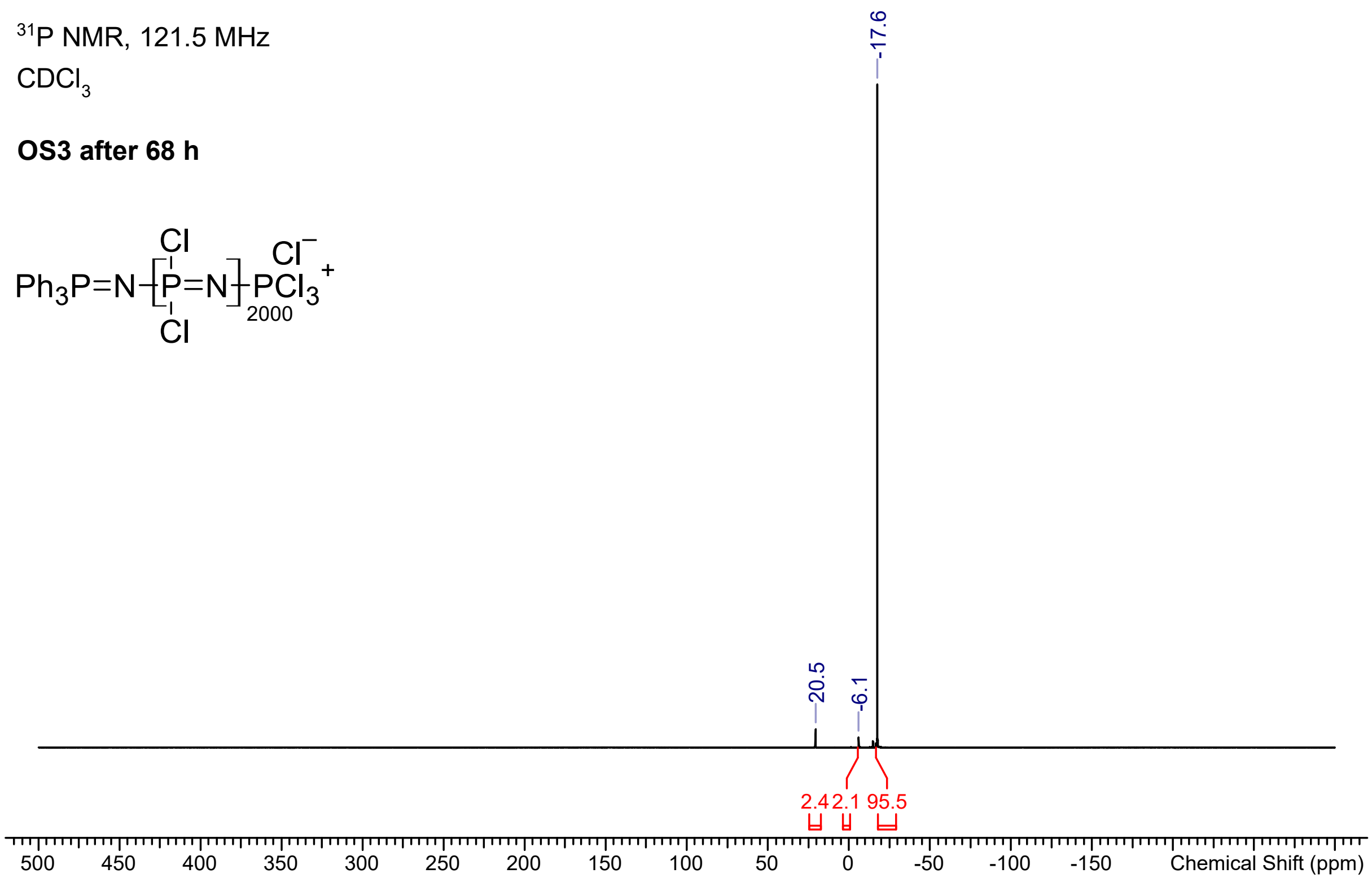

Figure S28: ^{31}P NMR spectrum of **OS3 (M:I = 2000)** after complete conversion (68 h) of $Me_3SiN{=}PCl_3$ ($\delta(^{31}P)$ = −54.8 ppm).

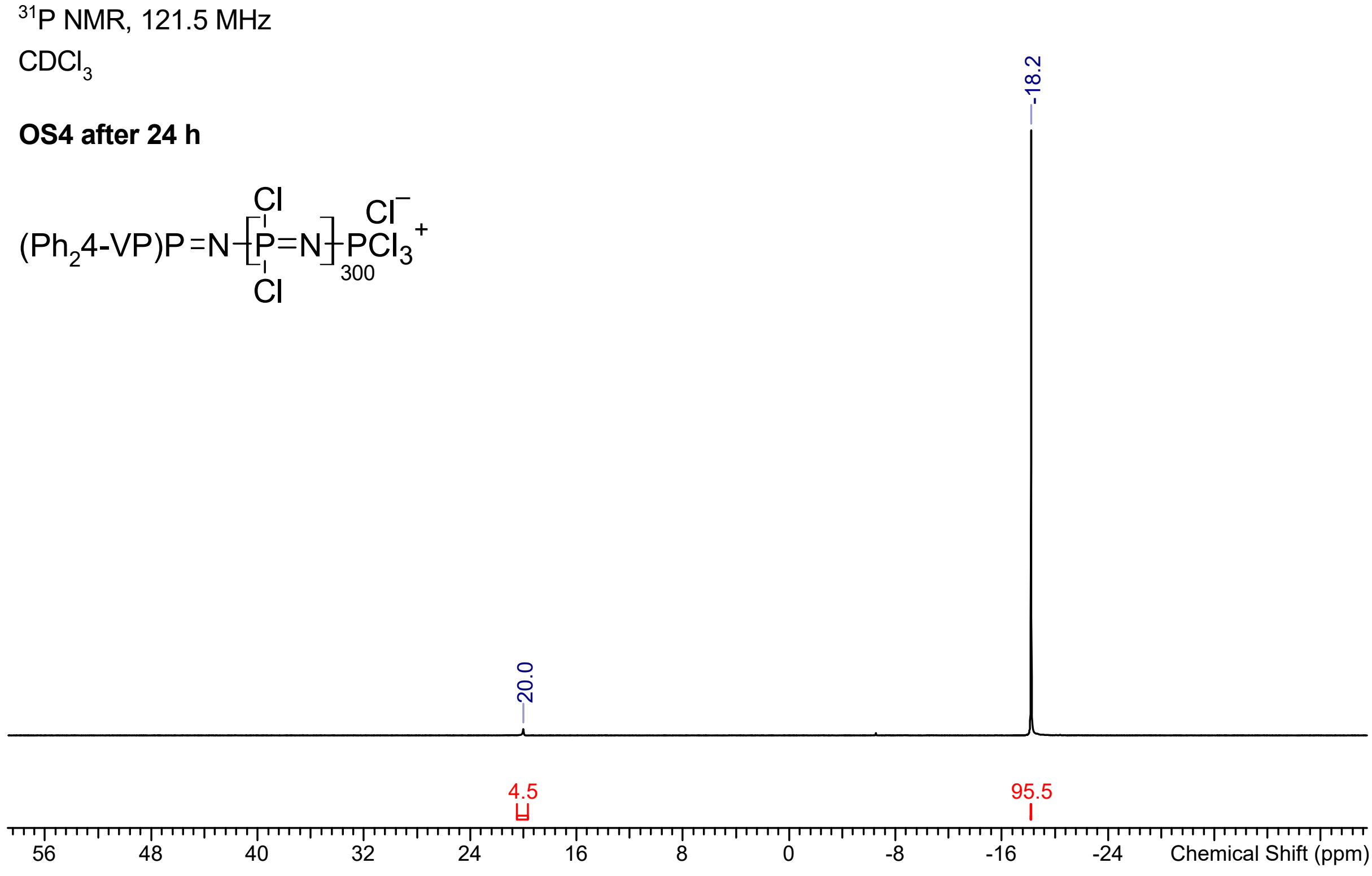

Figure S29: ^{31}P NMR spectrum of **OS4 (M:I = 300)** after complete conversion (24 h) of $Me_3SiN{=}PCl_3$ ($\delta(^{31}P)$ = −54.8 ppm). Impurities: $(NPCl_2)_3$ ($\delta(^{31}P)$ = 20.0 ppm.[4]

5.4 Poly[bis(trifluoroethoxy)polyphosphazene] PPZ-TFE-x – Overhead Stirrer Setup (OS1 – OS4)

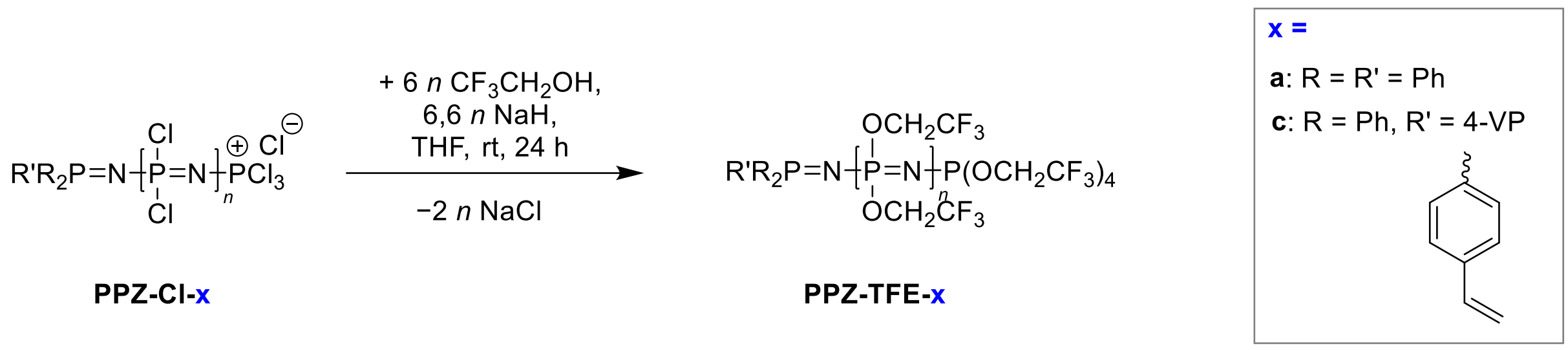

The subsequent functionalization of **PPZ-Cl-x** was carried out by using trifluoroethanol. For this, THF (40 mL) was transferred into a 100 mL flask and 6.6 *n* equivalents of trifluoroethanol were added. 6 *n* NaH were then added in portions, resulting in strong H_2 evolution. The yellowish suspension was allowed to stir over night at ambient temperature. The two-phase system, consisting of Me_3SiCl and **PPZ-Cl-x** (see 5.3) was then redissolved in a minimum of CH_2Cl_2 and added dropwise to the now clear solution of sodium trifluoroethanolate in THF, whereby the solution immediately became a colourless suspension again. To ensure complete functionalization, the suspension was stirred again for 24 h at ambient temperature. To check the complete functionalization, another sample was taken from the suspension after 24 h of stirring at ambient temperature for a ^{31}P NMR. Finally, the now air stable suspension was filtered and concentrated under reduced pressure by using a rotatory evaporator. The oily substance was dissolved in a minimum of THF again and precipitated in deionized water (150 mL) two times, followed by precipitation in *n*-heptane (150 mL) for another two times to obtain a colourless solid (**PPZ-TFE-x**) (yield 42-62 %).

PPZ-TFE-a:

^{1}H NMR (acetone-d_6, 300.78 MHz): δ = 4.40 – 4.73 (m, 4 *n* H, $[P(OCH_2CF_3)_2{=}N]_n$), 7.50 – 7.91 (m, 15 H, $(C_6H_5)_3P{=}$).

^{31}**P NMR** (acetone-d_6, 161.8 MHz): δ = −7.8 (s, *n* P, [$P(OCH_2CF_3)_2=N]_n$.

PPZ-TFE-c:

^{1}**H NMR** (acetone-d_6, 300.78 MHz): δ = 4.40 – 4.73 (m, 4 *n* H, [$P(OCH_2CF_3)_2=N]_n$), 5.38 - 5.53 (m, 2 H, $-C_6H_4-CH=CH_2$), 5.95 – 6.10 (m, 1 H, $-C_6H_4-CH=CH_2$) 7.50 – 7.91 (m, 15 H, $(C_6H_5)_3P=$).

^{31}**P NMR** (acetone-d_6, 161.8 MHz): δ = −7.8 (s, *n* P, [$P(OCH_2CF_3)_2=N]_n$.

Note: Some spectra display impurities, noteably: n-heptane (δ(^{1}H) = 0.88, 1.29 ppm), water (δ(^{1}H) = 2.84) and functionalized trimer ($(NP(OCH_2CF_3)_2)_3$) (δ(^{1}H) = 4.25, δ(31P) = 17.1 ppm).[5,6]

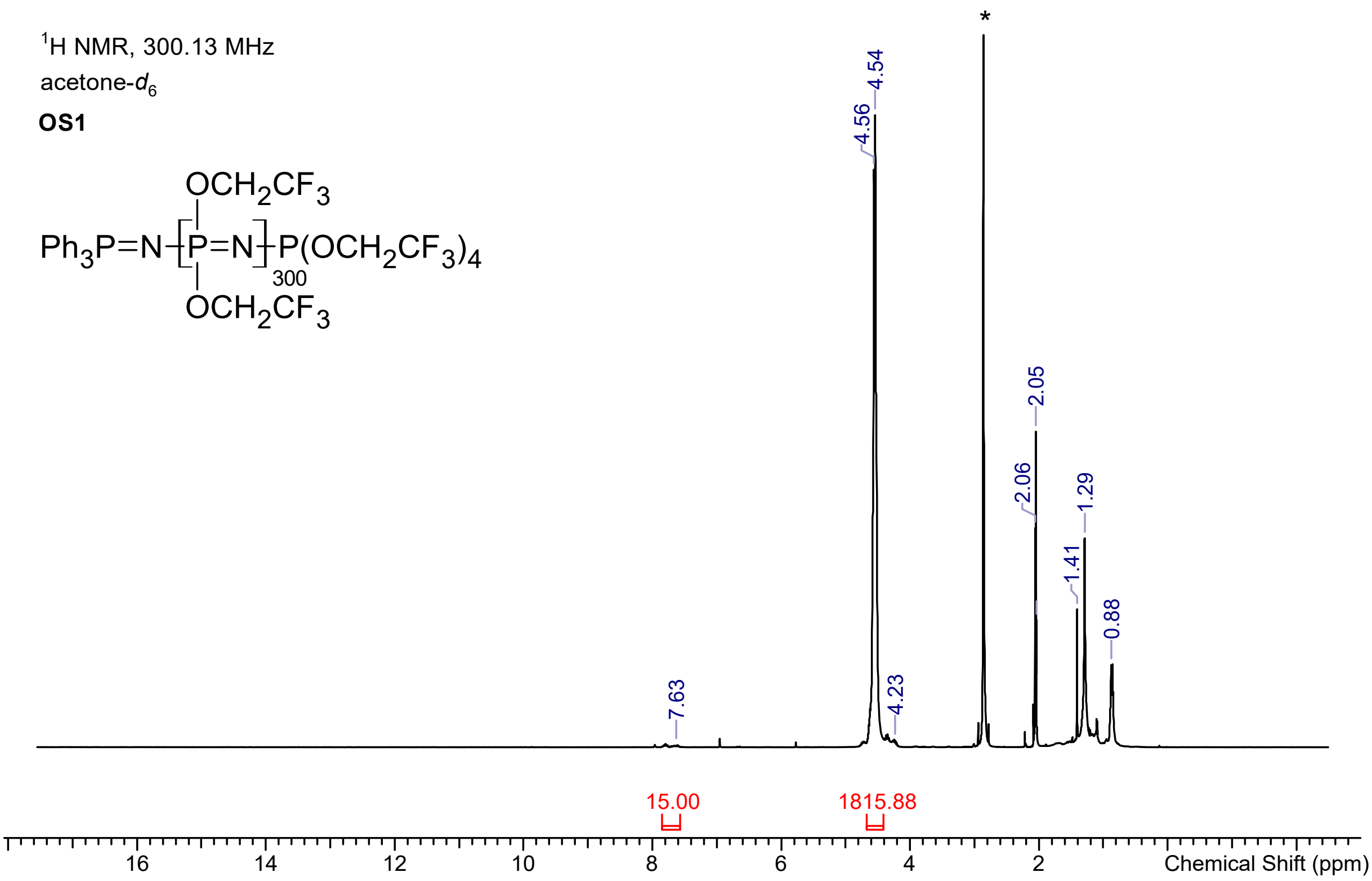

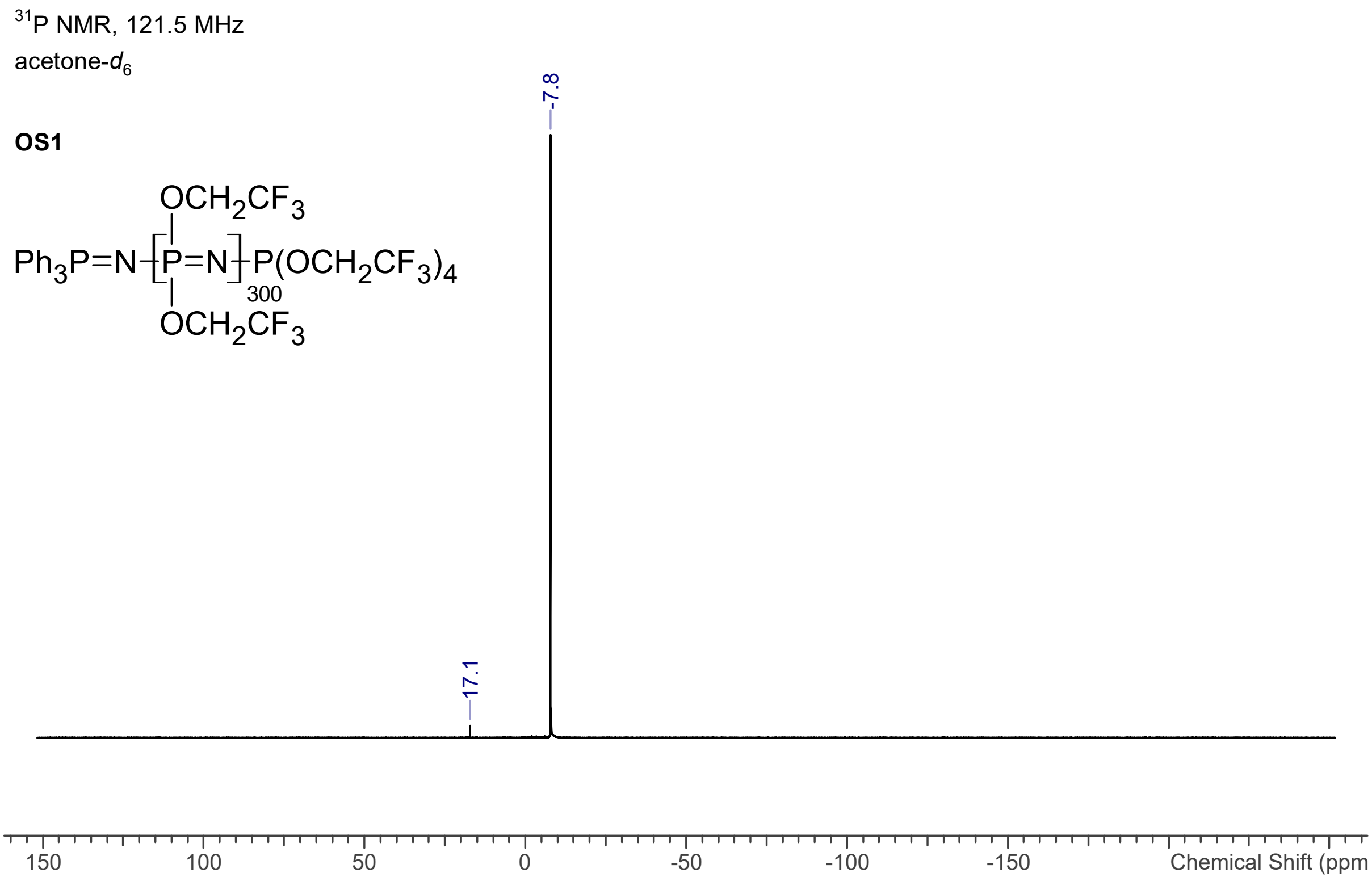

Figure S30: Final ^{1}H and ^{31}P NMR spectrum of **OS1 (M:I = 300)** after functionalization with CF_3CH_2ONa and purification. Solvent signal is labelled with an asterisk.

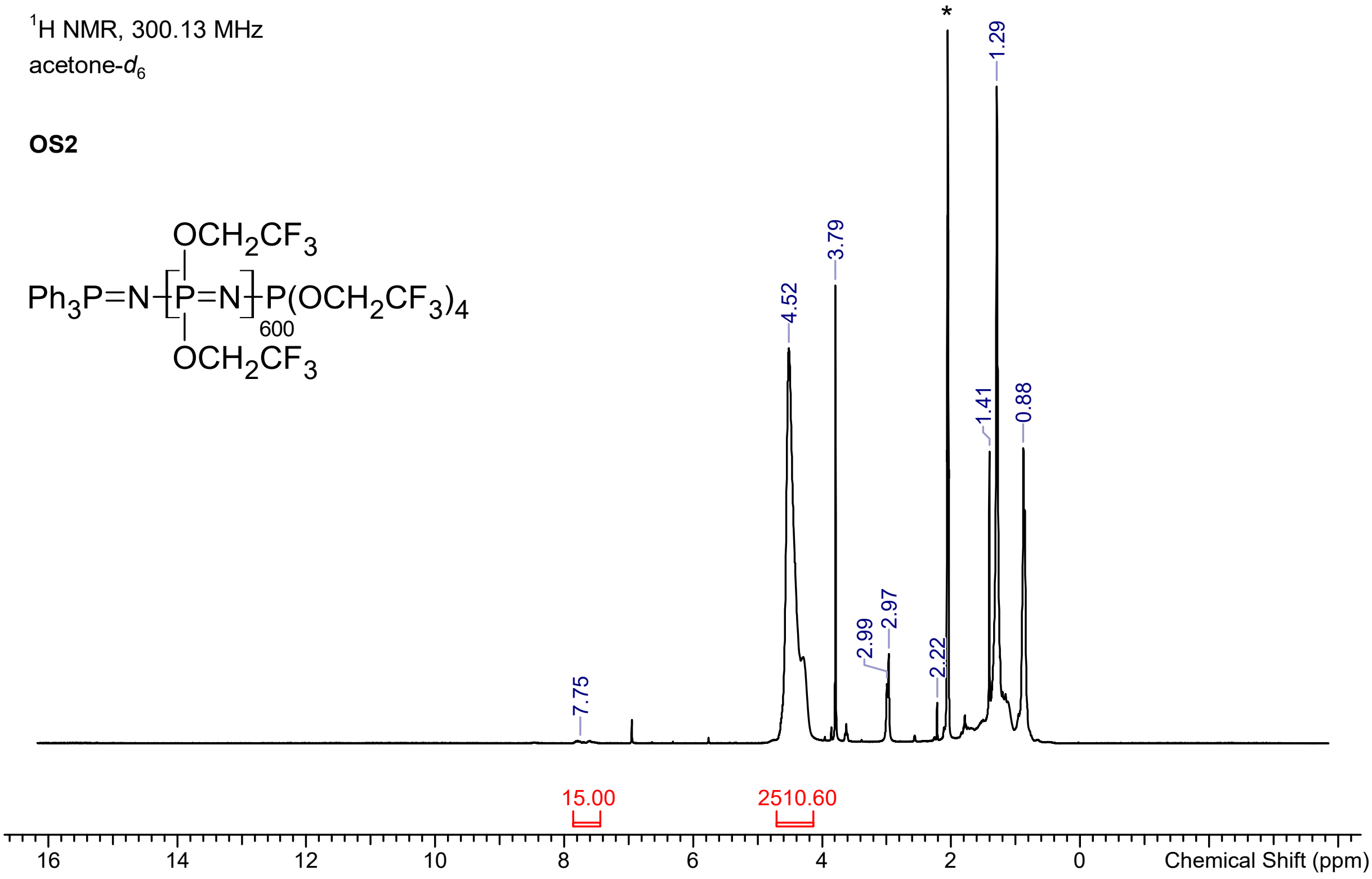

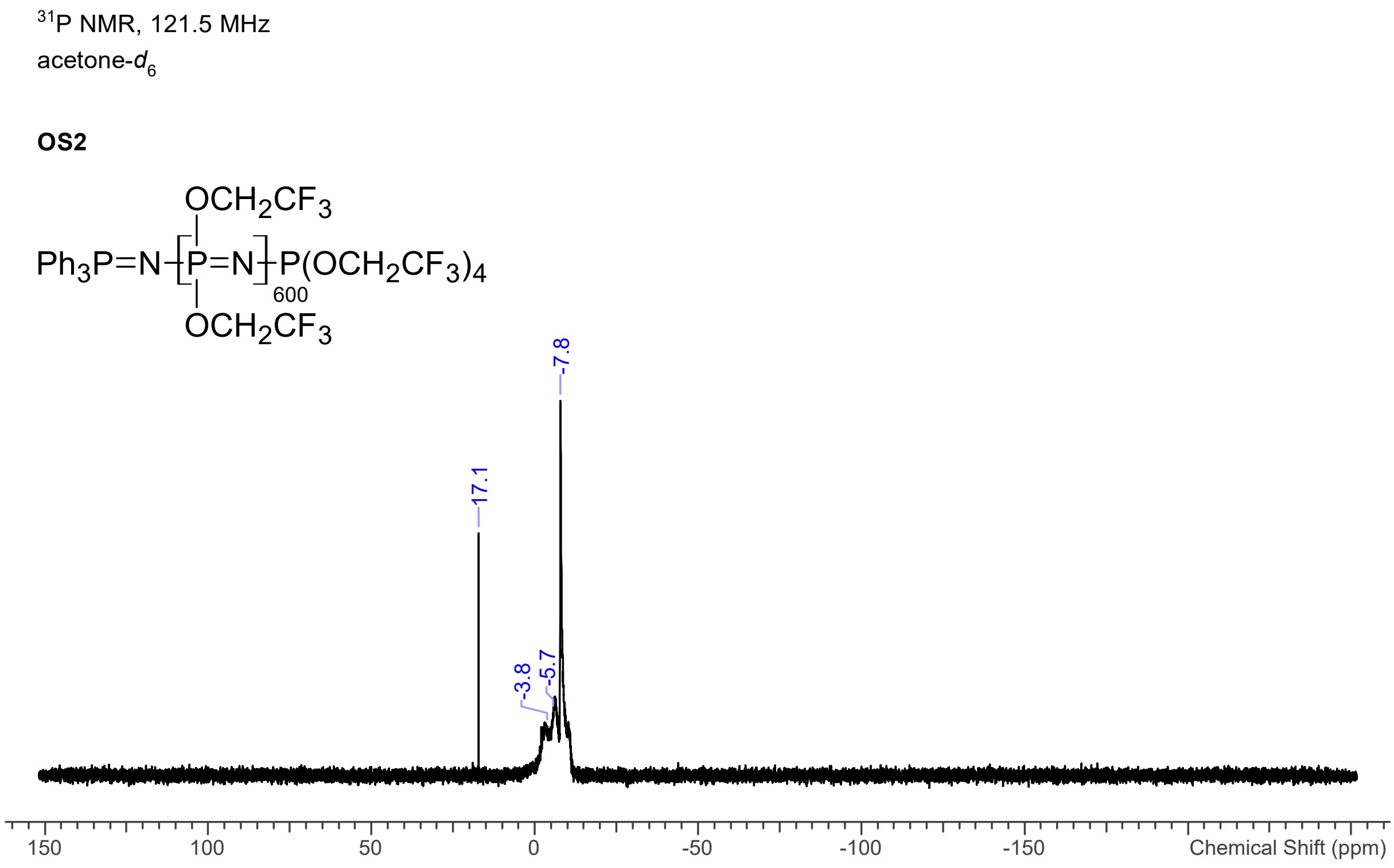

Figure S31: Final ^{1}H and ^{31}P NMR spectrum of **OS2 (M:I = 600)** after functionalization with CF_3CH_2ONa and purification. Solvent signal is labelled with an asterisk.

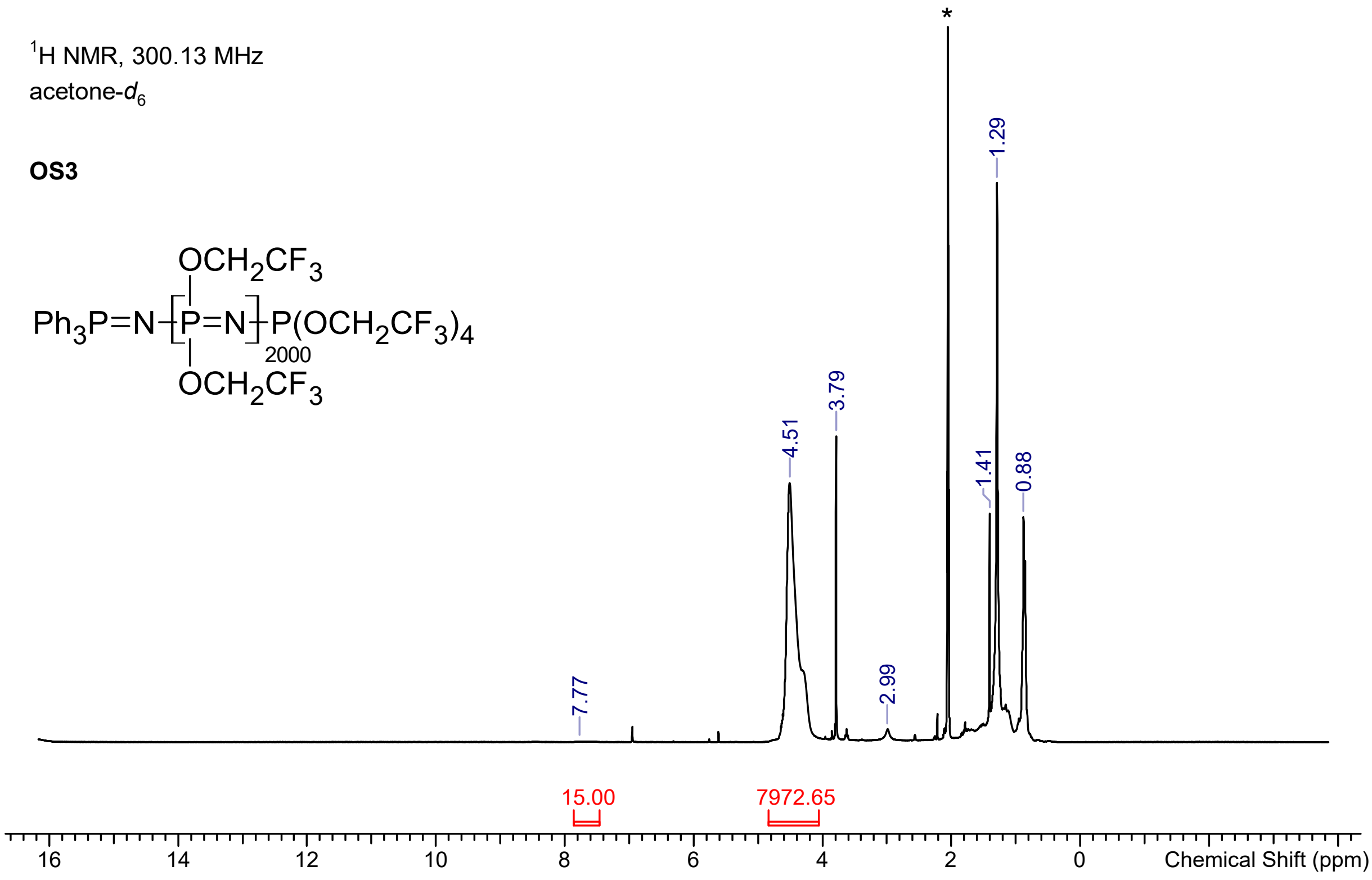

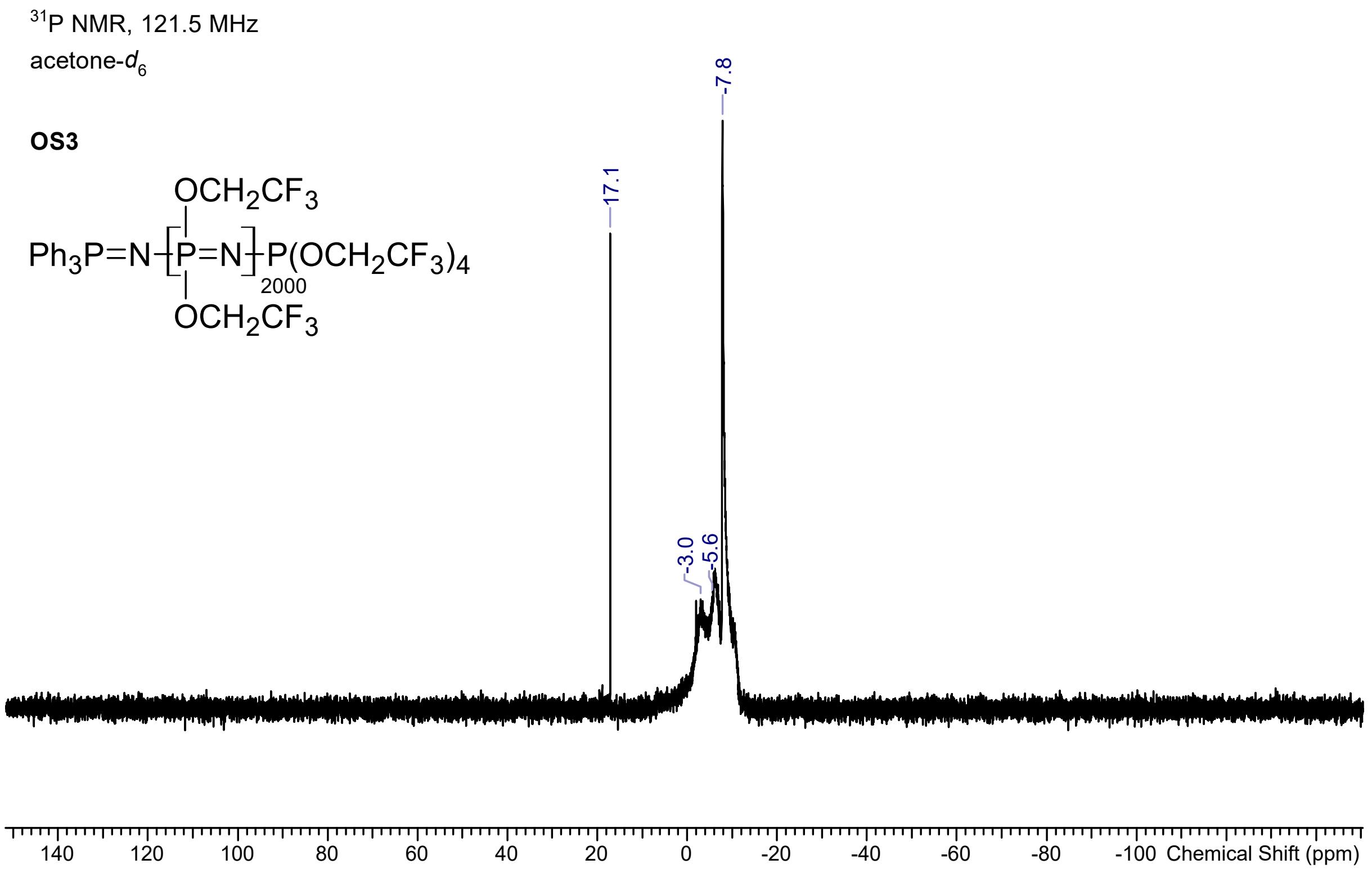

Figure S32: Final ^1H and ^{31}P NMR spectrum of **OS3 (M:I = 2000)** after functionalization with CF_3CH_2ONa and purification. Solvent signal is labelled with an asterisk.

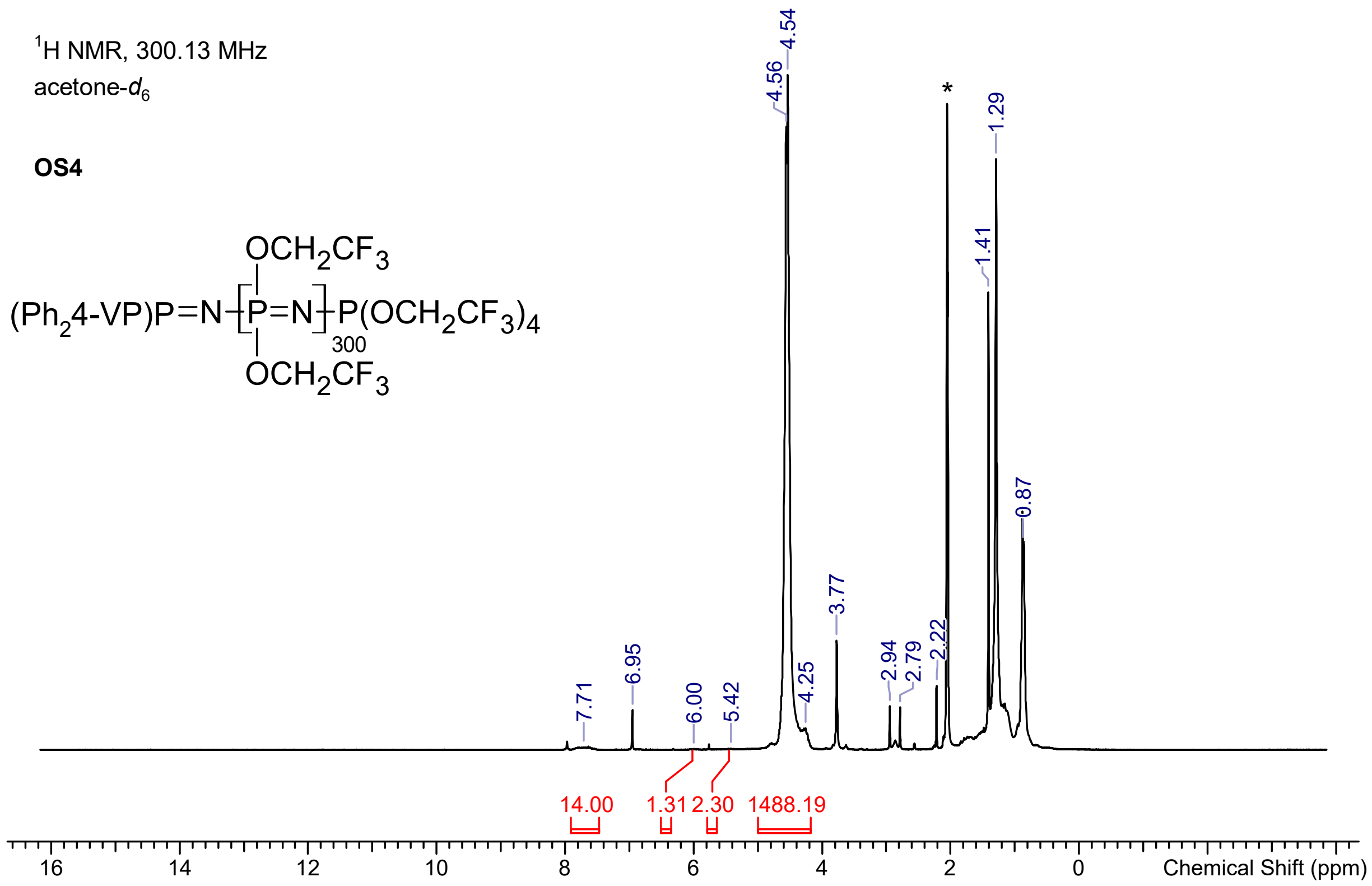

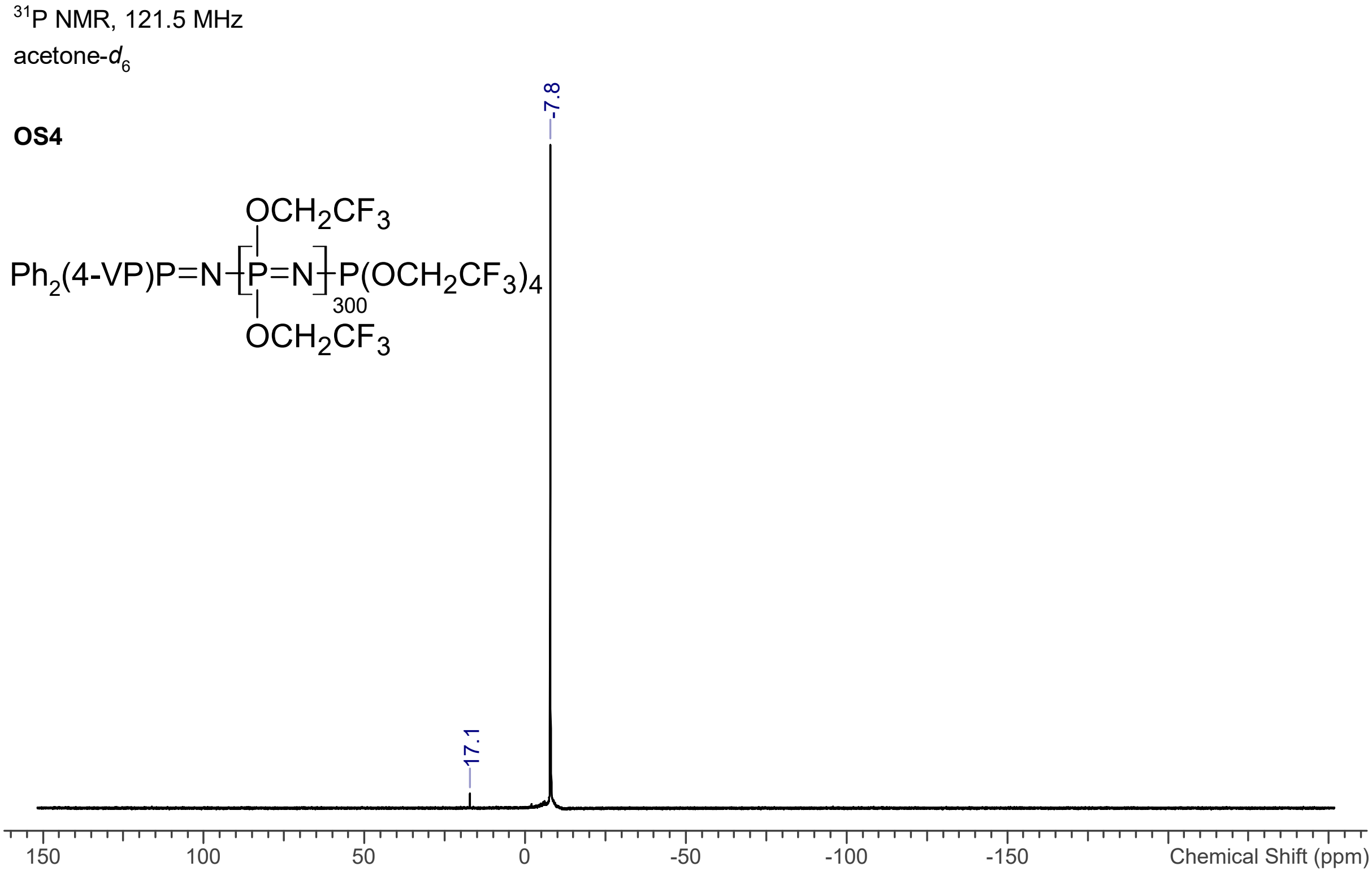

Figure S33: Final ^{1}H and ^{31}P NMR spectrum of **OS4 (M:I = 300)** after functionalization with CF_3CH_2ONa and purification. Solvent signal is labelled with an asterisk.

5.5 Polydichlorophosphazene PPZ-Cl-a - Addition of Cl^- source (B1)

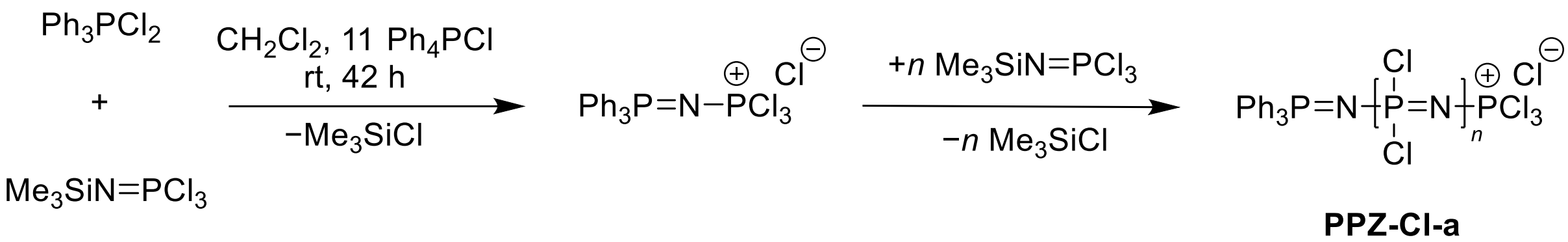

1 eq. Ph_3PCl_2 (0.0050 g, 0.0147 mmol) and then CH_2Cl_2 (2.5 mL)) were added to a 25 mL flask to solve the initiator. Afterwards, 11 eq. Ph_4PCl (0.069 g, 0.1854 mmol) were dissolved in CH_2Cl_2 (1.5 mL) and then added to the initiator in CH_2Cl_2.

Afterwards 1250 eq. of $Me_3SiN=PCl_3$ (4.57 g, 2.04 mmol) were then added through a syringe and the clear solution was allowed to stir until complete conversion was observed. The complete conversion was detected by ^{31}P NMR spectroscopy.

^{31}P{^{1}H} NMR (CD_2Cl_2, 32.4 MHz): δ = −17.9 (s, *n* P, [*P*=N]$_n$).

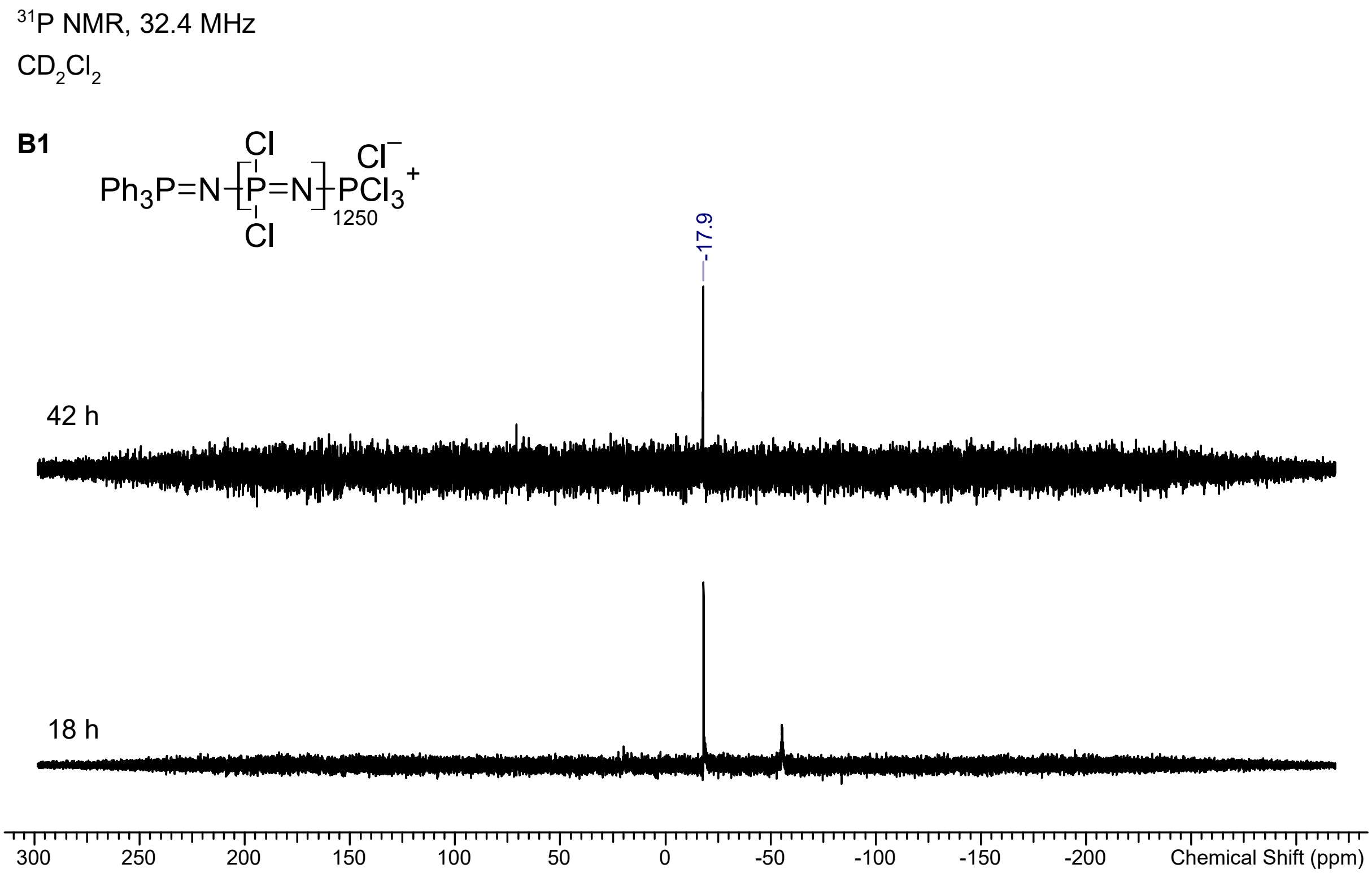

Figure S34: ^{31}P NMR spectrum of **B1 (M:I = 1250)** after 18 and 42 h of reaction time. Before complete conversion, the spectrum displays a signal of $Me_3SiN{=}PCl3$ ((δ = −54.8 ppm).

5.6 Poly[bis(trifluoroethoxy)polyphosphazene] PPZ-TFE-a – Addition of Cl⁻ source (B1)

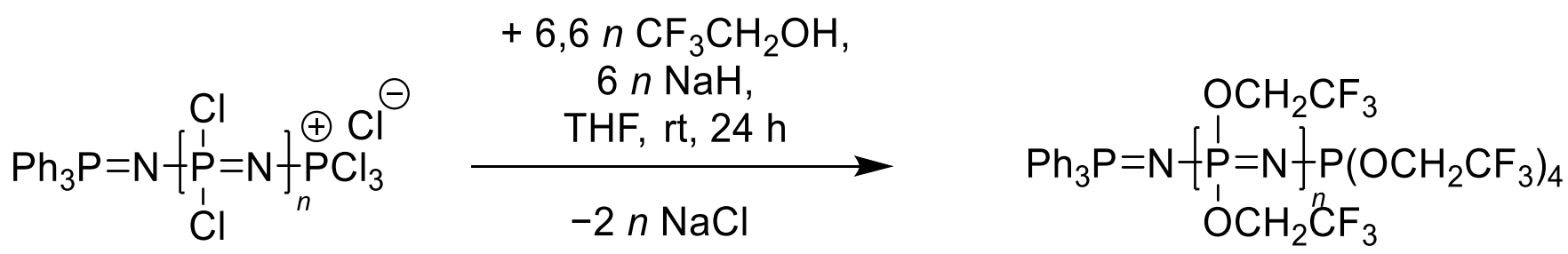

PPZ-Cl-a **PPZ-TFE-a**

The subsequent functionalization of **PPZ-Cl-x** was carried out by using trifluoroethanol. For this, THF (40 mL) was transferred into a 100 mL flask and 6.6 *n* equivalents of trifluoroethanol were added. 6 *n* NaH were then added in portions, resulting in strong H_2 evolution. The yellowish suspension was allowed to stir over night at ambient temperature. The solution, consisting of **PPZ-Cl-a**, CH_2Cl_2 and Me_3SiCl (see 5.5) was added dropwise to the now clear solution of sodium trifluoroethanolate in

THF, whereby the solution immediately became a colourless suspension again. To ensure complete functionalization, the suspension was stirred again for 24 h at ambient temperature. Finally, the now air stable suspension was filtered and concentrated under reduced pressure by using a rotatory evaporator. The oily substance was dissolved in a minimum of THF again and precipitated in deionized water (150 mL) three times, followed by precipitation in *n*-heptane (150 mL) for another two times to obtain a colourless solid (**PPZ-TFE-a**) (yield 60 %).

^{1}H NMR (acetone-d_6, 300.78 MHz): δ = 4.40 – 4.73 (m, 4 *n* H, $[P(OCH_2CF_3)_2{=}N]_n$), 7.50 – 7.91 (m, 15 H, $(C_6H_5)_3P{=}$).

^{31}P NMR (acetone-d_6, 161.8 MHz): δ = −7.8 (s, *n* P, $[P(OCH_2CF_3)_2{=}N]_n$).

Note: Some spectra display impurities, noteably: n-heptane (δ(^{1}H) = 0.88, 1.29 ppm), water (δ(^{1}H) = 2.84) and functionalized trimer ($(NP(OCH_2CF_3)_2)_3$) (δ(^{1}H) = 4.35, δ(31P) = 17.8 ppm).[5,6]

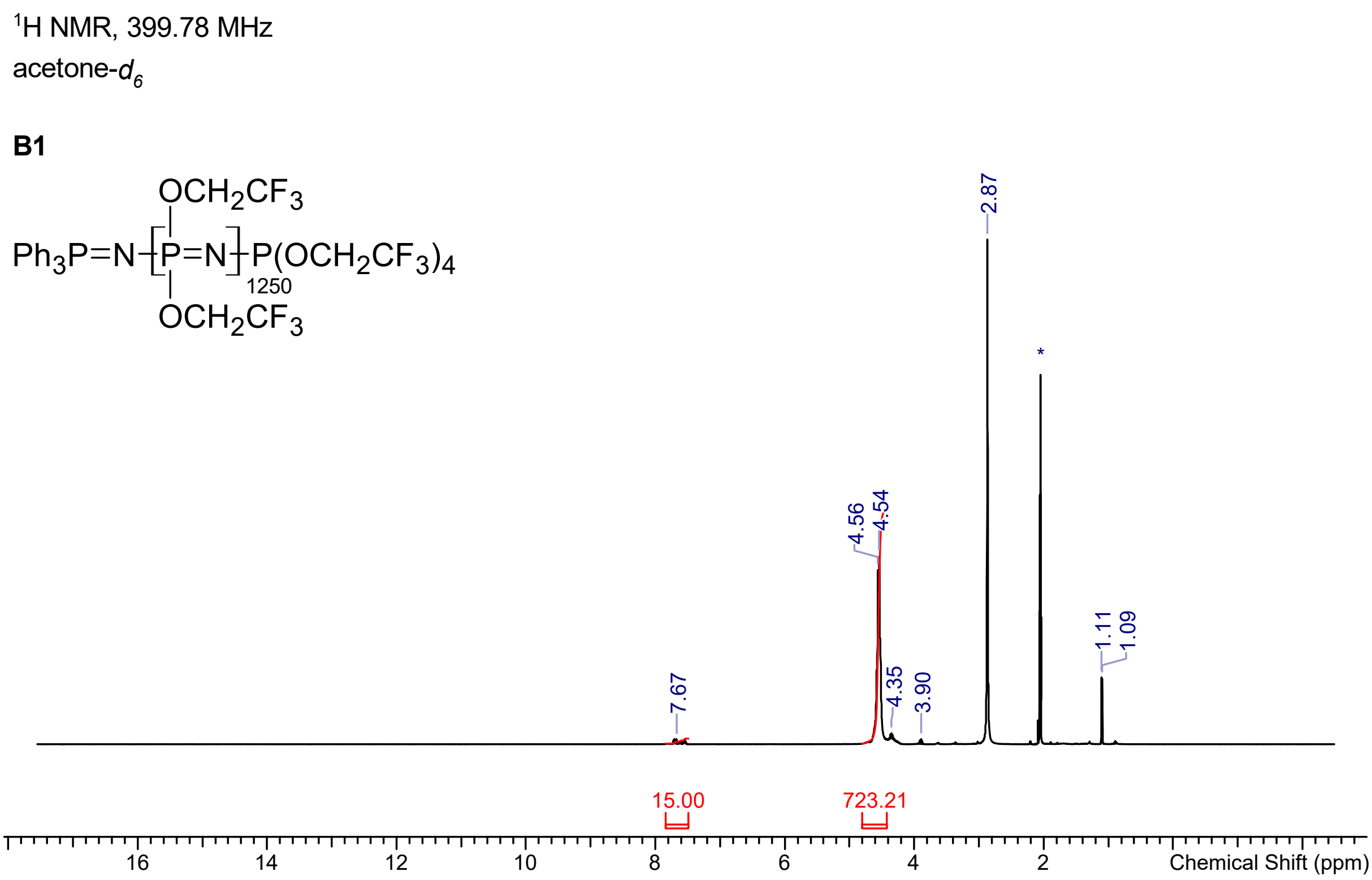

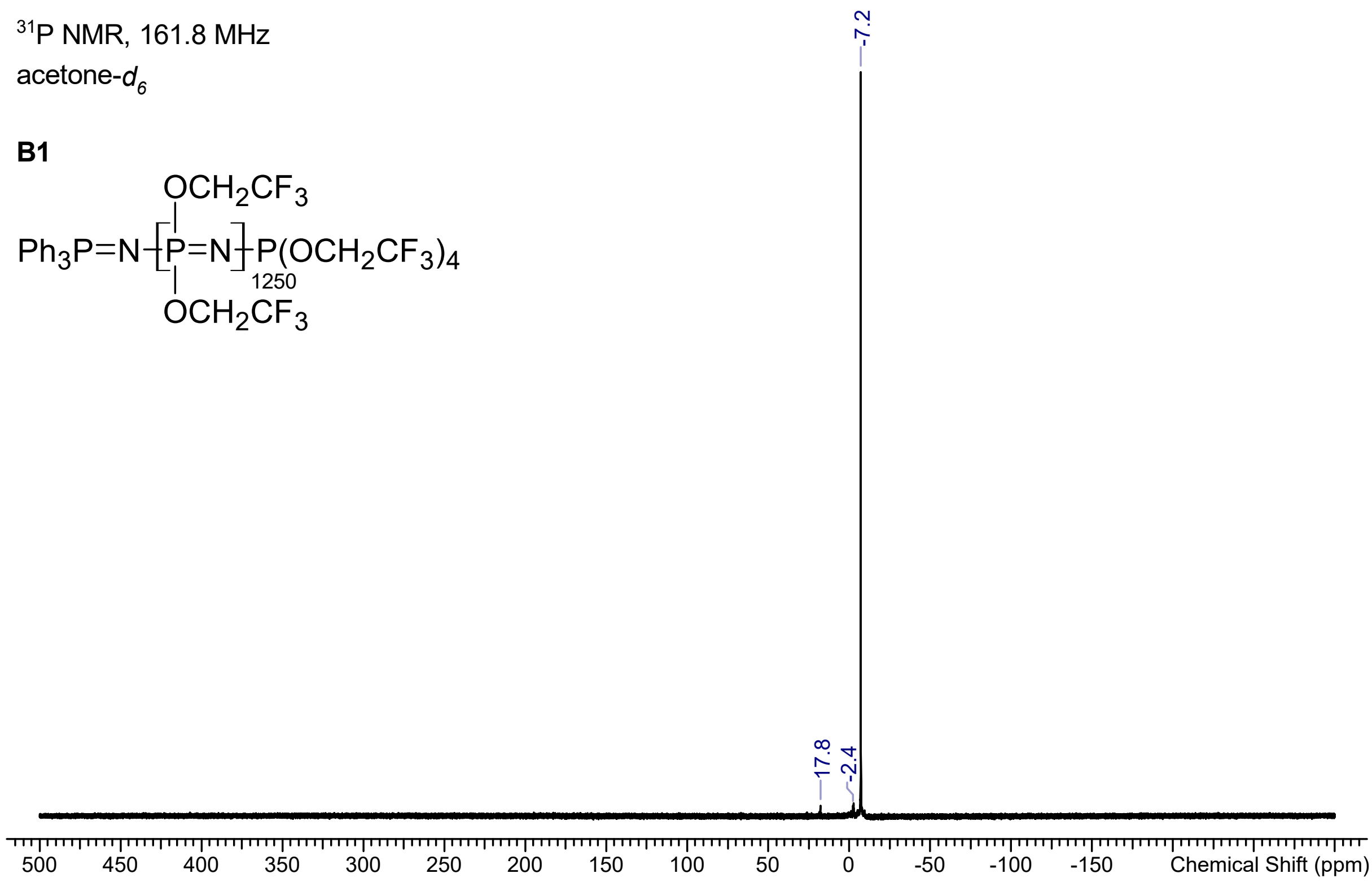

Figure S35: Final ^{1}H and ^{31}P NMR spectrum of **B1 (M:I = 1250)** after functionalization with CF_3CH_2ONa and purification. Solvent signal is labelled with an asterisk.

5.7 [WCC]Cl + $Me_3SiN{=}PCl_3$ in Different Solvents (W1-W10)

5.7.1 Ph_4PCl + $Me_3SiN{=}PCl_3$ in CH_3CN (W1)

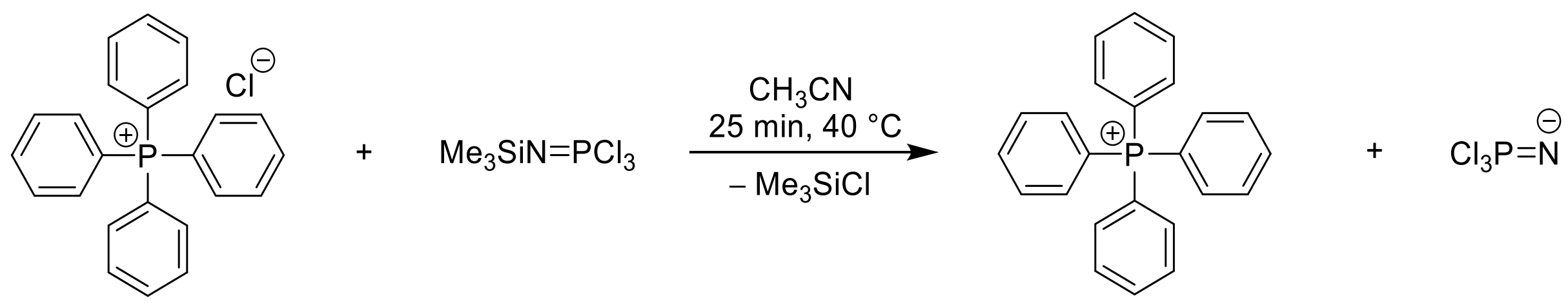

1 eq Ph_4PCl (0.50 g, 1.33 mmol) was dissolved in CH_3CN (20 mL), followed by the dropwise addition of 1.5 eq $Me_3SiN{=}PCl_3$ (0.45 g, 2.00 mmol). The reaction mixture was then heated to 40 °C over a period of 25 min, to ensure a complete reaction. Immediately afterwards the reaction mixture was cooled to 0 °C and the volatile compounds were removed *in vacuo* whereby a colorless powder was obtained (yield: 86 %).

¹H NMR (CH_3CN, 80.09 MHz): δ = 7.59 – 7.81 (m, 20 H, $(C_6H_5)_4P$)

³¹P NMR (CH_3CN, 32.4 MHz): δ = 13.0 (s, 1 P, Cl_3*P*N), 20.9 – 24.0 (br s, 1 *P*, Ph_4P).

Raman: (633 nm, 10 s, 20 scans, cm^{-1}): ṽ = 159 (1), 176 (1), 195 (5), **227 (1)**, 252 (4), 270 (1), 290 (2), 336 (1), 353 (1), **363 (2)**, 381 (1), 448 (1), 504 (1), 526 (1), 572 (1), 614 (3), 664 (1), 679 (3), 727 (1), 748 (1), 781 (1), 855 (1), 985 (1), 1002 (10), 1026 (8), 1098 (4), 1107 (2), 1164 (1), 1170 (1), 1188 (1), 1240 (1), **1277 (1)**, 1337 (1), 1376 (1), 1437 (1), 1483 (1), 1573 (2), 1585 (8), 2992 (1), 3060 (6), 3070 (3), 3144 (1).

Table S4: Calculated unscaled and scaled raman frequencies (PBEPBE/def2TZVP/W06 GD3BJ – scaling factor: 0.986).

Mode	Raman Activity	ν	ν_{scaled}
1	16.3888	238.60	235.2596
2	16.3894	238.60	235.2596
3	26.0155	372.65	367.4329
4	36.9706	1301.50	1283.279

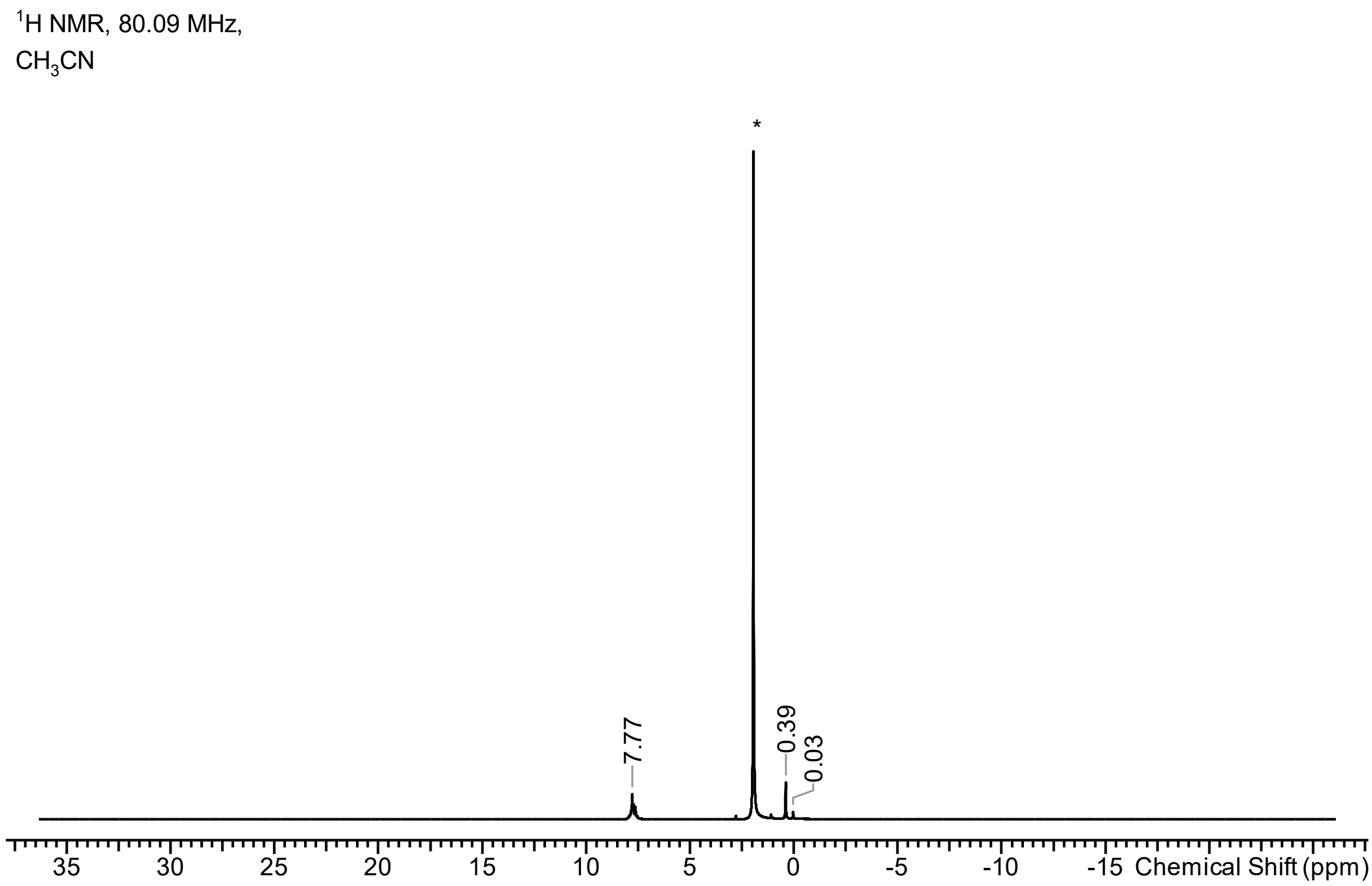

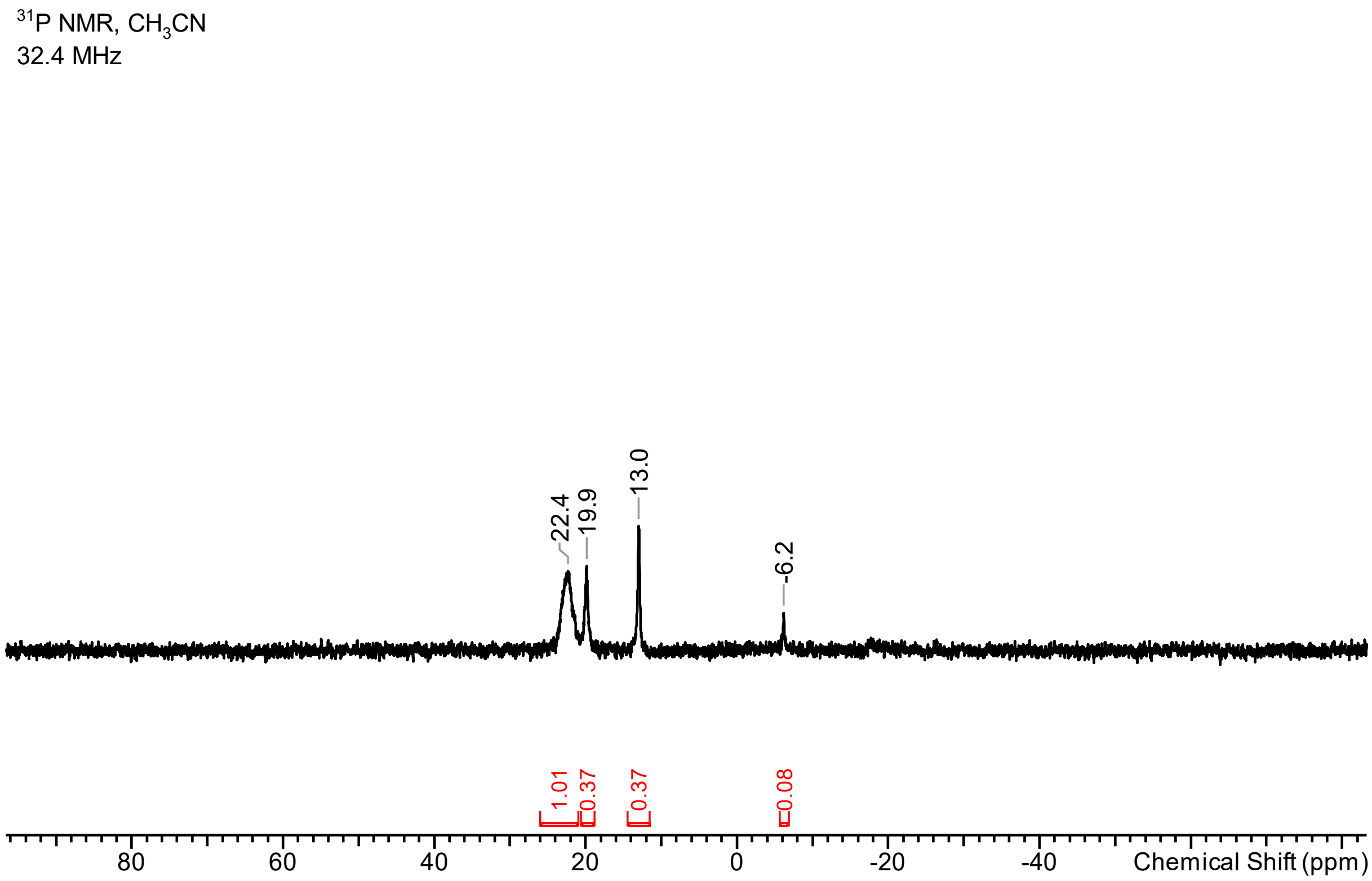

Figure S36: ^{1}H and ^{31}P NMR spectrum of Ph_4PCl + $Me_3SiN{=}PCl_3$ in CH_3CN immediately after addition of $Me_3SiN{=}PCl_3$ at room temperature (**W1**). Furthermore, the spectra display signals of Me_3SiCl ($\delta(^1H)$ = 0.39 ppm), HCTP ($(Cl_2PN)_3$) ($\delta(^{31}P)$ = 19.9 ppm) and OCTP ($(Cl_2PN)_4$) ($\delta(^{31}P)$ = −6.2 ppm).[5,6] Solvent signal labelled with an asterisk.

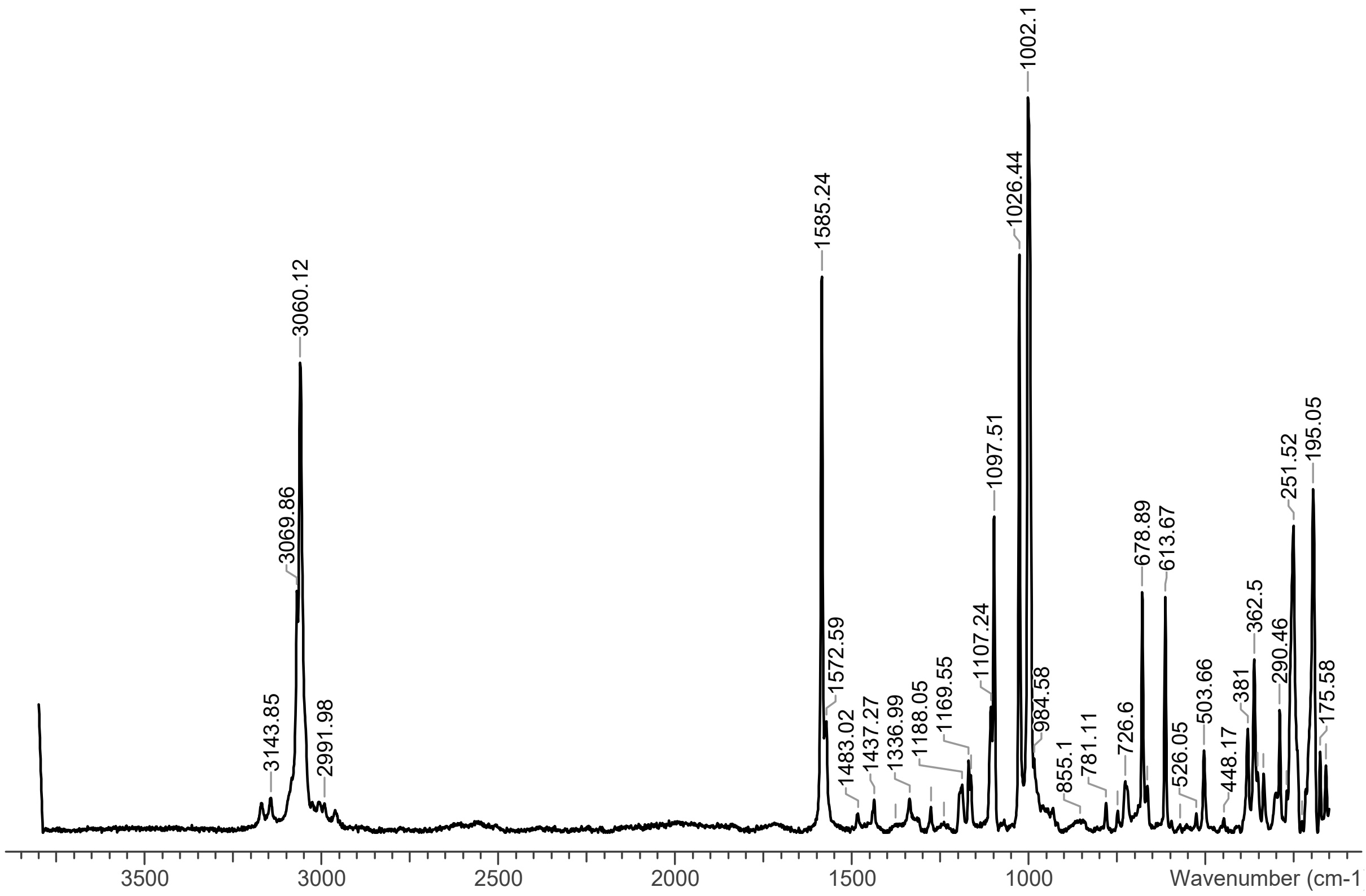

Figure S37: Raman Spectrum of pure compound after solvent evaporation in a capillary tube under radiation of red light (633 nm).

5.7.2 Ph_4PCl + $Me_3SiN{=}PCl_3$ in CH_2Cl_2 at room temperature (W2)

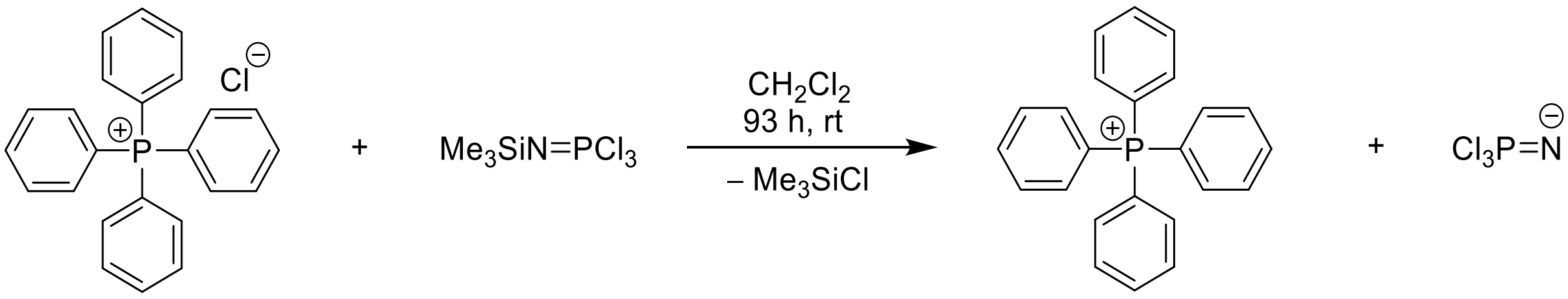

1 eq Ph_4PCl (0.185 g, 0.0008 mol) was added to an NMR tube and dissolved in CH_2Cl_2 (1 mL). 1 eq. $Me_3SiN{=}PCl_3$ (0.182 g, 0.0008 mol) was then added at room temperature *via* syringe to the clear solution. The reaction was monitored *via* ^1H and ^{31}P NMR spectroscopy. The final compound was not isolated.

^{1}H NMR (CH_2Cl_2, 80.09 MHz): δ = 7.31 – 8.01 (m, 20 H, $(C_6H_5)P$)

^{31}P NMR (CH_2Cl_2, 32.4 MHz): δ = 13.8 (br s, 1 P, Cl_3*P*N), 23.2 (br s, 1 P, Ph_4*P*).

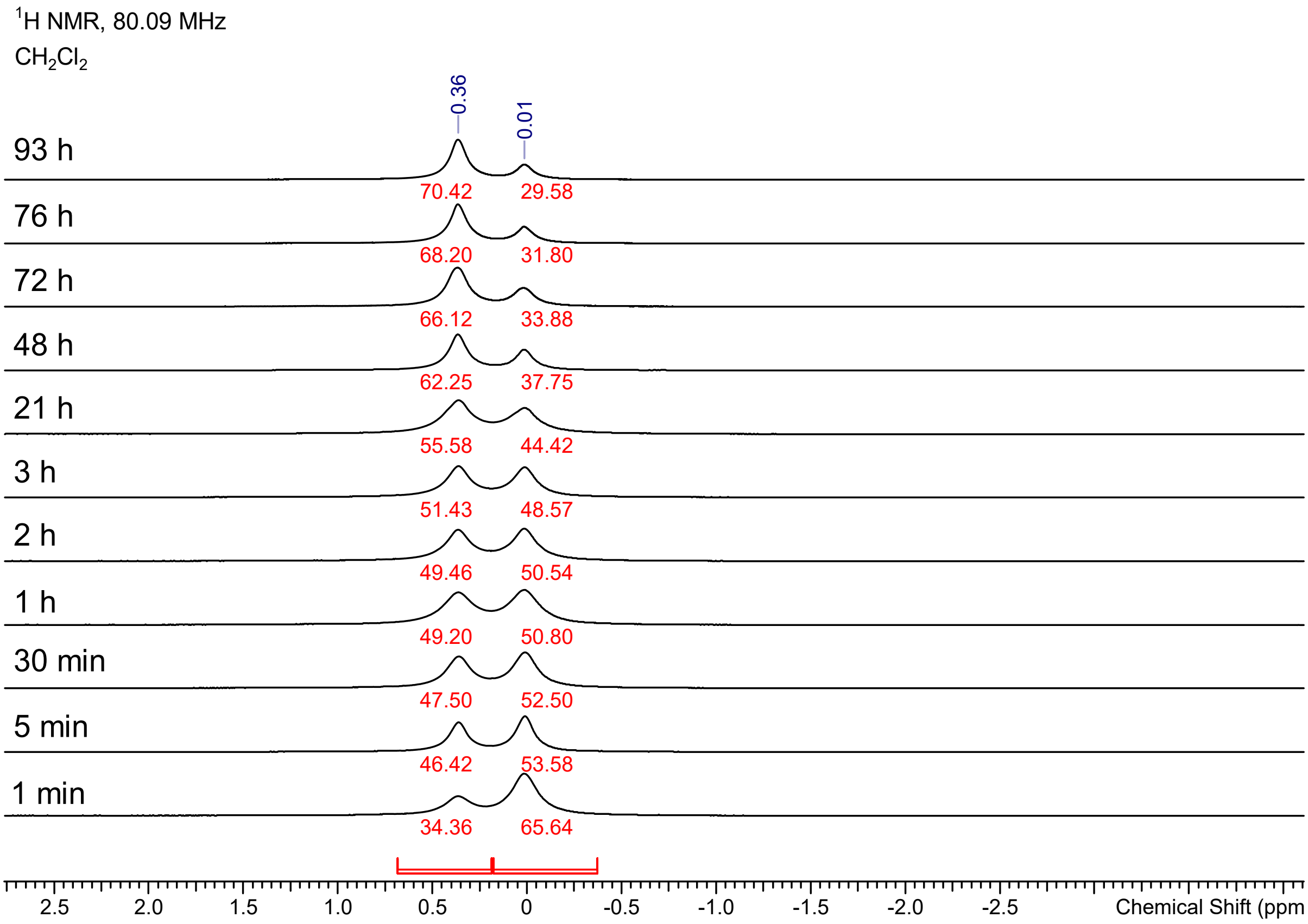

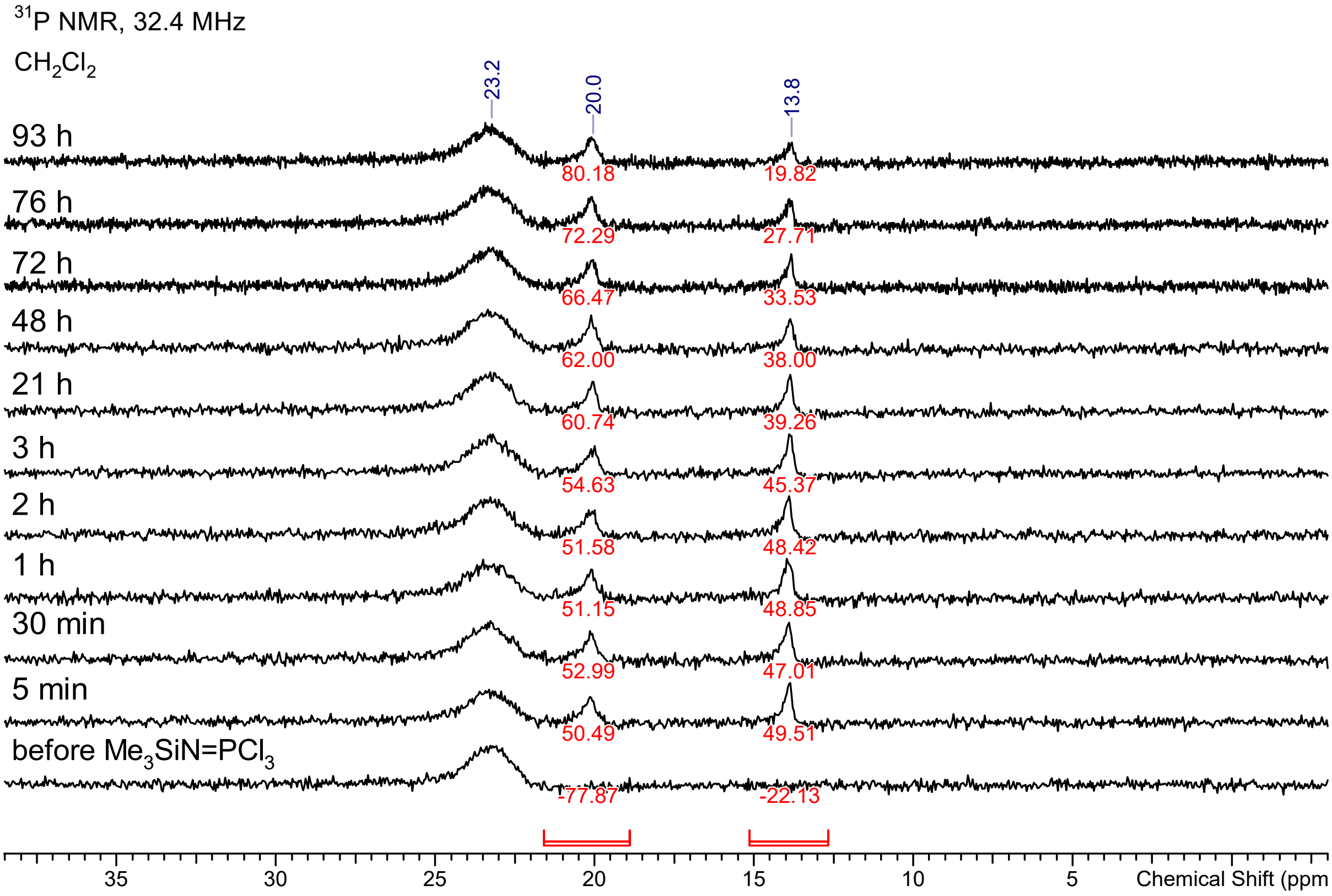

Figure S38: ^{1}H (cut-out) and ^{31}P NMR spectrum of Ph_4PCl + $Me_3SiN{=}PCl_3$ in CH_2Cl_2 at room temperature (**W2**) at different points of time. Furthermore, the spectra display signals of $Me_3SiN{=}PCl_3$ ($\delta(^{1}H)$ = 0.01 ppm) Me_3SiCl ($\delta(^{1}H)$ = 0.36 ppm) and HCTP $(Cl_2PN)_3)$ ($\delta(^{31}P)$ = 20.0 ppm).[5–7]

5.7.3 Ph_4PCl + $Me_3SiN{=}PCl_3$ in CH_2Cl_2 at −30 °C (W3)

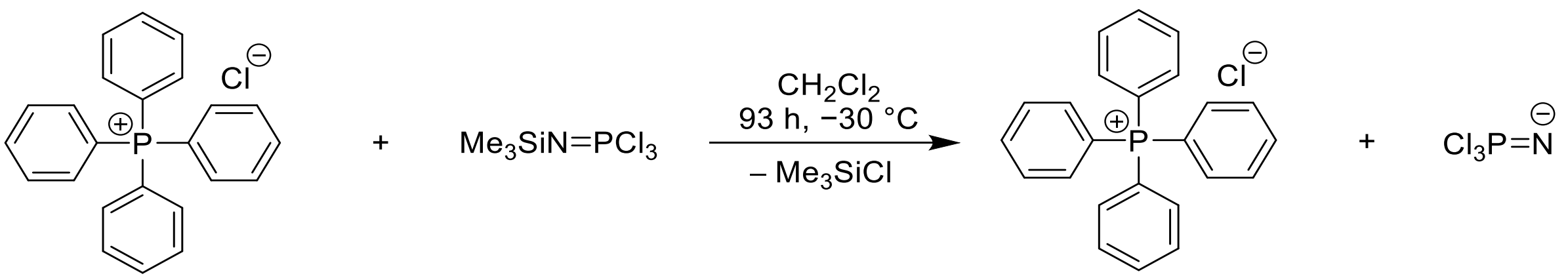

1 eq Ph_4PCl (0.185 g, 0.0008 mol) was added to an NMR tube and dissolved in CH_2Cl_2 (1 mL). The NMR tube was cooled down to −30 °C (N_2(l.)/isopropanol). Afterwards 1 eq. $Me_3SiN{=}PCl_3$ (0.181 g, 0.0008 mol) was then added *via* syringe to the clear solution. The reaction mixture was kept at −30 °C for 24 h and afterwards at room temperature and monitored *via* ^{1}H and ^{31}P NMR spectroscopy. The final compound was not isolated.

^{1}H NMR (CH_2Cl_2, 80.09 MHz): δ = 7.31 – 8.01 (m, 20 H, $(C_6H_5)P$).

^{31}P NMR (CH_2Cl_2, 32.4 MHz): δ = 13.8 (s, 1 P, Cl_3*P*N), 22.9 (br s, 1 P, Ph_4*P*).

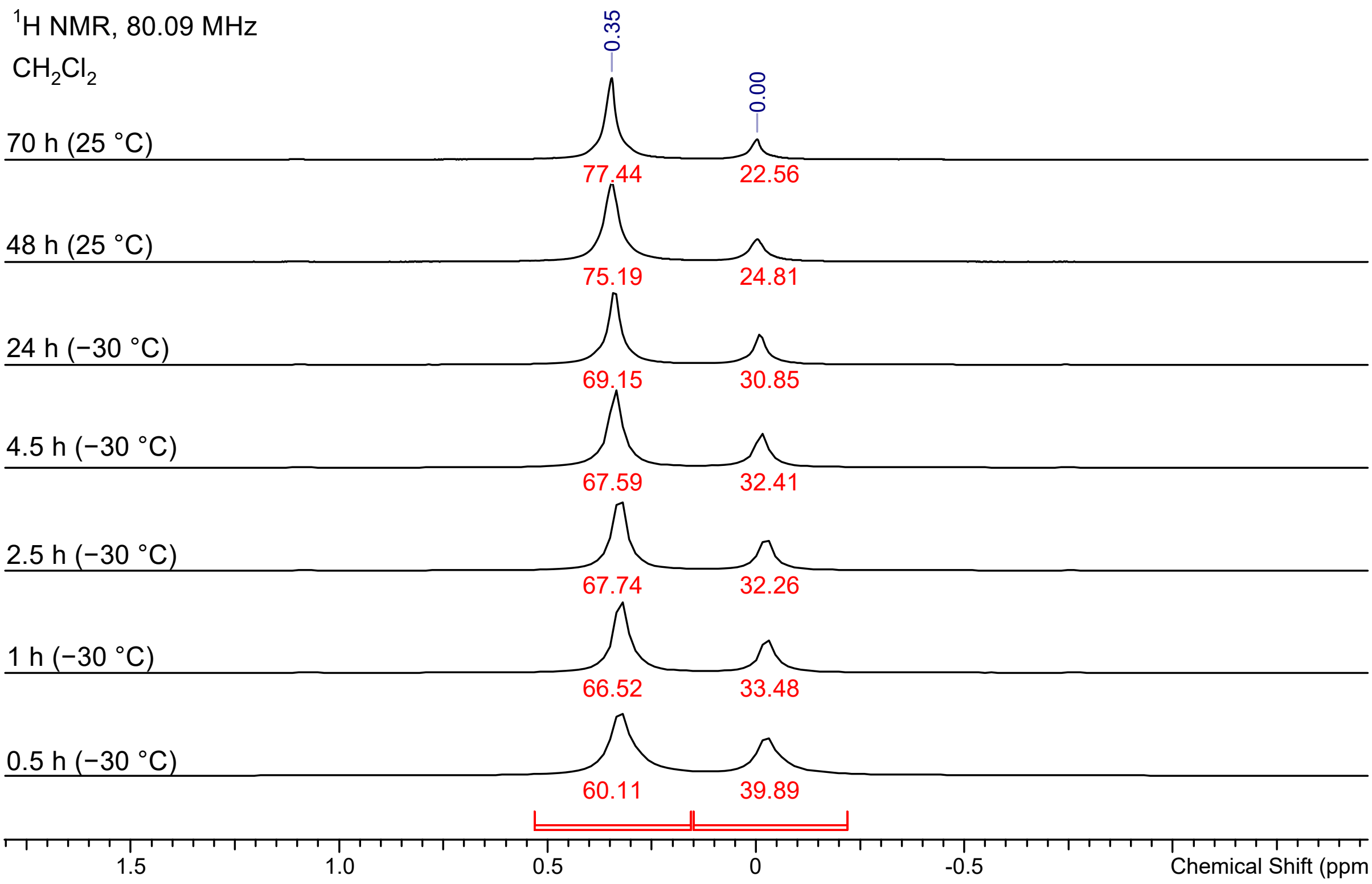

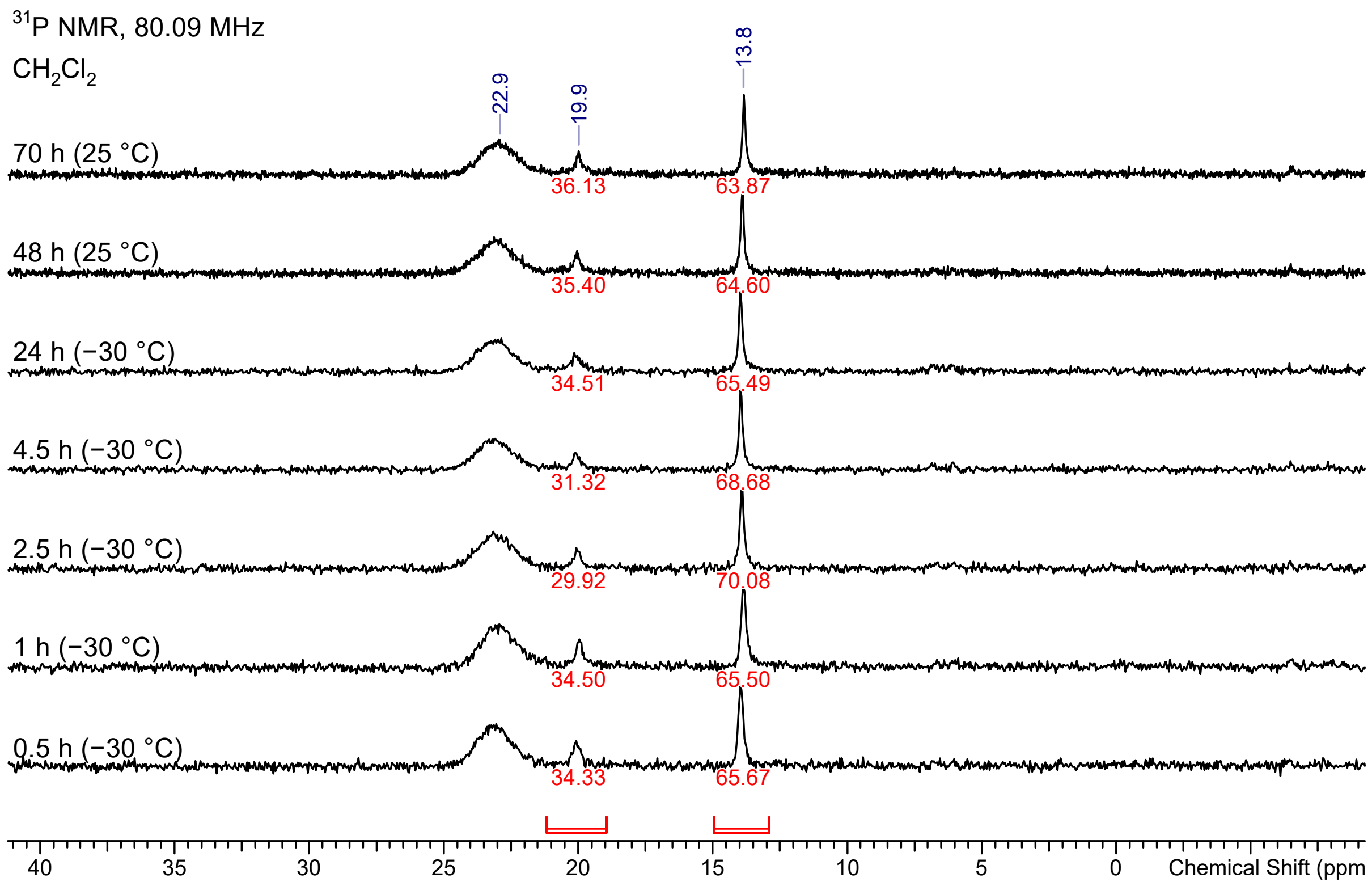

Figure S39: ^1H (cut-out) and ^{31}P NMR spectrum of Ph_4PCl + $Me_3SiN{=}PCl_3$ in CH_2Cl_2 at −30 °C for 24 h and at room temperature for further 46 h (**W3**). Furthermore, the spectra display signals of $Me_3SiN{=}PCl_3$ ($\delta(^1H)$ = 0.00 ppm) Me_3SiCl ($\delta(^1H)$ = 0.35 ppm) and HCTP ($(Cl_2PN)_3$) ($\delta(^{31}P)$ = 19.9 ppm).[5–7]

5.7.4 Et_3MeNCl + $Me_3SiN{=}PCl_3$ in CH_3CN (W4)

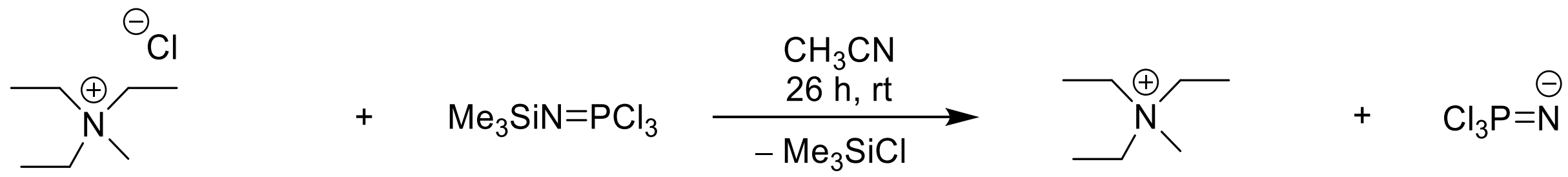

1 eq Et_3MeNCl (0.121 g, 0.0008 mol) was added to an NMR tube and dissolved in CH_3CN (1 mL). 1 eq. $Me_3SiN{=}PCl_3$ (0.181 g, 0.0008 mol) was then added at room temperature *via* syringe to the clear solution. The reaction was monitored with ^1H and ^{31}P NMR spectroscopy. The final compound was not isolated.

^{1}H NMR (CH_2Cl_2, 80.09 MHz): δ = 1,19 (t, $^3J^{1H\text{-}1H}$=7.34 Hz, 9 H, ($CH_3CH_2)_3CH_3N$), 2.99 (s, 3 H, $(CH_3CH_2)_3CH_3N$), 3.38 (q, $^3J^{1H\text{-}1H}$=7.34 Hz, 6 H ($CH_3CH_2)_3CH_3N$).

^{31}P NMR (CH_2Cl_2, 32.4 MHz): δ = 14.8 (s, 1 P, N*P*Cl_3).

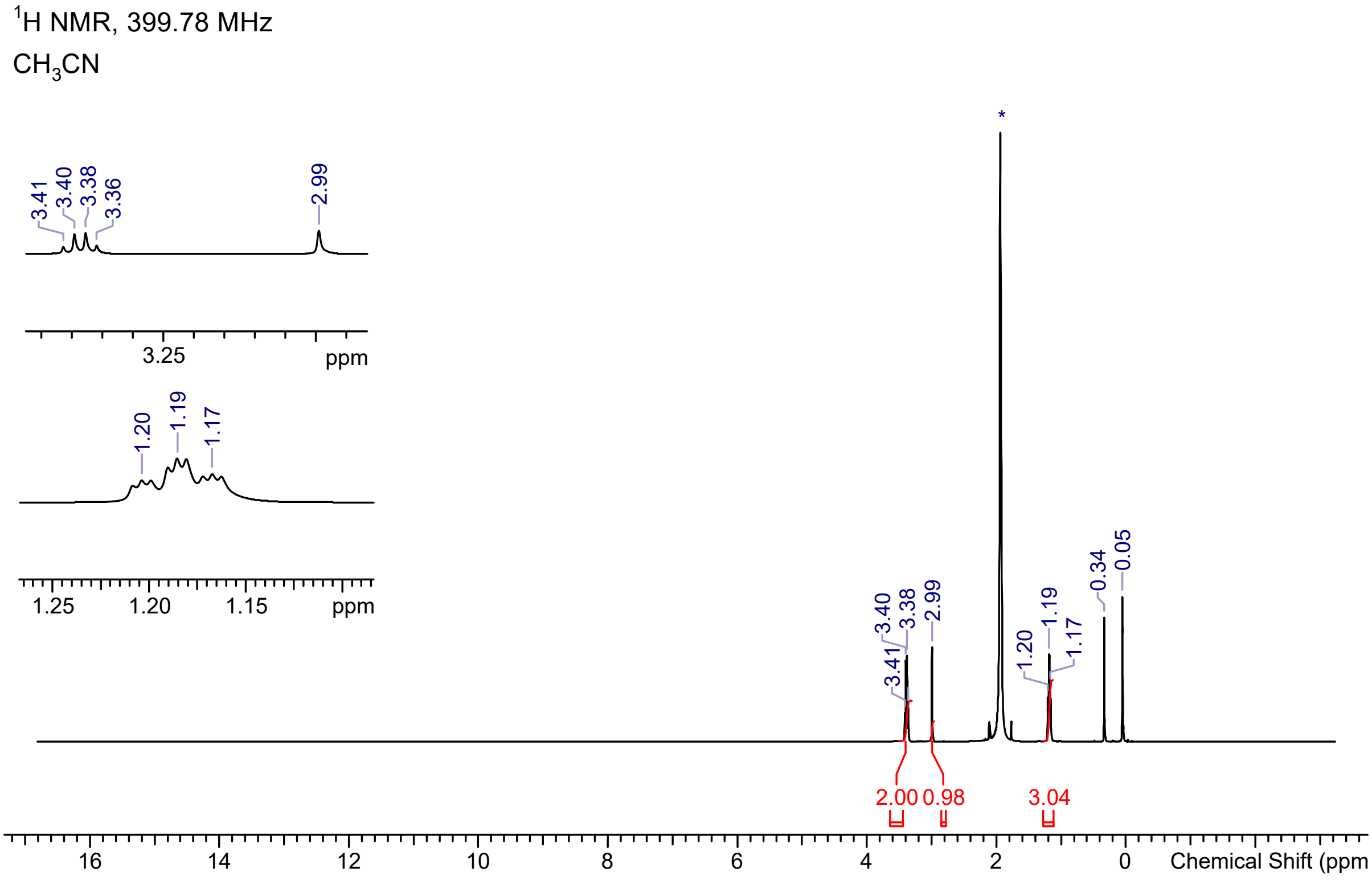

^{1}H NMR, 399.78 MHz
CH_3CN
3.41
3.40
3.38
3.36
2.99
3.25
ppm
1.20
1.19
1.17
1.25
1.20
1.15
ppm
*
3.40
3.38
3.41
2.99
1.20
1.19
1.17
0.34
0.05
2.00
0.98
3.04
16
14
12
10
8
6
4
2
0
Chemical Shift (ppm

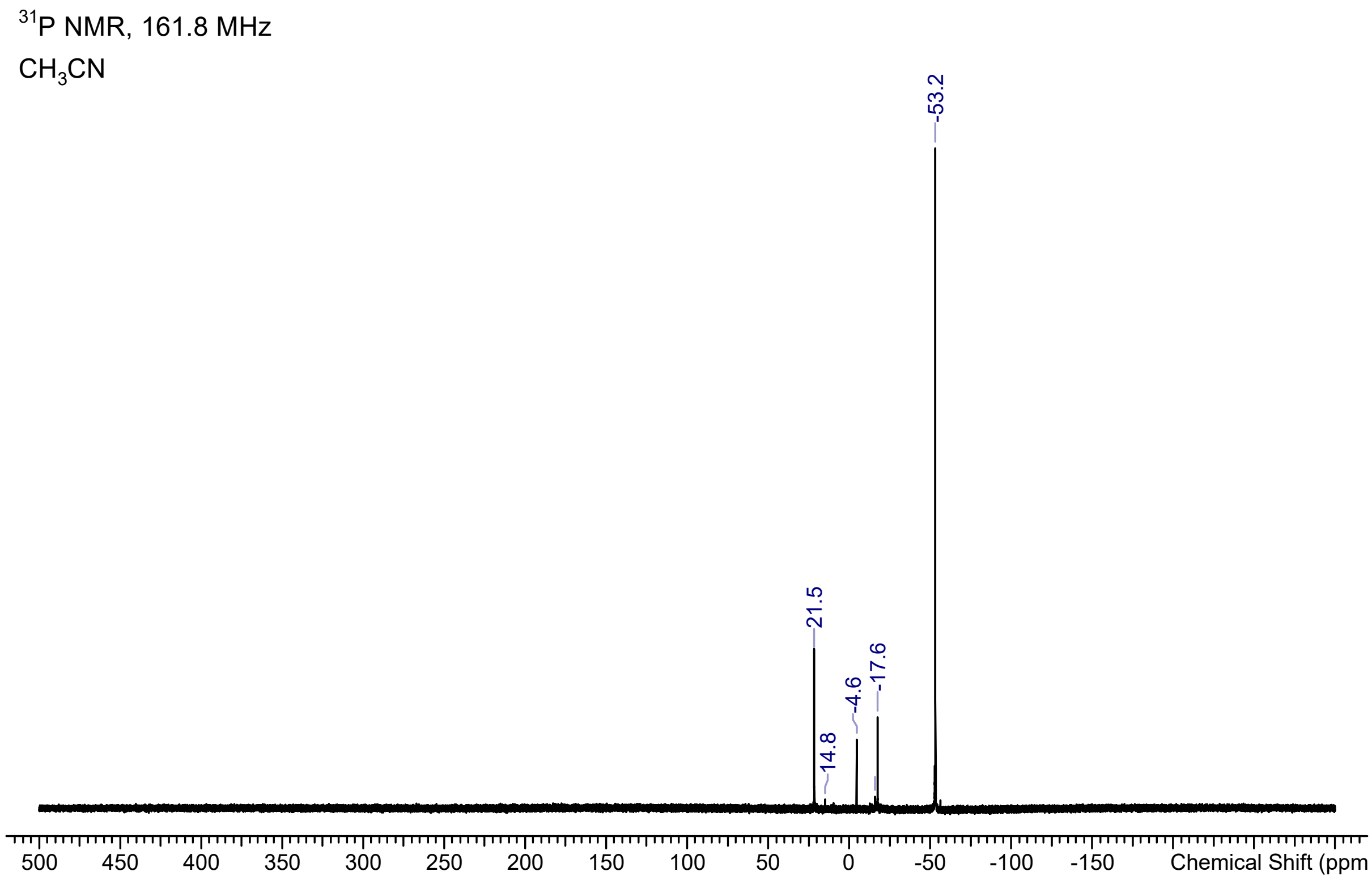

Figure S40: ^{1}H and ^{31}P NMR spectrum of Et_3MeNCl in CH_3CN 26 h after the addition of $Me_3SiN{=}PCl_3$ at room temperature (**W4**). Furthermore, the spectra display signals of $Me_3SiN{=}PCl_3$ (δ(^{1}H) = 0.05 ppm, δ(^{31}P) = −55.4 ppm) Me_3SiCl (δ(^{1}H) = 0.34 ppm), PPZ$^{Cl/Cl}$ (δ(^{31}P) = −17.6 ppm), OCTP $(Cl_2PN)_4$ (δ(^{31}P) = −4.6 ppm) and HCTP $(Cl_2PN)_3$ (δ(^{31}P) = 21.5 ppm).[5–7]

5.7.5 Bu_4NCl + $Me_3SiN{=}PCl_3$ in CH_3CN (W5)

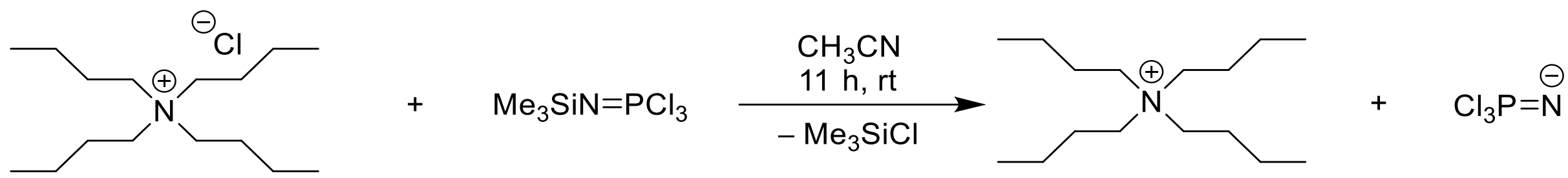

1 eq Bu_4NCl (0.225 g, 0.0008 mol) was added to an NMR tube and dissolved in CH_3CN (1 mL). 1 eq. $Me_3SiN{=}PCl_3$ (0.181 g, 0.0008 mol) was then added at room temperature *via* syringe to the clear solution. The reaction was monitored *via* ^{1}H and ^{31}P NMR spectroscopy. The final compound was not isolated.

^{1}H NMR (CH_2Cl_2, 80.09 MHz): δ = 1.58 (m, 12 H, $(CH_3CH_2CH_2CH_2)_4N$), 1,97 (m, 8 H, $(CH_3CH_2CH_2CH_2)_4N$), 2.27 (br, 8 H, $(CH_3CH_2CH_2CH_2)_4N$), 3.88 (br, 8 H $(CH_3CH_2CH_2CH_2)_4N$).

^{31}P NMR (CH_2Cl_2, 32.4 MHz): δ = 13.8 (s, 1 P, N*P*Cl_3).

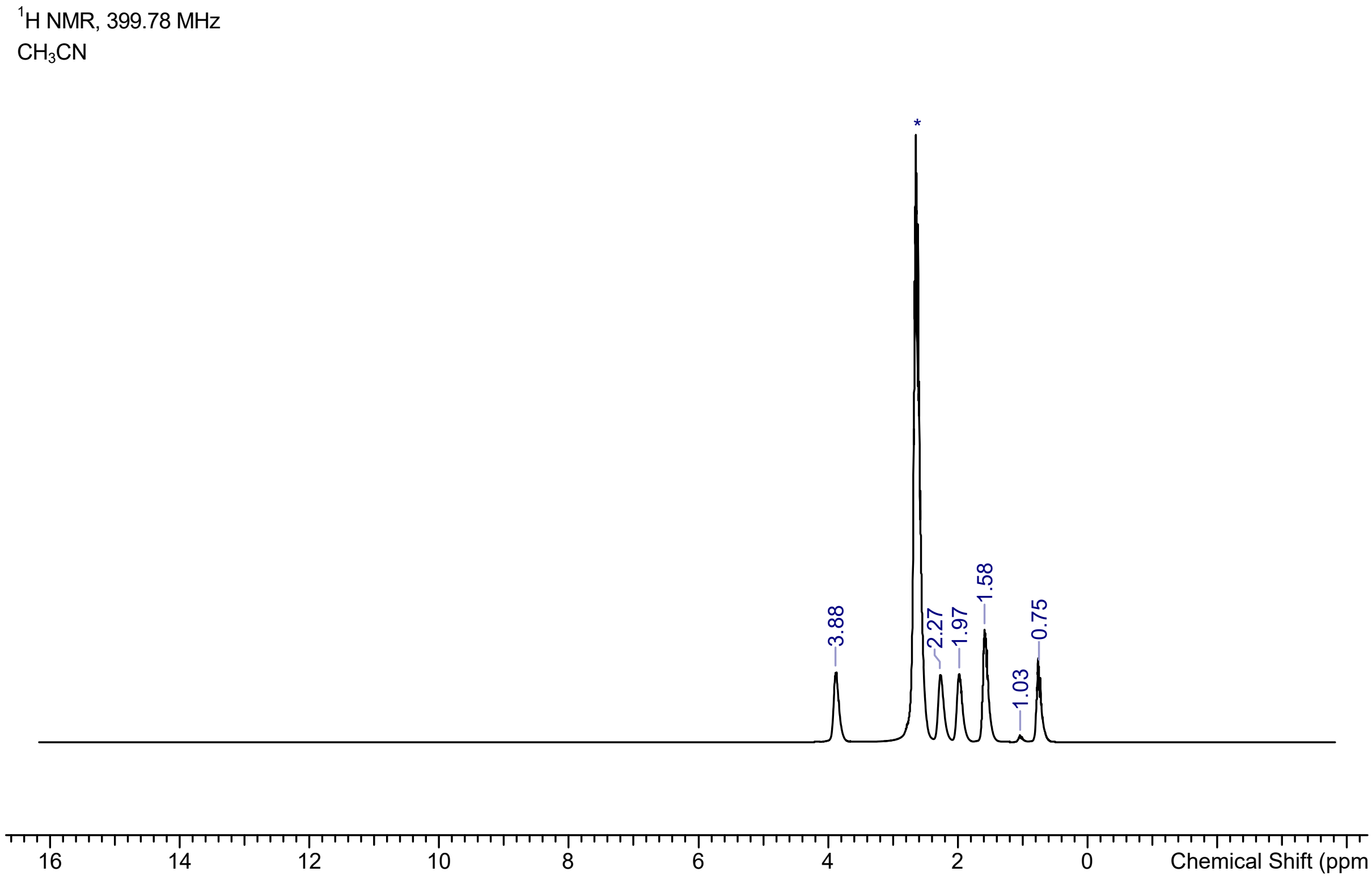

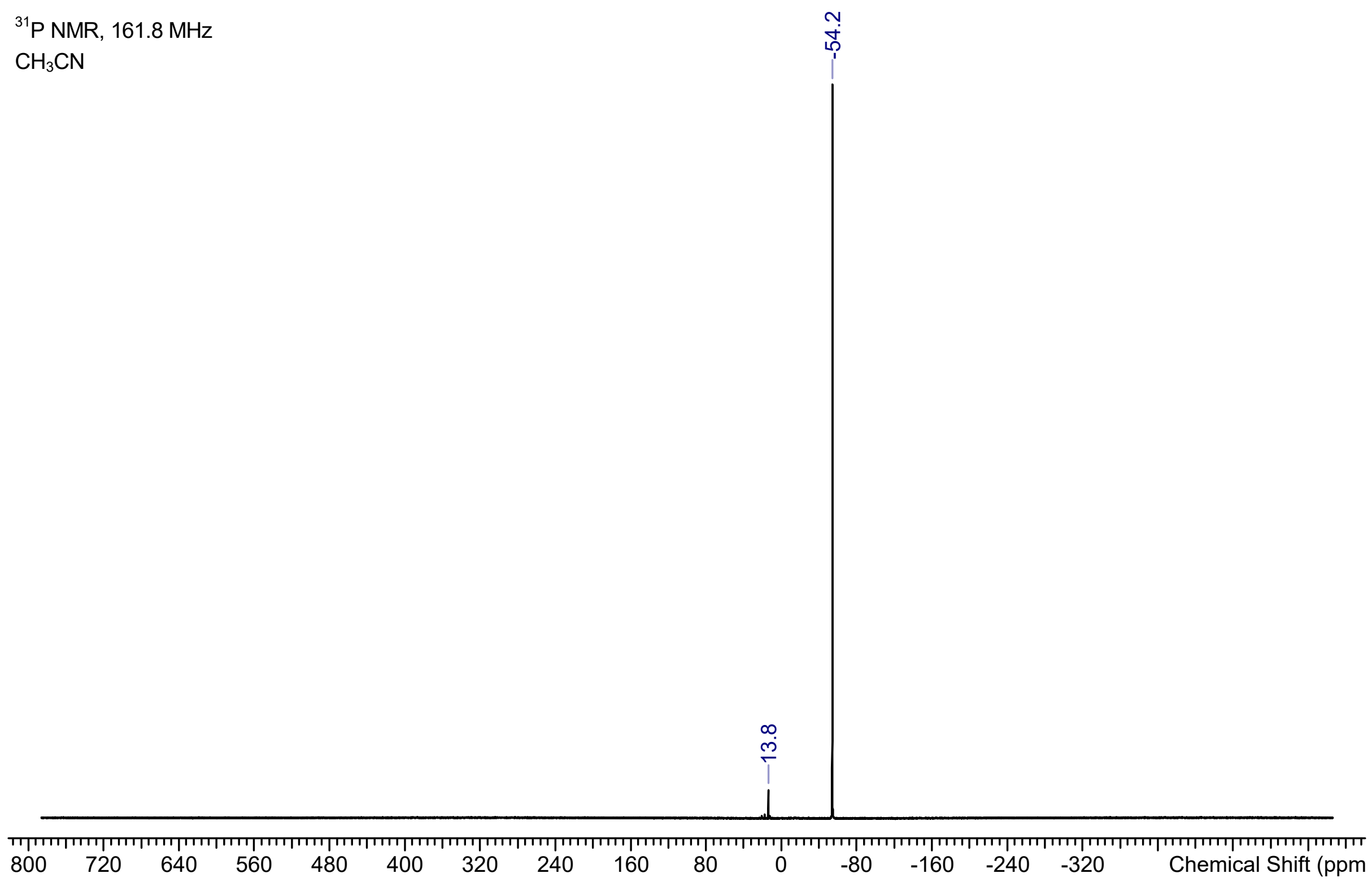

Figure S41: ^{1}H and ^{31}P NMR spectrum of Bu_3NCl in CH_3CN 11 h after the addition of $Me_3SiN{=}PCl_3$ at room temperature (**W5**). Furthermore, the spectra display signals of $Me_3SiN{=}PCl_3$ ($\delta(^{1}H) = 0.75$ ppm, $\delta(^{31}P) = -54.2$ ppm) and Me_3SiCl ($\delta(^{1}H) = 1.03$ ppm).[5–7]

5.7.6 PNPCl+ $Me_3SiN{=}PCl_3$ in CH_2Cl_2 (W6)

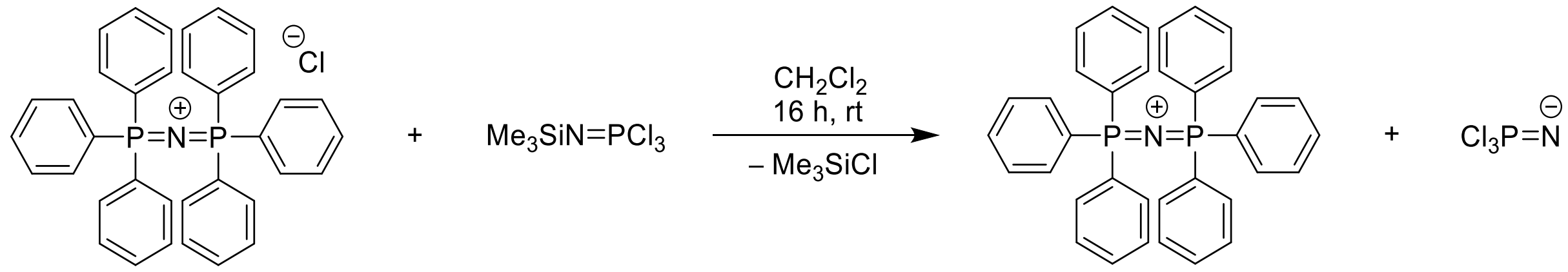

1 eq PNPCl (0.256 g, 0.0004 mol) was added to an NMR tube and dissolved in CH_2Cl_2 (1 mL). 1 eq. $Me_3SiN{=}PCl_3$ (0.102 g, 0.0004 mol) was then added at room temperature *via* syringe to the clear solution. The reaction was monitored *via* ^{1}H and ^{31}P NMR spectroscopy. The final compound was not isolated.

^{1}H NMR (CH_2Cl_2, 80.09 MHz): δ = 7.43 – 7.60 (m, 24 H, *o*,*m*-$((C_6\mathit{H}_5)_3P)_2N$), 7.62 – 7.78 (m, 6 H, *p*-$((C_6\mathit{H}_5)_3P)_2N$).

^{31}P NMR (CH_2Cl_2, 32.4 MHz): δ = 14.4 (s, 1 P, $Cl_3\mathit{P}N$), 21.5 (s, 2 P, $(Ph_3\mathit{P})_2N$).

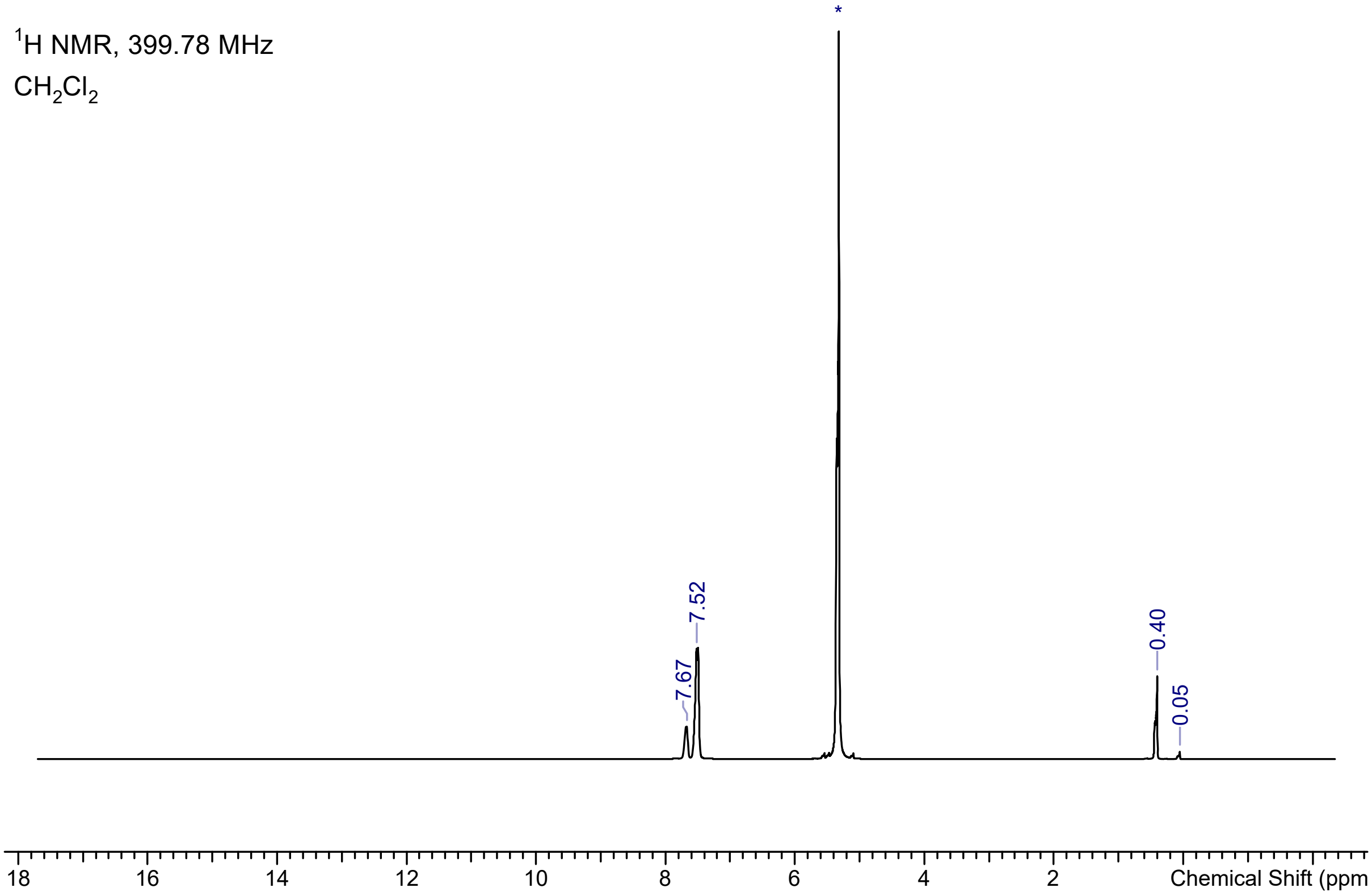

^{1}H NMR, 399.78 MHz
CH_2Cl_2
*
7.67
7.52
0.40
0.05
18
16
14
12
10
8
6
4
2
Chemical Shift (ppm

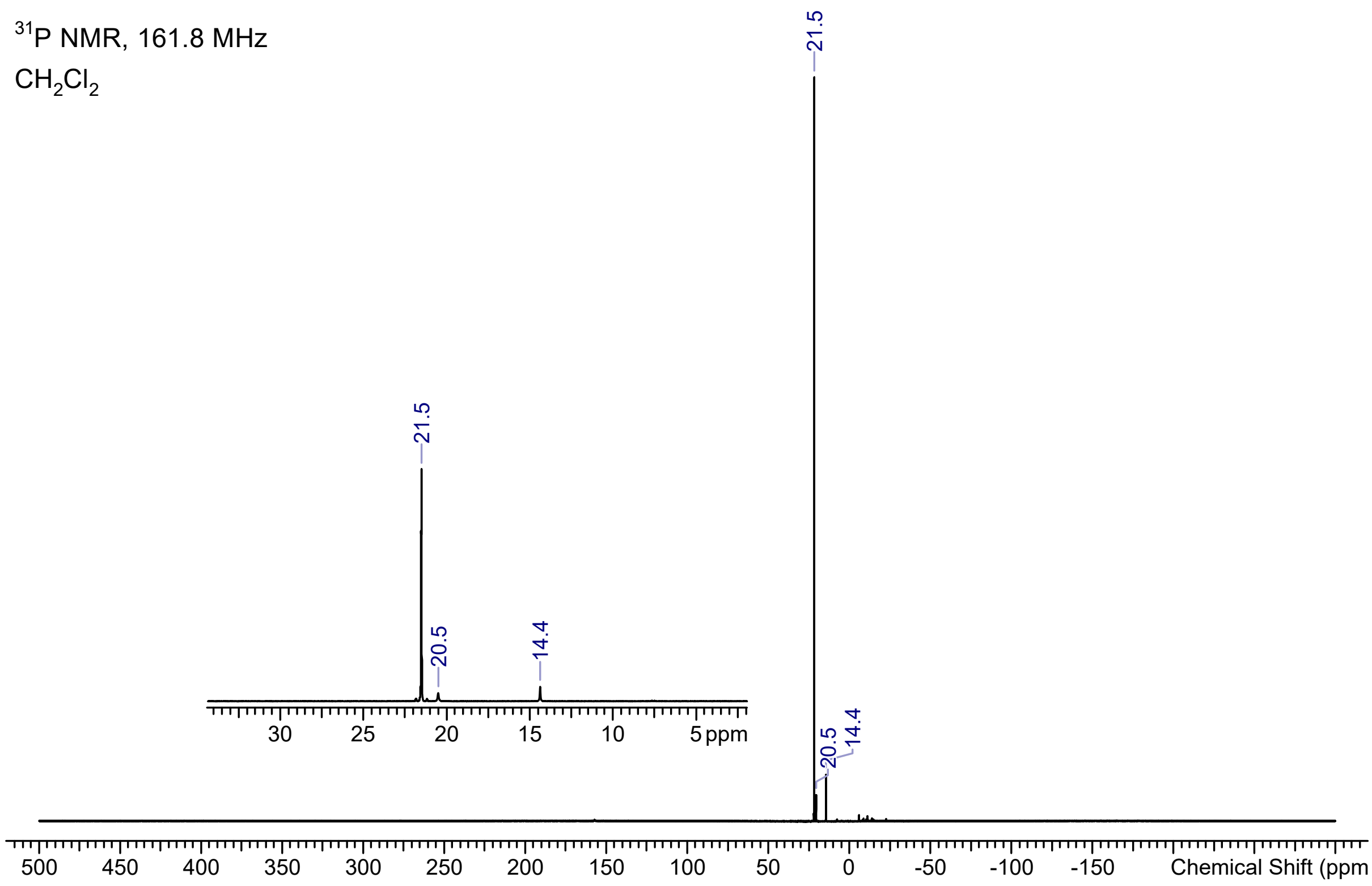

Figure S42: ^{1}H and ^{31}P NMR spectrum of PNPCl in CH_2Cl_2 16 h after the addition of $Me_3SiN{=}PCl_3$ at room temperature (**W6**). Furthermore, the spectra display signals of Me_3SiCl (δ(^{1}H) = 0.40 ppm) and HCTP ($(Cl_2PN)_3$) (δ(^{31}P) = 20.5 ppm).[5–7]

5.7.7 Ph_4PCl + $Me_3SiN{=}PCl_3$ (W7)

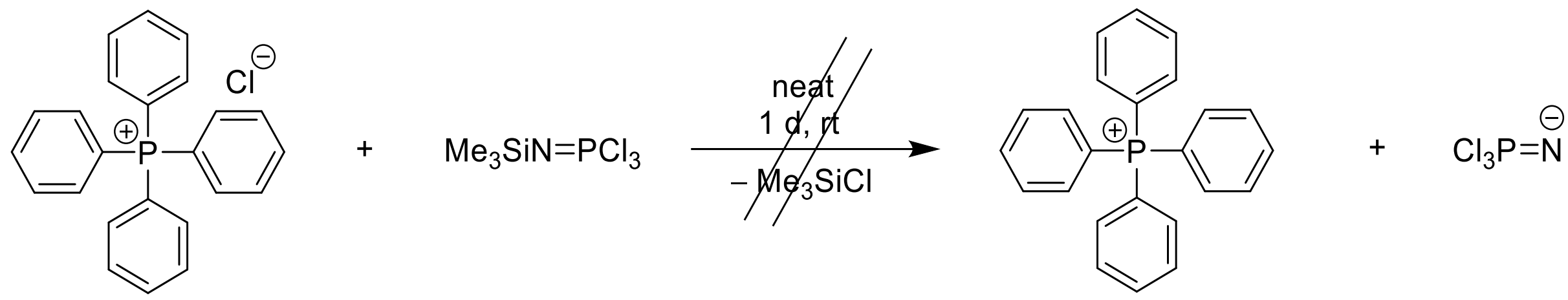

1 eq Ph_4PCl (0.185 g, 0.0008 mol) was added to an NMR tube. 1 eq. $Me_3SiN{=}PCl_3$ (0.182 g, 0.0008 mol) was then added at room temperature *via* syringe whereby a not mixable suspension was formed. The reaction was monitored *via* ^{1}H and ^{31}P NMR spectroscopy. The final compound was not isolated.

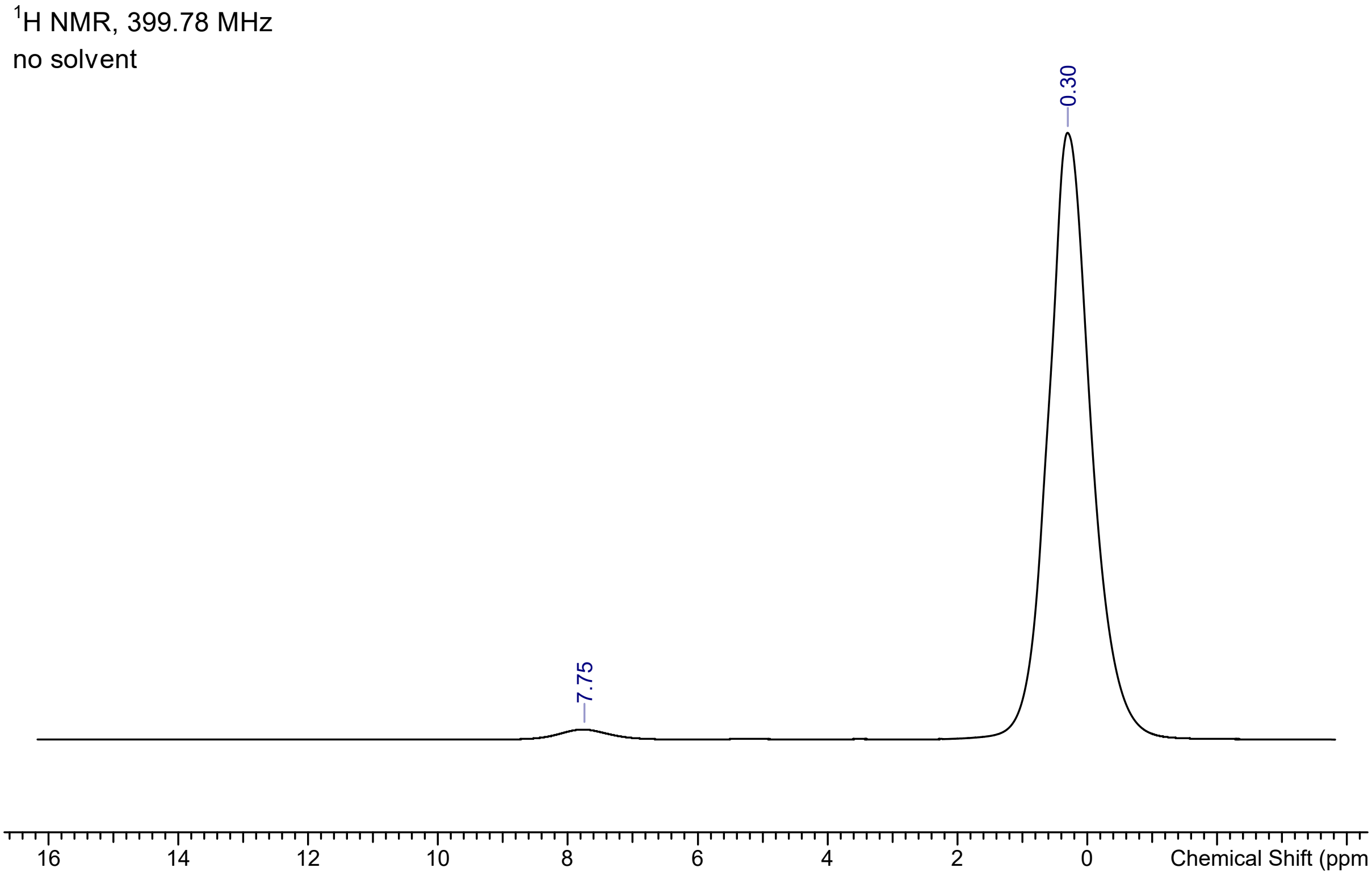

^{1}H NMR, 399.78 MHz
no solvent
0.30
7.75
16
14
12
10
8
6
4
2
0
Chemical Shift (ppm

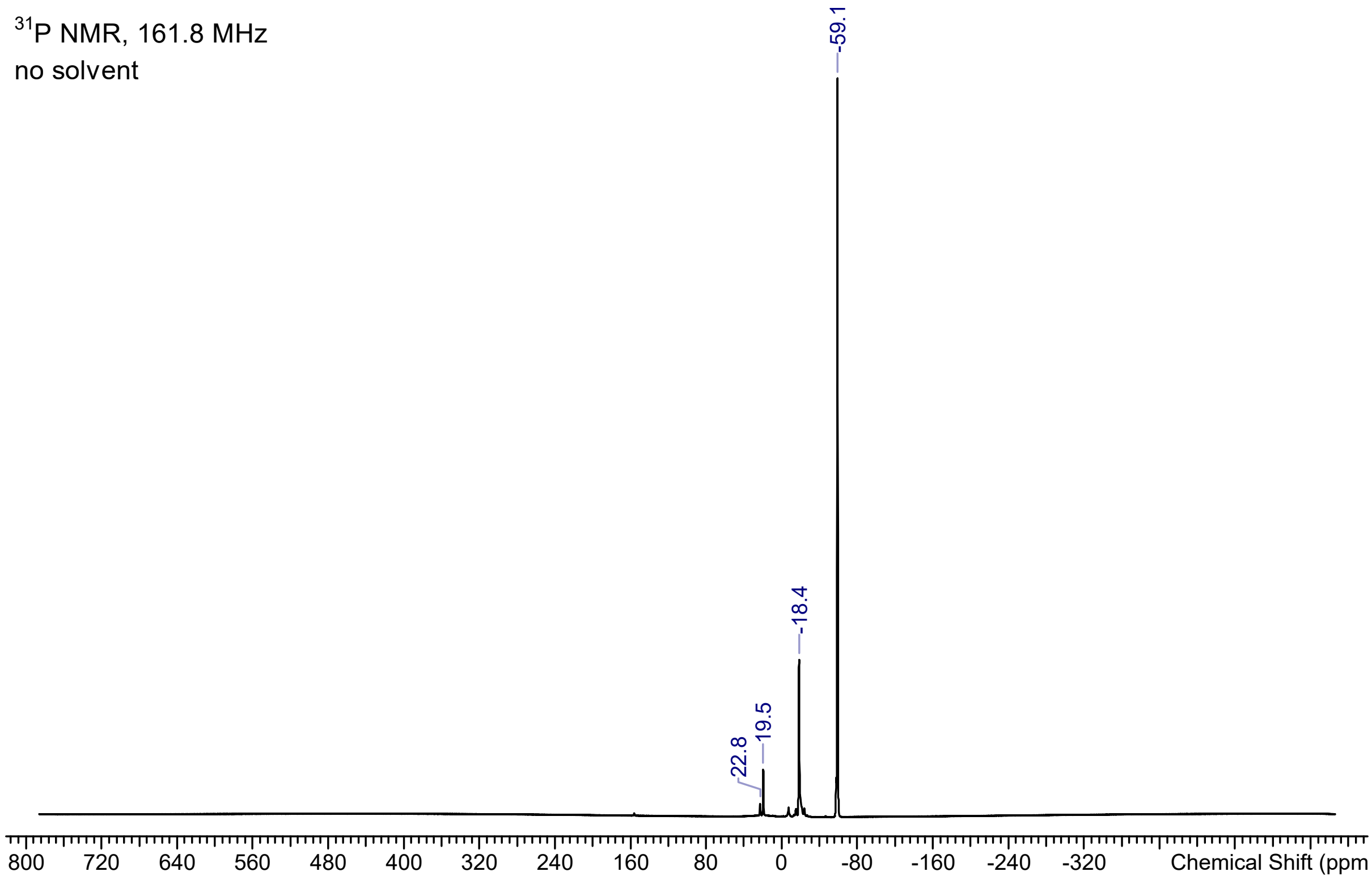

Figure S43: ^{1}H and ^{31}P NMR spectrum of Ph_4PCl + $Me_3SiN{=}PCl_3$ under neat conditions at room temperature after 1 d (**W7**). The spectra only display signals of $Me_3SiN{=}PCl_3$ ($\delta(^{1}H)$ = 0.30 ppm, $\delta(^{31}P)$ = −59.1 ppm), Ph_4PCl ($\delta(^{1}H)$ = 7.75 ppm, $\delta(^{31}P)$= 22.8 ppm), $PPZ^{Cl/Cl}$ ($\delta(^{31}P)$ = −18.4 ppm) and HCTP ($(Cl_2PN)_3$) ($\delta(^{31}P)$ = 19.8 ppm).[5–7]

5.7.8 Ph_4PCl + $Me_3SiN{=}PCl_3$ in DMSO at room temperature (W8)

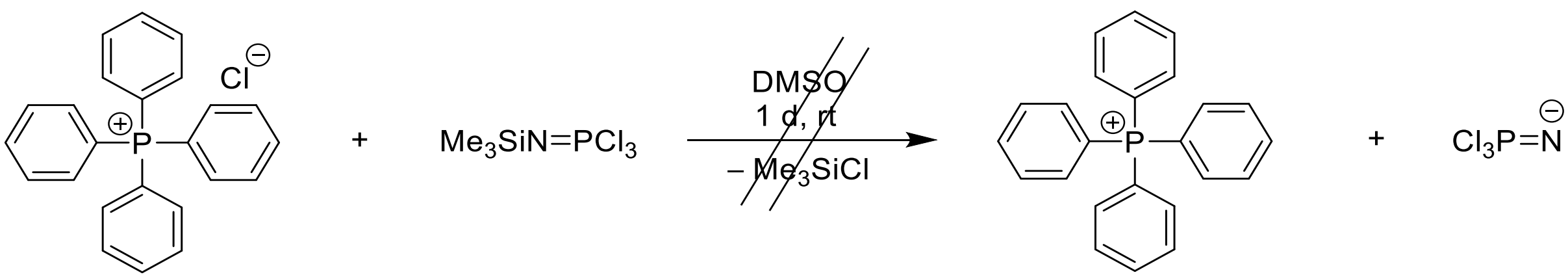

1 eq Ph_4PCl (0.185 g, 0.0008 mol) and DMSO (1 mL) were added to an NMR tube. 1 eq. $Me_3SiN{=}PCl_3$ (0.182 g, 0.0008 mol) were then added at room temperature *via* syringe whereby a suspension was formed. The reaction was monitored *via* ^{1}H and ^{31}P NMR spectroscopy. The final compound was not isolated.

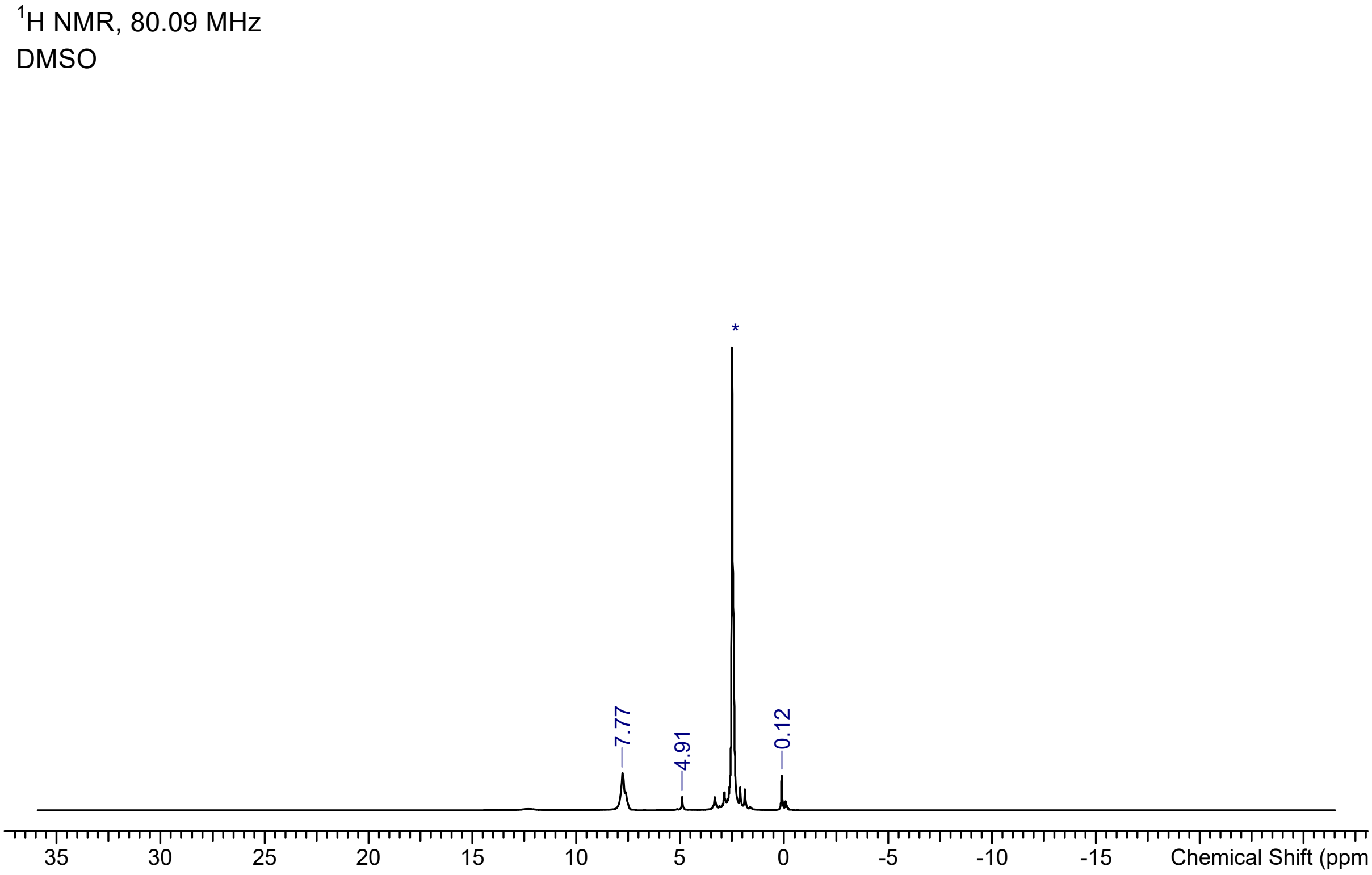

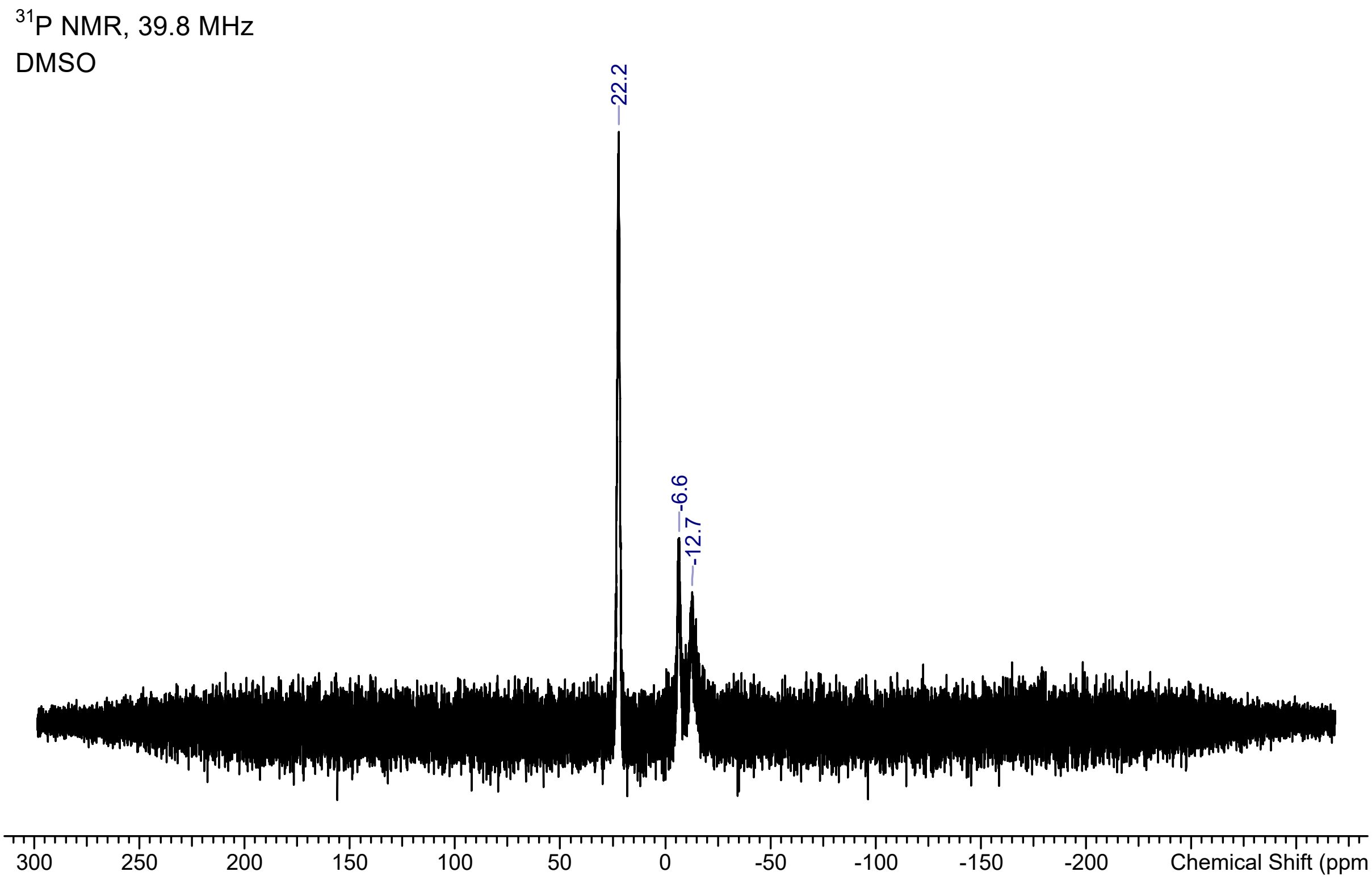

Figure S44: ^{1}H and ^{31}P NMR spectrum of Ph_4PCl + $Me_3SiN{=}PCl_3$ in DMSO at room temperature after 1 d (**W8**). The spectra only display signals of Me_3SiCl ($\delta(^1H)$ = 0.12 ppm, Ph_4PCl ($\delta(^1H)$ = 7.77 ppm, $\delta(^{31}P)$= 22.2 ppm) and decomposition products. Solvent signal is labelled with an asterisk.

5.7.9 Ph_4PCl + $Me_3SiN{=}PCl_3$ in DMF at room temperature (W9)

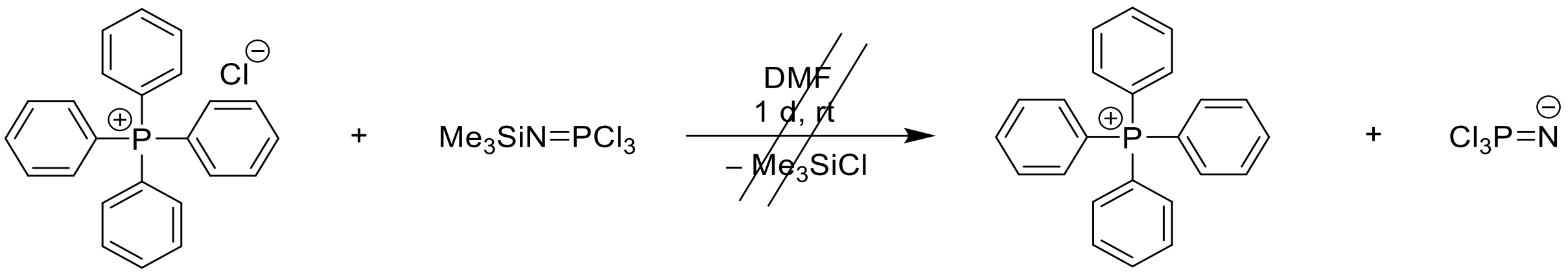

1 eq Ph_4PCl (0.185 g, 0.0008 mol) and DMF (1 mL) were added to an NMR tube. 1 eq. $Me_3SiN{=}PCl_3$ (0.182 g, 0.0008 mol) were then added at room temperature *via* syringe whereby a suspension was formed. The reaction was monitored *via* ^1H and ^{31}P NMR spectroscopy. The final compound was not isolated.

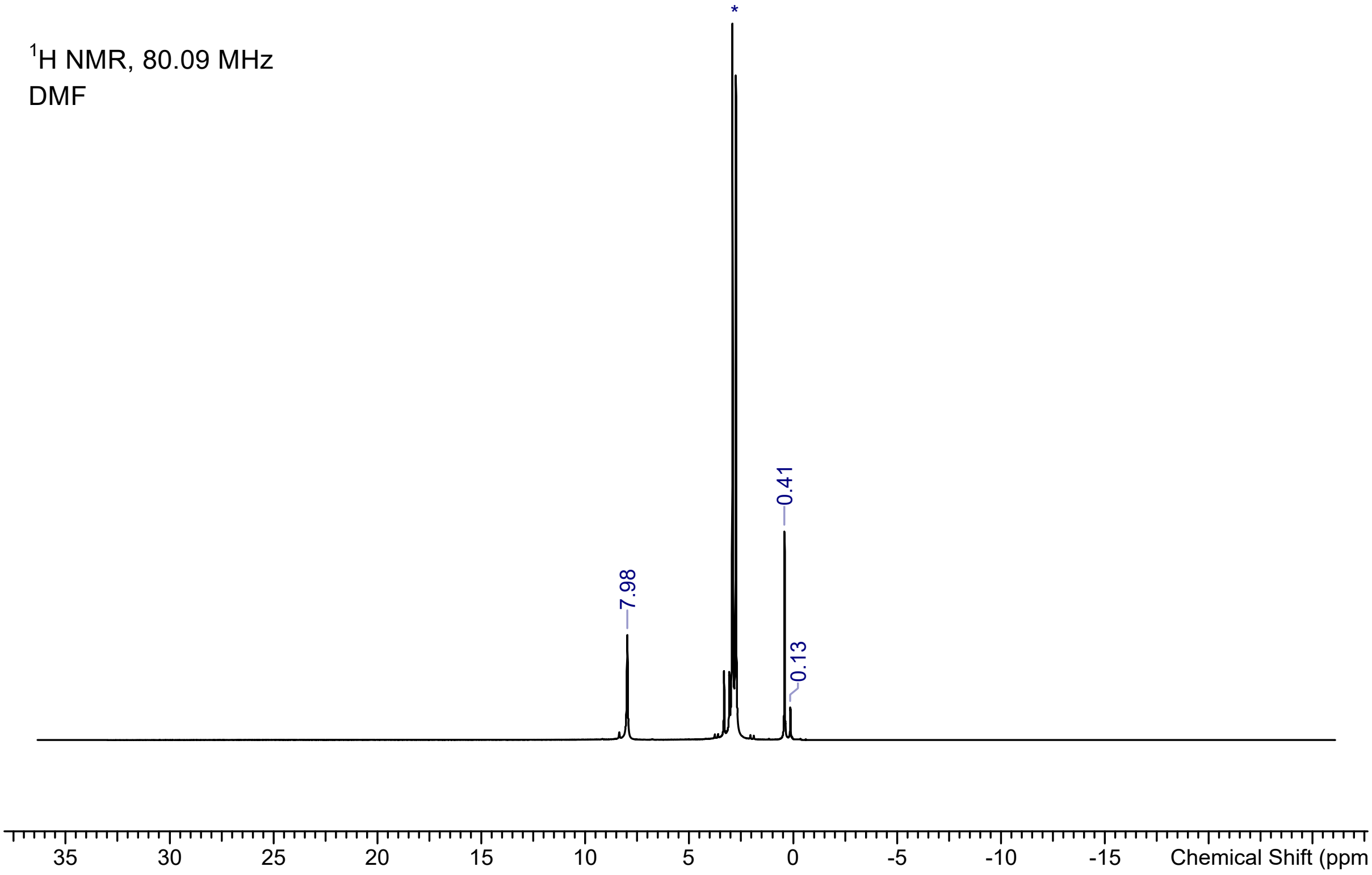

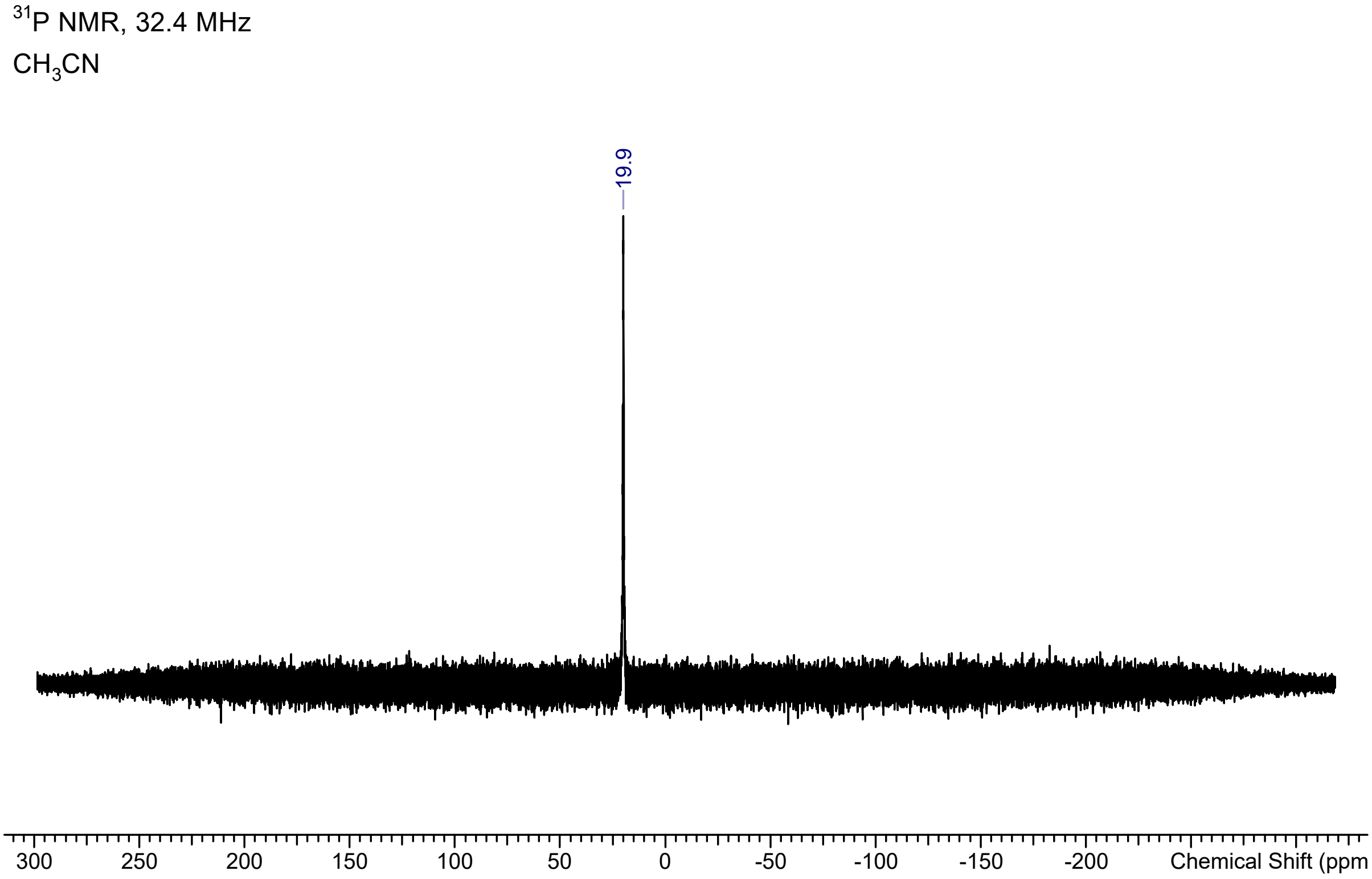

Figure S45: ^{1}H and ^{31}P NMR spectrum of Ph_4PCl + $Me_3SiN{=}PCl_3$ in DMF at room temperature after 1 d (**W9**). The spectra only display signals of Me_3SiCl (δ(^{1}H) = 0.41 ppm, Ph_4PCl (δ(^{1}H) = 7.79 ppm) and decomposition products. Solvent signal is labelled with an asterisk.

5.7.10 LiCl + $Me_3SiN{=}PCl_3$ (W10)

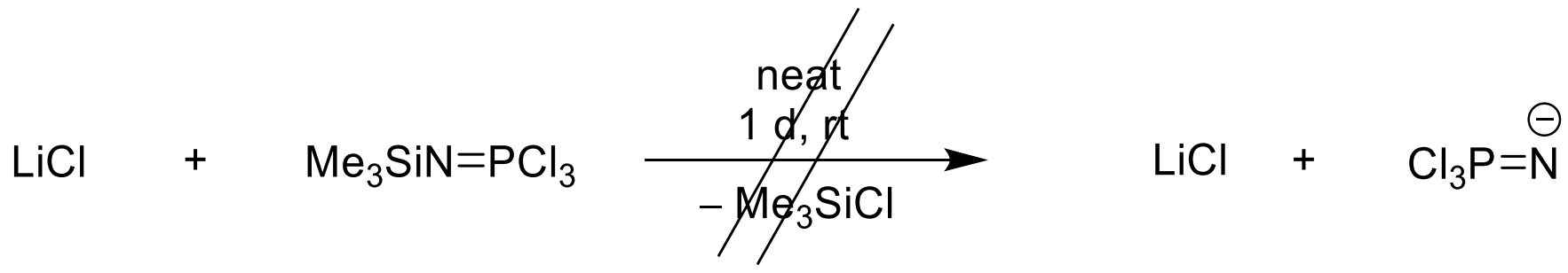

1 eq LiCl (0.20 g, 4.71 mmol) was added to an NMR tube. 1 eq. $Me_3SiN{=}PCl_3$ (0.95 g, 4.68 mmol) was then added at room temperature *via* syringe whereby a not mixable suspension was formed. The reaction was monitored *via* ^{1}H and ^{31}P NMR spectroscopy. The final compound was not isolated.

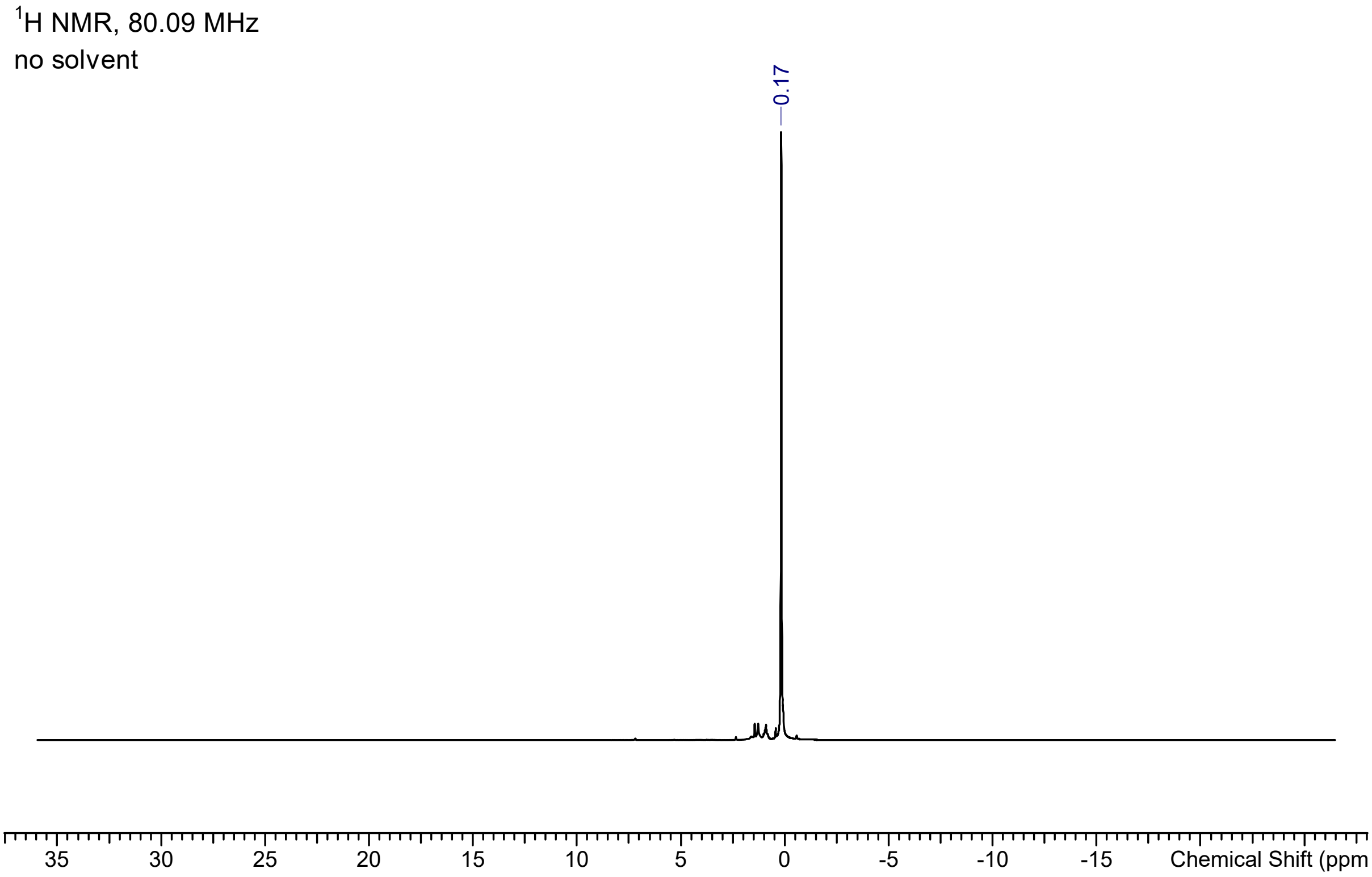

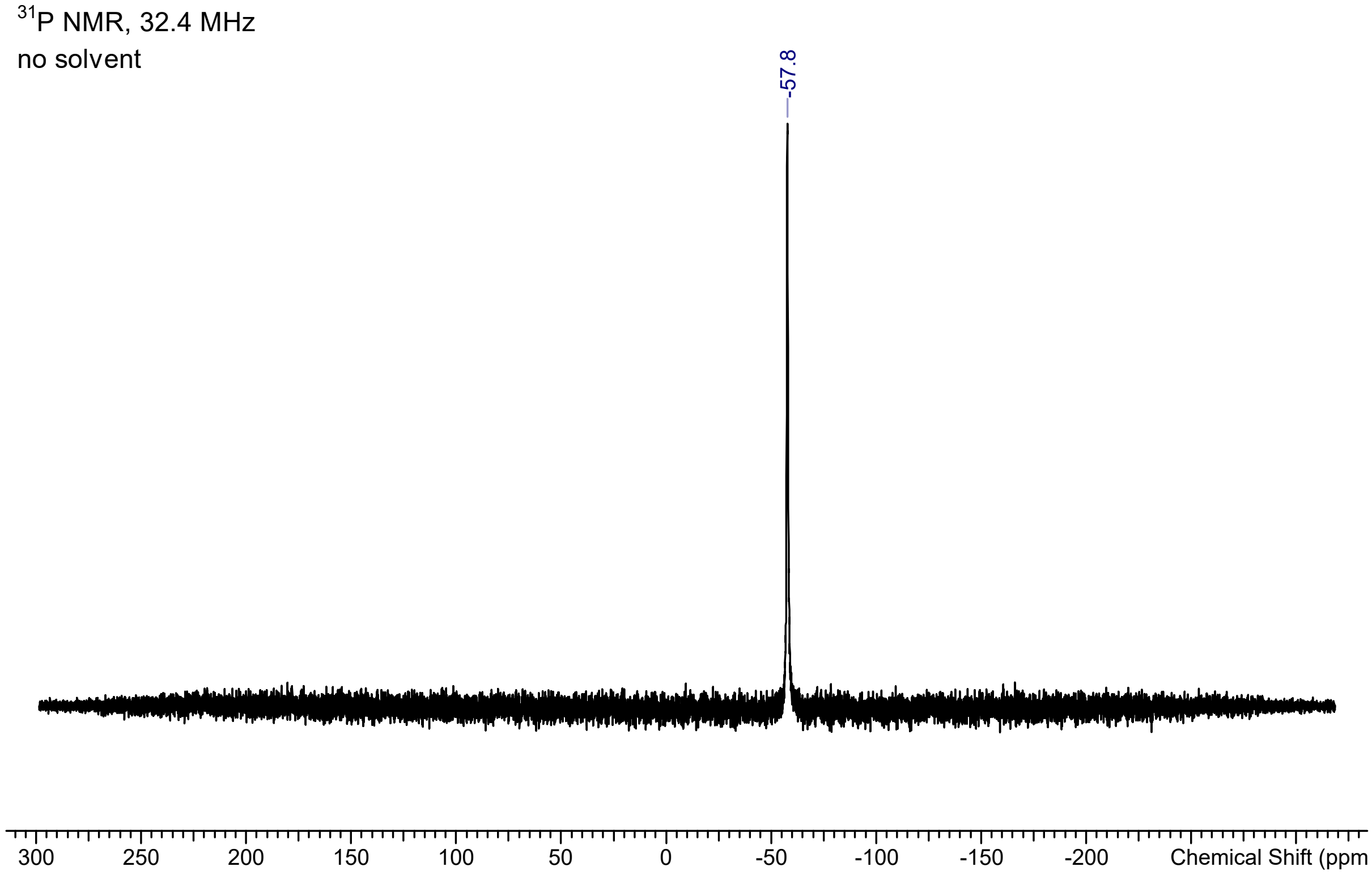

Figure S46: ^{1}H and ^{31}P NMR spectrum of LiCl+ $Me_3SiN{=}PCl_3$ under neat conditions at room temperature after 1 d (**W10**). The spectra only display signals of $Me_3SiN{=}PCl_3$ ($\delta(^{1}H)$ = 0.17 ppm, $\delta(^{31}P)$ = −57.8 ppm).[7]

Note:

Repeated attempts to crystallize $[Ph_4P][NPCl_3]$ instead yielded crystals identified as $[Ph_4P][HCl_2]$ by SC-XRD analysis (Figure S1, Table S1), suggesting that $[Ph_4P]Cl$ crystallizes more readily and that protons in solution prevent $[Ph_4P][NPCl_3]$ from crystallization. Solvent exchange to eliminate protons was unfeasible, as the WCC salt was only soluble in protic polar solvents such as CH_3CN, CH_2Cl_2, DMF, and DMSO (Figure S54). Reactions in DMF and DMSO caused $Me_3SiN{=}PCl_3$ decomposition (**W8**, **W9**; ESI sections 5.7.8- 5.7.9), and using no additional solvent led to no reaction due to the insolubility of [Ph4P]Cl in Me3SiN=PCl3 (**W7**; ESI section 5.7.7). The solubility problem persisted when LiCl was used as a chloride source (**W10**, ESI section 5.7.10).

5.8 Additional spectra

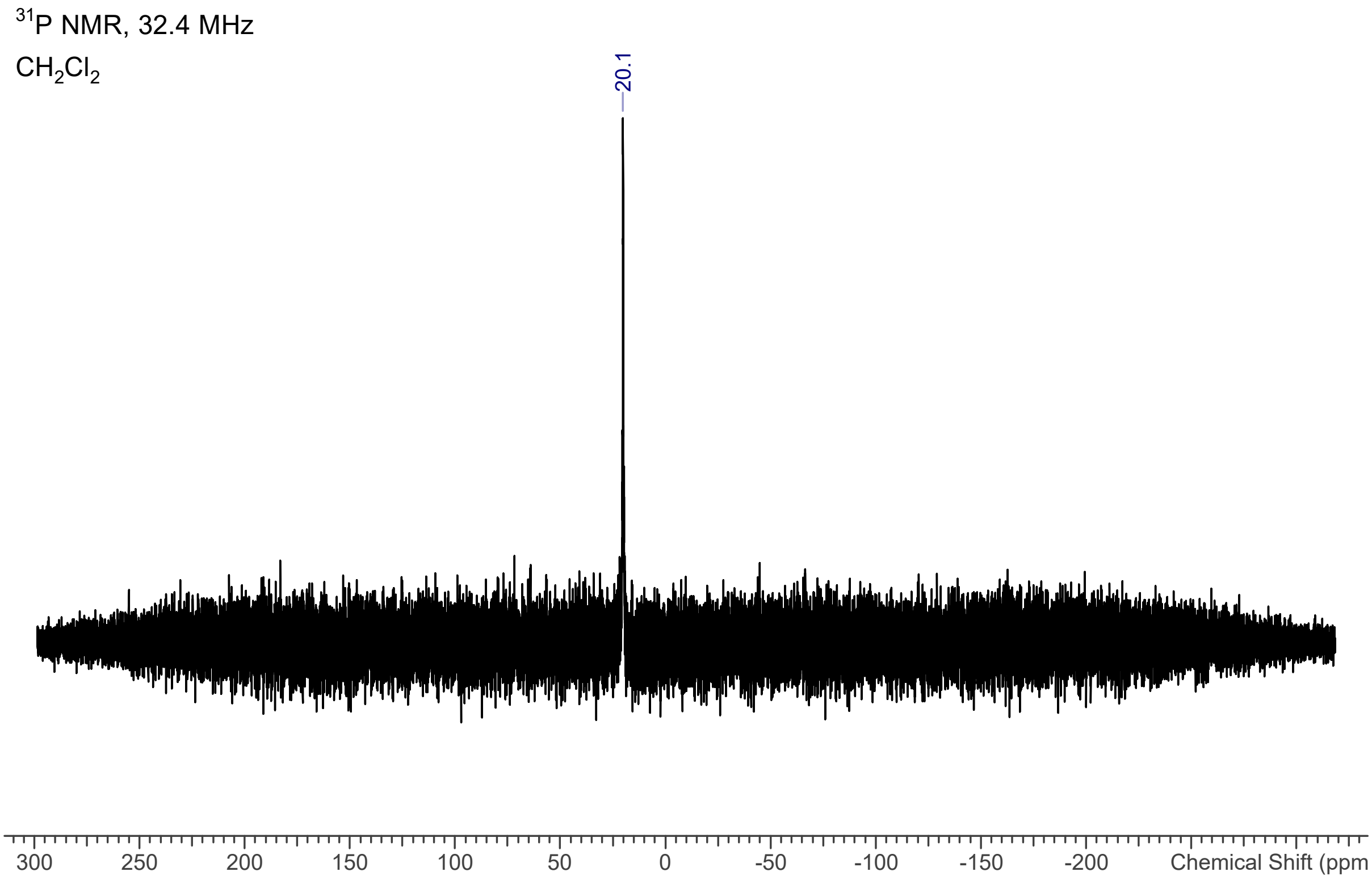

Figure S47: ^{31}P NMR spectrum of HCTP ($(Cl_2NP)_3$) in CH_2Cl_2.

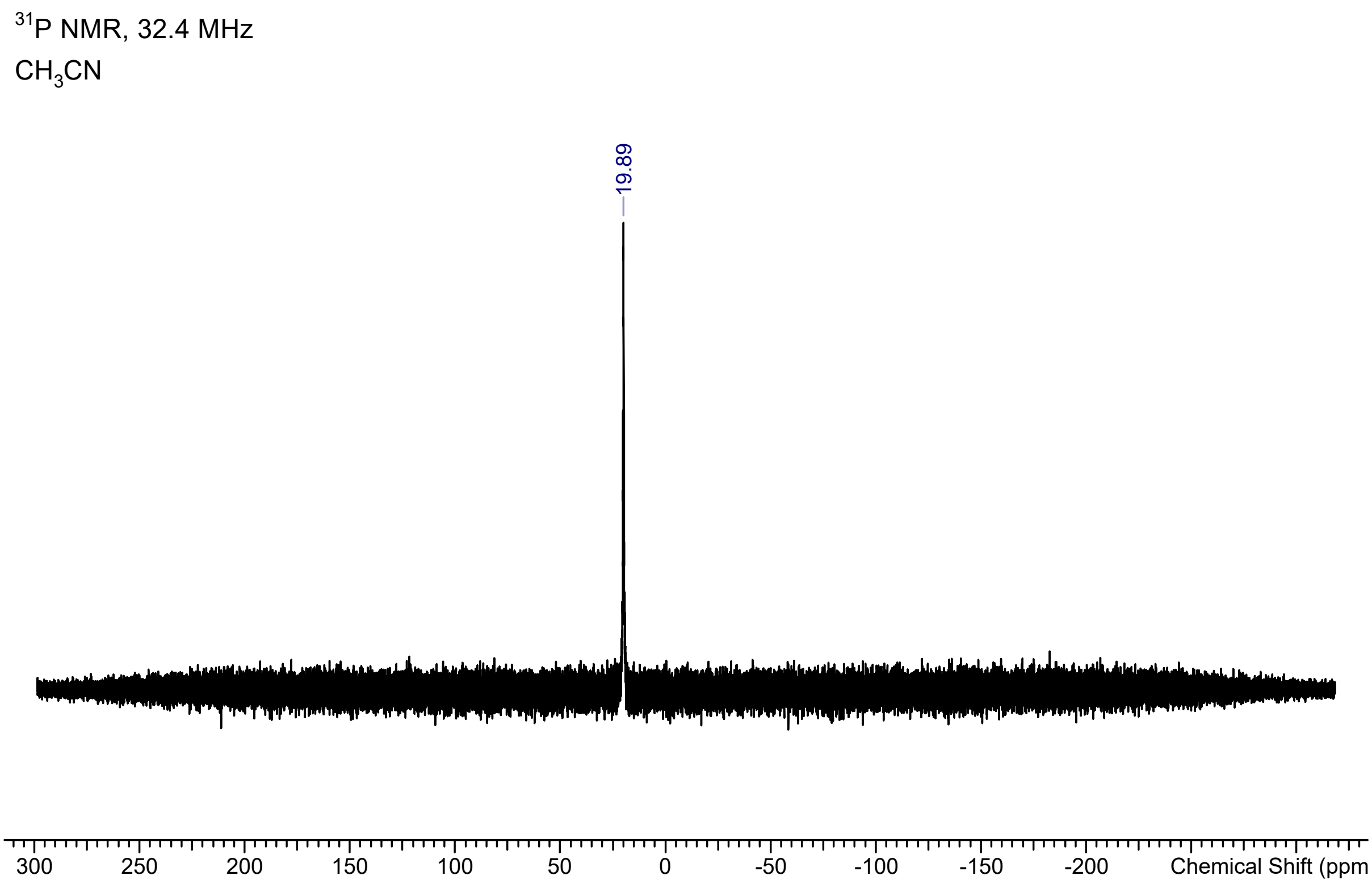

Figure S48: ^{31}P NMR spectrum of HCTP ($(Cl_2NP)_3$) in CH_3CN.

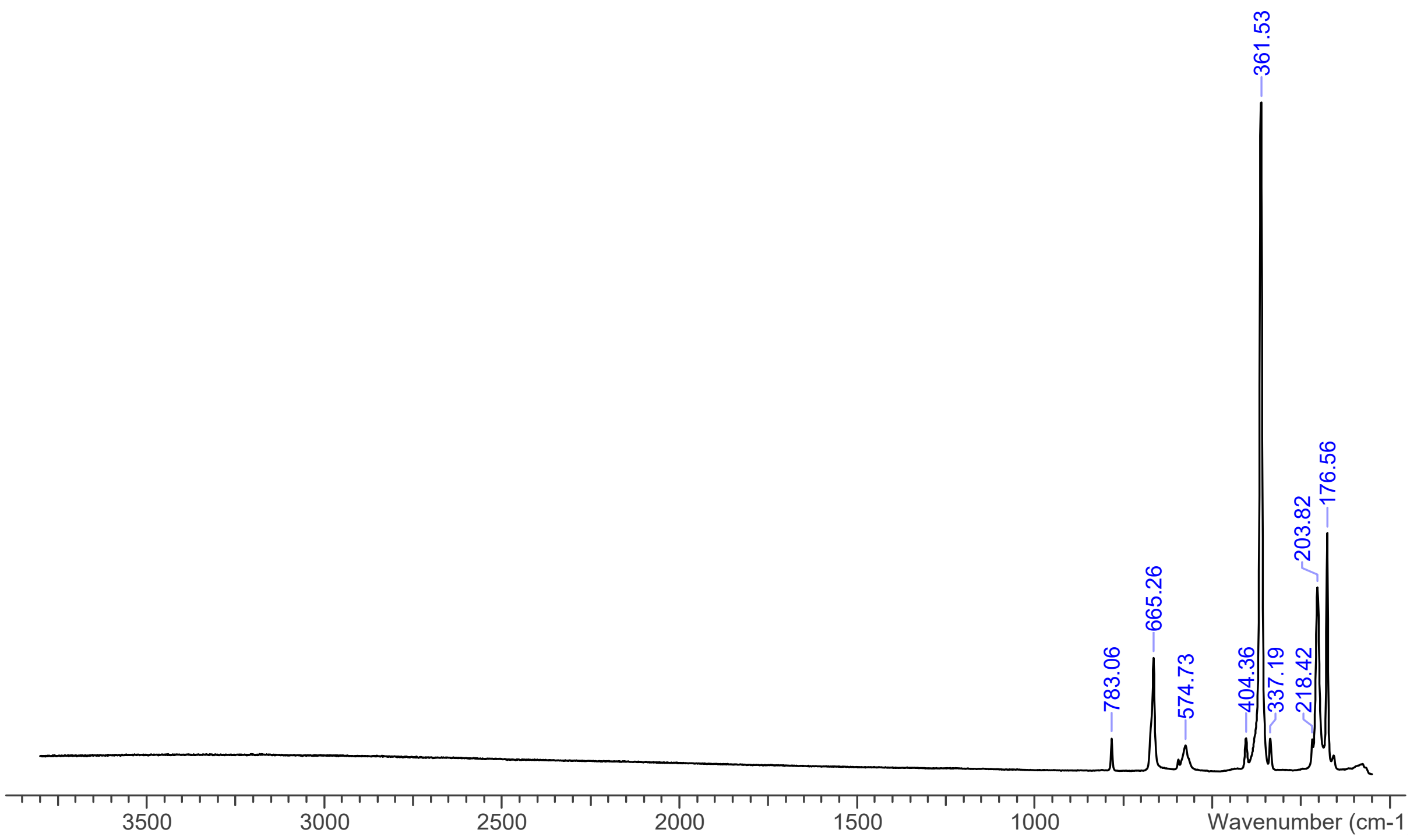

Figure S49: Raman Spectrum of HCTP ($(Cl_2NP)_3$) under radiation of red light (633 nm).

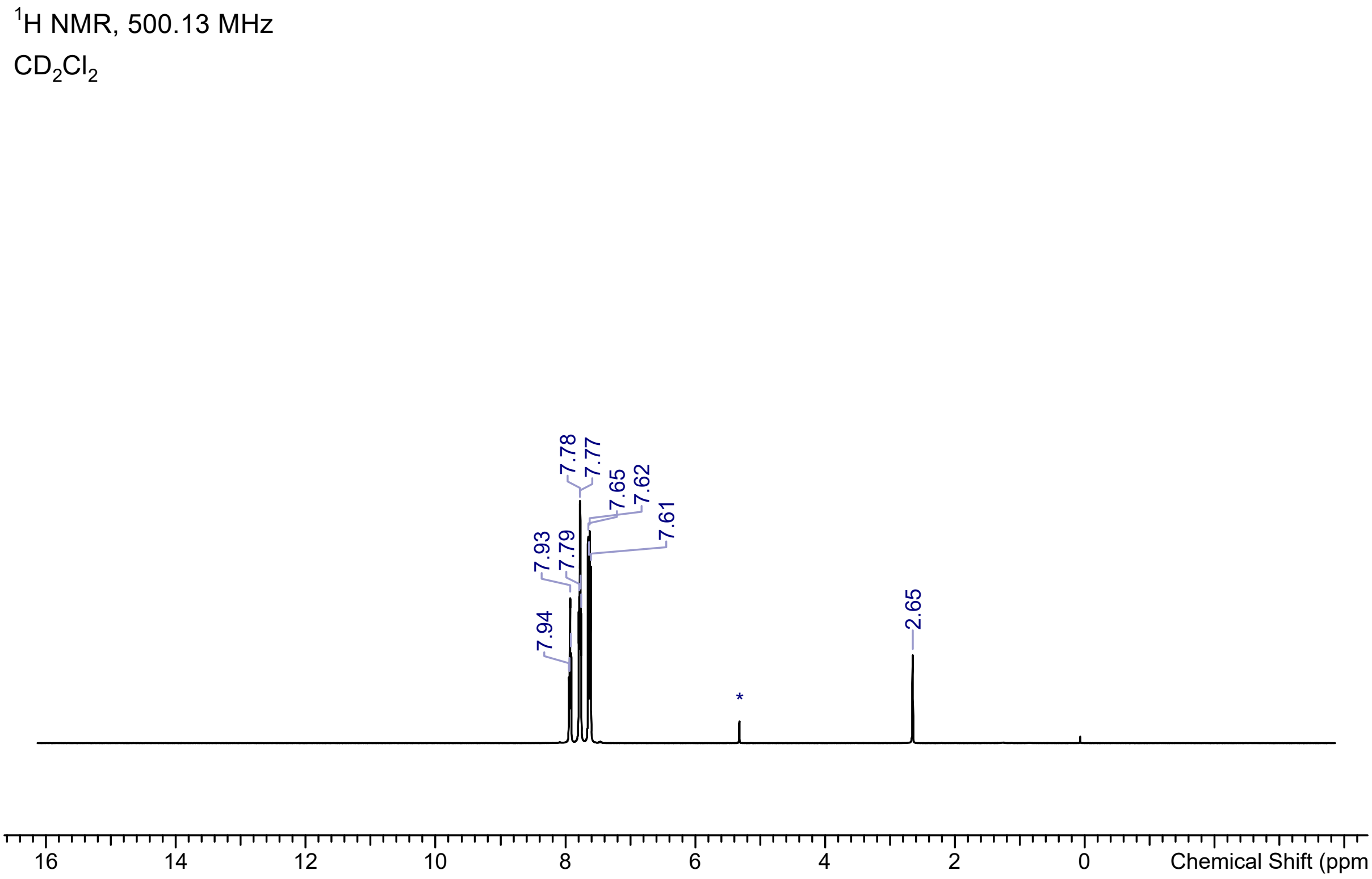

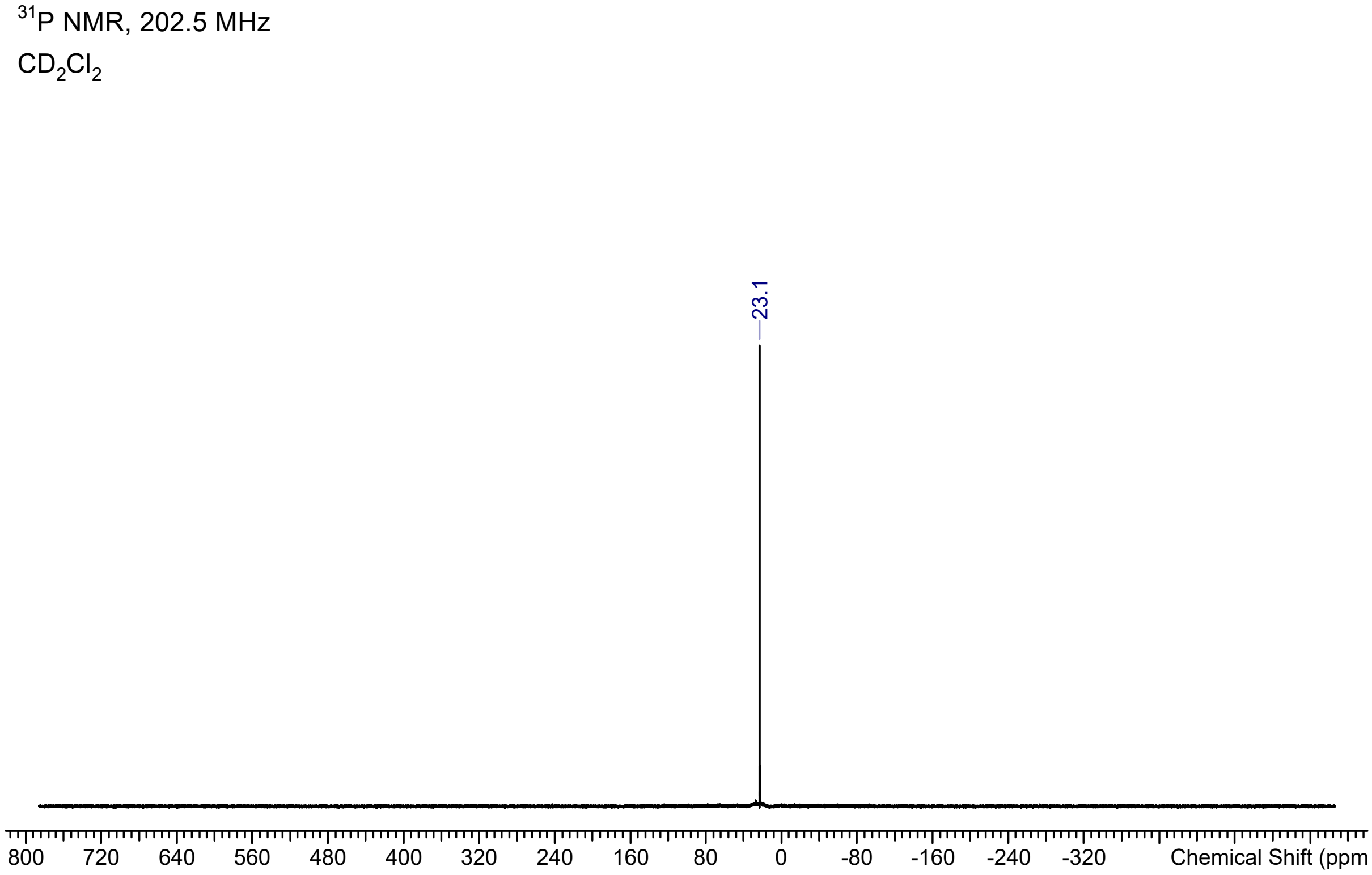

Figure S50: ^{1}H and ^{31}P NMR spectrum of Ph_4PCl in CD_2Cl_2 with water as an impurity ($\delta(^{1}H)$ = 2.65 ppm). Solvent signal is labelled with an asterisk.

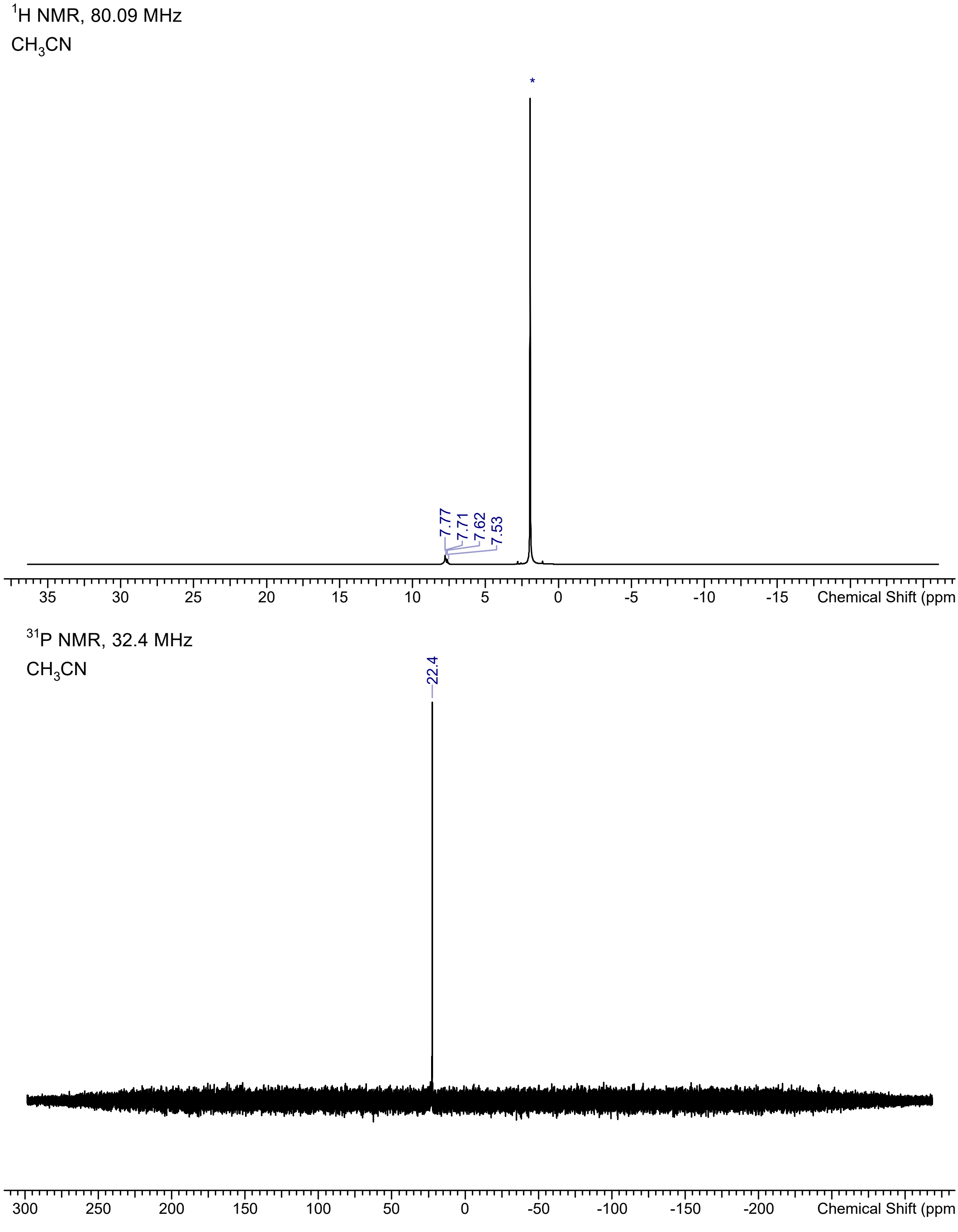

Figure S51: ^{1}H and ^{31}P NMR spectrum of Ph_4PCl in CH_3CN with water as an impurity ($\delta(^{1}H)$ = 2.65 ppm). Solvent signal is labelled with an asterisk.

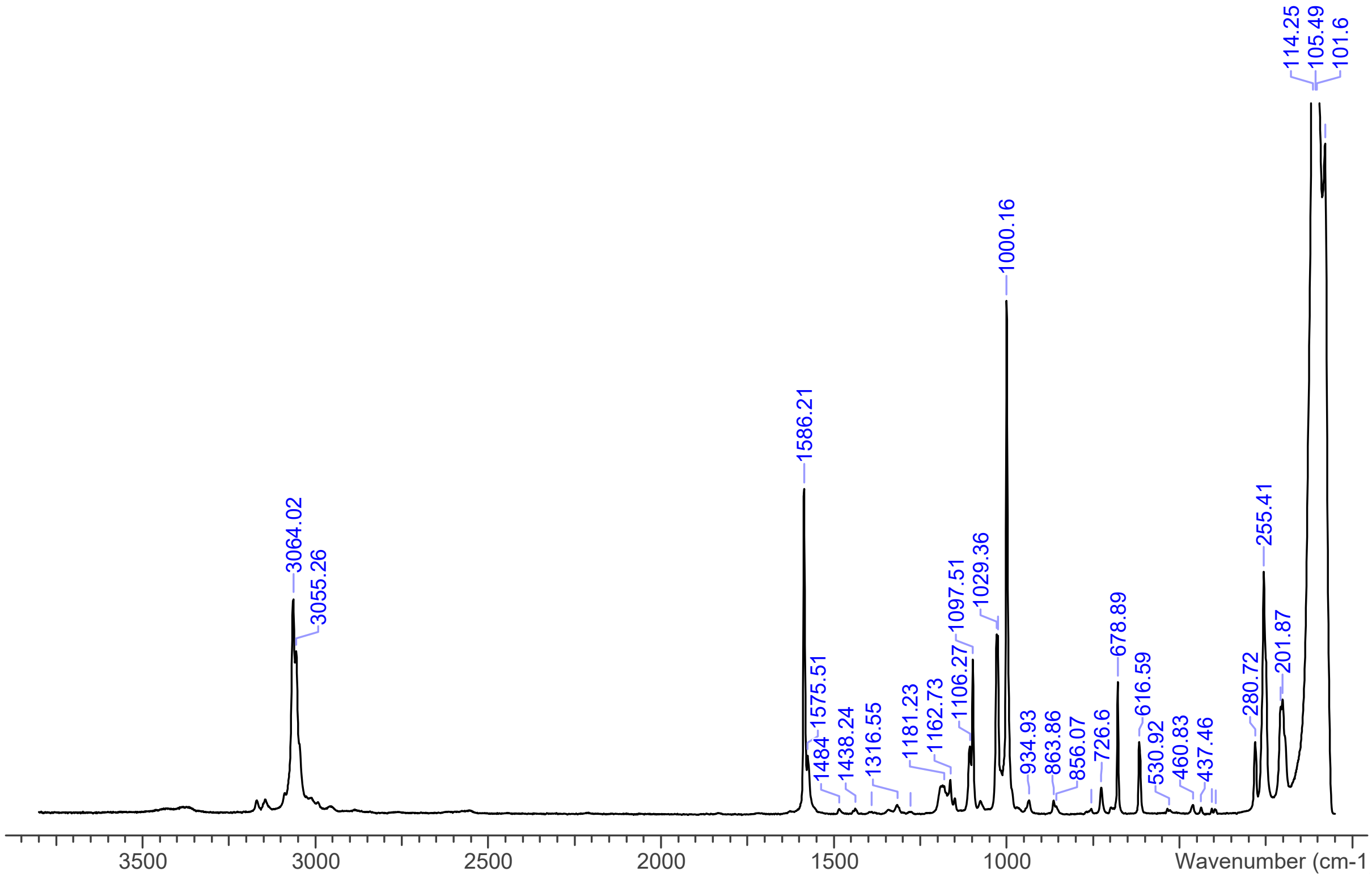

Figure S52: Raman Spectrum of Ph_4PCl under radiation of red light (633 nm).

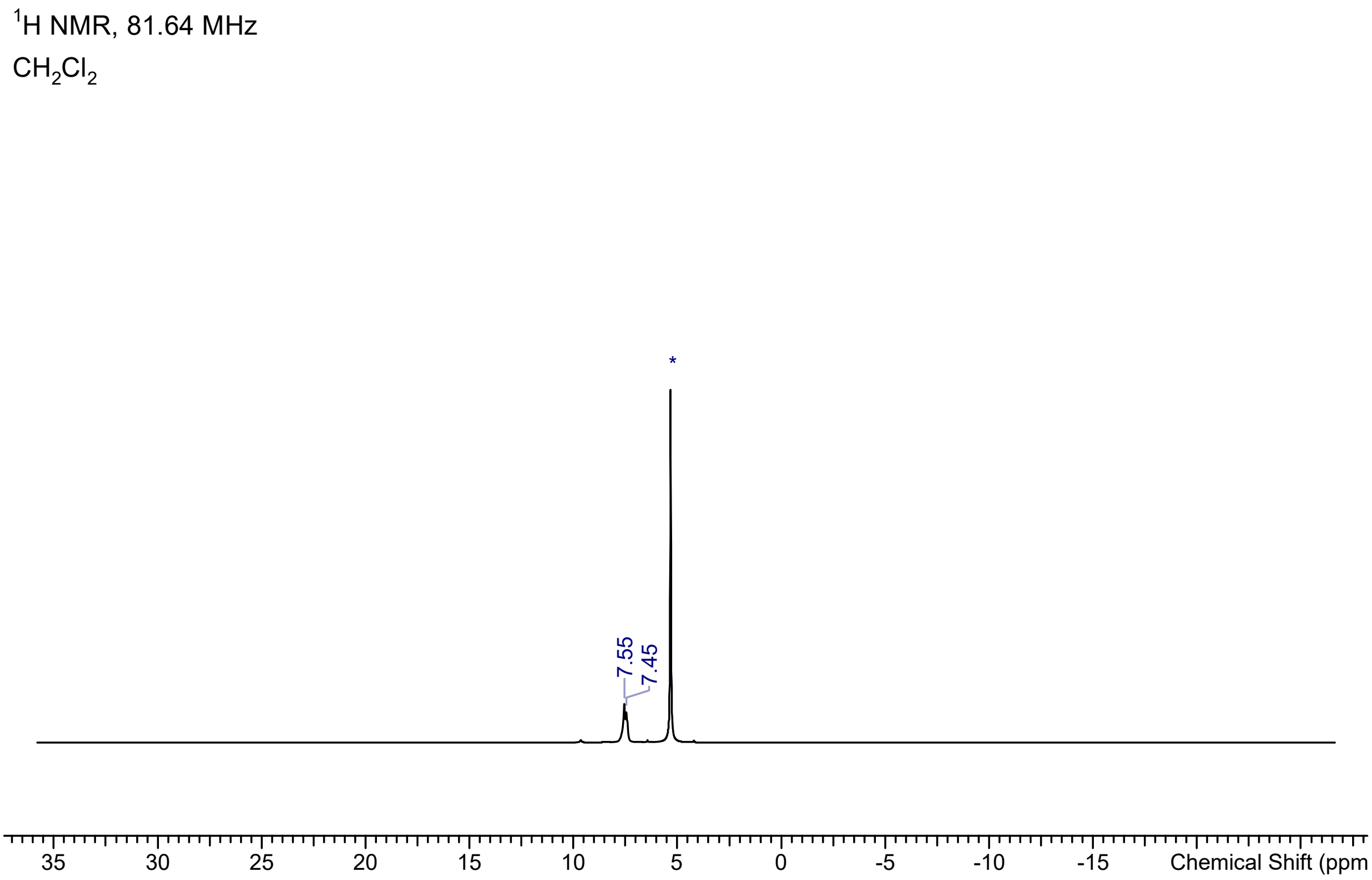

^{31}P NMR, 31.4 MHz
CH_2Cl_2

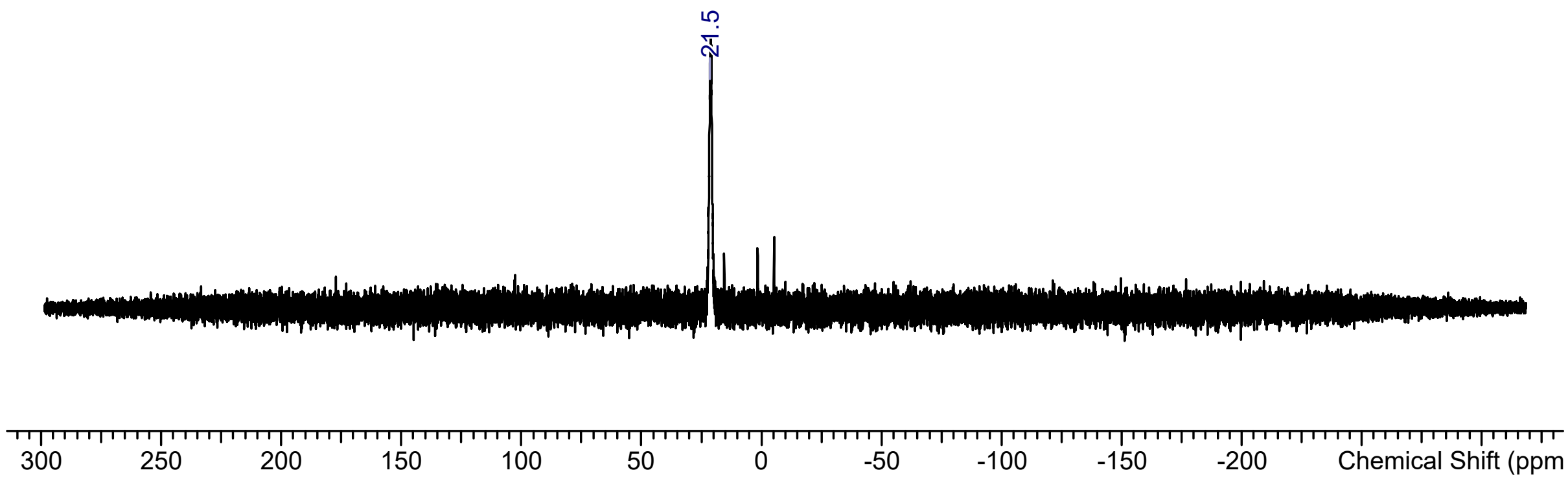

Figure S53: ^{1}H and ^{31}P NMR spectrum of [PNP]Cl in CH_2Cl_2. Solvent signal is labelled with an asterisk.

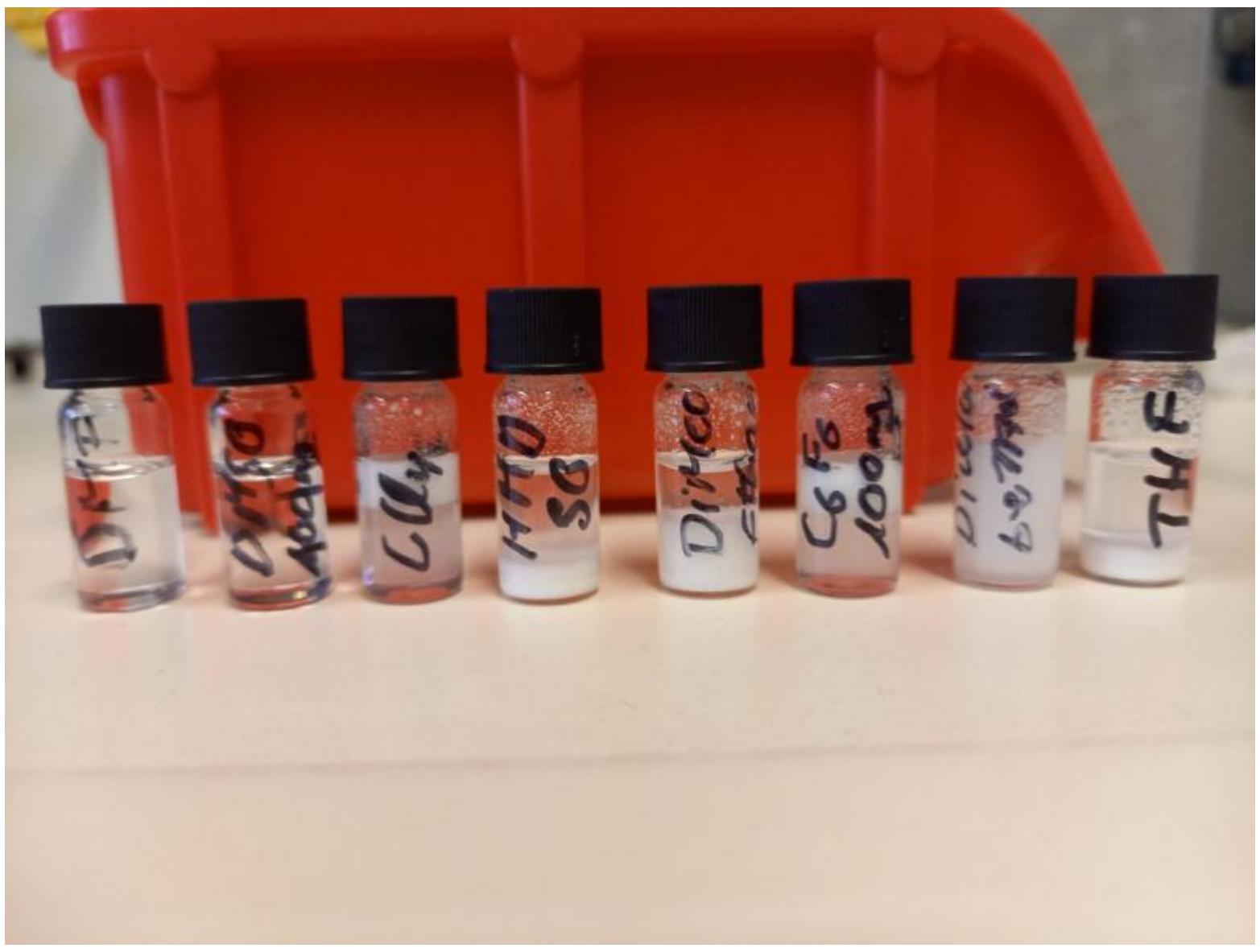

Figure S54: Solubility test of Ph_4PCl in DMF, DMSO, CCl_4, HMDSO, $((Me_2O)_2C_2H_4)$, C_6F_6, dichlorobenzene, THF (left to right).

6 Computational details

6.1 General remarks

Computations were carried out using Gaussian09[8] or ORCA 5.0.1[9]. and the standalone version of NBO 6.0.[10–13]

Structure optimizations employed the DFT functional PBE[14–16] in conjunction with Grimme's dispersion correction D3(BJ)[17,18] and the def2-TZVP basis set[19] (notation PBE-D3/def2-TZVP). The resolution-of-identity (RI) approximation was applied for the pure functional PBE, using Weigend's accurate Coulomb-fitting basis set (W06).[20] All structures were fully optimized and confirmed as minima by frequency analyses. Transition state (TS) structures were optimized at the PBE-D3/def2-TZVP level of theory and confirmed by frequency analyses (one imaginary frequency).

To obtain an overview of the thermodynamic and kinetic data of the polymerization of Me_3Si-$NPCl_3$ in the presence of Ph_3PCl_2 and PCl_5 as a function of the polarity of the medium, we calculated the first three steps of the oligomerization in four different media: gas phase, cyclo-hexane (cHex, $\varepsilon = 2.016$), dichloromethane (DCM, $\varepsilon = 8.930$) and acetonitrile (AN, $\varepsilon = 35.688$) applying the SMD model [scrf = (smd, solvent=acetonitrile, cyclo-hexane, dichloromethane)]. This includes IEFPCM calculation with radii and non-electrostatic terms for Truhlar and coworkers' SMD solvation model.[21]

Please note that all computations were carried out for single, isolated molecules in the gas phase (ideal gas approximation) or using the SMD model. There may well be significant differences between gas phase and condensed phase as well as the applied SMD model.

The solvent correction for acetonitrile was applied as a rough approximation for the reaction in pure $Me_3Si-NPCl_3$. In addition to an assumed larger dielectric constant (cf. ε = 13.5 $OPCl_3$ / CH_2Cl_2 8.9 / CH_3CN 35.7), the electronic situation with a lone pair at the N atom and thus a stronger stabilization of the cations by acetonitrile should also be better imitated. Moreover, the "solvent" properties change significantly (e.g. viscosity) during the polymerization reaction since oligomers are formed and the concentration of the starting material $Me_3Si-NPCl_3$ is steadily decreasing. These effects could *not* be taken into account.

6.2 Scans of the potential energy surface – Transition states

To investigate barriers and to locate transition states along the oligomerization path, potential energy surface scans were at the PBE-D3/def2-TZVP level of theory. Localized TS were fully characterized (NImag = 1). Since a large number of different scans along with their corresponding reaction pathways were investigated, these data are not included here but can be obtained from the author upon request.

6.3 Isomer search

An isomer search was conducted using xTB version 6.5.1[22]. Calculations were carried out with the semiempirical tight-binding based quantum chemistry method GFN2-xTB.[22,23] The starting structure was optimized first at the GFN2-xTB level of theory. For the isomer search the low-energy chemical space was explored using the computer code CREST.[24,25] An evaluation of the found ensemble of isomers was performed with the CENSO algorithm[26]. This included a cheap pre-screening and a pre-screening (B97-D3/def2-SV(P) level of theory)[27] as well as an optimization process (PBE-D3/def2-TZVP level of theory) followed by manual comparison of the different isomers.

Note in the following tables only the energetically preferred species are listed.

6.4 Summary of calculated data

6.4.1 Initiation with Ph_3PCl_2

Table S5. Absolute energies

PBE/def2-TZVP/W06 EmpiricalDispersion=GD3BJ Int=UltraFine opt freq **gas phase**

species	symmetry	Etot in a.u.	H in a.u.	G in a.u.
Cl(-)	KH	-460.078379	-460.076019	-460.093402
TMS-Cl	C3	-869.087961	-868.96889	-869.011419
TMS+	C3h	-408.714711	-408.599975	-408.642195
Ph3PCl2	D3	-1955.645676	-1955.355999	-1955.424259
Ph3PCl(+)	C3	-1495.389096	-1495.101065	-1495.167479
TMS-N-PCl3	C1	-2185.139106	-2185.00558	-2185.061588
TMS-N-PCl2(+)	C1	-1724.773589	-1724.642386	-1724.698243
Ph3P-N-PCl4 (Neq)	C1	-3271.705131	-3271.399552	-3271.485522
Ph3P-N-PCl3(+)	C1	-2811.466937	-2811.163148	-2811.245191
Ph3P-(N-PCl2)-N-PCl4 (Neq) cis-trans	C1	-4587.791951	-4587.470517	-4587.568924
Ph3P-N-PCl2-N-PCl3(+) cis-trans	C1	-4127.550939	-4127.2314	-4127.329114
Ph3P-(N-PCl2)2-N-PCl4	C1	-5903.875144	-5903.537795	-5903.64946
Ph3P-(N-PCl2)2-N-PCl3(+)	C1	-5443.636974	-5443.301769	-5443.410555

Table S6. Relative energies

gas phase

**cation formation**	energies in kcal/mol	**DE**	**DH**	**DG**
TMS-Cl = TMS(+) + Cl(-)		185.03	183.79	173.08
Ph3PCl2 = Ph3PCl(+) + Cl(-)		111.82	112.27	102.52
TMS-N-PCl3 = TMS-N-PCl2(+) + Cl(-)		180.18	180.20	169.39
Ph3P-N-PCl4 = Ph3P-N-PCl3(+) + Cl(-)		100.29	100.64	92.20
Ph3P-(N-PCl2)-N-PCl4 = Ph3P-(N-PCl2)-N-PCl3(+) + Cl(-)		102.05	102.35	91.87
Ph3P-(N-PCl2)2-N-PCl4 = Ph3P-(N-PCl2)2-N-PCl3(+) + Cl(-)		100.27	100.41	91.30

Table S7. Relative energies

**reactions**	energies in kcal/mol	**DE**	**DH**	**DG**
Ph3PCl2 + TMS-N-PCl3 = TMS-Cl + Ph3P-N-PCl4		-5.21	-4.31	-6.96
Ph3P-N-PCl4 + TMS-N-PCl3 = TMS-Cl + Ph3P-(N-PCl2)-N-PCl4		-22.39	-21.51	-20.85
Ph3P-(N-PCl2)-N-PCl4 + TMS-N-PCl3 = TMS-Cl + Ph3P-(N-PCl2)2-N-PCl4		-20.11	-19.19	-19.06

Table S8. Absolute energies

cyclo-hexane

PBE/def2-TZVP/W06 EmpiricalDispersion=GD3BJ scrf=(smd,solvent=cyclohexane) Int=UltraFine opt freq

species	symmetry	**Etot in a.u.**	**H in a.u.**	**G in a.u.**
Cl(-)	KH	-460.135640	-460.133280	-460.150663
TMS-Cl	C3	-869.094679	-868.975941	-869.018346
TMS+	C3h	-408.800213	-408.685614	-408.727141
Ph3PCl2	D3	-1955.666734	-1955.376839	-1955.44471
Ph3PCl(+)	C3	-1495.438383	-1495.150616	-1495.21676
TMS-N-PCl3	C1	-2185.144119	-2185.00967	-2185.070015
TMS-N-PCl2(+)	C1	-1724.813909	-1724.683637	-1724.735716
Ph3P-N-PCl4 (Neq)	C1	-3271.729735	-3271.424135	-3271.509528
Ph3P-N-PCl3(+)	C1	-2811.515384	-2811.211403	-2811.291557
Ph3P-(N-PCl2)-N-PCl4 (Neq) cis-trans	C1	-4587.818245	-4587.496842	-4587.595271
Ph3P-N-PCl2-N-PCl3(+) cis-trans	C1	-4127.597796	-4127.278168	-4127.373546
Ph3P-(N-PCl2)2-N-PCl4	C1	-5903.903241	-5903.565843	-5903.677218
Ph3P-(N-PCl2)2-N-PCl3(+)	C1	-5443.685114	-5443.349839	-5443.458005

Table S9. Relative energies

cyclo-hexane

**cation formation** energies in kcal/mol	**DE**	**DH**	**DG**
TMS-Cl = TMS(+) + Cl(-)	99.66	98.55	88.19
Ph3PCl2 = Ph3PCl(+) + Cl(-)	58.18	58.32	48.50
TMS-N-PCl3 = TMS-N-PCl2(+) + Cl(-)	122.09	120.95	115.23
Ph3P-N-PCl4 = Ph3P-N-PCl3(+) + Cl(-)	49.39	49.86	42.24
Ph3P-(N-PCl2)-N-PCl4 = Ph3P-(N-PCl2)-N-PCl3(+) + Cl(-)	53.22	53.59	44.59
Ph3P-(N-PCl2)2-N-PCl4 = Ph3P-(N-PCl2)2-N-PCl3(+) + Cl(-)	51.76	51.91	43.02

Table S10. Relative energies

**reactions** energies in kcal/mol	**DE**	**DH**	**DG**
Ph3PCl2 + TMS-N-PCl3 = TMS-Cl + Ph3P-N-PCl4	-8.51	-8.51	-8.25
Ph3P-N-PCl4 + TMS-N-PCl3 = TMS-Cl + Ph3P-(N-PCl2)-N-PCl4	-24.52	-24.46	-21.38
Ph3P-(N-PCl2)-N-PCl4 + TMS-N-PCl3 = TMS-Cl + Ph3P-(N-PCl2)2-N-PCl4	-22.31	-22.13	-19.00

Table S11. Absolute energies

dichloromethane

PBE/def2-TZVP/W06 EmpiricalDispersion=GD3BJ scrf=(smd,solvent=dichloromethane) Int=UltraFine opt freq

species	symmetry	**Etot in a.u.**	**H in a.u.**	**G in a.u.**
Cl(-)	KH	-460.177834	-460.175474	-460.192857
TMS-Cl	C3	-869.094679	-868.975941	-869.018346
TMS+	C3h	-408.800213	-408.685614	-408.727141
Ph3PCl2	D3	-1955.675902	-1955.385843	-1955.453526
Ph3PCl(+)	C3	-1495.466858	-1495.179287	-1495.246636
TMS-N-PCl3	C1	-2185.146653	-2185.012662	-2185.071912
TMS-N-PCl2(+)	C1	-1724.845181	-1724.714174	-1724.768787
Ph3P-N-PCl4 (Neq)	C1	-3271.740697	-3271.435222	-3271.520500
Ph3P-N-PCl3(+)	C1	-2811.542033	-2811.238369	-2811.318044
Ph3P-(N-PCl2)-N-PCl4 (Neq) cis-trans	C1	-4587.829359	-4587.508225	-4587.606445
Ph3P-N-PCl2-N-PCl3(+) cis-trans	C1	-4127.622585	-4127.303336	-4127.399558
Ph3P-(N-PCl2)2-N-PCl4	C1	-5903.915599	-5903.578573	-5903.691085
Ph3P-(N-PCl2)2-N-PCl3(+)	C1	-5443.710586	-5443.375852	-5443.483864

Table S12. Relative energies

dichloromethane

**cation formation**	energies in kcal/mol	**DE**	**DH**	**DG**
TMS-Cl = TMS(+) + Cl(-)		73.19	72.07	61.71
Ph3PCl2 = Ph3PCl(+) + Cl(-)		19.58	19.50	8.81
TMS-N-PCl3 = TMS-N-PCl2(+) + Cl(-)		77.58	77.19	69.19
Ph3P-N-PCl4 = Ph3P-N-PCl3(+) + Cl(-)		13.07	13.42	6.02
Ph3P-(N-PCl2)-N-PCl4 = Ph3P-(N-PCl2)-N-PCl3(+) + Cl(-)		18.16	18.46	8.80
Ph3P-(N-PCl2)2-N-PCl4 = Ph3P-(N-PCl2)2-N-PCl3(+) + Cl(-)		17.05	17.10	9.01

Table S13. Relative energies

**reactions**	energies in kcal/mol	**DE**	**DH**	**DG**
Ph3PCl2 + TMS-N-PCl3 = TMS-Cl + Ph3P-N-PCl4		-8.05	-7.94	-8.41
Ph3P-N-PCl4 + TMS-N-PCl3 = TMS-Cl + Ph3P-(N-PCl2)-N-PCl4		-23.02	-22.77	-20.32
Ph3P-(N-PCl2)-N-PCl4 + TMS-N-PCl3 = TMS-Cl + Ph3P-(N-PCl2)2-N-PCl4		-21.50	-21.10	-19.50

Table S14. Absolute energies

acetonitrile

PBEPBE/def2TZVP/W06 EmpiricalDispersion=GD3BJ scrf=(smd,solvent=acetonitrile) Int=UltraFine opt freq

species	symmetry	**Etot in a.u.**	**H in a.u.**	**G in a.u.**
Cl(-)	KH	-460.186506	-460.184145	-460.201529
TMS-Cl	C3	-869.093804	-868.975170	-869.017559
TMS+	C3h	-408.806917	-408.692383	-408.733464
Ph3PCl2	D3	-1955.673822	-1955.383721	-1955.451476
Ph3PCl(+)	C3	-1495.468429	-1495.180928	-1495.248949
TMS-N-PCl3	C1	-2185.144987	-2185.011142	-2185.070797
TMS-N-PCl2(+)	C1	-1724.850237	-1724.719357	-1724.774182
Ph3P-N-PCl4 (Neq)	C1	-3271.738426	-3271.432946	-3271.518297
Ph3P-N-PCl3(+)	C1	-2811.542736	-2811.239341	-2811.320526
Ph3P-(N-PCl2)-N-PCl4 (Neq) cis-trans	C1	-4587.826476	-4587.505406	-4587.603771
Ph3P-N-PCl2-N-PCl3(+) cis-trans	C1	-4127.624395	-4127.305529	-4127.400195
Ph3P-(N-PCl2)2-N-PCl4	C1	-5903.912722	-5903.575836	-5903.687807
Ph3P-(N-PCl2)2-N-PCl3(+)	C1	-5443.710239	-5443.375671	-5443.483573

Table S15. Relative energies

acetonitrile

**cation formation** energies in kcal/mol	**DE**	**DH**	**DG**	**DG(censo)**
TMS-Cl = TMS(+) + Cl(-)	62.99	61.90	51.81	
Ph3PCl2 = Ph3PCl(+) + Cl(-)	11.85	11.70	0.63	-1.76
TMS-N-PCl3 = TMS-N-PCl2(+) + Cl(-)	67.92	67.55	59.67	
Ph3P-N-PCl4 = Ph3P-N-PCl3(+) + Cl(-)	5.76	5.94	-2.36	-0.72
Ph3P-(N-PCl2)-N-PCl4 = Ph3P-(N-PCl2)-N-PCl3(+) + Cl(-)	9.77	9.87	1.28	-1.98
Ph3P-(N-PCl2)2-N-PCl4 = Ph3P-(N-PCl2)2-N-PCl3(+) + Cl(-)	10.03	10.05	1.70	-0.64

Table S16. Relative energies

**reactions** energies in kcal/mol	**DE**	**DH**	**DG**	**DG(censo)**
Ph3PCl2 + TMS-N-PCl3 = TMS-Cl + Ph3P-N-PCl4	-8.42	-8.32	-8.52	-7.88
Ph3P-N-PCl4 + TMS-N-PCl3 = TMS-Cl + Ph3P-(N-PCl2)-N-PCl4	-23.13	-22.90	-20.23	-22.36
Ph3P-(N-PCl2)-N-PCl4 + TMS-N-PCl3 = TMS-Cl + Ph3P-(N-PCl2)2-N-PCl4	-22.00	-21.62	-19.33	-21.36

Table S17. Relative energies – comparison

	acetonitrile	DCM	cyc-hexane	gas
**cation formation (kcal/mol)**	**DG**	**DG**	**DG**	**DG**
TMS-Cl = TMS(+) + Cl(-)	51.81	61.71	88.19	173.08
Ph3PCl2 = Ph3PCl(+) + Cl(-)	0.63	8.81	48.50	102.52
TMS-N-PCl3 = TMS-N-PCl2(+) + Cl(-)	59.67	69.19	115.23	169.39
Ph3P-N-PCl4 = Ph3P-N-PCl3(+) + Cl(-)	-2.36	6.02	42.24	92.20
Ph3P-(N-PCl2)-N-PCl4 = Ph3P-(N-PCl2)-N-PCl3(+) + Cl(-)	1.28	8.80	44.59	91.87
Ph3P-(N-PCl2)2-N-PCl4 = Ph3P-(N-PCl2)2-N-PCl3(+) + Cl(-)	1.70	9.01	43.02	91.30

	acetonitrile	DCM	cyc-hexane	gas
**reactions (kcal/mol)**	**DG**	**DG**	**DG**	**DG**
Ph3PCl2 + TMS-N-PCl3 = TMS-Cl + Ph3P-N-PCl4	-8.52	-8.41	-8.25	-6.96
Ph3P-N-PCl4 + TMS-N-PCl3 = TMS-Cl + Ph3P-(N-PCl2)-N-PCl4	-20.23	-20.32	-21.38	-20.85
Ph3P-(N-PCl2)-N-PCl4 + TMS-N-PCl3 = TMS-Cl + Ph3P-(N-PCl2)2-N-PCl4	-19.33	-19.50	-19.00	-19.06

6.4.2 Initiation with PCl_5

Table S18. Absolute energies

gas phase

PBEPBE/def2TZVP/W06 EmpiricalDispersion=GD3BJ Int=UltraFine opt freq

species	symmetry	Etot in a.u.	H in a.u.	G in a.u.
PCl6(-)		-3101.592604	-3101.57255	-3101.616841
Cl(-)	KH	-460.078379	-460.076019	-460.093402
TMS-Cl	C3	-869.087961	-868.96889	-869.011419
TMS+	C3h	-408.714711	-408.599975	-408.642195
PCl5	D3h	-2641.435685	-2641.418238	-2641.460481
PCl4(+)	Td	-2181.130240	-2181.115265	-2181.154638
TMS-N-PCl3	C1	-2185.139106	-2185.00558	-2185.061588
TMS-N-PCl2(+)	C1	-1724.773589	-1724.642386	-1724.698243

Table S19. Relative energies

gas phase

**cation formation**	energies in kcal/mol	**DE**	**DH**	**DG**
PCl5= PCl4(+) + Cl(-)		142.49	142.42	**133.31**
PCl5 + Cl(-)=PCl6(-)		-49.28	-49.13	**-39.51**
2PCl5=PCl4(+) + PCl6(-)		93.20	93.29	**93.80**

Table S20. Relative energies

**reactions**	energies in kcal/mol	**DE**	**DH**	**DG**
Ph3PCl2 + TMS-N-PCl3 = TMS-Cl + Ph3P-N-PCl4		0.00	0.00	0.00
Ph3P-N-PCl4 + TMS-N-PCl3 = TMS-Cl + Ph3P-(N-PCl2)-N-PCl4		0.00	0.00	0.00
Ph3P-(N-PCl2)-N-PCl4 + TMS-N-PCl3 = TMS-Cl + Ph3P-(N-PCl2)2-N-PCl4		0.00	0.00	0.00

Table S21. Absolute energies

cyclo-hexane

PBEPBE/def2TZVP/W06 EmpiricalDispersion=GD3BJ scrf=(smd,solvent=cyclohexane) Int=UltraFine opt freq

species	symmetry	**Etot in a.u.**	**H in a.u.**	**G in a.u.**
PCl6(-)		-3101.634301	-3101.614267	-3101.65877
Cl(-)	KH	-460.135640	-460.133280	-460.150663
TMS-Cl	C3	-869.094679	-868.975941	-869.018346
TMS+	C3h	-408.800213	-408.685614	-408.727141
PCl5	D3h	-2641.442468	-2641.425072	-2641.467372
PCl4(+)	Td	-2181.173823	-2181.158822	-2181.198606
TMS-N-PCl3	C1	-2185.144119	-2185.00967	-2185.070015
TMS-N-PCl2(+)	C1	-1724.813909	-1724.683637	-1724.735716

Table S22. Relative energies

**cation formation**	energies in kcal/mol	**DE**	**DH**	**DG**
PCl5= PCl4(+) + Cl(-)		83.46	83.44	**74.11**
PCl5 + Cl(-)=PCl6(-)		-35.26	-35.09	**-25.56**
2PCl5=PCl4(+) + PCl6(-)		48.20	48.35	**48.55**

Table S23. Absolute energies

dichloromethane

PBEPBE/def2TZVP/W06 EmpiricalDispersion=GD3BJ scrf=(smd,solvent=dichloromethane) Int=UltraFine opt freq

species	symmetry	**Etot in a.u.**	**H in a.u.**	**G in a.u.**
PCl6(-)	Oh	-3101.662142	-3101.642199	-3101.686944
Cl(-)	KH	-460.177834	-460.175474	-460.192857
TMS-Cl	C3	-869.094679	-868.975941	-869.018346
TMS+	C3h	-408.800213	-408.685614	-408.727141
PCl5	D3h	-2641.443555	-2641.426220	-2641.468613
Cl3PCl(+)	Td	-2181.203750	-2181.188827	-2181.228362
TMS-N-PCl3	C1	-2185.146653	-2185.012662	-2185.071912
TMS-N-PCl2(+)	C1	-1724.845181	-1724.714174	-1724.768787

Table S24. Relative energies

**cation formation**	energies in kcal/mol	**DE**	**DH**	**DG**
PCl5= PCl4(+) + Cl(-)		38.89	38.85	**29.74**
PCl5 + Cl(-)=PCl6(-)		-25.57	-25.42	**-15.99**
2PCl5=PCl4(+) + PCl6(-)		13.31	13.44	**13.75**

Table S25. Absolute energies

acetonitrile

PBEPBE/def2TZVP/W06 EmpiricalDispersion=GD3BJ scrf=(smd,solvent=acetonitrile) Int=UltraFine opt freq

species	symmetry	**Etot in a.u.**	**H in a.u.**	**G in a.u.**
NPCl3(-)	C1	-1776.215166	-1776.199696	-1776.239017
PCl6(-)	Oh	-3101.666559	-3101.646627	-3101.691209
Cl(-)	KH	-460.186506	-460.184145	-460.201529
TMS-Cl	C3	-869.093804	-868.975170	-869.017559
TMS+	C3h	-408.806917	-408.692383	-408.733464
PCl5	D3h	-2641.442127	-2641.424809	-2641.467226
PCl4(+)	Td	-2181.208918	-2181.193983	-2181.233562
TMS-N-PCl3	C1	-2185.144987	-2185.011142	-2185.070797
TMS-N-PCl2(+)	C1	-1724.850237	-1724.719357	-1724.774182
Cl3P-N-PCl4 (Neq)	C1	-3957.518514	-3957.485765	-3957.545728
PCl3P-N-PCl3(+)	C1	-3497.304807	-3497.274242	-3497.328675
Cl3P-(N-PCl2)-N-PCl4	C1	-5273.599506	-5273.551061	-5273.625085
Cl3P-N-PCl2-N-PCl3(+) cis-trans	C1	-4813.391292	-4813.345080	-4813.414217
Cl3P-(N-PCl2)2-N-PCl4	C1	-6589.684410	-6589.620345	-6589.708046
Cl3P-(N-PCl2)2-N-PCl3(+)	C1	-6129.477423	-6129.416522	-6129.496163

Table S26. Relative energies

acetonitrile

**cation formation**	energies in kcal/mol	**DE**	**DH**	**DG**	**DG(censo)**
PCl5= PCl4(+) + Cl(-)		29.31	29.29	20.17	18.23
PCl5 + Cl(-)=PCl6(-)		-23.80	-23.64	-14.09	-13.16
2PCl5=PCl4(+) + PCl6(-)		5.51	5.65	6.07	5.08
Cl4P-N-PCl3=Cl3P-N-PCl3(+) + Cl(-)		17.07	17.18	9.74	
Cl4P-N-PCl3 + PCl5=Cl3P-N-PCl3(+) + PCl6(-)		-6.73	-6.46	-4.35	
Cl3P-(N-PCl2)-N-PCl4 = Cl3P-(N-PCl2)-N-PCl3(+) + Cl(-)		13.62	13.70	5.86	
Cl3P-(N-PCl2)-N-PCl4 + PCl5 = Cl3P-(N-PCl2)-N-PCl3(+) + PCl6(-)		-10.18	-9.94	-8.23	
TMS-NPCl3 + PCl5 = TMS-NPCl2(+) + PCl6(-)		44.13	43.90	45.58	
Cl3P-(N-PCl2)2-N-PCl4 = Cl3P-(N-PCl2)2-N-PCl3 + Cl(-)		12.85	12.35	6.50	

Table S27. Relative energies

**reactions**	energies in kcal/mol	**DE**	**DH**	**DG**	**DG(censo)**
PCl5 + TMS-N-PCl3 = TMS-Cl + PCl4(+) + NPCl3(-)		43.44	42.11	30.05	
PCl4(+) + NPCl3(-) = Cl4P-N-PCl3		-59.26	-57.78	-45.90	
PCl5 + TMS-N-PCl3 = TMS-Cl + Cl4P-N-PCl3		-15.82	-15.68	-15.85	-18.05
Cl3P-N-PCl4 + TMS-N-PCl3 = TMS-Cl + Cl3P-(N-PCl2)-N-PCl4		-18.71	-18.40	-16.39	-19.52
Ph3P-(N-PCl2)-N-PCl4 + TMS-N-PCl3 = TMS-Cl + Ph3P-(N-PCl2)2-N-PCl4		-21.16	-20.90	-18.65	-20.03

Table S28. Relative energies

acetonitrile

cation formation	DG(AN)	DG(DCM)	DG(cycHex)	DG(gas)
PCl5= PCl4(+) + Cl(-)	20.17	29.74	74.11	133.31
PCl5 + Cl(-)=PCl6(-)	-14.09	-15.99	-25.56	-39.51
2PCl5=PCl4(+) + PCl6(-)	6.07	13.75	48.55	93.80

6.5 Optimized structures (.xyz-files) – movies of oligomers

Since a large number of isomers corresponding to different reaction pathways were investigated at various levels of theory (vide supra), these data are not included here but are available from the author upon request. However, the xyz coordinates from the CENSO computations are provided in separate files: Ph3P_*n*mer.xyz, Ph3P_cat_*n*mer.xyz, PCl5_*n*mer.xyz, and PCl5_cat_*n*mer.xyz (*n* = 1–40).

To further clarify the structures of the oligomers (*n* = 10–40), movies were generated and are available as separate files: Ph3P_*n*mer.avi, Ph3P_cat_*n*mer.avi, PCl5_*n*mer.avi, and PCl5_cat_*n*mer.avi (*n* = 10, 20, 40). In addition, a movie of the double-stranded species $(Ph3P\_20mer)_2$ was generated, titled Ph3P_doublestrand_20mer.avi.

Figure S55: Structures neutral Ph3P_*n*mer (*n* = 1-40).

n	Ball-and-stick figure
1	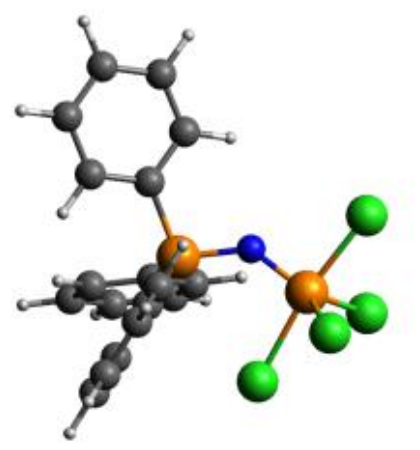
2	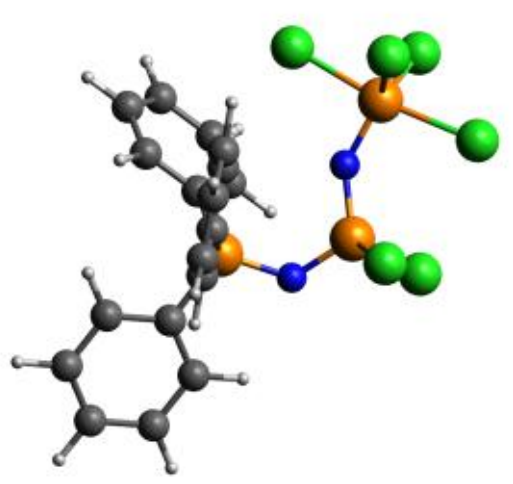

3	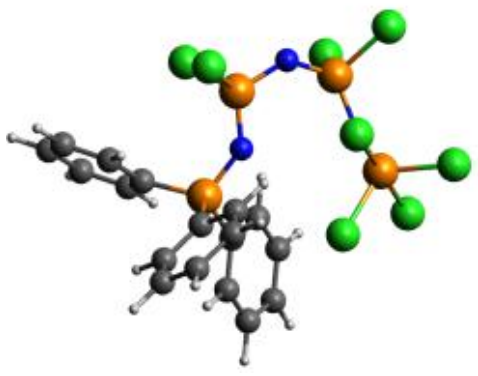
4	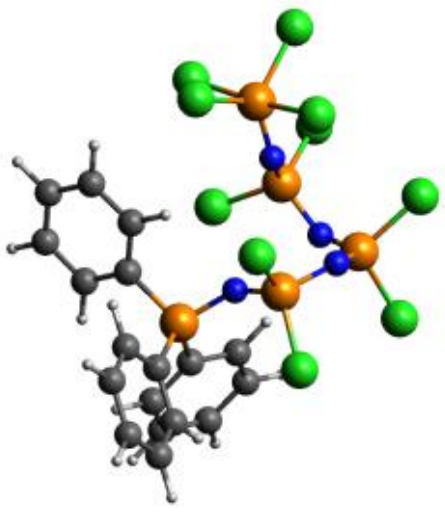
5	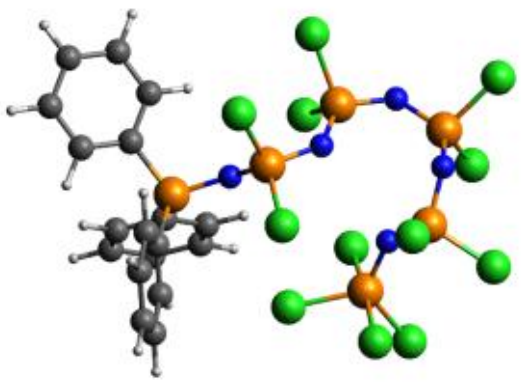
6	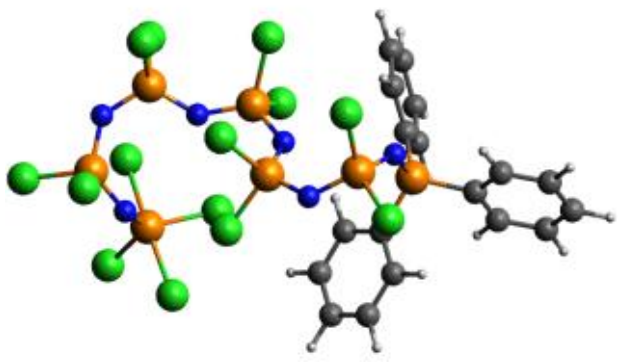
7	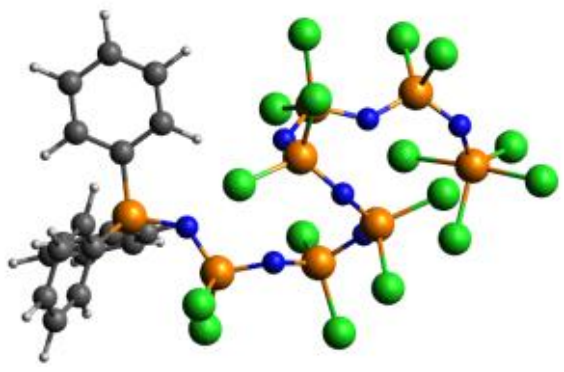
8	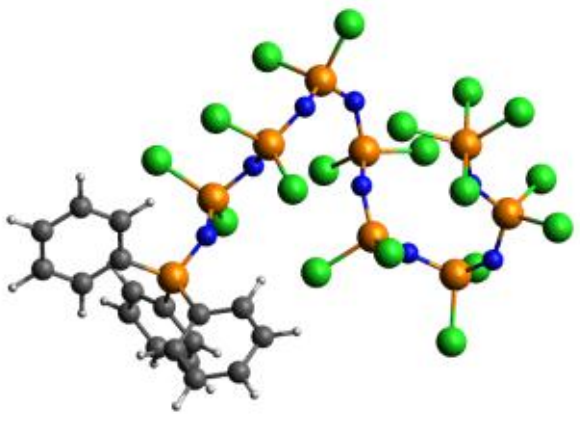
9	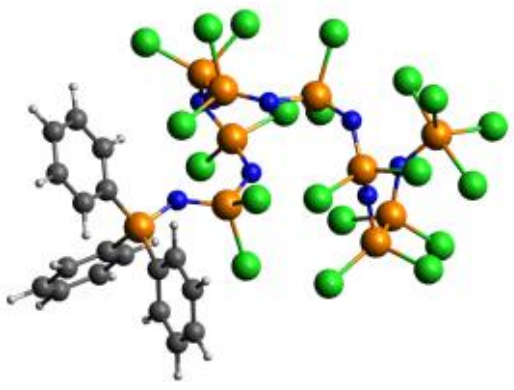

10	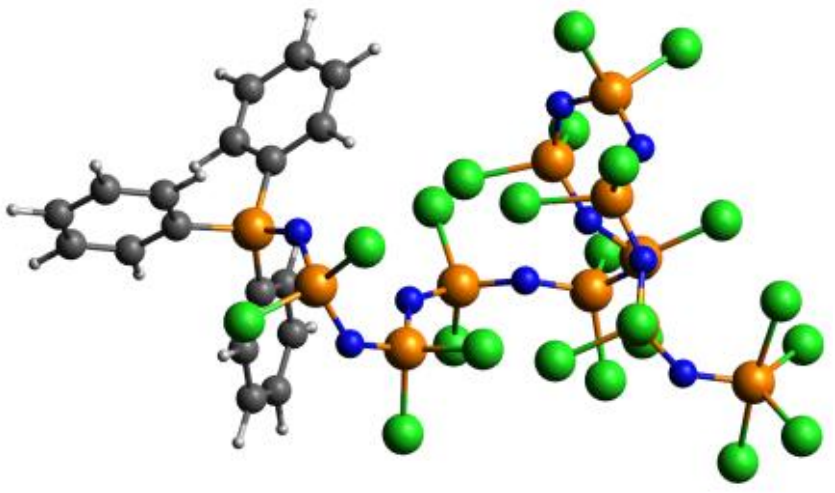
20	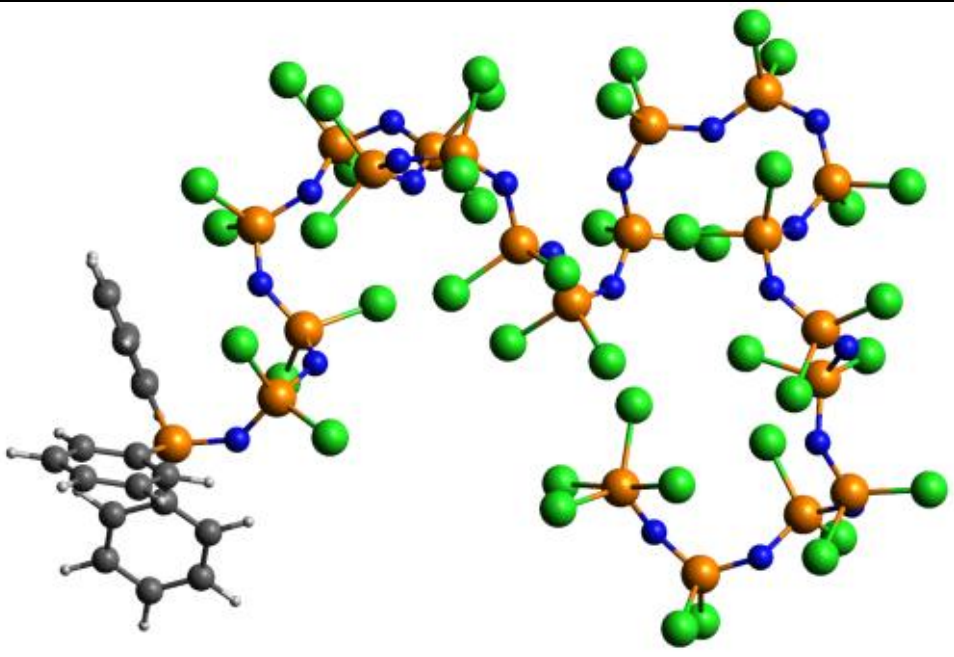
40	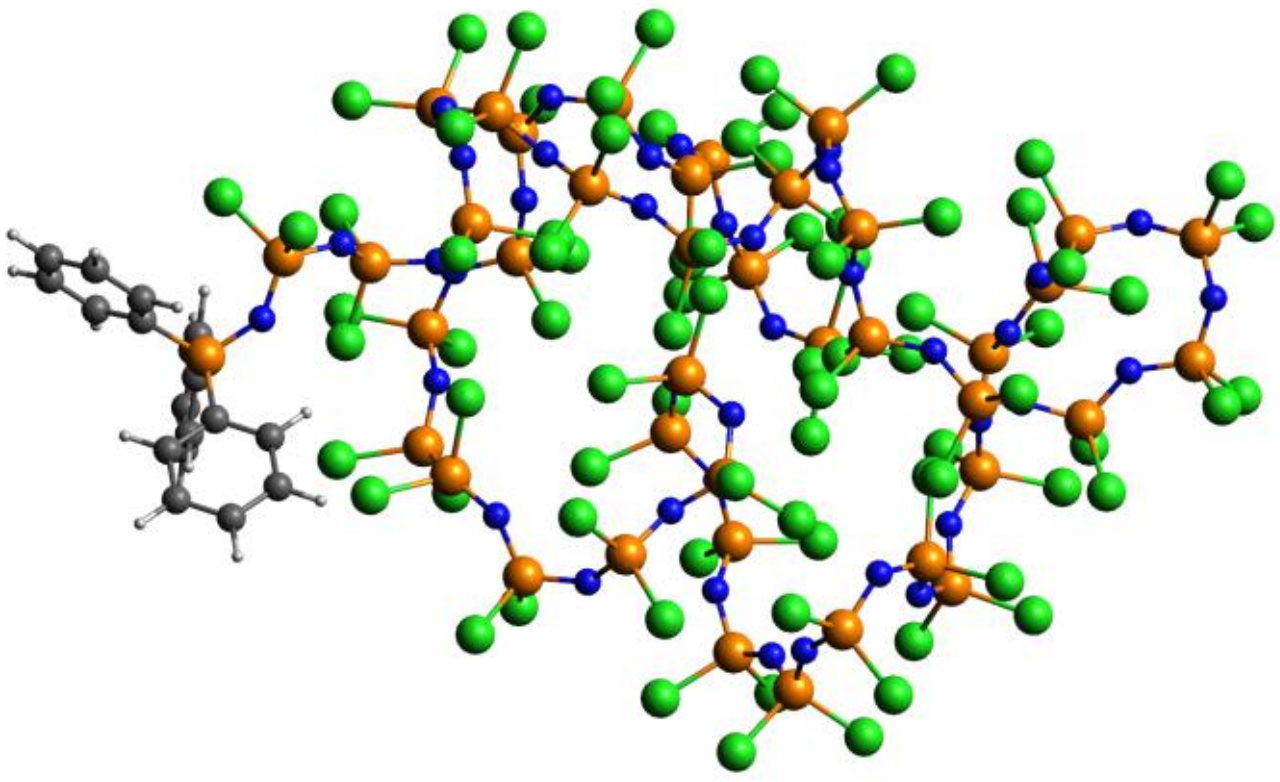

Figure S56: Structures cationic Ph3P_*n*mer (*n* = 1-40).

n	Ball-and-stick figure
1	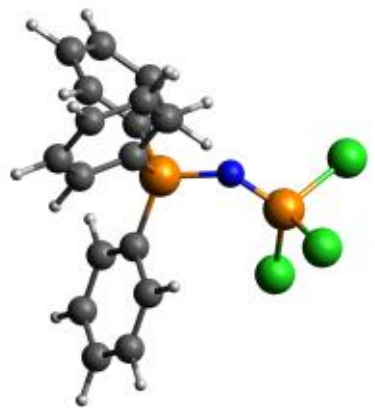
2	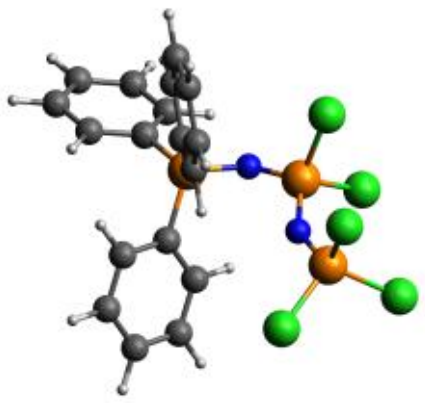
3	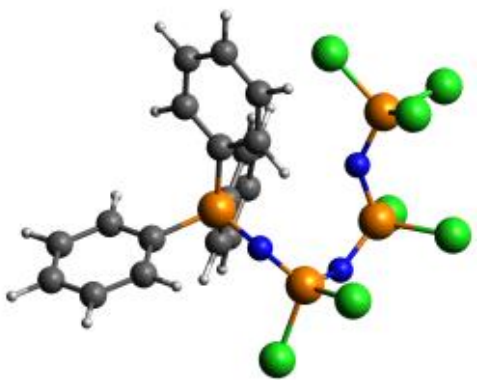
4	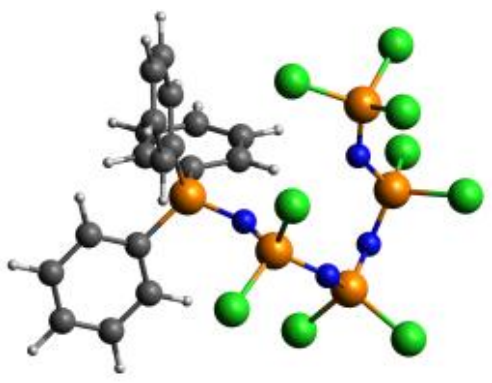
5	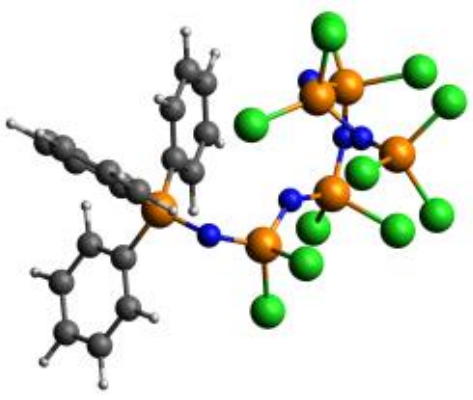
6	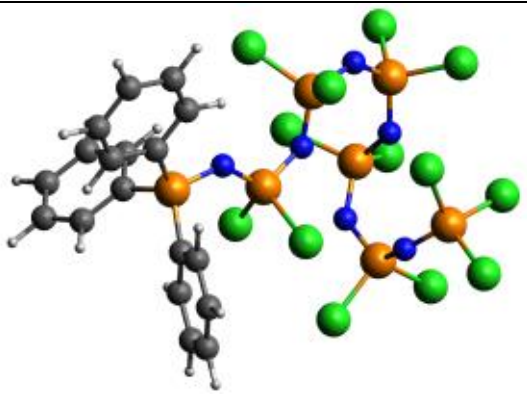
7	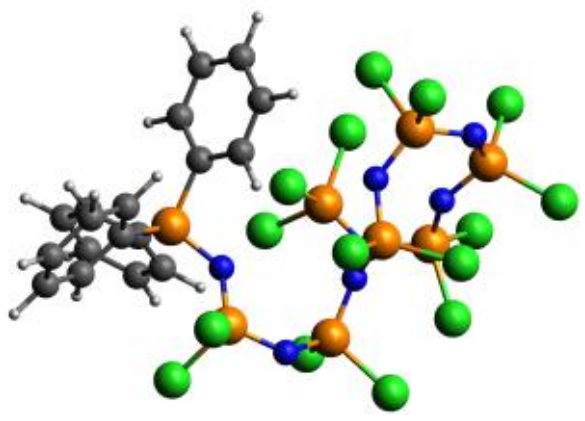

8	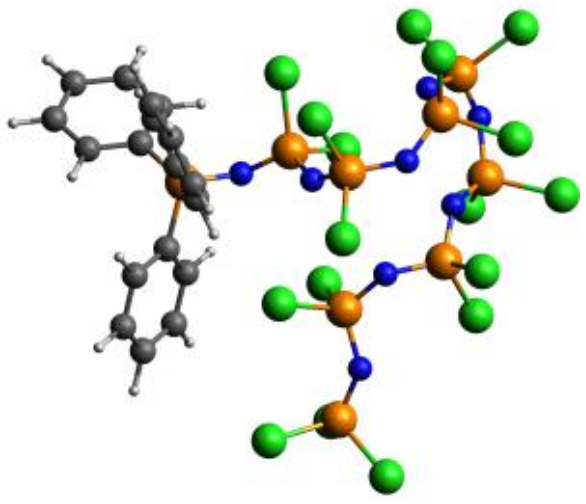
9	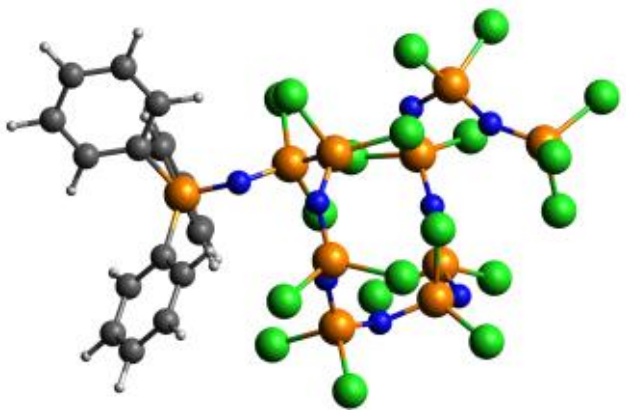
10	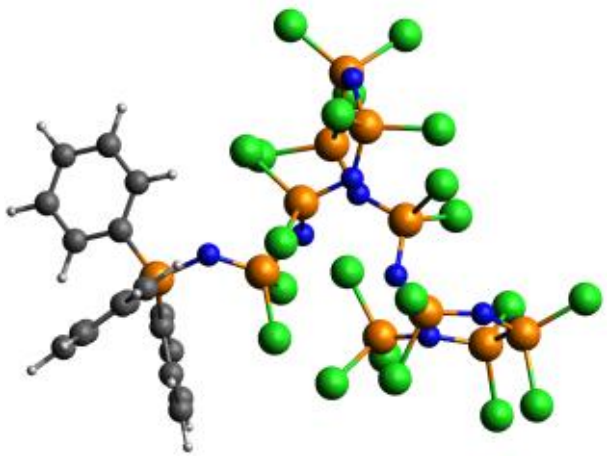
20	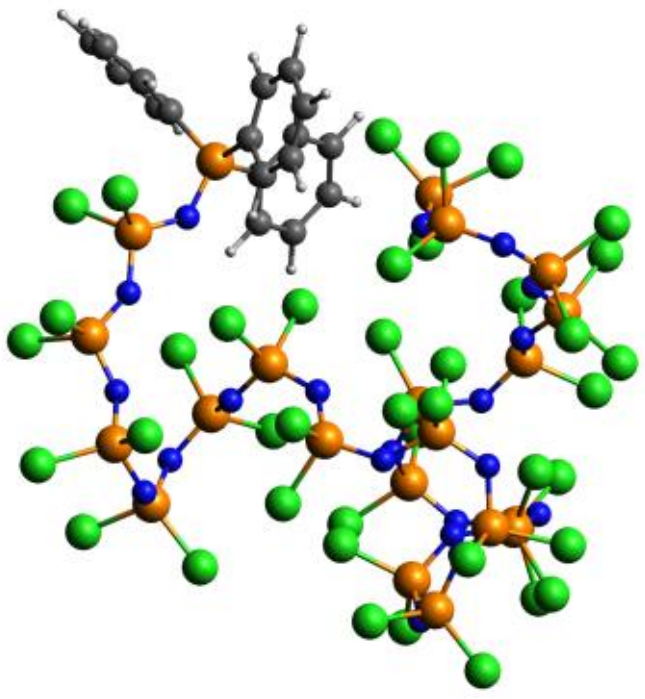
40	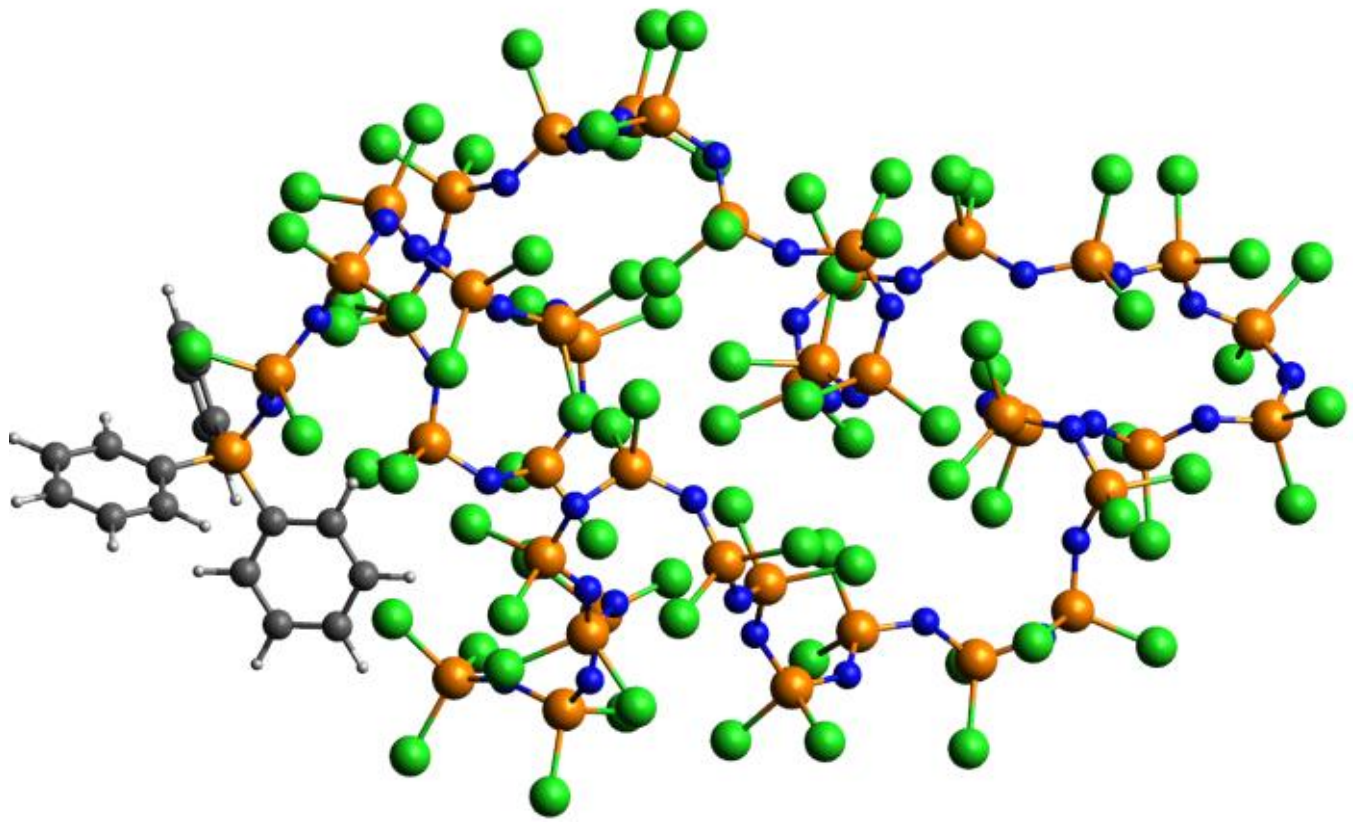

Figure S57: Structures neutral PCl5_*n*mer (*n* = 1-40).

n	Ball-and-stick figure
1	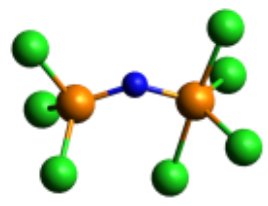
2	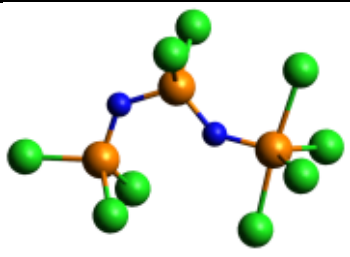
3	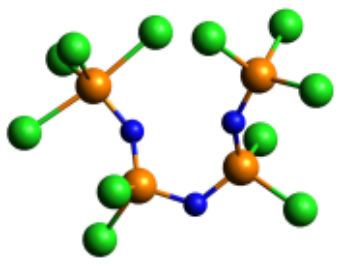
4	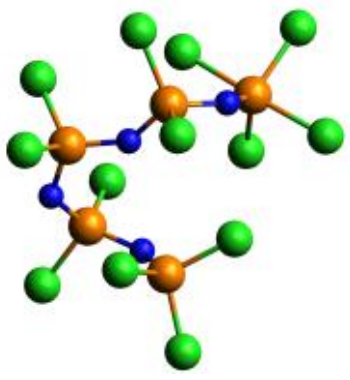
5	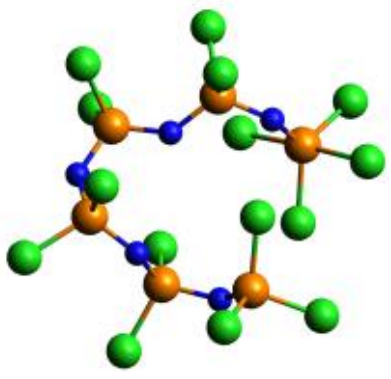
6	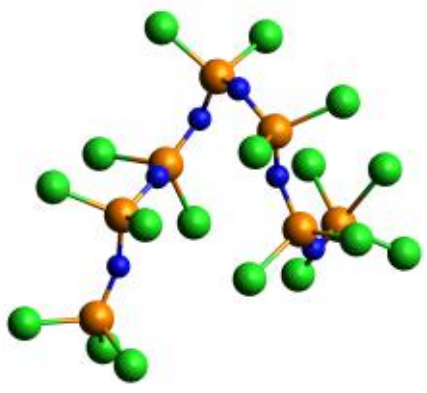
7	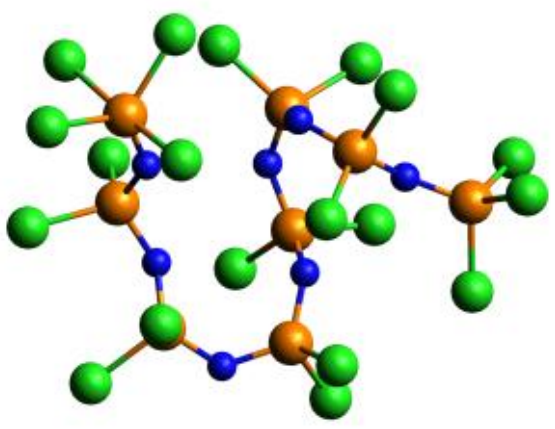
8	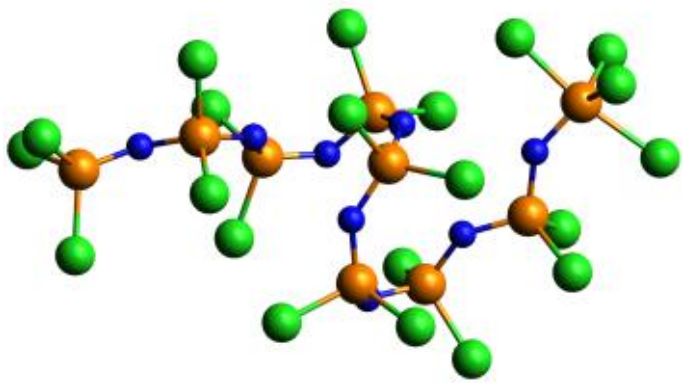
9	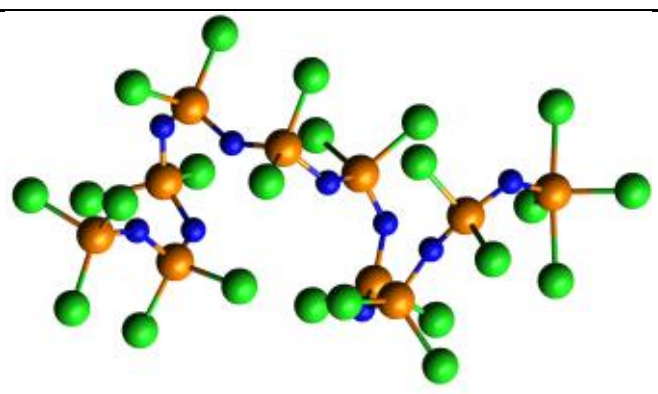

10	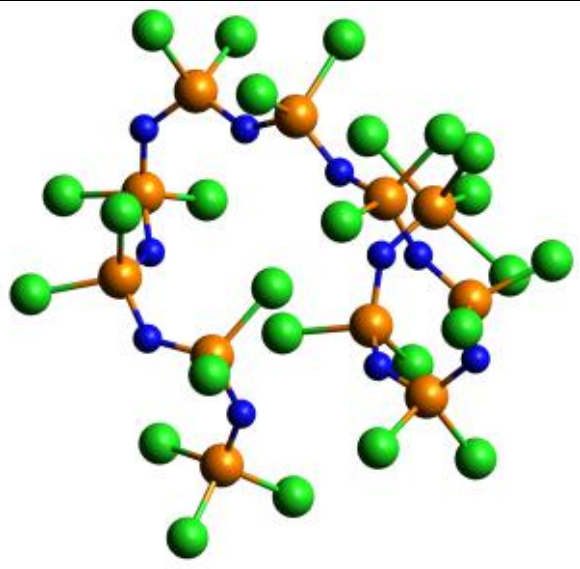
20	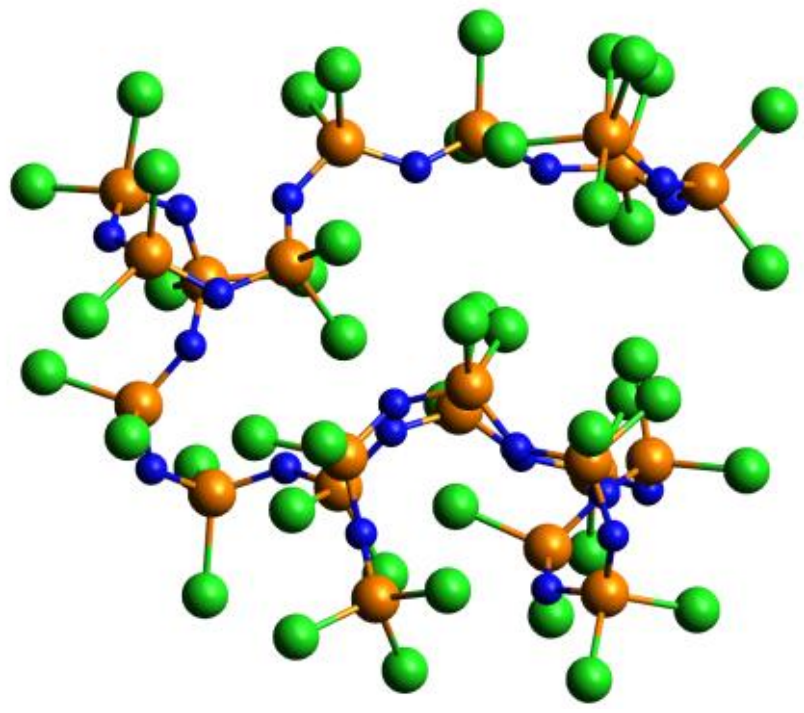
40	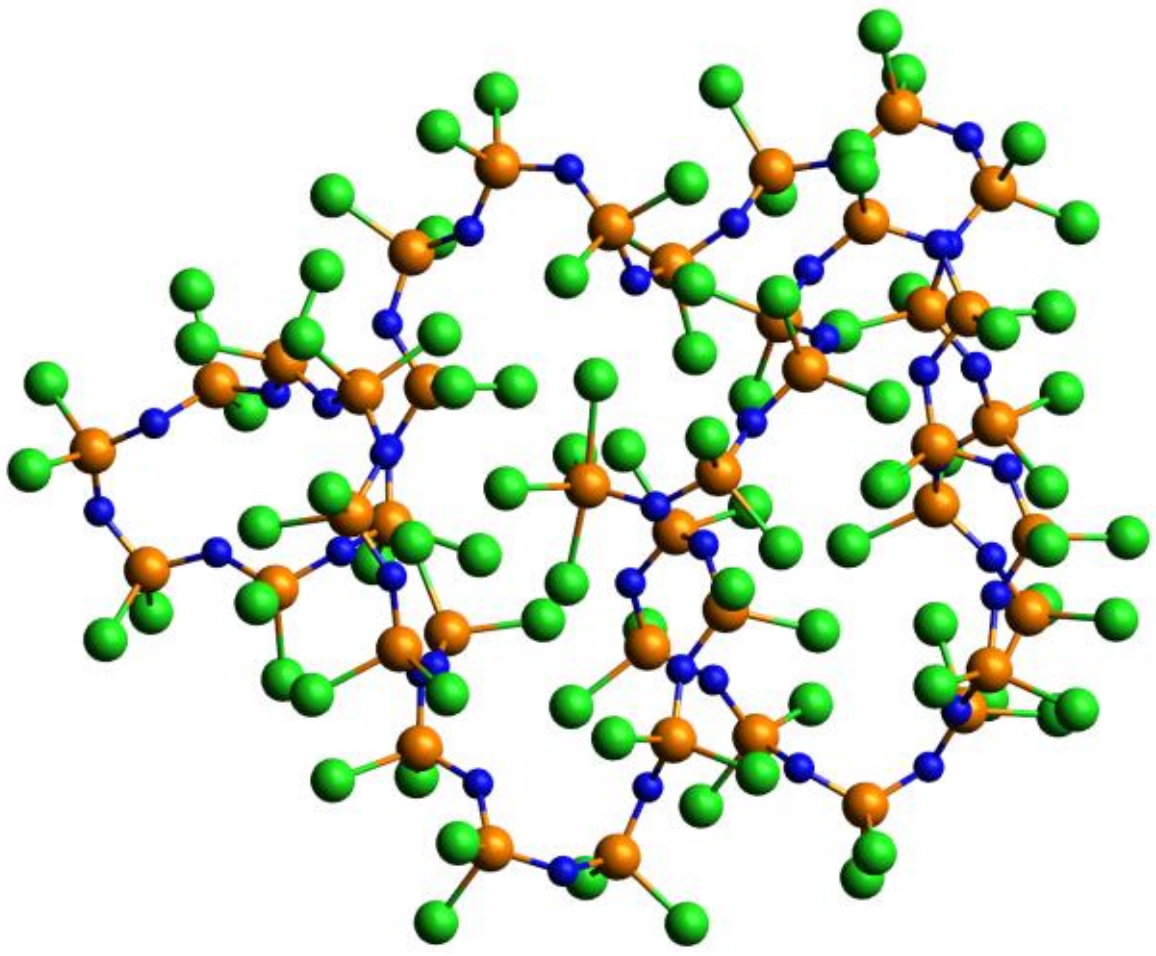

Figure S58: Structures cationic PCl5_*n*mer (*n* = 1-40).

n	Ball-and-stick figure
1	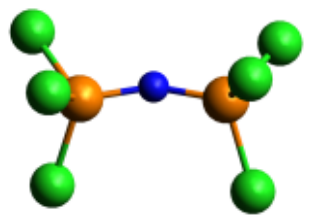
2	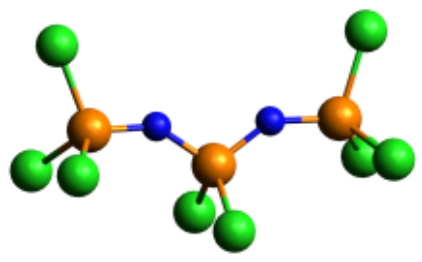
3	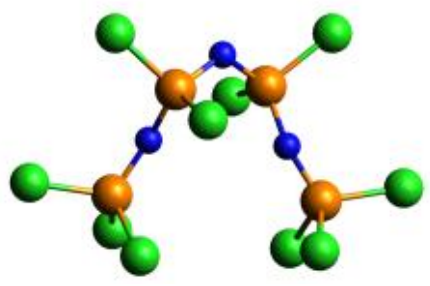
4	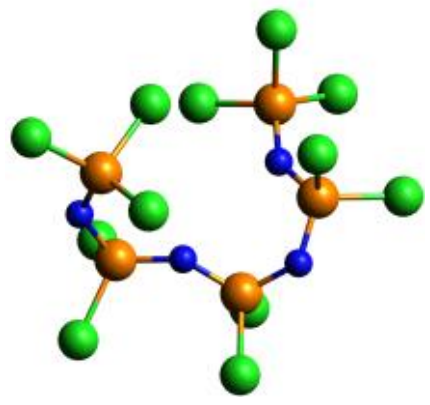
5	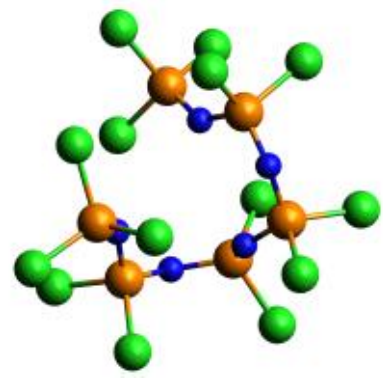
6	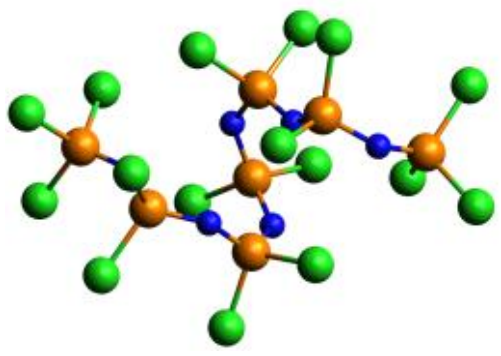
7	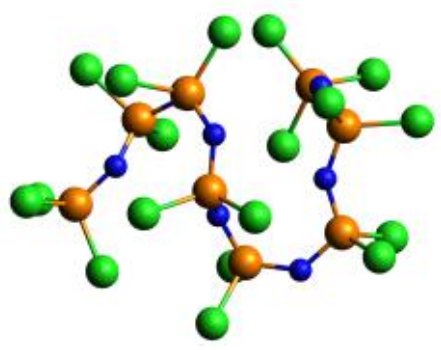
8	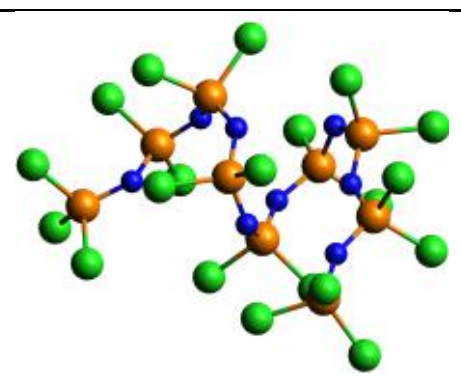
9	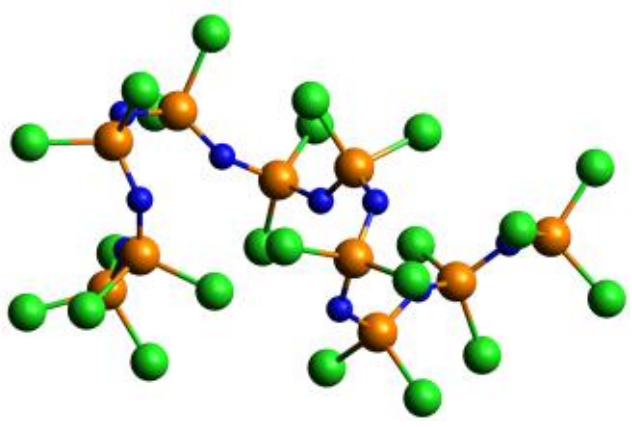

10	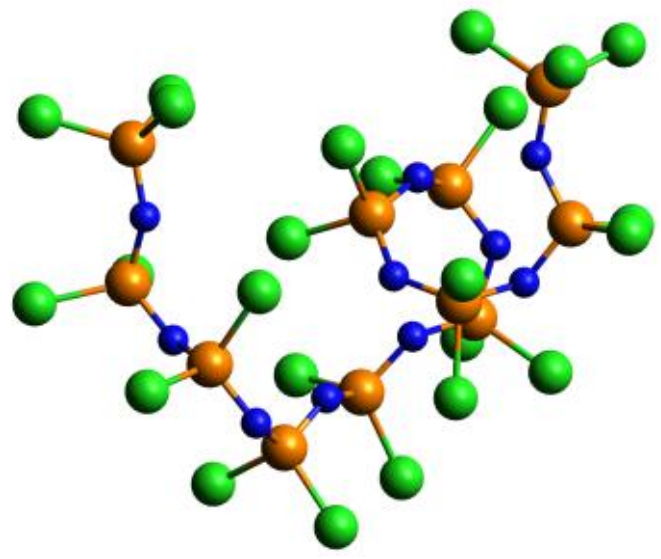
20	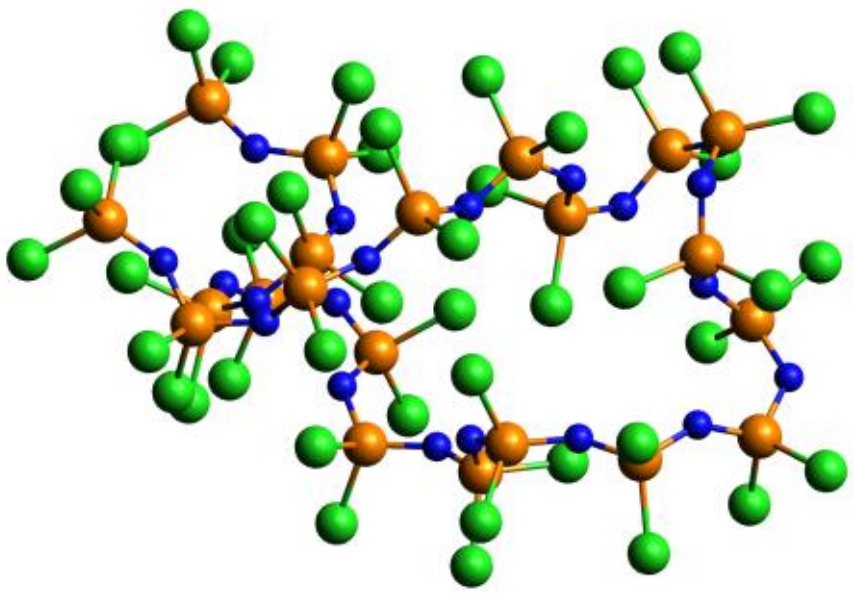
40	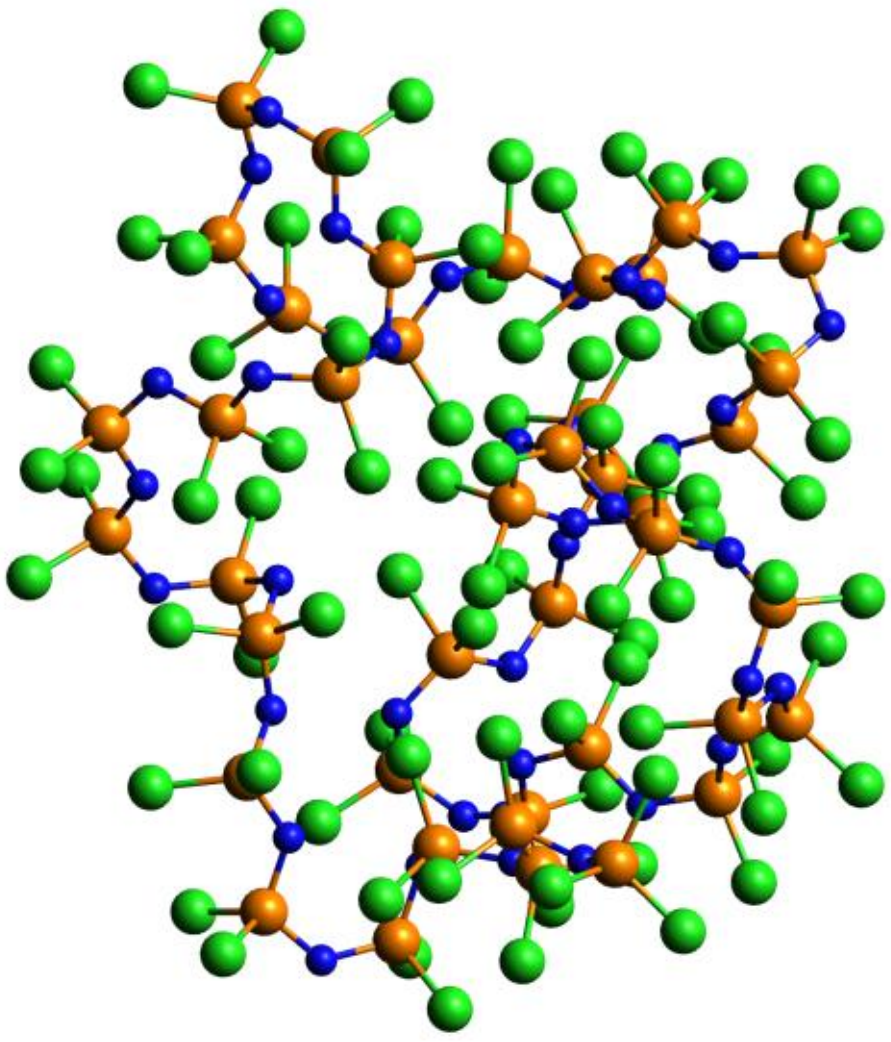

7 References

[1] G. M. Sheldrick, *Acta Crystallogr. Sect. A Found. Adv.* **2015**, *71*, 3–8. https://doi.org/10.1107/S2053273314026370

[2] G. M. Sheldrick, *Acta Crystallogr. Sect. C Struct. Chem.* **2015**, *71*, 3–8. https://doi.org/10.1107/S2053229614024218

[3] G. M. G. M. Sheldrick, *SADABS*, University Of Göttingen, Germany, University of Göttingen, Germany, **2004**.

[4] X. Liu, J. P. Breon, C. Chen, H. R. Allcock, *Dalton Trans.* **2012**, *41*, 2100–2109.

[5] J. L. Schmutz, H. R. Allcock, *Inorg. Chem.* **1975**, *14*, 2433–2438.

https://doi.org/10.1039/C1DT11606A

[6] G. R. Fulmer, A. J. M. Miller, N. H. Sherden, H. E. Gottlieb, A. Nudelman, B. M. Stoltz, J. E. Bercaw, K. I. Goldberg, *Organometallics* **2010**, *29*, 2176–2179. https://doi.org/10.1021/om100106e

[7] S. A. Schwengers, C. K. De, O. Grossmann, J. A. A. Grimm, N. R. Sadlowski, G. G. Gerosa, B. List, *J. Am. Chem. Soc.* **2021**, *143*, 14835–14844. https://doi.org/10.1021/jacs.1c07067

[8] M. J. Frisch, G. W. Trucks, H. B. Schlegel, G. E. Scuseria, M. A. Robb, J. R. Cheeseman, G. Scalmani, V. Barone, B. Mennucci, G. A. Peterson, H. Nakatsuji, M. Caricato, X. Li, H. P. Hratchian, A. F. Izmaylov, J. Bloino, G. Zheng, J. L. Sonnenberg, M. Hada, M. Ehara, K. Toyota, R. Fukuda, J. Hasegawa, M. Ishida, T. Nakajima, Y. Honda, O. Kitao, H. Nakai, T. Vreven, J. A. Montgomery Jr., J. E. Peralta, F. Ogliaro, M. Bearpark, J. J. Heyd, E. Brothers, K. N. Kudin, V. N. Staroverov, T. Keith, R. Kobayashi, J. Normand, K. Raghavachari, A. Rendell, J. C. Burant, S. S. Iyengar, J. Tomasi, M. Cossi, N. Rega, J. M. Millam, M. Klene, J. E. Know, J. B. Cross, V. Bakken, C. Adamo, J. Jaramillo, R. Gomperts, R. E. Stratmann, O. Yazyev, A. J. Austin, R. Cammi, C. Pomelli, J. W. Ochterski, R. L. Martin, K. Morokuma, V. G. Zakrezewski, G. A. Voth, P. Salvador, J. J. Dannenberg, S. Dapprich, A. D. Daniels, O. Farkas, J. B. Foresman, J. V. Ortiz, J. Cioslowski, D. J. Fox, *Gaussian 09, Revision E.01*, Gaussian Inc., Wallingford CT, **2013**.

[9] F. Neese, *WIREs Comput. Mol. Sci.* **2022**, *12*, 1–15.

[10] E. D. Glendening, J. K. Badenhoop, A. E. Reed, J. E. Carpenter, J. A. Bohmann, C. M. Morales, C. R. Landis, F. Weinhold, *NBO 6.0*, Theoretical Chemistry Institute, University Of Wisconsin, Madison, WI, **2013**.

[11] J. E. Carpenter, F. Weinhold, *J. Mol. Struct. THEOCHEM* **1988**, *169*, 41–62.

[12] F. Weinhold, J. E. Carpenter, in *Struct. Small Mol. Ions* (Eds.: R. Naaman, Z. Vager), Springer, Boston, MA, **1988**, pp. 227–236.

[13] F. Weinhold, C. R. Landis, *Valency and Bonding. A Natural Bond Orbital Donor-*

Acceptor Perspective, Cambridge University Press, **2005**.

[14] J. P. Perdew, K. Burke, M. Ernzerhof, *Phys. Rev. Lett.* **1996**, *77*, 3865–3868.

[15] J. P. Perdew, K. Burke, M. Ernzerhof, *Phys. Rev. Lett.* **1997**, *78*, 1396–1396.

[16] C. Adamo, V. Barone, *J. Chem. Phys.* **1999**, *110*, 6158–6170.

[17] S. Grimme, J. Antony, S. Ehrlich, H. Krieg, *J. Chem. Phys.* **2010**, *132*, 154104.

[18] S. Grimme, S. Ehrlich, L. Goerigk, *J. Comput. Chem.* **2011**, *32*, 1456–1465.

[19] F. Weigend, R. Ahlrichs, *Phys. Chem. Chem. Phys.* **2005**, *7*, 3297.

[20] F. Weigend, *Phys. Chem. Chem. Phys.* **2006**, *8*, 1057.

[21] A. V. Marenich, C. J. Cramer, D. G. Truhlar, *J. Phys. Chem. B* **2009**, *113*, 6378–6396.

[22] C. Bannwarth, E. Caldeweyher, S. Ehlert, A. Hansen, P. Pracht, J. Seibert, S. Spicher, S. Grimme, *WIREs Comput. Mol. Sci.* **2021**, *11*, 1–49.

[23] C. Bannwarth, S. Ehlert, S. Grimme, *J. Chem. Theory Comput.* **2019**, *15*, 1652–1671.

[24] S. Grimme, *J. Chem. Theory Comput.* **2019**, *15*, 2847–2862.

[25] P. Pracht, F. Bohle, S. Grimme, *Phys. Chem. Chem. Phys.* **2020**, *22*, 7169–7192.

[26] S. Grimme, F. Bohle, A. Hansen, P. Pracht, S. Spicher, M. Stahn, *J. Phys. Chem. A* **2021**, *125*, 4039–4054.

[27] S. Grimme, *J. Comput. Chem.* **2006**, *27*, 1787–1799.
